# HLA molecules in transplantation, autoimmunity and infection control: A comic book adventure

**DOI:** 10.1111/tan.14626

**Published:** 2022-05-15

**Authors:** Eric Reits, Jacques Neefjes

**Affiliations:** ^1^ Department of Medical Biology Amsterdam UMC Amsterdam The Netherlands; ^2^ Department of Cell and Chemical Biology ONCODE Institute, Leiden University Medical Centre LUMC Leiden The Netherlands

**Keywords:** MHC, HLA, antigen presentation, immunology, transplantation, autoimmunity

1

This article has been translated into different languages. To view the translations, please visit https://ccb.lumc.nl/comic-book-about-hla-molecules-271


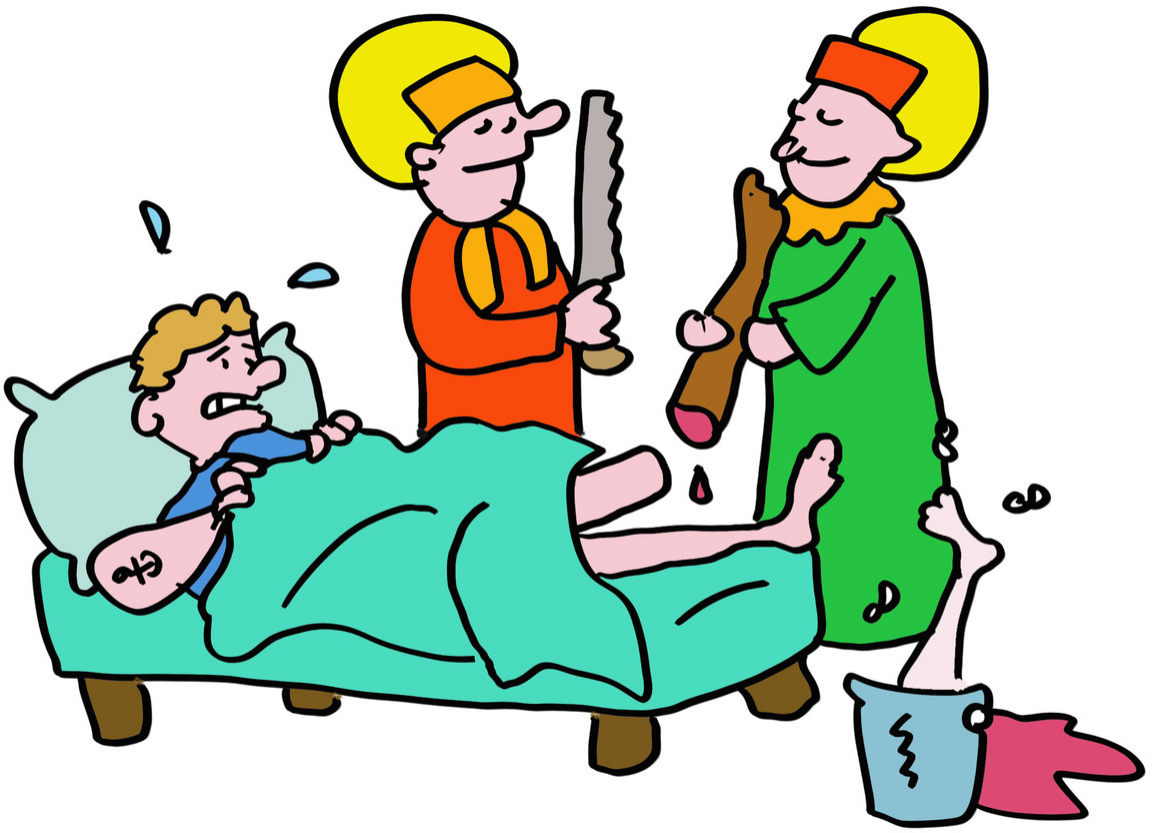
Some 1900 years ago, two Arab brothers and clinicians Cosmas and Damianus performed the first known transplantation, replacing a merchant's gangrenous leg with that of his slave. The fate of the slave is unknown in history, but it was unlikely a voluntary donation.

2


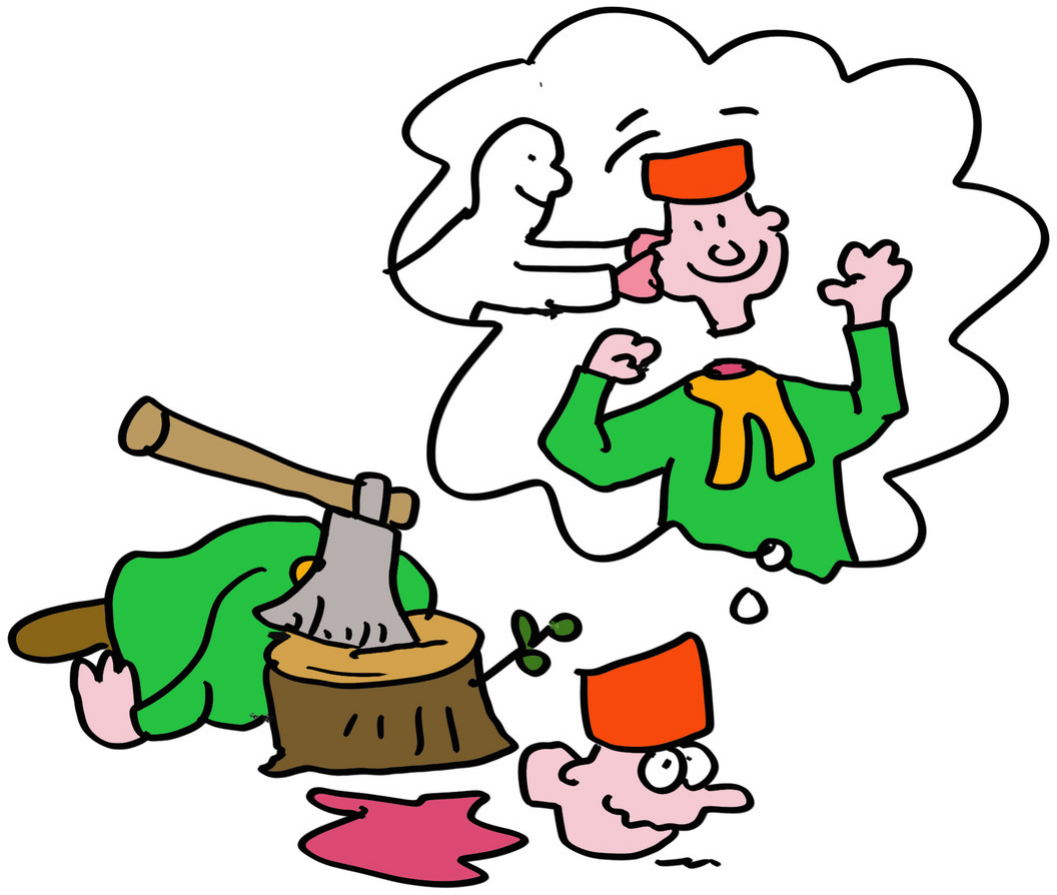
This “miraculous” transplantation contributed to their beatification, leading to their becoming the Patron Saints of transplantation. It did not hurt that they were beheaded due to their Christian faith, which was presumably corrected upon their ascension to heaven.

3


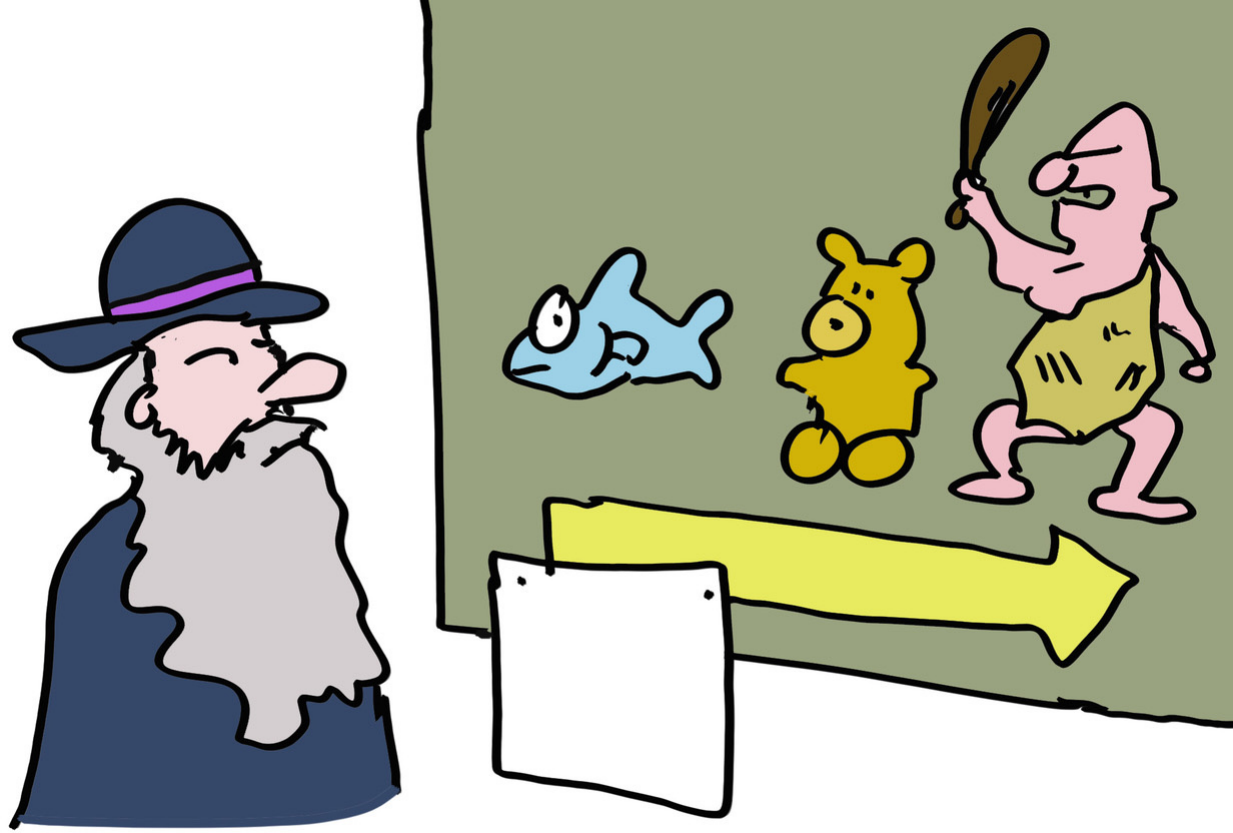
Why is transplantation so difficult, what are the evolutionary factors? Even Darwin must have wondered… but he did not know about a unique class of proteins that are expressed by almost all multicellular eukaryotes.

4


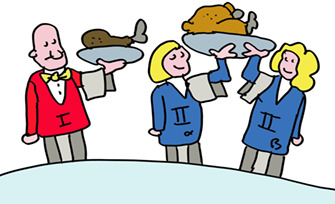
Let us start with current understanding of two unique classes of proteins in our body; the ones that have the highest degree of polymorphism (differences between individuals). And these are unique as almost all other proteins are near‐identical between humans. These polymorphic proteins are the “transplantation antigens” and are in general termed MHC class I and MHC class II molecules. In humans they are termed HLA class I and HLA class II.

5


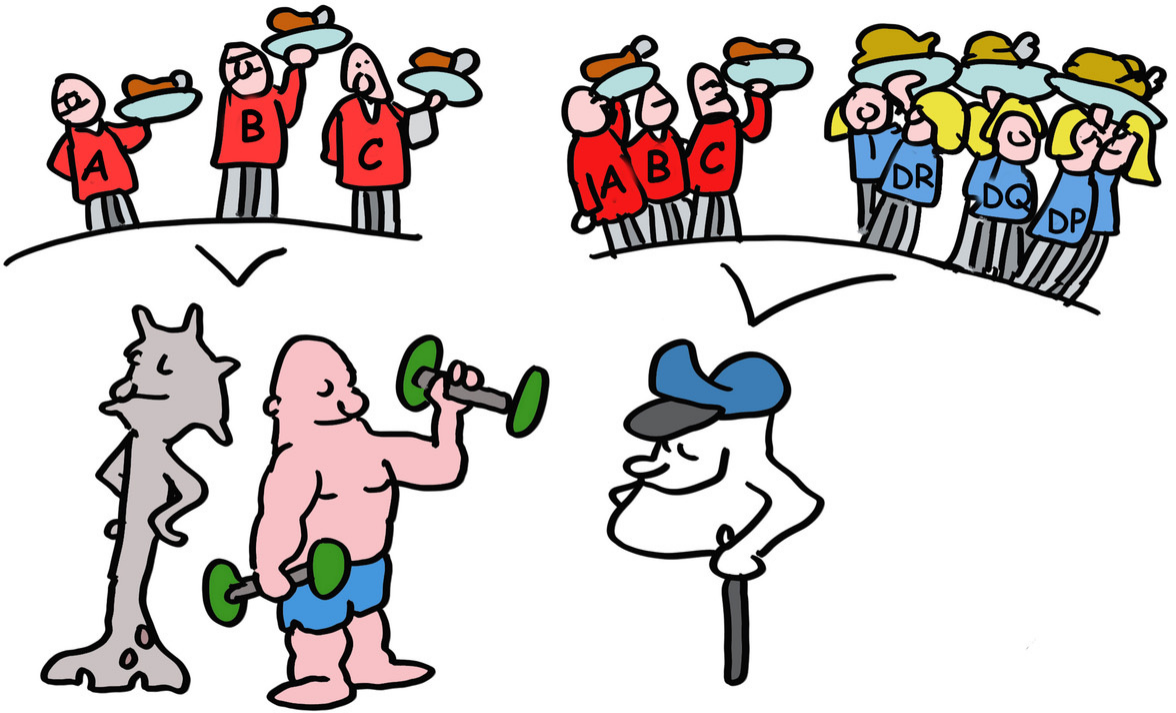
The most important HLA molecules for transplantation are termed HLA‐A, HLA‐B and HLA‐C for MHC class I and HLA‐DR, HLA‐DQ and HLA‐DP for MHC class II. HLA‐A, ‐B and ‐C are present on virtually all our cells (except red blood cells) while HLA‐DR, HLA‐DQ and HLA‐DP are mainly on immune cells.

6


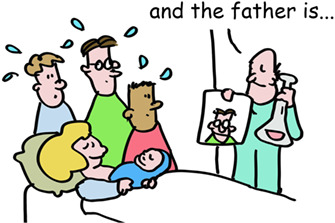
HLA molecules are so polymorphic that pregnant women often make antibodies to the different HLA types of the father. This could be used to determine the father in times before genetic testing was available. But these sera of pregnant women were also used for tissue transplantation. In workshops, sera from these women were exchanged between labs and the different serum responses named in HLA workshops. This is how HLA‐A, ‐B and ‐C were identified and different forms of these as well. These were simply numbered HLA‐A1, the next one HLA‐A2 etc. This also happened with the HLA‐DR, ‐DQ, and ‐DP molecules. So, your tissues may have (as an example) HLA‐A1, ‐B8, ‐Cw7, ‐DR3, ‐DQ2, and DPw1 proteins from your mother and HLA‐A2, ‐B27, ‐Cw1, ‐DR4, ‐DQ3, and DPw4 proteins from your father.

7


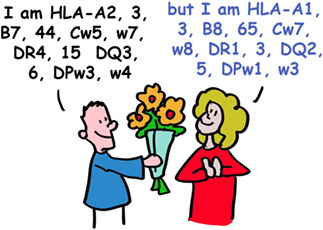
Today, HLA typing is routinely done by DNA analyses. There is some evidence that women can detect differences in men's HLA types by smell, and that this contributes to selection for genetically different mates.

8


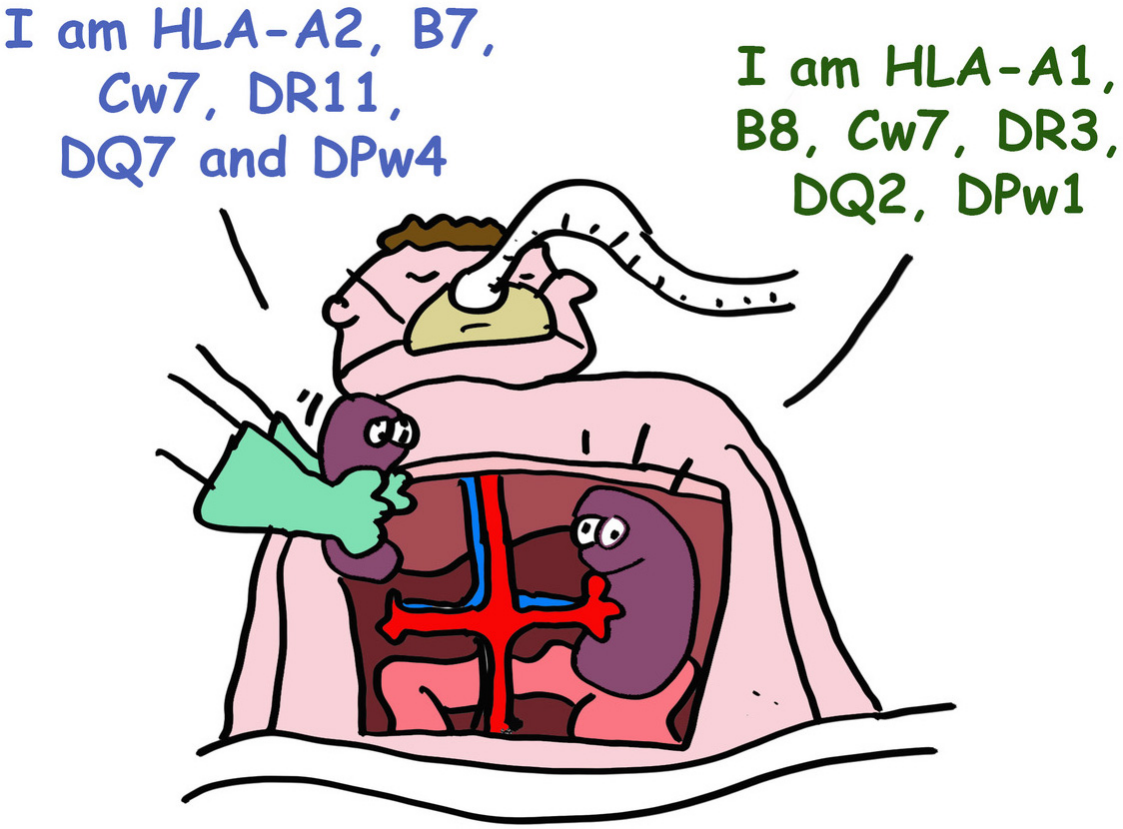


While HLA polymorphism may help diversify humanity, it is a huge barrier for successful organ transplantation, which requires matching, as closely as possible, the HLA types of the recipient and donor*. In the absence of a perfect match, effective immunosuppressive drugs are used to prevent organ rejection.

*A perfect HLA fit is very difficult to find. Family members provide the best chance (brother, sister).

9


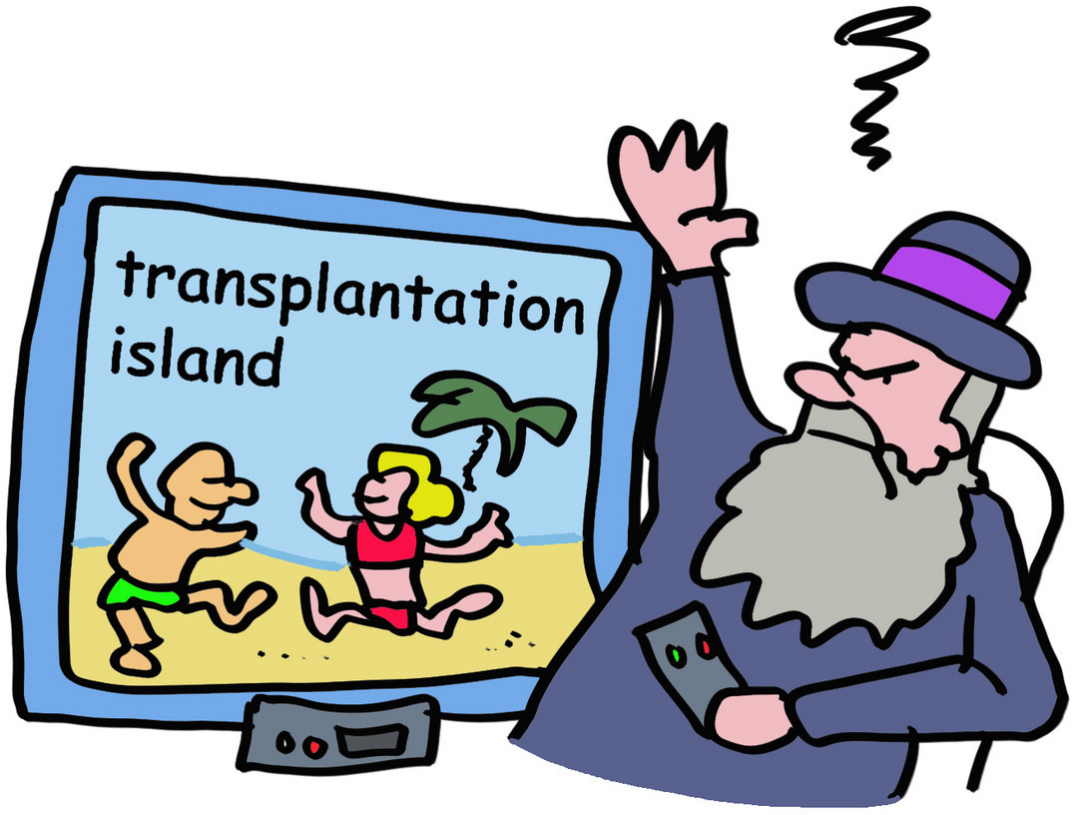
Darwin would be puzzled. Surely, smelling your perfect mate, preventing tissue transplantation, or finding the real father cannot be the principal evolutionary reasons for HLA polymorphism.

10


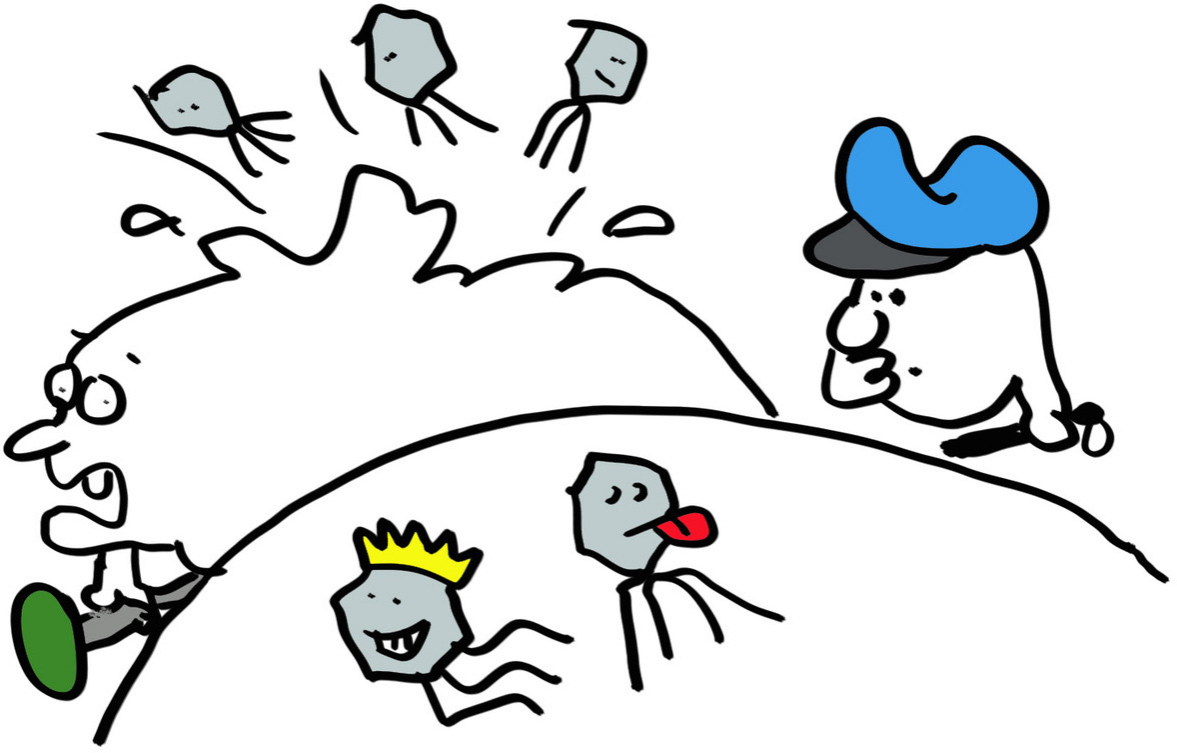
But there's another factor. Viruses and other microbial pathogens abound in nature. Corona, Influenza, Ebola, Smallpox and many other viruses use our cells to create their own families. Even “self‐limited” infections would be lethal without an immune system. And the question is simple: how can the immune system detect viruses lurking inside cells to kill them before they can kill us?

11


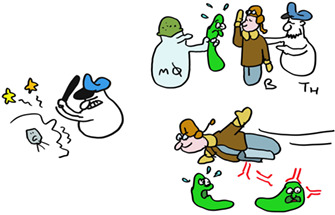
To limit damage from viruses, the immune system evolved multiple weapons. Macrophages eat bacteria and viruses, neutrophils release killing substances for bacteria, B cells make antibodies, T‐helper cells help B cells and other cells, T‐killer cells kill virus‐infected cells (and even cancer cells).

12


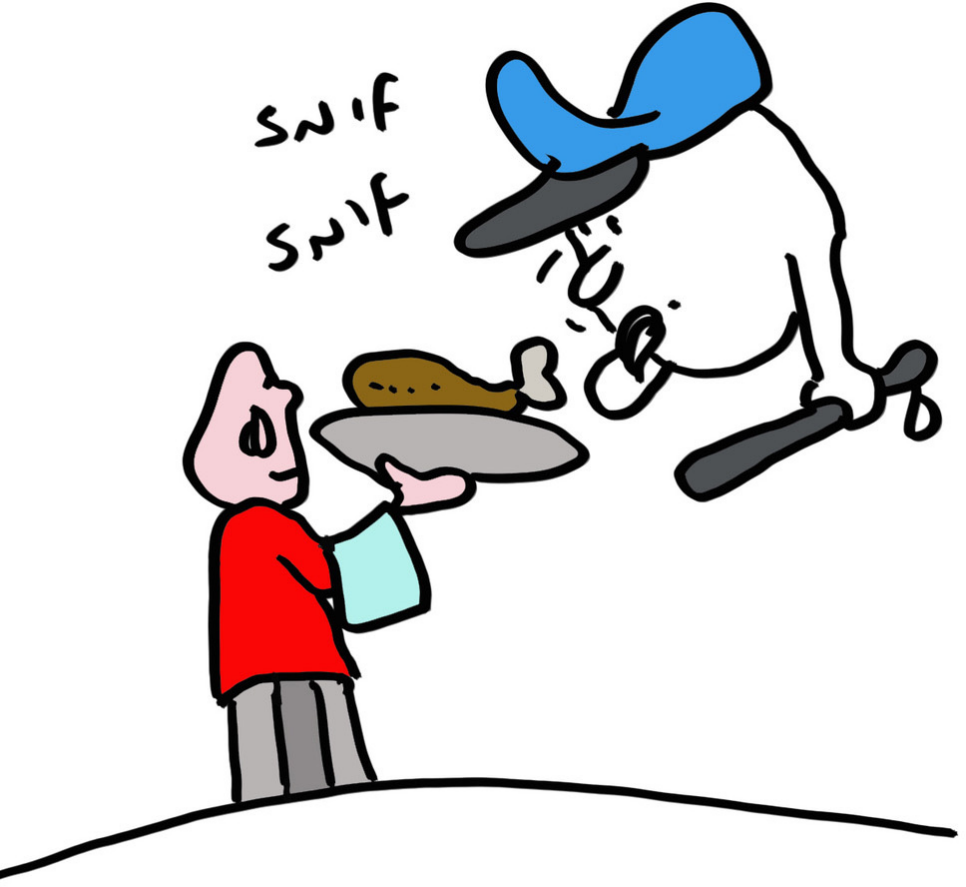
But how does a T‐killer cell know who to kill? The virus, being inside the cell, is shielded from detection, or is it? Indeed, as the virus replicates, tiny pieces of its proteins are delivered to HLA‐A, ‐B or ‐C molecules which carry them to the cell surface. The T‐killer cell recognizes this small fragment in the context of ONE specific HLA molecule. Discovering this phenomenon, termed HLA‐restriction, was sufficiently important to garner two Nobel Prizes. Each different type of MHC class I molecule presents a different repertoire of peptides to give the immune system lots of targets to aim for and kill the cells that produce these.

13


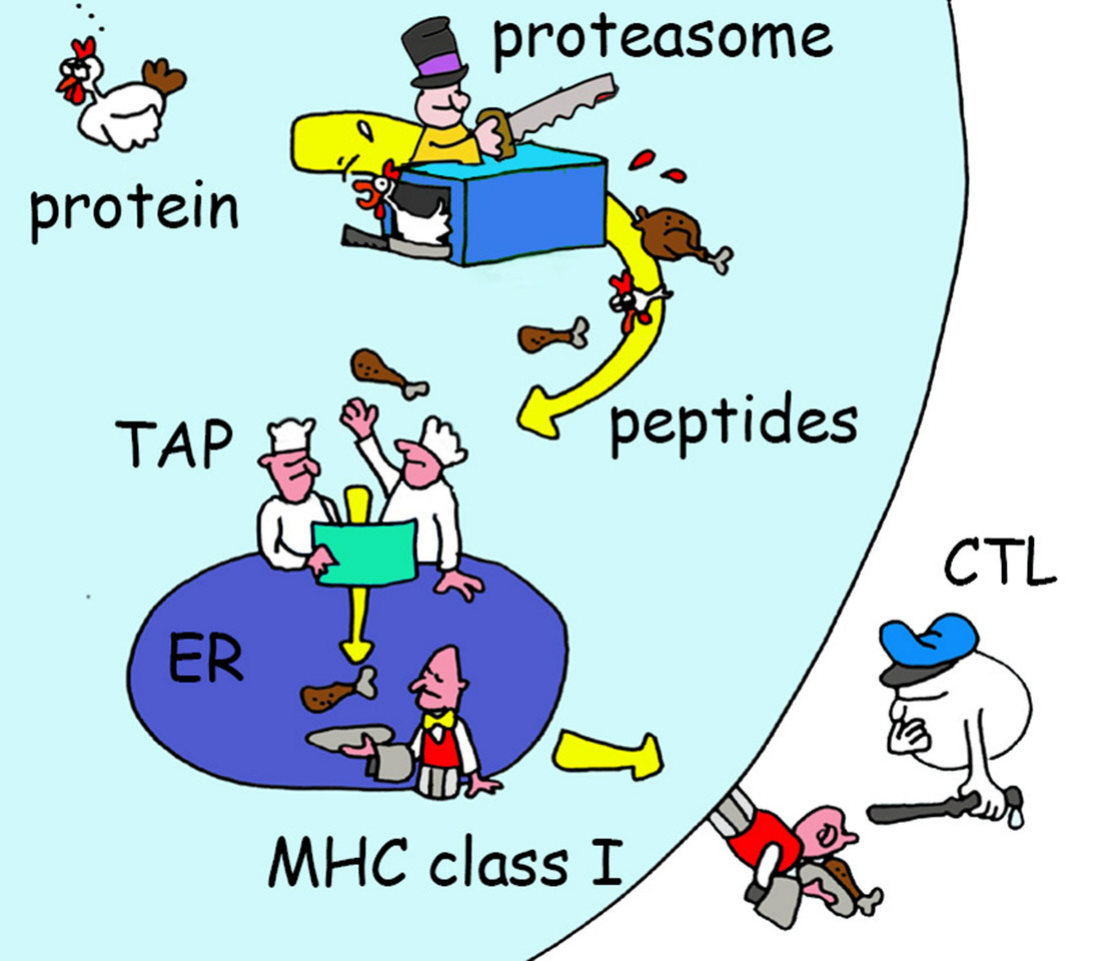
But how does a virus fragment get generated in the first place? Viral proteins ‐just like any other protein inside cells—are degraded. Proteins are fragmented by a remarkable nano‐machine called the proteasome, which is basically a DisposeAll for proteins. Other cell enzymes trim the ends of the fragments into smaller peptides, some of which are transported from the cytosol into the ER where they can bind HLA molecules. Once an HLA molecule has a bound peptide, it leaves the ER for the cell surface where it awaits detection by T‐killer cells.

14


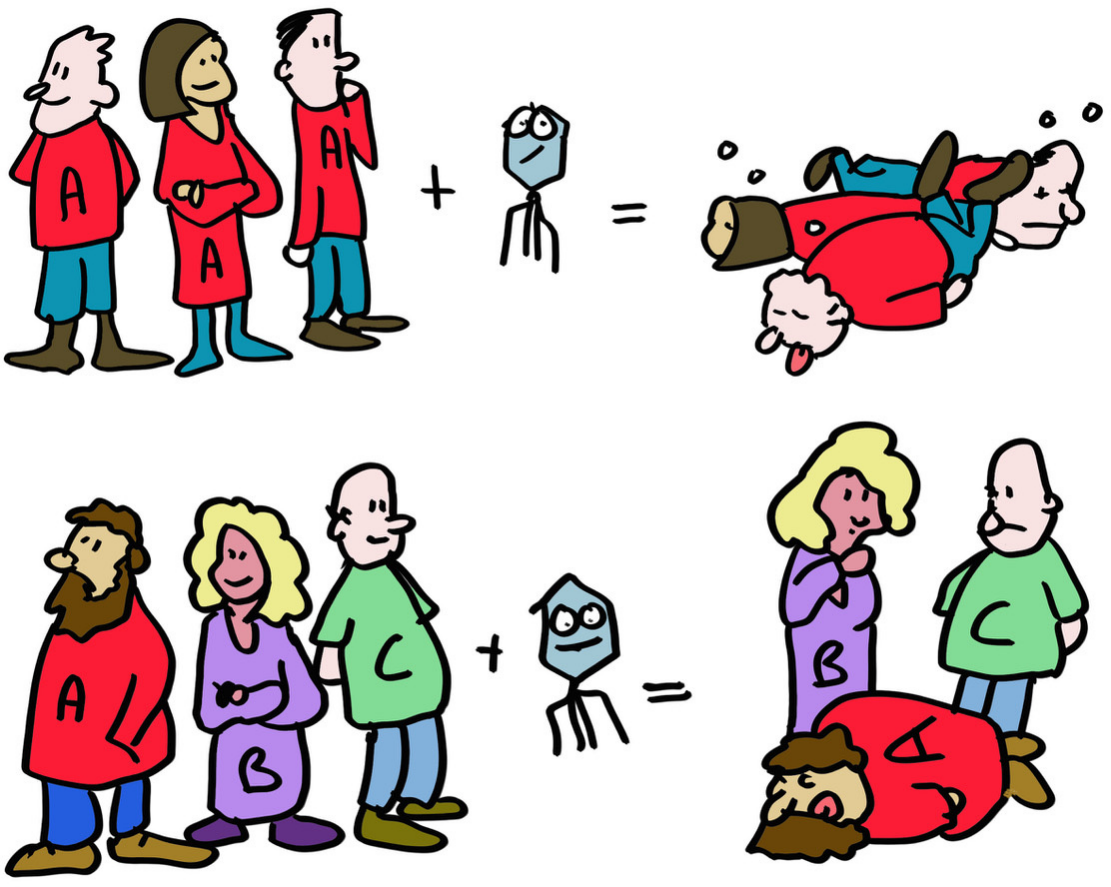
Let us get back to HLA polymorphism. As we all know from COVID‐19 and influenza, viruses are very good at changing to escape the antibody response (think alpha, delta, omicron….). To minimize this possibility for T cells, each of the different MHC alleles (varieties of genes) presents a different set of peptides. So many peptides are presented in one person, that virus evasion is difficult. The differences in HLA types between people means that even if this does happen, the evading virus will not keep up its deception in the next person. If we would all have been HLA identical, an escaping virus would kill the entire population, now it will ‘only’ kill a few individuals with HLA molecules unable to present viral peptides to the immune system. HLA polymorphism thus protects the population, the individual is less important. This provides a compelling explanation for the evolution of MHC polymorphism.

15


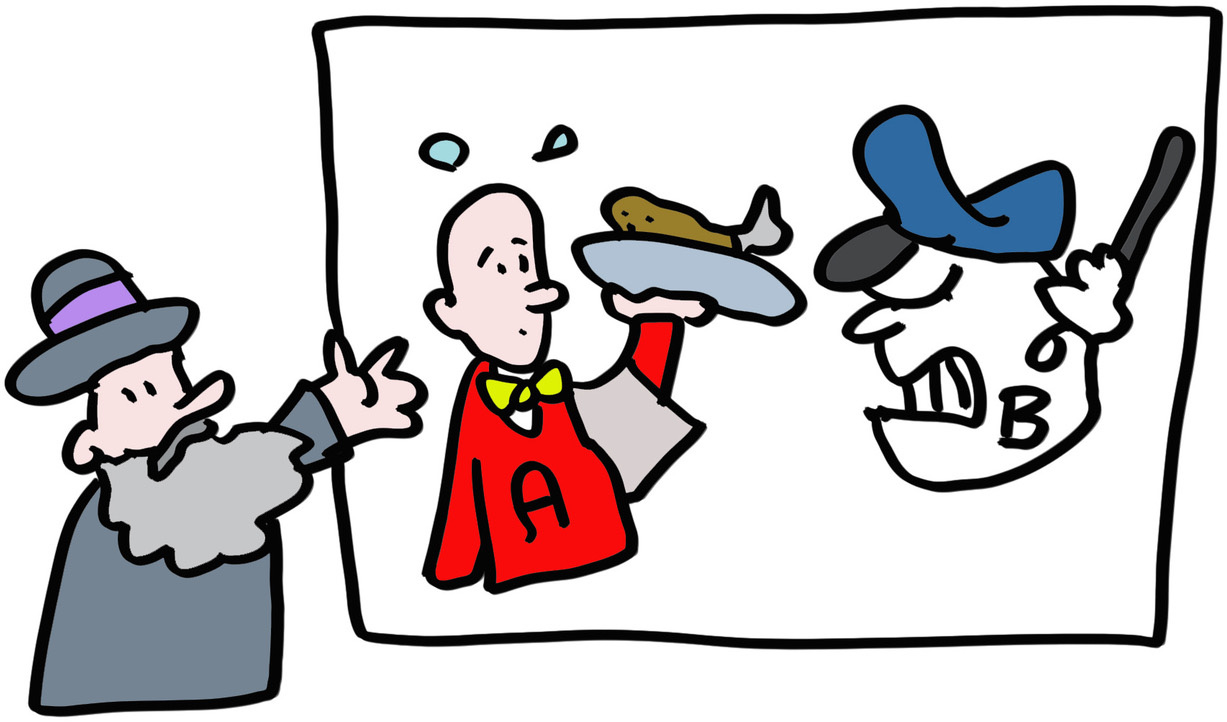
But alas, bad news for you, dear reader if you happen to need a new organ or two. HLA polymorphism promotes the survival of a species population, not an individual with kidney disease. Transplantation rejection is the consequence of the immune system confusing a donor organ with a virus infected organ and responding accordingly by attacking the organ resulting in transplant rejection.

16


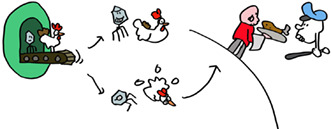
An important general lesson: nothing, including the immune system is perfect! Speaking of which, lets think about how T‐killer cells can find virus infected cells quickly enough to be of any use. Viruses can produce their offspring very quickly, in some cases in just a few hours. This is too slow to wait for viral proteins to be degraded at the end of their natural life. But just like the immune system itself, the synthesis of proteins, including viral proteins, is far from perfect. These imperfect proteins, termed DRiPs, are degraded immediately, coupling the start of virus infection to antigen presentation and allowing effective T‐killer cell immunosurveillance.

17


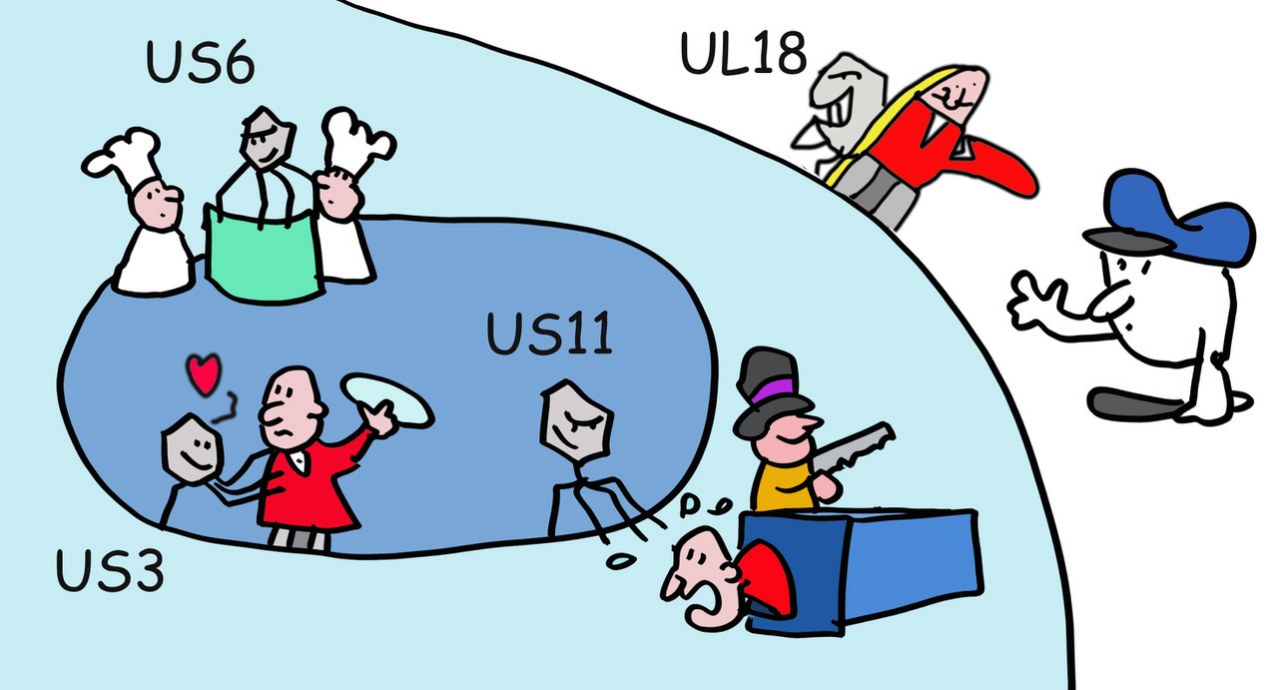
Check mate, immune system? Not so fast! Some clever viruses, especially herpesviruses, have evolved to interfere with antigen presentation. Human cytomegalovirus HCMV, which infects 60% of humanity makes a suite of proteins (US2, US3, US6, US11, and US18) that limit peptide production or interfere with HLA class I function.

18


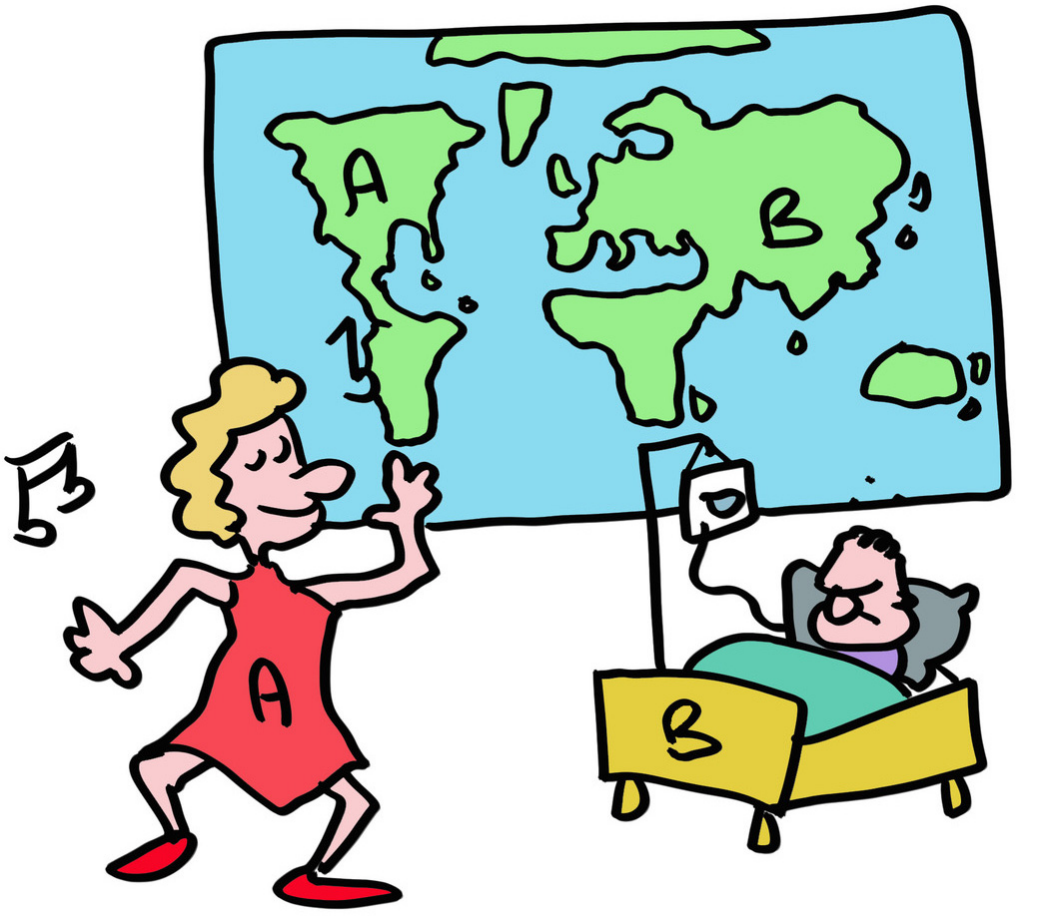
Is it then possible that some HLA alleles are better in handling virus infections than others? Indeed, some HLA‐B alleles protect better against HIV, others are better for Covid. The different HLA alleles have been selected over the eons to deal with different pathogens. For example, HLA‐A2 is found in 40% of the European population, the highest prevalence of any HLA allele in a given group. This probably results from the ability of HLA‐A2 to protect against a pathogen at one point back in time, which may well be no longer a major cause of human disease.

19


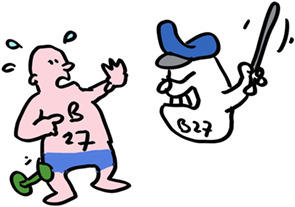
But there are collateral consequences. Take the HLA allele HLA‐B*27:05. Present in 8% of the Caucasian population, over 90% of Ankylosing Spondylitis patients have this allele, which likely triggers an autoimmune T cell reaction in the spine. The immune system operates on a knife edge between providing effective immunity to without damaging tissues from friendly fire.

20


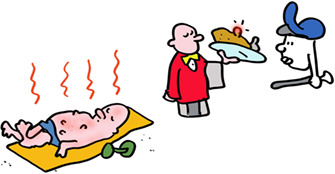
T cell autoimmunity can also be beneficial. Cancer cells typically have many mutations and other alterations that lead to generating peptides that differ from normal cellular peptides. Cancer immunotherapy exploits mechanisms used by the immune system in recognizing viral and bacterial infections to kill cancer cells.

21


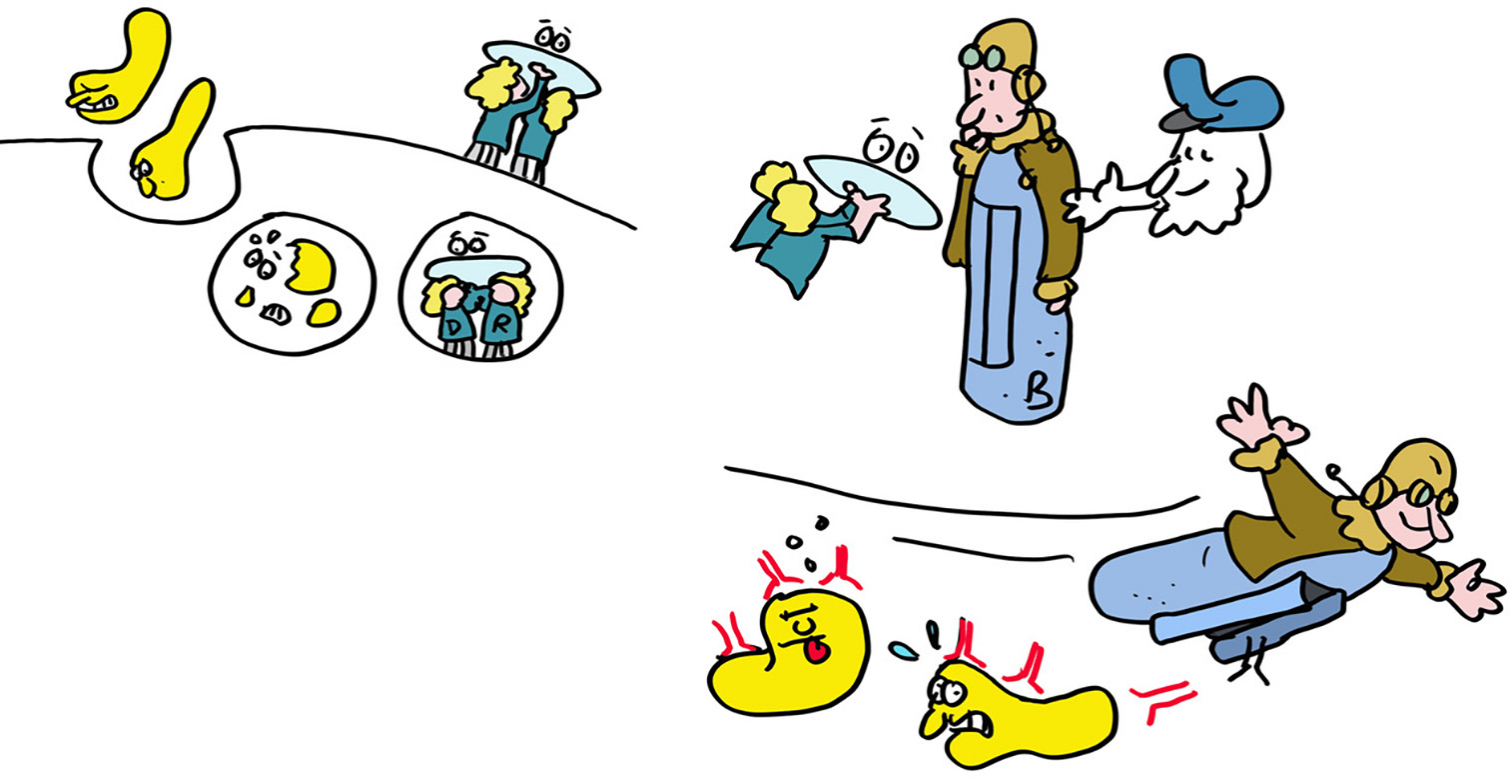
But what about the HLA‐DR, ‐DQ and ‐DP MHC class II molecules? These molecules present pathogenic peptides to T‐helper cells, which then produce cytokines to help B cells differentiate into antibody producing factories. T‐helper cells also help to optimize T‐killer responses.

MHC class II are highly similar in shape to MHC class I but present protein fragments that are longer and made in lysosomes, which are small organelles that degrade proteins acquired from outside of the cells.

22


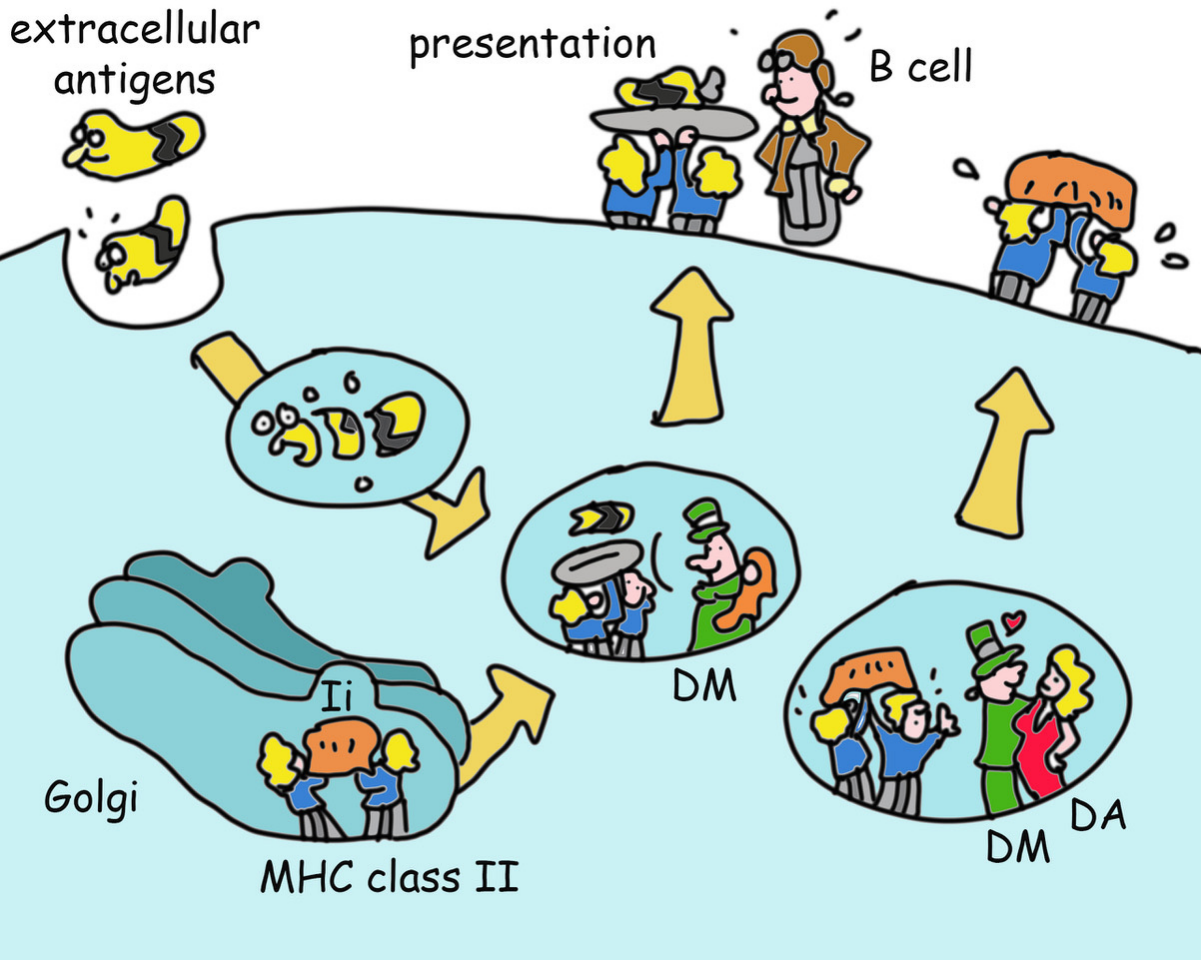
How do they do this? MHC class II is made in the ER (like any other protein that has to go to the cell's outer membrane or lysosomes) where its binds a protein (invariant chain) that mimics a peptide and chaperones MHC class II to the lysosome. Here, invariant chain is removed and exchanged for a peptide created by lysosomal enzymes. This process is optimized by yet another type of MHC molecule (HLA‐DM, which looks similar to MHC class II, and in some cells works in concert with HLA‐DO, another class II‐like molecule. Evolution is lazy, when it has developed a working module, it will simply copy and modify it for new functions). The net result of this complicated dance is the delivery of MHC class II molecules to the cell surface with peptides that enable T‐helper cell activation.

23


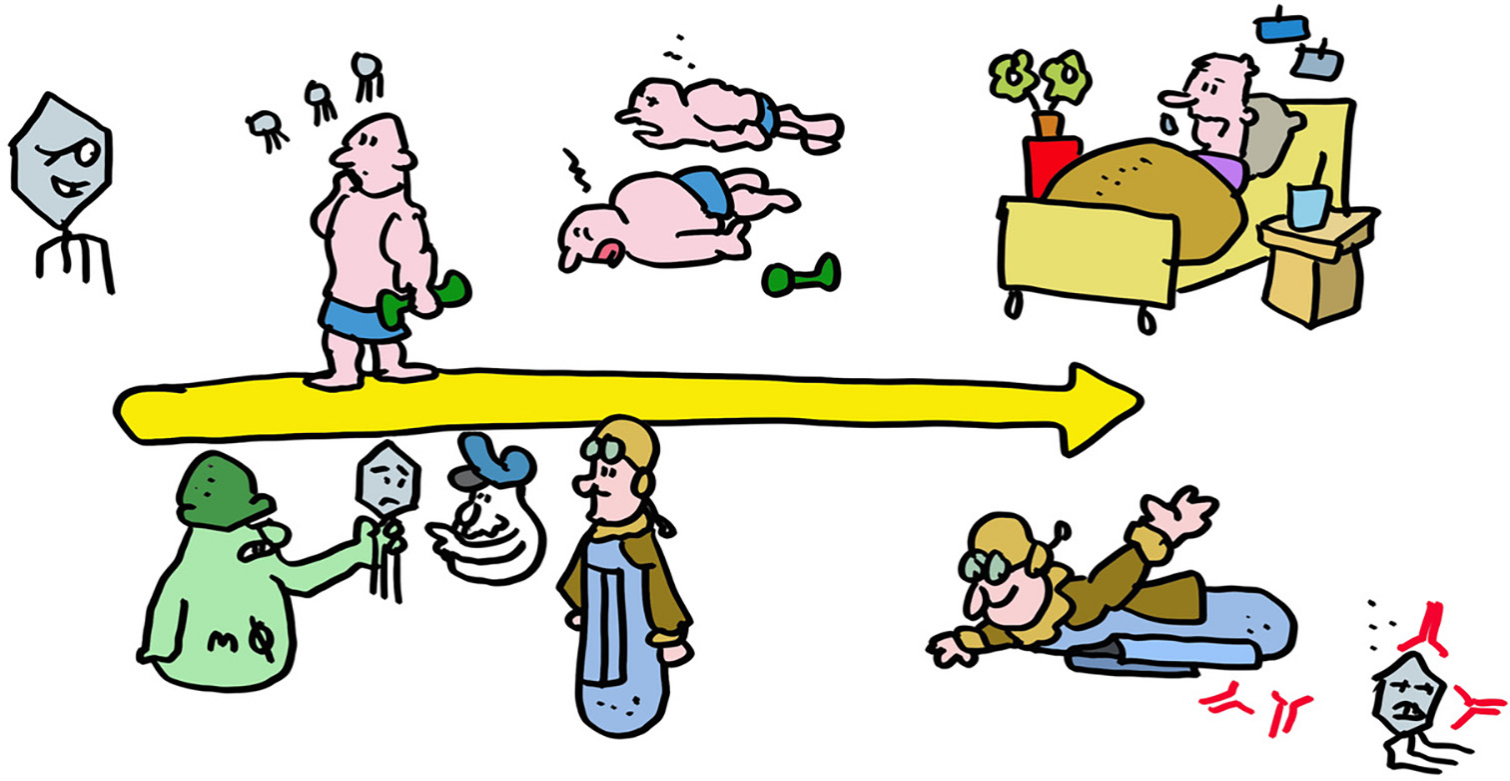
This process of pathogen recognition by the immune system is complex… but it is also relatively slow. The first time you encounter a virus, the immune system takes time to ramp up the anti‐viral response. If you are unlucky, this can result in disease or death from unchecked viral replication. Vaccination prepares the immune system for an infection, enabling it in some cases to prevent infection completely, and otherwise to respond more quickly and effectively and greatly reduce the chances of a severe infection.

24


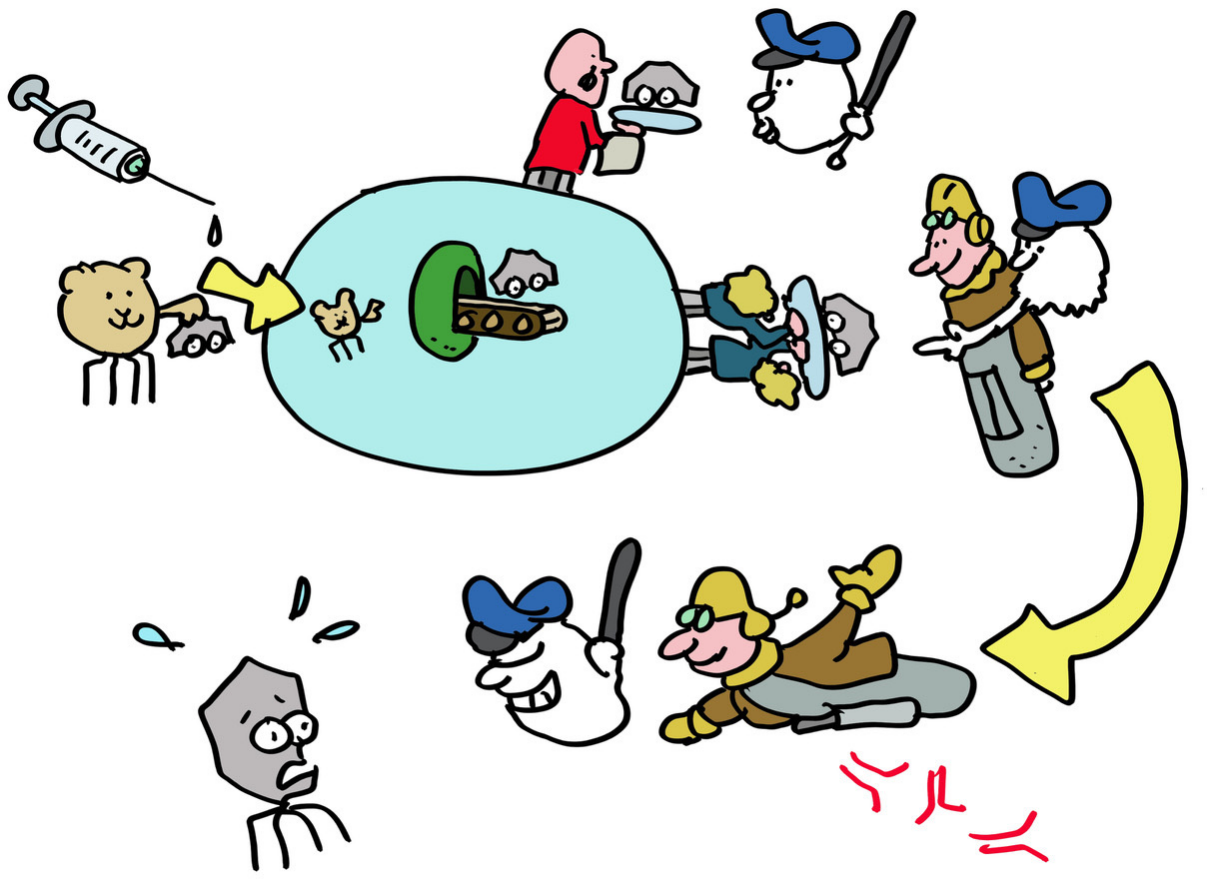
MHC molecules are critical participants in vaccination. All vaccines make use of MHC class II molecules to induce T‐helper cells needed for antibody responses and make the proteins against which the antibody responses are directed. Adenovirus and mRNA vaccines also use MHC class I molecules to induce T‐killer cells. T cells induced by vaccines last for many years, even decades in some cases, on the alert for a new infection with the original virus. Vaccines have saved far more lives than all other medical interventions combined. Spread this message, not the disease, get vaccinated!

## 25 EPILOGUE

So MHC molecules control infections, regulate immune responses and are now helping to cure cancer. This is well worth the downside of auto‐immunity and transplant rejection. And that is why you ‐living in a world filled with pathogens—survived to read this Comic Book. For more details on how to survive even better, please see references 1, 2, 3, 4, 5, 6.

## Supporting information

Supporting information.

## Data Availability

Data sharing is not applicable to this article as no new data were created or analyzed in this study.
