# Supplementary material for: HLA molecules in transplantation, autoimmunity and infection control: A comic book adventure
Source: HLA. 2022 May 15;100(4):301–11. doi: 10.1111/tan.14626 (PMC9545814; doi:10.1111/tan.14626)
Supplement: Supplementary file 1 — Supporting information. [file TAN-100-301-s001.zip › Supplementary files/PP_Bengali_Rishov Mukhopadhyay.1.pdf]

প্রতিস্থাপন, অটোইমিউনিটি এবং সংক্রমণ নিয়ন্ত্রণে HLA অনুর ভূমিকা: একটি কমিক বুক অ্যাডভেঞ্চার

HLA molecules in transplantation, autoimmunity and infection control.
A comic Book adventure.

Translated by R.Mukhopadhyay. Original text : ***https://doi.org/10.1111/tan.14626***

Department of Cell and Chemical Biology, ONCODE Institute, Leiden University Medical Centre LUMC, The Netherlands

স্লাইড १: (Slide 1)

প্রায় ৭৯00-বছর আগে, দুই আরব ভাই এবং ডাক্তার কসমাস এবং ড্যামিয়ানাস প্রথম সফল প্রতিস্থাপন করেছিলেন, একজন বণিকের গ্যাংগ্রিনাস পা তার ক্রীতদাসের সাথে প্রতিস্থাপন করেছিলেন। ক্রীতদাসের ভাগ্য ইতিহাসে অজানা, তবে এটি একটি স্বেচ্ছায় দান অসম্ভাব্য ছিল।

স্লাইড ২: (Slide 2)

এই "অলৌকিক" প্রতিস্থাপন তাদের প্রফুল্লতায় অবদান রাখে, যার ফলে তারা প্রতিস্থাপনের পৃষ্ঠপোষক সাধু হয়ে ওঠে। খ্রিস্টান বিশ্বাসের কারণে তাদের শিরশ্ছেদ করা হয়েছিল, যা সম্ভবত স্বর্গে তাদের আরোহণের সময় সংশোধন করা হয়েছিল বলে তাতে ক্ষতি হয়নি।

স্লাইড ৩: (Slide 3)

কেন প্রতিস্থাপন এত কঠিন? তা বিবর্তনীয় কারণ কি? এমনকি ডারউইনও নিশ্চয়ই অবাক হয়েছিলেন... কিন্তু তিনি এক অনন্য শ্রেণীর প্রোটিন সম্পর্কে জানতেন না যা পরে প্রকাশ করা হয়।

স্লাইড ৪: (Slide 4)

আমাদের শরীরের দুটি অনন্য শ্রেণীর প্রোটিনের কাজ বোঝার সাথে শুরু করা যাক; যেগুলো সর্বোচ্চ্য ভাবে পলিমরফিক (ব্যাক্তিত্বের মধ্যে পার্থক্য)। এবং এগুলি অনন্য, কারণ প্রায় সমস্ত অন্যান্য প্রোটিন মানুষের মধ্যে প্রায় অভিন্ন। এই পলিমরফিক প্রোটিনগুলি হল "ট্রান্সপ্লান্টেশন অ্যান্টিজেন" এবং সাধারণভাবে MHC ক্লাস I এবং MHC ক্লাস II অণু বলা হয়। মানুষের মধ্যে তাদের HLA ক্লাস I এবং HLA ক্লাস II বলা হয়।

স্লাইড ৫: (Slide 5)

প্রতিস্থাপনের জন্য সবচেয়ে গুরুত্বপূর্ণ HLA অণুগুলিকে MHC ক্লাস I এর জন্য HLA-A, HLA-B এবং HLA-C এবং MHC ক্লাস II এর জন্য HLA-DR, HLA-DQ এবং HLA-DP বলা হয়। HLA-A, -B এবং -C কার্যত আমাদের সমস্ত কোষে (লাল রক্তকণিকা ব্যতীত) উপস্থিত থাকে যখন HLA-DR, HLA-DQ এবং HLA-DP প্রধানত রোগ প্রতিরোধক কোষে থাকে।

স্লাইড ৬: (Slide 6)

HLA অণুগুলি এতটাই বহুরূপী যে গর্ভবতী মহিলারা প্রায়শই পিতার বিভিন্ন HLA-এর প্রতি এন্টিবডি তৈরী করে থাকেন। জেনেটিক পরীক্ষা উপলব্ধ হওয়ার আগে এটি পিতা নির্ধারণ করতে ব্যবহার করা যেতে পারতো। কিন্তু গর্ভবতী মহিলাদের এই সেরাগুলি টিস্যু প্রতিস্থাপনের জন্যও ব্যবহার করা হয়েছিল। কর্মশালায়, এই মহিলাদের থেকে সেরাগুলি ল্যাব এবং HLA কর্মশালায় নাম দেওয়া বিভিন্ন সিরাম প্রতিক্রিয়াগুলির মধ্যে বিনিময় করা হয়েছিল। এভাবেই HLA-A, -Bএবং -C চিহ্নিত করা হয়েছিল এবং এগুলির বিভিন্ন রূপও। এগুলিকে সহজভাবে HLA-A1, পরেরটি HLA-A2 ইত্যাদি নম্বর দেওয়া হয়েছিল। এটি HLA-DR, -DQ এবং -DP অণুর সাথেও ঘটেছে। সুতরাং, আপনার টিস্যুতে (উদাহরণস্বরূপ) আপনার মায়ের থেকে HLA-A1, -B8, -Cw7, -DR3, -DQ2, এবং DPw1 প্রোটিন থাকতে পারে এবং HLA-A2, -B27, -Cw1, -DR4, -DQ3, এবং আপনার বাবার থেকে DPw4 প্রোটিন থাকতে পারে।

স্লাইড ৭: (Slide 7)

আজকাল, HLA টাইপিং নিয়মিতভাবে ডিএনএ বিশ্লেষণের মাধ্যমে করা হয়। কিছু প্রমাণ রয়েছে যে মহিলারা গন্ধের মাধ্যমে পুরুষদের HLA- এর ধরণের মধ্যে পার্থক্য সনাক্ত করতে পারে এবং এটি জিনগতভাবে ভিন্ন সঙ্গীর জন্য নির্বাচন করতে অবদান রাখে।

স্লাইড ৮: (Slide 8)

যদিও HLA পলিমরফিজম মানবতার বৈচিত্র্য আনতে সাহায্য করতে পারে, এটি সফল অঙ্গ প্রতিস্থাপনের জন্য একটি বিশাল বাধা, যার জন্য গ্রহীতা এবং দাতার HLA ধরনের যতটা সম্ভব ঘনিষ্ঠভাবে মিলানো প্রয়োজন। একটি নিখুঁত মিলের অনুপস্থিতিতে, অঙ্গ প্রত্যাখ্যান প্রতিরোধে কার্যকর ইম্মুনোসাপ্রেসিভ ওষুধ ব্যবহার করা হয়।

স্লাইড ৯: (Slide 9)

ডারউইন হতবুদ্ধি হয়ে যাবে। অবশ্যই, আপনার নিখুঁত সঙ্গীর গন্ধ পাওয়া, টিস্যু প্রতিস্থাপন রোধ করা বা প্রকৃত পিতা খুঁজে পাওয়া HLA পলিমারফিজমের প্রধান বিবর্তনীয় কারণ হতে পারে না।

স্লাইড ১০: (Slide 10)

কিন্তু অন্য ফ্যাক্টর আছে। ভাইরাস এবং অন্যান্য মাইক্রোবায়াল প্যাথোজেন প্রকৃতিতে প্রচুর। করোনা, ইনফ্লুয়েঞ্জা, ইবোলা, স্মলপক্স এবং আরও অনেক ভাইরাস তাদের নিজস্ব পরিবার তৈরি করতে আমাদের কোষ ব্যবহার করে। এমনকি "স্ব-সীমিত" সংক্রমণ একটি ইমিউন সিস্টেম ছাড়াই প্রাণঘাতী হবে। এবং প্রশ্নটি সহজ: কীভাবে ইমিউন সিস্টেম কোষের ভিতরে লুকিয়ে থাকা ভাইরাসগুলিকে সনাক্ত করতে পারে যাতে তারা আমাদের হত্যা করার আগে আমরা তাদের হত্যা করতে পারি?

স্লাইড ১১: (Slide 11)

ভাইরাস থেকে ক্ষতি সীমিত করতে, ইমিউন সিস্টেম একাধিক অস্ত্র উদ্ভাবন করেছে। ম্যাক্রোফেজগুলি ব্যাকটেরিয়া এবং ভাইরাস শেষ করে, নিউট্রোফিলগুলি ব্যাকটেরিয়ার জন্য হত্যাকারী উপাদানগুলি নিঃসৃত করে, বি কোষগুলি অ্যান্টিবডি তৈরি করে, টি-হেল্পার কোষগুলি বি কোষ এবং অন্যান্য কোষকে সাহায্য করে, টি-কিলার কোষগুলি ভাইরাস-সংক্রমিত কোষগুলিকে (এবং এমনকি ক্যান্সার কোষগুলিকে) হত্যা করে।

স্লাইড ১২: (Slide 12)

কিন্তু টি-কিলার সেল কিভাবে জানবে কাকে হত্যা করতে হবে? সেল এর ভিতরে থাকা ভাইরাস সুরক্ষিত থাক| সত্যিই কি তাই? প্রকৃতপক্ষে, ভাইরাসের প্রতিলিপি হওয়ার সাথে সাথে, এর প্রোটিনের ক্ষুদ্র টুকরো এইচএলএ-এ, -বি বা -সি অণুতে সরবরাহ করা হয় যা তাদের কোষের পৃষ্ঠে নিয়ে যায়। টি-কিলার সেল একটি নির্দিষ্ট এইচএলএ অণুর প্রসঙ্গে এই ছোট খণ্ডটিকে স্বীকৃতি দেয়। এই ঘটনাটি আবিষ্কার করা, যাকে HLA-সীমাবদ্ধতা বলা হয়, দুটি নোবেল পুরস্কার অর্জনের জন্য যথেষ্ট গুরুত্বপূর্ণ ছিল। প্রতিটি ভিন্ন ধরনের MHC ক্লাস । অণু পেপটাইডের একটি আলাদা ভাণ্ডার উপস্থাপন করে যাতে ইমিউন সিস্টেমকে অনেক টার্গেট দেওয়া হয় যা এইগুলি তৈরি করে এবং কোষগুলিকে মেরে ফেলতে পারে।

স্লাইড ১৩: (Slide 13)

কিন্তু কিভাবে একটি ভাইরাস টুকরো প্রথম স্থানে উৎপন্ন হয়? ভাইরাল প্রোটিন - কোষের অভ্যন্তরে অন্যান্য প্রোটিনের মতোই - অবনমিত হয়। প্রোটিনগুলি প্রোটিসোম নামক একটি অসাধারণ ন্যানো-মেশিন দ্বারা খণ্ডিত হয়, যা মূলত প্রোটিনের জন্য একটি ডিস্পোজাল । অন্যান্য কোষের এনজাইম টুকরোর প্রান্তগুলিকে ছোট পেপটাইডগুলিতে ছাঁটাই করে, যার মধ্যে কিছু সাইটোসল থেকে ER- এ তে স্থানান্তরিত হয় যেখানে তারা HLA অণুগুলিকে আবদ্ধ করতে পারে। একবার একটি এইচএলএ অণুর একটি আবদ্ধ পেপটাইড হয়ে গেলে, এটি কোষের পৃষ্ঠের জন্য ER ছেড়ে যায় যেখানে এটি টি-কিলার কোষ দ্বারা সনাক্তকরণের জন্য অপেক্ষা করে।

স্লাইড ১৪: (Slide 14)

আসুন এইচএলএ পলিমারফিজমের দিকে ফিরে যাই। পাশাপাশি COVID-19 এবং ইনফ্লুয়েঞ্জা থেকে সবাই জানে, ভাইরাসগুলি অ্যান্টিবডি প্রতিক্রিয়া থেকে বাঁচতে পরিবর্তন করতে খুব ভাল (ভাবুন আলফা, ডেল্টা, ওমিক্রন...)। টি কোষের জন্য এই সম্ভাবনা কমানোর জন্য, প্রতিটি বিভিন্ন MHC অ্যালিল (জিনের বিভিন্ন প্রকার) পেপটাইডের একটি ভিন্ন সেট উপস্থাপন করে। এত পেপটাইড এক ব্যক্তির মধ্যে উপস্থাপিত হয়, যে ভাইরাস এড়ানো কঠিন। মানুষের মধ্যে HLA প্রকারের পার্থক্যের মানে হল যে এটি ঘটলেও, এড়িয়ে যাওয়া ভাইরাস পরবর্তী ব্যক্তির মধ্যে তার প্রতারণা বজায় রাখবে না। যদি আমরা সবাই এইচএলএ অভিন্ন হতাম, একটি পালিয়ে যাওয়া ভাইরাস সমগ্র জনসংখ্যাকে মেরে ফেলবে, এখন এটি 'শুধুমাত্র' এইচএলএ অণু সহ কিছু ব্যক্তিকে হত্যা করবে যা ইমিউন সিস্টেমে ভাইরাল পেপটাইড উপস্থাপন করতে অক্ষম। এইচএলএ পলিমারফিজম এইভাবে জনসংখ্যাকে রক্ষা করে, ব্যক্তি কম গুরুত্বপূর্ণ। এটি এমএইচসি পলিমারফিজমের বিবর্তনের জন্য একটি আকর্ষক ব্যাখ্যা প্রদান করে।

স্লাইড ১৫: (Slide 15)

কিন্তু হায়, আপনার জন্য খারাপ খবর, প্রিয় পাঠক আপনার যদি একটি বা দুটি নতুন অঙ্গের প্রয়োজন হয়। এইচএলএ পলিমারফিজম কিডনি রোগে আক্রান্ত ব্যক্তি নয়, একটি প্রজাতির জনসংখ্যার বেঁচে থাকার প্রচার করে। ট্রান্সপ্লান্টেশন প্রত্যাখ্যান হল ইমিউন সিস্টেমের ফলাফল যা একটি দাতা অঙ্গকে ভাইরাস দ্বারা সংক্রামিত অঙ্গের সাথে বিভ্রান্ত করে এবং অঙ্গটিকে আক্রমণ করে সেই অনুযায়ী প্রতিক্রিয়া জানায় যার ফলে প্রতিস্থাপন প্রত্যাখ্যান হয়।

স্লাইড ১৬: (Slide 16)

একটি গুরুত্বপূর্ণ সাধারণ পাঠ: ইমিউন সিস্টেম সহ কিছুই নিখুঁত নয়! যার কথা বলতে গিয়ে, আসুন চিন্তা করি কীভাবে টি-কিলার কোষগুলি ভাইরাস সংক্রামিত কোষগুলিকে খুব দ্রুত খুঁজে পেতে পারে। ভাইরাস খুব দ্রুত তাদের সন্তান উৎপাদন করতে পারে, কিছু ক্ষেত্রে মাত্র কয়েক ঘন্টার মধ্যে। ভাইরাল প্রোটিনগুলি তাদের স্বাভাবিক জীবনের শেষে ধ্বংস হবার অপেক্ষা খুবই দীর্ঘ। কিন্তু ইমিউন সিস্টেমের মতোই, ভাইরাল প্রোটিন সহ প্রোটিনের সংশ্লেষণ নিখুঁত থেকে অনেক দূরে। এই অসম্পূর্ণ প্রোটিন, যাকে DRiPs বলা হয়, অবিলম্বে ক্ষয়প্রাপ্ত হয়, ভাইরাস সংক্রমণের শুরুকে অ্যান্টিজেন উপস্থাপনের সাথে যুক্ত করে এবং কার্যকর টি-কিলার সেল ইমিউনো নজরদারির অনুমতি দেয়।

স্লাইড ১৭: (Slide 17)

কিস্তিমাত, ইমিউন সিস্টেম? এত দ্রুত না! কিছু চতুর ভাইরাস, বিশেষ করে হারপিসভাইরাস, অ্যান্টিজেন উপস্থাপনে হস্তক্ষেপ করার জন্য বিবর্তিত হয়েছে। হিউম্যান সাইটোমেগালোভাইরাস HCMV, যা ৬0% মানবতাকে সংক্রামিত করে প্রোটিনের একটি স্যুট (US2, US3, US6, US11, এবং US18) তৈরি করে যা পেপটাইড উৎপাদন সীমিত করে বা HLA ক্লাস ১ ফাংশনে হস্তক্ষেপ করে।

স্লাইড ১৮: (Slide 18)

তাহলে কি এটা সম্ভব যে কিছু এইচএলএ অ্যালিল অন্যদের তুলনায় ভাইরাস সংক্রমণ মোকাবেলায় ভালো? প্রকৃতপক্ষে, কিছু এইচএলএ-বি অ্যালিল এইচআইভি থেকে আরও ভাল সুরক্ষা দেয়, অন্যগুলি কোভিডের জন্য ভাল। বিভিন্ন রোগজীবাণু মোকাবেলা করার জন্য যুগে যুগে বিভিন্ন HLA অ্যালিল নির্বাচন করা হয়েছে। উদাহরণস্বরূপ, HLA-A2 ইউরোপীয় জনসংখ্যার 80% এর মধ্যে পাওয়া যায়, একটি নির্দিষ্ট গোষ্ঠীতে যে কোনো HLA অ্যালিলের সর্বাধিক প্রকোপ। এটি সম্ভবত HLA-A2-এর ক্ষমতার ফলে এক সময়ে একটি রোগজীবাণু থেকে রক্ষা করতে পারে, যা আর মানুষের রোগের প্রধান কারণ হতে পারে না।

স্লাইড ১৯: (Slide 19)

কিন্তু সমান্তরাল পরিণতি আছে। HLA অ্যালিল এইচএলএ–বি*২৭:0৫ নিন। ককেশীয় জনসংখ্যার ৮% এর মধ্যে উপস্থিত, ৯0% এর বেশি অ্যাঙ্কাইলোজিং স্পন্ডিলাইটিস রোগীদের এই অ্যালিল থাকে, যা সম্ভবত মেরুদণ্ডে একটি অটোইমিউন টি সেল প্রতিক্রিয়া শুরু করে। ইমিউন সিস্টেম বন্ধুত্বপূর্ণ আগুন থেকে টিস্যুগুলির ক্ষতি না করে কার্যকর অনাক্রম্যতা প্রদানের মধ্যে একটি ছুরির ধারে কাজ করে

স্লাইড ২০: (Slide 20)

টি সেল অটোইমিউনিটিও উপকারী হতে পারে। ক্যান্সার কোষে সাধারণত অনেক মিউটেশন এবং অন্যান্য পরিবর্তন থাকে।এরা পেপটাইড তৈরি করে, যা সাধারণ সেলুলার পেপটাইড থেকে আলাদা। ক্যান্সার ইমিউনোথেরাপি ক্যান্সার কোষকে হত্যা করার জন্য ভাইরাল এবং ব্যাকটেরিয়া সংক্রমণ সনাক্ত করতে ইমিউন সিস্টেম দ্বারা ব্যবহৃত প্রক্রিয়াগুলিকে কাজে লাগায়।

স্লাইড ২১: (Slide 21)

কিন্তু HLA-DR, -DQ এবং -DP MHC ক্লাস II অণু সম্পর্কে কি? এই অণুগুলি টি-হেল্পার কোষগুলিতে প্যাথোজেনিক পেপটাইডগুলি উপস্থাপন করে, যা পরে বি কোষগুলিকে অ্যান্টিবডি তৈরির কারখানায় পার্থক্য করতে সাহায্য করার জন্য সাইটোকাইন তৈরি করে। টি-হেল্পার সেলগুলি টি-কিলার প্রতিক্রিয়াগুলিকে ঠিক করতেও সাহায্য করে।

MHC ক্লাস II এর আকারের সাথে MHC ক্লাস I-এর অত্যন্ত মিল রয়েছে কিন্তু বর্তমান প্রোটিন টুকরা যা লম্বা এবং লাইসোসোমে তৈরি, যা ছোট অর্গানেল যা কোষের বাইরে থেকে অর্জিত প্রোটিনগুলিকে হ্রাস করে।

স্লাইড ২২: (Slide 22)

তারা এটা কিভাবে করে? MHC ক্লাস II ER-তে তৈরি করা হয় (কোষের বাইরের ঝিল্লি বা লাইসোসোমে যেতে হয় এমন অন্য প্রোটিনের মতো) যেখানে এটি একটি প্রোটিন (অপরিবর্তনশীল শৃঙ্খল) আবদ্ধ করে যা একটি পেপটাইডের অনুকরণ করে এবং MHC ক্লাস II কে চাপেরণের দ্বারা লাইসোসোমের দিকে প্রসারিত করে। এখানে, অপরিবর্তনীয় চেইন অপসারণ করা হয় এবং লাইসোসোমাল এনজাইম দ্বারা তৈরি একটি পেপটাইডের জন্য বিনিময় করা হয়। এই প্রক্রিয়াটি অন্য ধরনের MHC অণু (HLA-DM, যা দেখতে MHC ক্লাস II-এর মতো, এবং কিছু কোষে HLA-DO-এর সাথে মিলিতভাবে কাজ করে, আরেকটি ক্লাস II-এর মতো অণু দ্বারা ঠিক করা হয়। বিবর্তনের পদ্ধতি ধীর, এটি যখন একটি কার্যকরী শাখা তৈরী করে তখন সেটিকে শুধুমাত্র অনুলিপি এবং সংশোধন করেই কাজ চালায়। নতুন আরেকটি শাখা তৈরী করতে এরা অলস। এই জটিল নৃত্যের নেট ফলাফল হল পেপটাইড সহ MHC ক্লাস II অণু কোষের পৃষ্ঠে সরবরাহ করা যা টি-হেল্পার সেল সক্রিয়করণে সক্ষম করে।

স্লাইড ২৪: (Slide 24)

MHC অণুগুলি টিকাদানে গুরুত্বপূর্ণ অংশগ্রহণকারী। সমস্ত ভ্যাকসিন এমএইচসি ক্লাস ॥ অণু ব্যবহার করে অ্যান্টিবডি প্রতিক্রিয়ার জন্য। প্রয়োজনীয় টি-হেল্পার কোষগুলিকে প্ররোচিত করে এবং প্রোটিন তৈরি করে যার বিরুদ্ধে অ্যান্টিবডি প্রতিক্রিয়া নির্দেশিত হয়। অ্যাডেনোভাইরাস এবং এমআরএনএ ভ্যাকসিনগুলিও টি-কিলার কোষগুলিকে প্ররোচিত করতে MHC ক্লাস । অণু ব্যবহার করে। ভ্যাকসিন দ্বারা প্ররোচিত টি কোষগুলি মূল ভাইরাসের সাথে একটি নতুন সংক্রমণের জন্য সতর্কতার জন্য অনেক বছর, এমনকি কিছু ক্ষেত্রে কয়েক দশক ধরে চলে। ভ্যাকসিনগুলি অন্যান্য সমস্ত চিকিৎসা হস্তক্ষেপের চেয়ে অনেক বেশি জীবন বাঁচিয়েছে। এই বার্তা ছড়িয়ে দিন, রোগ নয়, টিকা নিন!

পরিশেষ: (Epilogue)

তাই MHC অণু সংক্রমণ নিয়ন্ত্রণ করে, রোগ প্রতিরোধ ক্ষমতা নিয়ন্ত্রণ করে এবং এখন ক্যান্সার নিরাময়ে সাহায্য করছে। এটি অটো-ইমিউনিটি এবং ট্রান্সপ্লান্ট প্রত্যাখ্যানের নেতিবাচক দিকগুলির জন্য ভাল। সেই কারণেই আপনি, জীবাণুতে ভরা এই পৃথিবীতে বাস করছেন এবং এই কমিক বইটি জীবিত অবস্থায় পড়তে পারছেন । কীভাবে আরও ভালভাবে বেঁচে থাকা যায় সে সম্পর্কে আরও বিশদ বিবরণের জন্য, অনুগ্রহ করে রেফারেন্স ১-৬ দেখুন।
