# Supplementary material for: HLA molecules in transplantation, autoimmunity and infection control: A comic book adventure
Source: HLA. 2022 May 15;100(4):301–11. doi: 10.1111/tan.14626 (PMC9545814; doi:10.1111/tan.14626)
Supplement: Supplementary file 1 — Supporting information. [file TAN-100-301-s001.zip › Supplementary files/PP_Chinese_Tan_Xu.1.pdf]

移植、自身免疫和感染控制中的人类白细胞抗原分子：漫画书的冒险

HLA molecules in transplantation, autoimmunity and infection control. A comic Book adventure

by

Eric Reits and Jacques Neefjes

Translated by Minkang Tan and Ningning Xu Original text : ***https://doi.org/10.1111/tan.14626***

Department of Cell and Chemical Biology, ONCODE Institute, Leiden University Medical Centre LUMC, The Netherlands

幻灯片 1

大约 1900 **年前，两个阿拉伯兄弟和**临床医生Cosmas 和 Damianus 进行了第**一次已知的移植手**术，即用一个商人的奴隶的腿代替了商人的坏疽腿。 这个**奴隶的命运在**历史上无从考证，但这不太可能是一次自愿捐赠。

幻灯片 2

这种“**奇迹般的**”**移植促成了他**们的宣福礼，使他们成为移植的守护圣徒。**由于他**们的基督教信仰，即便他们被斩首，也不会有很大的影响，因为在他们升天后这很可能得到纠正。

幻灯片 3

为什么移植这么难，进化的因素有哪些？**即使是达**尔文也一定想过……**但他不知道有一**类独特的蛋白质，几乎所有的多细胞真核生物都表达这种蛋白质。

幻灯片 4

让我们从目前对我们体内两类独特蛋白质的了解开始，它们具有极高程度的**多**态性（个体间存在差异）。**它**们是独一无二的，因为所有其他蛋白质在人类之间是几乎相同的。这些多态性蛋白质就是“**移植抗原**”，**通常被称**为 MHC I 类和 MHC II 类分子。在人类中，它们被命名为人类白细胞抗原（以下简称HLA）I 类和 II 类。

幻灯片 5

用于移植最重要的 HLA 分子在MHC I 类中被称为HLA-A, HLA-B 和HLA-C, 在MHC II 类中被称为HLA-DR, HLA-DQ 和HLA-DP。HLA-A, HLA-B 和 HLA-C 几乎存在于我们所有的细胞中（红细胞除外）， 而 HLA-DR, HLA-DQ 和 HLA-DP 主要存在于免疫细胞中。

幻灯片 6

HLA 分子是如此多态，以至于孕妇经常产生针对父亲的不同 HLA 类型的抗体。 在基因检测可用之前，这种方法可以用来确定谁是孩子的父亲。但是这些孕妇的血清也被用于组织移植。 在研讨会上，这些女性的血清在实验室之间被交换，不同的血清反应也在 HLA 研讨会中被命名。 这就是 HLA-A, -B 和 -C以及它们的不同形式被识别的方式。 它们被简单地编号为 HLA-A1， 下一个是 HLA-A2 等等。这种命名方式也被用在 HLA-DR, -DQ 和 -DP 分子上。 因此，你的组织可能有（例如）来自母亲的HLA-A1, -B8, -Cw7, -DR3, -DQ2和DPw1蛋白质以及来自父亲的HLA-A2， -B27， -Cw1， -DR4， -DQ3和DPw4蛋白质。

幻灯片 7

如今，HLA 分型通常通过 **DNA 分析完成。** **有**证据表明，女性可以通过气味检测男性 HLA 类型的差异，这有助于选择基因型不同的配偶。

幻灯片 8

虽然 **HLA 多**态性可能有助于人类多样化，但它是器官移植成功的巨大障碍, **成功的器官移植需要受体和供体的 HLA** 类型匹配，并且尽可能地接近。 **在缺乏完美匹配的情况下，有效的免疫抑制**药物可以被用来预防器官排斥。

幻灯片 9

达尔文**会感到困惑。当然，嗅出你的完美伴**侣，阻止组织移植，或者找到**真正的父**亲，不可能是**HLA多**态性的主要进化原因。

幻灯片 10

但还有另一个因素。 **病毒和其他微生物病原体在自然界中大量存在。 冠状病毒、流感病毒、埃博拉病毒、天花病毒和**许多其他病毒都利用我们的细胞来创造它们自己的家族。 **如果没有免疫系**统，即使是“**自限性**”**的感染也是致命的。然后**问题很简单：**免疫系**统是如何检测潜伏在细胞内的病毒，在它们杀**死我**们之前杀死它们?

幻灯片 11

为了减轻病毒的伤害，免疫系统进化出多种武器。巨噬细胞吞噬细菌和病毒，**中性粒**细胞释放针对细菌的杀伤物质，B细胞制造抗体，辅助性T细胞帮助B细胞和其他细胞，杀伤性T细胞杀死被病毒感染的细胞(**甚至癌**细胞)。

幻灯片 12

但杀伤性T细胞**是如何知道要**杀死谁呢？细胞内部的病毒是不会被发现吗？事实上，随着病毒的复制，其蛋白质的微小片段会被传递给**HLA-A、-B或-C分子**，这些分子将它们带到细胞表面。杀伤性T细胞**会在特定的HLA分子的背景下**识别这个小片段。这种被称之为**“HLA限制”的**现象的发现，其重要性足以获**得两个**诺贝尔奖。每种不同类型的**MHC I**类分子都会呈递不同的多肽组合，使**免疫系**统有很多目标可以瞄准，并且杀死产生这些多肽的细胞。

幻灯片 13

但是病毒片段首先是如何产生的呢？病毒的蛋白质就像细胞内的任何其他蛋白一样是会被降解的。病毒蛋白质被一种出色的纳米机器**——蛋白酶体分解成片段，而蛋白酶体基本**上可以处理所有蛋白质。其他细胞酶将片段的末端**修剪成更小的**肽，其中一些肽从细胞溶胶中被运送到内质网，在那里它们可**以与HLA分子**结合。一旦**HLA分子与来自病毒的**肽结合，它就会离开内质网到细胞表面，在**那里等待**杀伤性T细胞的检测。

幻灯片 14

让我们回到HLA多态性的问题， **正如大家从COVID-19和流感中知道的那样，病毒非常善于改**变以逃避抗体反应（想想新型冠状病毒亚种 alpha，delta，omicron....）。为了最小化T细胞检测不到病毒抗原的可能性，每个不同的MHC**等位基因**（**基因的**变种）都呈递出一组不同的肽。一个人身上有如此多**的**肽，因此病毒很难逃逸。人与人之间HLA类型的差异意味着，即使这种情**况真的**发生，**逃逸的病毒也不会在下一个人身上**继续欺骗杀伤性T细胞。**如果我**们所有人都是相同HLA**基因型**，**逃逸的病毒会**杀死整个群体。现在它"**只**"杀**死少数个体**，这些个体的HLA**分子无法向免疫系**统呈递病毒肽。因此，HLA多态性保护了整个群体，个人就不那么重要了。这为MHC**多**态性的进化提供了**一个令人信服的解**释。

幻灯片 15

但对于亲爱的读者可惜的是，如果你碰巧需要一两个新的器官，这对你来说**将是个坏消息。HLA多**态性促进了一个物种群体的生存，而不是患有肾脏疾**病的个体。移植排斥是免疫系**统将捐赠器官与受病毒感染的器官相混淆，并**作出相**应的反应来攻击移植器官，从而导致的后果。

幻灯片 16

一个重要且普遍的教训是：没有什么东西是完美的，甚至包括免疫系统！提**到**这点，让我们想一想，杀伤性T细胞是**如何能足**够迅速地找到被病毒感染的细胞从而发挥作用的。病毒繁殖后代速度非常快，在某些情况下只需要几个**小**时。但病毒繁殖的过程相对于等待病毒蛋白在其自然生命结束时被降解来说还是太慢了。但就像免疫系统本身一样，蛋白质的合成，包括病毒蛋白质**的合成**，远非完美。这些不完美的蛋白质，被称为DRiPs，**会被立即降解**，**将病毒感染的开始与抗原呈**递联系起来，**并且允**许有效的T细胞免疫监督。

幻灯片 17

检查伴侣，免疫系统？没那么快! **一些**聪明的病毒，特别是疱疹病毒，已经进**化到可以干**扰抗原呈递。人类巨细胞病毒HCMV**感染了**60%**的人**类，它制造**了一套蛋白**质（US2、US3、US6、US11和US18），**限制**肽的产生或干扰HLA I类分子功能。

幻灯片 18

那么是否有可能某些HLA等位基因在处理病毒感染方面比其他的更好？的确，**有些HLA-B等位基因**对**HIV有更好的保**护作用，而其他的则对**Covid有更好的保**护作用。不同的**HLA等位基因在**过去的岁月里被选择来处理不同的病原体。**例如，**HLA-A2**在40%的欧洲人口中被**发现，是在特定群体中的最为流行**的HLA等位基因。**这可能是由于在过去的某个时间点**HLA-A2有能力抵御某种病原体，而**这种病原体很可能已经不再是人类疾病的主要原因。

幻灯片 19

但也有附带的后果。以**HLA等位基因**HLA-B*27:05为例，它存在于**8%的高加索人口中，超过**90%**的**强直性脊柱炎患者有这种等位基因，这很可能引发脊**柱的自身免疫性**T细胞反应。免疫系统在提供有效的免疫力与不破坏组织的友军射击之间的刀刃上运行。

幻灯片 20

T细胞的自身免疫也可以是有益的。癌细胞通常有许多突变和其他改变，导致**生成的**肽与正常细胞的肽不同。癌症免疫疗法就是利用免疫系统使用的识别**病毒和**细菌感染的机制，来杀死癌细胞。

幻灯片 21

但是HLA-DR、-DQ和-DP MHC II类分子呢？这些分子将致病肽呈递给辅助性T细胞，然后辅助性T细胞产生细胞因子，帮助B细胞分化为抗体生产工厂。辅助性T细胞还帮助优化杀伤性T细胞反应。

MHC II类分子在形状上与MHC I类分子高度相似，但呈递的蛋白质片段较长且在溶酶体中制造，而溶酶体是降解从细胞外获得的蛋白质的小细胞器。

幻灯片 22

它们是如何做到这一点的？**MHC II**类分子是在内质网中制造的（就像其他必须进入细胞外膜或溶酶体的蛋白质一样），**在那里它与一种模仿**肽的蛋白质（**不**变链）结合，并将**MHC II**类伴侣送到溶酶体中。在这里，不变链被移除**并与溶酶体**创造的肽交换。这一过程由另一种类型的**MHC分子**（**HLA-DM,看起来与**MHC II类相似，在一些细胞中与**HLA-DO**，**另一种**II类分子协同工作）进行优化。进化是懒惰的，当它已经开发了一个工作模块，它将简单地复**制和修改以**获得新的功能）。这种复杂的舞蹈的最终结果是将**MHC II**类分子**与**肽一起送到细胞表面，使辅助性**T**细胞得到激活。

幻灯片 23

免疫系统识别病原体的这一过程很复杂……**但它也相**对缓慢。当你第一次遇到**病毒**时，免疫系统需要时间来加强抗病毒反应。如果你不走运，由于病毒复**制不受控制**，这可能会导致疾病或死亡。疫苗接种使免疫系统为感染做好准备，在某些情况下能够完全防止感染，或者是免疫系统会更快，更有效地作**出反**应，大大减少严重感染的可能性。

幻灯片 24

MHC分子是疫苗接种的关键参与者。所有疫苗都利用**MHC II**类分子来诱导抗**体反**应所需的辅助性T细胞，并制造抗体反应所针对的蛋白质。腺病毒和mRNA**疫苗也使用**MHC I类分子来诱导杀伤性**T**细胞。疫苗诱导的**T**细胞会持续很多年，在某些情况下甚至几十年，会对原始病毒的重新感染时刻保持警**惕。疫苗所拯救的生命**远多于所有其他医疗干预措施的总和。 传播这一信息，**而不是疾病，接种疫苗！"**。

后记

MHC分子可以控制感染，调节免疫反应，目前正被应用于帮助治疗癌症。这对改善自身免疫和移植排斥的弊端来说是非常有价值的。这就是为什么你**--生活在一个充**满病原体的世界里**--能活着**读这本漫画书的原因。关于如何更好地**生存的更多**细节，请参见参考文献1-6。
