# Supplementary material for: HLA molecules in transplantation, autoimmunity and infection control: A comic book adventure
Source: HLA. 2022 May 15;100(4):301–11. doi: 10.1111/tan.14626 (PMC9545814; doi:10.1111/tan.14626)
Supplement: Supplementary file 1 — Supporting information. [file TAN-100-301-s001.zip › Supplementary files/PP_Dutch_Neefjes Theirlynck.1.pdf]

HLA-moleculen bij transplantatie, auto-immuniteit en infectiebestrijding Een stripboekavontuur.

HLA molecules in transplantation, autoimmunity and infection control.
A comic Book adventure

by Eric Reits and Jacques Neefjes

Vertaald door Jacques Neefjes en Julia Theirlynck. Original text : ***https://doi.org/10.1111/tan.14626***

Department of Cell and Chemical Biology, ONCODE Institute, Leiden University Medical Centre LUMC, The Netherlands

Tekening 1

Zo'n 1900 jaar geleden voerden twee Arabische broers en clinici, Cosmas en Damianus, de eerste beentransplantatie uit. Zij vervingen het gangreneuze been van een koopman door dat van zijn slaaf. Het lot van de slaaf is in de geschiedenis onbekend gebleven, maar dat dit vrijwillig ging, lijkt onwaarschijnlijk.

Tekening 2

Deze "wonderbaarlijke" transplantatie droeg bij aan hun zaligverklaring en zij werden daardoor tot patroonheiligen van de transplantatie benoemd. Zij namen voor lief dat zij werden onthoofd vanwege hun christelijk geloof, hetgeen waarschijnlijk aan de hemelpoort zou worden gecorrigeerd.

Tekening 3

Waarom is transplantatie zo moeilijk? Wat zijn de evolutionaire redenen? Zelfs Darwin moet het zich hebben afgevraagd… Hij wist immers niets af over een unieke klasse van eiwitten die door bijna alle meercellige eukaryoten gemaakt worden.

Tekening 4

Laten we beginnen met het huidige begrip van twee unieke klassen van eiwitten in ons lichaam om dat te begrijpen. Deze eiwitten hebben de hoogste graad van polymorfisme (verschillen tussen ons mensen). Deze zijn uniek omdat bijna alle andere eiwitten nagenoeg identiek zijn bij mensen. Deze polymorfe eiwitten zijn de "transplantatie-antigenen" en worden in het algemeen MHC-klasse I en MHC-klasse II moleculen genoemd. Bij mensen heet het HLA-klasse I en HLA-klasse II.

Tekening 5

De belangrijkste HLA-moleculen voor transplantatie worden HLA-A, HLA-B en HLA-C voor MHC klasse I genoemd en HLA-DR, HLA-DQ en HLA-DP voor MHC klasse II. HLA-A, -B en -C zijn aanwezig in vrijwel al onze cellen (behalve rode bloedcellen), terwijl HLA-DR, HLA-DQ en HLA-DP voornamelijk op immuuncellen zitten.

Tekening 6

HLA-moleculen zijn zo verschillend (polymorf) dat zwangere vrouwen vaak antistoffen aanmaken tegen de verschillende HLA-types van de vader die de foetus weer geërfd heeft. In de tijd dat er nog geen genetische tests beschikbaar waren, kon hiermee de vader bepaald worden. Deze sera van zwangere vrouwen werden echter ook gebruikt bij weefseltransplantatie. Laboratoria wisselden sera van deze zwangere vrouwen uit en de verschillende serumreacties werden in HLA-workshops besproken. Zo werden HLA-A, -B en -C geïdentificeerd en ook verschillende vormen hiervan. Deze werden simpelweg HLA-A1 genummerd, de volgende HLA-A2 enz. Dit gebeurde ook met de HLA-DR-, -DQ- en -DP-moleculen. Uw weefsels kunnen dus (bijvoorbeeld) HLA-A1, -B8, -Cw7, -DR3, -DQ2 en DPw1-eiwitten van uw moeder en HLA-A2, -B27, -Cw1, -DR4, -DQ3, en DPw4-eiwitten van uw vader bevatten.

Tekening 7

Tegenwoordig wordt HLA-typering routinematig uitgevoerd door middel van DNA-analyses. Er zijn aanwijzingen dat vrouwen de verschillen in HLA-types van mannen kunnen onderscheiden aan de hand van geur, hetgeen bijdraagt aan een natuurlijke selectie van genetisch verschillende partners.

Tekening 8

Hoewel HLA-polymorfisme kan helpen om de mensheid verschillend te maken, werpt het een enorme barrière op voor succesvolle orgaantransplantaties, omdat daarbij de HLA-typen van de ontvanger en de donor zo goed mogelijk op elkaar moeten worden afgestemd. Als er geen perfecte match is, worden effectieve immunosuppressiva gebruikt om orgaanafstoting te voorkomen.

Tekening 9

Darwin zou verbaasd hebben gestaan! Want hoe je perfecte partner ruikt, hoe je weefseltransplantatie kan voorkomen en hoe je de biologische vader kan bepalen, dit zullen niet de belangrijkste evolutionaire redenen zijn voor HLA-polymorfisme.

Tekening 10

Er is echter nog een andere factor. Virussen en andere pathogenen zijn in overvloed aanwezig in de natuur. Corona, Influenza, Ebola, Pokken en vele andere virussen gebruiken onze cellen om hun eigen nageslacht te creëren. Zelfs "zelfbeperkende" infecties zouden dodelijk zijn zonder een immuunsysteem. De vraag is simpel: hoe kan het immuunsysteem virussen detecteren die in cellen op de loer liggen, om ze te doden voordat ze ons kunnen doden?

Tekening 11

Om schade door virussen te beperken, heeft het immuunsysteem meerdere wapens ontwikkeld. Een kleine opsomming: macrofagen eten bacteriën en virussen; neutrofielen geven dodelijke stoffen af aan bacteriën; B-cellen maken antilichamen; T-helper cellen helpen B-cellen en andere cellen; T-killer cellen (CTL) doden met virus geïnfecteerde cellen (en zelfs kankercellen).

Tekening 12

Maar hoe weet een T-killer cel wat hij moet doden? Het virus, dat zich in de cel bevindt, is afgeschermd tegen detectie en dus onzichtbaar, of klopt dit niet? Inderdaad, terwijl het virus zich vermenigvuldigt, worden kleine stukjes van zijn eiwitten afgeleverd aan HLA-A-, -B- of -C-moleculen die ze naar het celoppervlak transporteren. De T-killer-cel herkent dit kleine fragment samen met het specifiek HLA-molecuul. De ontdekking van dit fenomeen, dat virussen herkend worden via de eiwitten die ook transplantatie afstoting kunnen veroorzaken, was voldoende belangrijk voor twee Nobelprijzen! Elk ander type MHC klasse I-molecuul presenteert een andere set van peptiden van een virus om het immuunsysteem efficiënt virus-geïnfecteerde cellen te doden.

Tekening 13

Maar hoe wordt een fragment van een viruseiwit eigenlijk gemaakt? Virale eiwitten worden - net als elk ander eiwit in cellen – na een tijdje afgebroken. Eiwitten worden gefragmenteerd door een opmerkelijke nanomachine, het zogenaamde proteasoom, dat feitelijk een afvalbak voor eiwitten is. Andere enzymen in de cel knippen de uiteinden van de fragmenten in kleinere fragmenten (ook wel peptiden genoemd), waarvan sommigen van het cytosol naar de ER worden getransporteerd waar ze HLA-moleculen kunnen binden. Zodra een HLA-molecuul een gebonden peptide heeft, verlaat het de ER en verplaatst zich naar het celoppervlak waar het wacht op detectie door T-killercellen.

Tekening 14

Laten we terugkeren naar HLA-polymorfisme. Zoals iedereen nu weet wat COVID-19 en griep zijn, zijn virussen erg goed in het veranderen en kunnen daardoor ontsnappen aan de antilichamen tegen het eerdere virus (denk aan alfa, delta, omicron....). Om te voorkomen dat een virus aan het afweersysteem kan ontsnappen, presenteert elk van de verschillende MHC-allelen (je hebt er drie van je vader en van je moeder gekregen!) een andere set peptiden. Er worden zoveel verschillende peptiden in één persoon gepresenteerd, dat virussen moeilijk alle sequenties kunnen aanpassen. De verschillen in HLA-types tussen mensen betekenen dat, zelfs als dit gebeurt, het ontwijkende virus dit bedrog bij de volgende persoon niet zal volhouden. Als we allemaal HLA-identiek zouden zijn, dan kan een ontsnappend virus de hele bevolking doden! Nu zal het 'slechts' een paar individuen doden met HLA-moleculen die niet in staat zijn virale peptiden bij het immuunsysteem aan te bieden. Andere mensen met andere HLA moleculen tonen gewoon weer andere peptiden van het zelfde virus aan het afweersysteem. HLA-polymorfisme beschermt dus de populatie; het individu is minder belangrijk. Dit biedt een overtuigende verklaring voor de evolutie van MHC-polymorfisme.

.

Tekening 15

Maar helaas, beste lezer, is dit slecht nieuws voor u indien u nieuwe organen nodig heeft. HLA-polymorfisme bevordert het voortbestaan van een soort populatie, maar niet van een individu met een nierziekte. Transplantatie-afstoting volgt indien het immuunsysteem een donororgaan verwart met een door een virus geïnfecteerd orgaan (de HLA moleculen met een virus peptide zien er hetzelfde uit voor het afweersysteem als andere HLA-allelen) en dan reageert door het orgaan aan te vallen, wat resulteert in afstoting van het transplantaat.

Tekening 16

Een belangrijke algemene les: niets, ook het immuunsysteem niet, is perfect! Laten wij bijvoorbeeld eens nadenken over het feit hoe T-killercellen snel genoeg virus geïnfecteerde cellen kunnen vinden om van enig nut te zijn. Virussen kunnen hun nakomelingen zeer snel produceren; in sommige gevallen in slechts enkele uren. Dit is echter te langzaam om te wachten tot virale eiwitten aan het einde van hun natuurlijke leven worden afgebroken (duurt al snel een uur of tien). Echter net als het immuunsysteem zelf, is de synthese van eiwitten, dus ook virale eiwitten, verre van perfect. Deze imperfecte eiwitten, DRiPs genaamd, worden onmiddellijk afgebroken, waardoor het begin van een virusinfectie wordt gekoppeld aan de presentatie van antigeen en effectieve T-killercellen en het immuno surveillance mogelijk maakt.

Tekening 17

Dus het immuunsysteem wint? Niet zo snel! Sommige slimme virussen, vooral herpesvirussen, zijn geëvolueerd om hun eigen presentatie door MHC moleculen te verstoren. Het afweersysteem is dan blind voor deze virussen. Humaan cytomegalovirus HCMV, dat 60% van de mensheid infecteert, maakt een reeks eiwitten (US2, US3, US6, US11 en US18) die de peptideproductie beperken of de HLA klasse I-functie verstoren.

Tekening 18

Is het dan mogelijk dat sommige HLA-allelen beter zijn in het controleren van virusinfecties dan andere? Inderdaad, sommige HLA-B-allelen beschermen beter tegen HIV, terwijl anderen beter zijn tegen Covid. De verschillende HLA-allelen zijn in de loop van de eeuwen geselecteerd om met verschillende pathogenen om te gaan. HLA-A2 wordt bijvoorbeeld aangetroffen bij 40% van de Europese bevolking. Dit is waarschijnlijk het gevolg van een virus die de mens ergens in het verleden kon doden tenzij deze mensen HLA-A2 aanmaakten om zich tegen dit virus te beschermen. Dit virus kan allang verdwenen zijn, maar HLA-A2 niet.

Tekening 19

Maar er zijn neveneffecten. Neem het HLA-allel HLA-B*27:05. Dit is aanwezig in 8% van de blanke populatie en meer dan 90% van de patiënten met Spondylitis Ankylopoetica (een soort reuma) heeft dit allel, wat waarschijnlijk een auto-immuun T-celreactie in de wervelkolom veroorzaakt. Het immuunsysteem werkt op het snijvlak tussen het bieden van effectieve immuniteit tegen infecties terwijl het de gezonde weefsels met rust moet laten om zo auto-immuunziekten - zoals reuma en suikerziekte - te voorkomen.

Tekening 20

Herkenning van eigen eiwitten door het afweersysteem kan ook gunstig zijn. Kankercellen kennen typisch veel mutaties en andere veranderingen die leiden tot het genereren van peptiden van gemuteerde eiwitten die verschillen van normale peptiden. Immunotherapie tegen kanker maakt gebruik van mechanismen die door het immuunsysteem worden gebruikt bij het herkennen van virale en bacteriële infecties om kankercellen te doden. En het werkt!

Tekening 21

Maar hoe zit het met de HLA-DR, -DQ en -DP MHC klasse II moleculen? Deze moleculen presenteren pathogene peptiden aan T-helpercellen, die vervolgens cytokinen produceren om B-cellen te helpen zich om te bouwen tot antilichaam-producerende fabrieken. T-helpercellen helpen ook om T-killerreacties te optimaliseren. MHC klasse II lijkt qua vorm sterk op MHC klasse I, maar bindt eiwitfragmenten die langer zijn en gemaakt in lysosomen. Dit zijn kleine blaasjes die eiwitten afbreken, die van buiten de cellen zijn verkregen.

Tekening 22

Hoe doen ze dit? MHC klasse II wordt gemaakt in het ER (zoals elk ander eiwit dat naar het buitenmembraan of de lysosomen van de cel moet) waar het een eiwit (invariante keten) bindt dat een peptide nabootst en MHC klasse II naar het lysosoom begeleidt. Hier wordt de invariante keten verwijderd en uitgewisseld voor een peptide dat is gemaakt door lysosomale proteases. Dit proces wordt geoptimaliseerd door nog een ander type MHC-molecuul (HLA-DM), dat lijkt op MHC klasse II en in sommige cellen samenwerkt met HLA-DO, weer een ander klasse II-achtig molecuul. Evolutie is lui, wanneer het een werkende molecuul heeft ontwikkeld, zal het deze eenvoudig kopiëren en wijzigen voor nieuwe functies. Het nettoresultaat van deze gecompliceerde dans is de aflevering van MHC klasse II-moleculen aan het celoppervlak met peptiden die T-helper cellen gaan activeren.

Tekening 23

Dit proces van herkenning van ziekteverwekkers door het immuunsysteem is complex en ook relatief traag. De eerste keer dat jouw lichaam een virus tegenkomt, heeft het immuunsysteem tijd nodig om de antivirale respons op te voeren. Als je pech hebt, kan dit leiden tot ziekte of overlijden door ongecontroleerde virusgroei. Vaccinatie bereidt het immuunsysteem voor op een infectie, waardoor het in sommige gevallen een infectie volledig kan voorkomen en anders sneller en effectiever kan reageren om zodoende de kans op een ernstige infectie aanzienlijk te verkleinen.

Tekening 24

MHC-moleculen zijn cruciale deelnemers voor vaccinatie. Alle vaccins maken gebruik van MHC klasse II-moleculen om T-helpercellen te induceren die nodig zijn voor antilichaamreacties en om de eiwitten te maken waartegen de antilichaamreacties zijn gericht. Adenovirus- en mRNA-vaccins gebruiken ook MHC klasse I-moleculen om T-killercellen te induceren. T-cellen die door vaccins worden geïnduceerd gaan vele jaren mee, soms zelfs tientallen jaren, en zijn op hun hoede voor een nieuwe infectie met het oorspronkelijke virus. Vaccins hebben veel meer levens gered dan alle andere medische interventies samen. Verspreid deze boodschap, niet de ziekte, laat je vaccineren!

Epiloog

Dus MHC-moleculen controleren infecties, reguleren immuunreacties en helpen nu kanker te genezen. Dit is het nadeel van auto-immuniteit en transplantaatafstoting zeker waard. En daarom heb jij – als levend wezen in een wereld vol ziekteverwekkers - het overleefd om dit stripboek te lezen. Zie referenties 1-6 voor meer informatie over hoe je nog beter kunt overleven.
