# Supplementary material for: HLA molecules in transplantation, autoimmunity and infection control: A comic book adventure
Source: HLA. 2022 May 15;100(4):301–11. doi: 10.1111/tan.14626 (PMC9545814; doi:10.1111/tan.14626)
Supplement: Supplementary file 1 — Supporting information. [file TAN-100-301-s001.zip › Supplementary files/PP_French_Stevenin.1.pdf]

Molécules HLA dans les greffes, l'auto-immunité et le contrôle des infections : une aventure en bande dessinée

HLA molecules in transplantation, autoimmunity and infection control.
A comic Book adventure.

by
Eric Reits and Jacques Neefjes

Traduit de l'anglais par Dr Virginie Stévenin. Texte original : ***https://doi.org/10.1111/tan.14626***

Department of Cell and Chemical Biology, ONCODE Institute, Leiden University Medical Centre LUMC, The Netherlands

1 DIAPOSITIVE 1

Il y a environ 1900 ans, deux frères et cliniciens arabes Cosmas et Damianus ont effectué la première transplantation connue, remplaçant la jambe gangrenée d'un marchand par celle de son esclave. Le sort de l'esclave est inconnu dans l'histoire, mais il était peu probable qu'il s'agisse d'un don volontaire.

2 DIAPOSITIVE 2

Cette transplantation « miraculeuse » contribua à leur béatification, les conduisant à devenir les saints patrons de la transplantation. Cela n'a pas fait de mal qu'ils aient été décapités en raison de leur foi chrétienne, qui a vraisemblablement été corrigée lors de leur ascension au ciel.

3 DIAPOSITIVE 3

Pourquoi la transplantation est-elle si difficile, quels sont les facteurs évolutifs ? Même Darwin a dû se poser la question… mais il ne connaissait pas une classe unique de protéines qui sont exprimées par presque tous les eucaryotes multicellulaires.

4 DIAPOSITIVE 4

Commençons par la compréhension actuelle de deux classes uniques de protéines dans notre corps ; ceux qui ont le plus haut degré de polymorphisme (différences entre les individus). Et ceux-ci sont uniques car presque toutes les autres protéines sont presque identiques entre les humains. Ces protéines polymorphes sont les « antigènes de transplantation » et sont généralement appelées molécules du CMH de classe I et du CMH de classe II. Chez l'homme, ils sont appelés HLA de classe I et HLA de classe II.

5 DIAPOSITIVE 5

Les molécules HLA les plus importantes pour la transplantation sont appelées HLA-A, HLA-B et HLA-C pour le CMH de classe I et HLA-DR, HLA-DQ et HLA-DP pour le CMH de classe II. HLA-A, -B et -C sont présents sur pratiquement toutes nos cellules (sauf les globules rouges) tandis que HLA-DR, HLA-DQ et HLA-DP sont principalement sur les cellules immunitaires.

6 DIAPOSITIVE 6

Les molécules HLA sont si polymorphes que les femmes enceintes fabriquent souvent des anticorps contre les différents types HLA du père. Cela pourrait être utilisé pour déterminer le père à une époque antérieure à la disponibilité des tests génétiques. Mais ces sérums de femmes enceintes ont également été utilisés pour la transplantation de tissus. Dans les rencontres scientifiques, les sérums de ces femmes ont été échangés entre les laboratoires et les différentes réponses sériques nommées dans les rencontres HLA. C'est ainsi que HLA-A, -B et -C ont été identifiés ainsi que différentes formes de ceux-ci. Celles-ci étaient simplement numérotées HLA-A1, la suivante HLA-A2, etc. Cela s'est également produit avec les molécules HLA-DR, -DQ et -DP. Ainsi, vos tissus peuvent avoir (par exemple) les protéines HLA-A1, -B8, -Cw7, -DR3, -DQ2 et DPw1 de votre mère et HLA-A2, -B27, -Cw1, -DR4, -DQ3, et les protéines DPw4 de votre père.

7 DIAPOSITIVE 7

Aujourd'hui, le typage HLA est effectué en routine par des analyses ADN. Il existe des preuves que les femmes peuvent détecter des différences dans les types HLA des hommes par l'odorat, et que cela contribue à la sélection de partenaires génétiquement différents.

8 DIAPOSITIVE 8

Bien que le polymorphisme HLA puisse aider à diversifier l'humanité, il s'agit d'un énorme obstacle à la réussite de la transplantation d'organes, qui nécessite de faire correspondre, aussi étroitement que possible, les types HLA du receveur et du donneur. En l'absence d'une compatibilité parfaite, des médicaments immunosuppresseurs efficaces sont utilisés pour prévenir le rejet d'organe.

9 DIAPOSITIVE 9

Darwin serait perplexe. Sûrement, sentir votre compagnon idéal, empêcher une greffe de tissu ou trouver le vrai père ne peuvent pas être les principales raisons évolutives du polymorphisme HLA.

10 DIAPOSITIVE 10

Mais il y a un autre facteur. Les virus et autres agents pathogènes microbiens abondent dans la nature. Corona, Grippe, Ebola, Variole et bien d'autres virus utilisent nos cellules pour créer leurs propres descendances. Même les infections « à guérisons spontanées » seraient mortelles sans système immunitaire. Et la question est simple : comment le système immunitaire peut-il détecter les virus qui se cachent à l'intérieur des cellules pour les tuer avant qu'ils ne puissent nous tuer?

11 DIAPOSITIVE 11

Pour limiter les dommages causés par les virus, le système immunitaire a développé plusieurs armes. Les macrophages mangent des bactéries et des virus, les neutrophiles libèrent des substances tueuses pour les bactéries, les cellules B fabriquent des anticorps, les cellules T auxiliaires aident les cellules B et d'autres cellules, les cellules T tueuses tuent les cellules infectées par le virus (et même les cellules cancéreuses).

12 DIAPOSITIVE 12

Mais comment une cellule T-tueuse sait-elle qui tuer ? Le virus, étant à l'intérieur de la cellule, est à l'abri de la détection, n'est-ce pas ? En effet, au fur et à mesure que le virus se réplique, de minuscules morceaux de ses protéines sont délivrés aux molécules HLA-A, -B ou -C qui les transportent à la surface des cellules. La cellule T-tueuse reconnaît ce petit fragment dans le contexte d'UNE molécule HLA spécifique. La découverte de ce phénomène, appelé restriction HLA, était suffisamment importante pour remporter deux prix Nobel. Chaque type différent de molécule du CMH de classe I présente un répertoire différent de peptides pour donner au système immunitaire de nombreuses cibles à viser et à tuer les cellules qui les produisent.

13 DIAPOSITIVE 13

Mais comment un fragment de virus est-il généré en premier lieu ? Les protéines virales, comme toute autre protéine à l'intérieur des cellules, sont dégradées. Les protéines sont fragmentées par une nano-machine remarquable appelée le protéasome, qui est essentiellement un centre de recyclage pour les protéines. D'autres enzymes cellulaires coupent les extrémités des fragments en peptides plus petits, dont certains sont transportés du cytosol vers le RE où ils peuvent se lier aux molécules HLA. Une fois qu'une molécule HLA a un peptide lié, elle quitte le RE pour la surface cellulaire où elle attend la détection par les cellules T-tueuses.

14 DIAPOSITIVE 14

Revenons au polymorphisme HLA. Comme tout le monde le sait depuis le COVID-19 et la grippe, les virus sont très doués pour se transformer et échapper à la réponse des anticorps (pensez alpha, delta, omicron….). Pour minimiser cette possibilité pour les lymphocytes T, chacun des différents allèles du CMH (variétés de gènes) présente un ensemble différent de peptides. Il y a tellement de peptides présentés chez une seule personne que l'évasion virale devient difficile. Les différences de types HLA entre les personnes signifient que même si cela se produit, le virus échappé ne poursuivra pas sa tromperie chez la personne suivante. Si nous aurions tous été HLA identiques, un virus en fuite tuerait toute la population, maintenant il ne tuera « que » quelques individus avec des molécules HLA incapables de présenter des peptides viraux au système immunitaire. Le polymorphisme HLA protège donc la population, l'individu est moins important. Cela fournit une explication convaincante de l'évolution du polymorphisme du CMH.

15 DIAPOSITIVE 15

Mais hélas, mauvaise nouvelle pour vous, cher lecteur, s'il vous arrive d'avoir besoin d'un nouvel organe ou de deux. Le polymorphisme HLA favorise la survie d'une population d'espèces, et non d'un individu atteint de maladie rénale. Le rejet de greffe est la conséquence du fait que le système immunitaire confond un organe donneur avec un organe infecté par un virus et réagit en conséquence en attaquant l'organe entraînant le rejet de greffe.

16 DIAPOSITIVE 16

Une leçon générale importante : rien, y compris le système immunitaire n'est parfait ! En parlant de cela, réfléchissons à la façon dont les cellules T tueuses peuvent trouver des cellules infectées par le virus assez rapidement pour être utiles. Les virus peuvent produire leur progéniture très rapidement, dans certains cas en quelques heures seulement. C'est trop lent pour attendre que les protéines virales soient dégradées à la fin de leur vie naturelle. Mais tout comme le système immunitaire lui-même, la synthèse des protéines, dont les protéines virales, est loin d'être parfaite. Ces protéines imparfaites, appelées DRiP, sont dégradées immédiatement, couplant le début de l'infection virale à la présentation de l'antigène et permettant une immuno-surveillance efficace des cellules T-tueuse.

17 DIAPOSITIVE 17

Échec et mat, système immunitaire ? Pas si vite ! Certains virus intelligents, en particulier les virus de l'herpès, ont évolué pour interférer avec la présentation d'antigène. Le cytomégalovirus humain HCMV, qui infecte 60 % de l'humanité, fabrique une suite de protéines (US2, US3, US6, US11 et US18) qui limitent la production de peptides ou interfèrent avec la fonction de HLA de classe I.

18 DIAPOSITIVE 18

Est-il alors possible que certains allèles HLA soient plus efficaces que d'autres pour gérer les infections virales ? En effet, certains allèles HLA-B protègent mieux contre le VIH, d'autres sont meilleurs pour le Covid. Les différents allèles HLA ont été sélectionnés au cours des ères pour faire face à différents agents pathogènes. Par exemple, HLA-A2 se trouve dans 40% de la population européenne, la prévalence la plus élevée de tous les allèles HLA dans un groupe donné. Cela résulte probablement de la capacité de HLA-A2 à protéger contre un agent pathogène à un moment donné dans le temps, qui pourrait bien ne plus être une cause majeure de maladie humaine.

19 DIAPOSITIVE 19

Mais il y a des conséquences collatérales. Prenez l'allèle HLA HLA-B*27:05. Présent chez 8 % de la population caucasienne, plus de 90 % des patients atteints de spondylarthrite ankylosante possèdent cet allèle, qui déclenche probablement une réaction auto-immune des lymphocytes T dans la colonne vertébrale. Le système immunitaire fonctionne sur le fil du rasoir entre fournir une immunité efficace et éviter d’endommager les tissus par des tirs amis.

20 DIAPOSITIVE 20

L'auto-immunité des lymphocytes T peut également être bénéfique. Les cellules cancéreuses présentent généralement de nombreuses mutations et autres altérations qui conduisent à la génération de peptides différents des peptides cellulaires normaux. L'immunothérapie anticancéreuse exploite les mécanismes utilisés par le système immunitaire pour reconnaître les infections virales et bactériennes afin de tuer les cellules cancéreuses.

21 DIAPOSITIVE 21

Mais qu'en est-il des molécules HLA-DR, -DQ et -DP du CMH de classe II ? Ces molécules présentent des peptides pathogènes aux cellules T auxiliaires, qui produisent ensuite des cytokines pour aider les cellules B à se différencier en usines de production d'anticorps. Les cellules T auxiliaires aident également à optimiser les réponses des cellules T-tueuses.

Le CMH de classe II a une forme très similaire au CMH de classe I mais présente des fragments de protéines plus longs et fabriqués dans des lysosomes, qui sont de petits organites qui dégradent les protéines acquises à l'extérieur des cellules.

22 DIAPOSITIVE 22

Comment font-ils ça ? Le CMH de classe II est fabriqué dans le RE (comme toute autre protéine qui doit aller à la membrane externe ou aux lysosomes de la cellule) où il lie une protéine (chaîne invariante) qui imite un peptide et chaperonne le CMH de classe II au lysosome. Ici, la chaîne invariante est supprimée et échangée contre un peptide créé par les enzymes lysosomales. Ce processus est optimisé par un autre type de molécule du CMH (HLA-DM, qui ressemble au CMH de classe II et, dans certaines cellules, agit de concert avec HLA-DO, une autre molécule de type classe II. L'évolution est paresseuse, lorsqu'elle a développé un module de travail, il le copiera et le modifiera simplement pour de nouvelles fonctions). Le résultat clair de cette complexe chorégraphie est la livraison de molécules du CMH de classe II à la surface cellulaire avec des peptides qui permettent l'activation des cellules T auxiliaires.

23 DIAPOSITIVE 23

Ce processus de reconnaissance des pathogènes par le système immunitaire est complexe… mais il est aussi relativement lent. La première fois que vous rencontrez un virus, le système immunitaire met du temps à accélérer la réponse antivirale. Si vous êtes malchanceux, cela peut entraîner une maladie ou la mort par réplication virale incontrôlée. La vaccination prépare le système immunitaire à une infection, lui permettant dans certains cas de prévenir complètement l'infection, et sinon de réagir plus rapidement et plus efficacement et de réduire considérablement les risques d'infection grave.

24 DIAPOSITIVE 24

Les molécules du CMH sont des participants essentiels à la vaccination. Tous les vaccins utilisent des molécules du CMH de classe II pour induire les cellules T auxiliaires nécessaires aux réponses anticorps et fabriquer les protéines contre lesquelles les réponses anticorps sont dirigées. Les vaccins à adénovirus et à ARNm utilisent également des molécules du CMH de classe I pour stimuler des cellules T-tueuses. Les cellules T stimulées par les vaccins durent de nombreuses années, voire des décennies dans certains cas, à l'affût d'une nouvelle infection par le virus d'origine. Les vaccins ont sauvé bien plus de vies que toutes les autres interventions médicales combinées. Faites passer ce message, pas la maladie, faites-vous vacciner !

25 ÉPILOGUE

Ainsi, les molécules du CMH contrôlent les infections, régulent les réponses immunitaires et aident maintenant à guérir le cancer. Cela vaut bien l'inconvénient de l'auto-immunité et du rejet de greffe. Et c'est pourquoi vous, qui vivez dans un monde rempli d'agents pathogènes, avez survécu pour lire cette bande dessinée. Pour plus de détails sur la façon de survivre encore mieux, veuillez consulter les références 1-6.
