# Supplementary material for: HLA molecules in transplantation, autoimmunity and infection control: A comic book adventure
Source: HLA. 2022 May 15;100(4):301–11. doi: 10.1111/tan.14626 (PMC9545814; doi:10.1111/tan.14626)
Supplement: Supplementary file 1 — Supporting information. [file TAN-100-301-s001.zip › Supplementary files/PP_German_Fleischhauer und Arrieta-Bolaños_1.pdf]

HLA Moleküle in der Transplantation, Autommunität und Infektion: eine Reise durch ein Karikaturbuch

HLA molecules in transplantation, autoimmunity and infection control.
A comic Book adventure

by

Eric Reits and Jacques Neefjes

Aus dem Englischen übersetzt von Prof. Katharina Fleischhauer und Dr. Thuja Arrieta-Bolaños, Universitätsklinikum Essen, Deutschland. Texte original : ***https://doi.org/10.1111/tan.14626***

Department of Cell and Chemical Biology, ONCODE Institute, Leiden University Medical Centre LUMC, The Netherlands

Folie 1

Vor etwa 1900 Jahren führten zwei arabische Brüder und Kliniker, Cosmas und Damian, die erste bekannte Transplantation durch, indem sie das abgestorbene Bein eines Kaufmanns mit dem seines Sklaven ersetzten. Das Schicksal des Sklaven in der Geschichte bleibt ungewiss, doch es ist unwahrscheinlich, dass es sich um eine freiwillige Spende gehandelt hat.

Folie 2

Diese « wundersame » Transplantation trug zur Heiligsprechung von Cosmas und Damian bei, wodurch sie zu den Schutzheiligen der Transplantation wurden. Hierbei war es nicht von Nachteil, dass sie später aufgrund ihres christlichen Glaubens enthauptet wurden, was vermutlich nach ihrem Aufstieg ins Himmelreich wieder gut gemacht wurde.

Folie 3

Warum ist eine Transplantation so schwierig, was sind die evolutionären Faktoren hierbei? Selbst Darwin muss sich diese Frage gestellt haben – aber er kannte eine einzigartige Klasse von Proteinen nicht, die auf der Oberfläche von fast allen vielzelligen Eukaryoten exprimiert werden.

Folie 4

Lasst uns damit beginnen, was über zwei einzigartige Klassen von Proteinen in unserem Körper bekannt ist; nämlich denen, die den höchsten Grad an Polymorphismus (d.h. Unterschiede zwischen verschiedenen Individuen) aufweisen. Und diese sind einzigartig, weil fast alle anderen Proteine in allen menschlichen Individuen annähernd identisch sind. Diese polymorphen Proteine sind die « Transplantationsantigene », welche im Allgemeinen als MHC Klasse I und MHC Klasse II Moleküle bezeichnet werden. Im Menschen heißen sie HLA Klasse I und HLA Klasse II.

Folie 5

Die für die Transplantation wichtigsten HLA Moleküle werden HLA-A, HLA-B und HLA-C für MHC Klasse I und HLA-DR, HLA-DQ, HLA-DP für MHC Klasse II genannt. HLA-A, HLA-B und HLA-C befinden sich auf buchstäblich all unseren Zellen (außer den roten Blutkörperchen), während HLA-DR, HLA-DQ und HLA-DP sich vor allem auf Immunzellen befinden.

Folie 6

Der Polymorphismus von HLA Molekülen ist so extrem, dass schwangere Frauen oft Antikörper gegen die verschiedenen HLA Typen des Vaters ausbilden. Diese Tatsache wurde früher für Vaterschaftsbestimmungen genutzt, in Zeiten bevor es genetische Testungen hierfür gab. Aber die Blutseren (welche die Antikörper enthalten) schwangerer Frauen waren auch für die Gewebetransplantation sehr hilfreich. Sie wurden nämlich im Rahmen von internationalen Kooperationen, den sogenannten «HLA workshops », zwischen verschiedenen Laboren ausgetauscht, und die Spezifität der jeweiligen Antikörper wurde bestimmt und mit Namen versehen. Dies führte zur Entdeckung von HLA-A, -B und –C und deren Untergruppen, welche einfach numerisch benannt wurden als HLA-A1, HLA-A2 usw. Genauso verfuhr man mit HLA-DR, -DQ und –DP Molekülen. Deshalb können menschliche Gewebe (z.B.) HLA-A1, -B8, -Cw7, -DR3, -DQ2 und DPw1 Proteine mütterlicherseits sowie HLA-A2, -B27, -Cw1, -DR4, -DQ3, und DPw4 Proteine väterlicherseits enthalten.

Folie 7

Heutzutage wird die HLA Typisierung routinemäßig durch DNA Untersuchungen durchgeführt. Es gibt Hinweise, dass Frauen HLA Unterschiede in Männern am Geruch erkennen können, und dass dies die Selektion von genetisch diversen Partnern fördert.

Folie 8

Der Polymorphismus der HLA Moleküle ist zwar äußerst hilfreich für die menschliche Diversität, er stellt aber ein enormes Hindernis für die erfolgreiche Organtransplantation dar, welche die bestmögliche Kompatibilität der HLA Typen von Empfänger und Spender erfordert. Wenn keine perfekte Kompatibilität vorhanden ist, werden wirksame immunsupprimierende Medikamente eingesetzt, um die Abstoßung des transplantierten Organs zu verhindern.

Folie 9

Darwin würde sich wundern. Es kann doch nicht sein, dass der HLA Polymorphismus evolutionär darin begründet liegt, das Erschnüffeln des besten Partners, die Verhinderung erfolgreicher Gewebetransplantationen oder die Vaterschaftsbestimmung zu ermöglichen.

Folie10

Es gibt jedoch einen weiteren Faktor. Viren und andere mikrobielle Pathogene gibt es in der Natur in großer Vielzahl. Corona, die Grippe, Ebola, Pocken und viele andere Viren nutzen unsere Zellen für ihre eigene Vermehrung. Und sogar «selbstlimitierende » Infektionen wären ohne ein Immunsystem tödlich. Die Frage ist also einfach: wie kann das Immunsystem Viren erkennen welche in der Zelle lauern, um diese zu töten bevor sie uns töten?

Folie 11

Um virusbedingte Schädigungen zu begrenzen, hat das Immunsystem eine Vielfalt von Waffen entwickelt. Makrophagen fressen Bakterien und Viren, neutrophile Granulozyten setzen Substanzen frei die Bakterien abtöten können, B Zellen produzieren Antikörper, T-Helferzellen kommen den B Zellen und anderen Immunzellen zu Hilfe, T-Killerzellen töten virus-infizierte Zellen (und sogar Krebszellen) ab.

Folie 12

Wie findet aber eine T-Killerzelle heraus, wen sie töten soll? Das Virus kann ja gar nicht entdeckt werden, da es in der Zelle sitzt, oder doch? Während sich das Virus vermehrt, werden in der Tat kleine Teile seines Proteins an die HLA-A, -B, oder –C Molekülen geliefert, welche diese zur Zelloberfläche befördern. Die T-Killerzelle erkennt dieses kleine Proteinfragment (auch Peptid genannt) zusammen mit EINEM einzigen spezifischen HLA Molekül. Die Entdeckung dieses Phänomens, das man heute HLA Restriktion nennt, war bedeutend genug um mit zwei Nobelpreisen ausgezeichnet zu werden. Jeder unterschiedliche Typ von MHC Klasse I Molekülen präsentiert ein unterschiedliches Repertoire an Peptiden, um dem Immunsystem eine große Menge an Zielscheiben zur Verfügung zu stellen für die Erkennung und Abtötung der Zellen, welche diese Peptide produzieren.

Folie 13

Wie wird ein Proteinfragment des Virus denn überhaupt generiert? Genau wie alle anderen Proteine im Zellinneren, werden auch virale Proteine zersetzt. Diese Zersetzung erfolgt in einer bemerkenswerten Nano-Maschine, die sich Proteasom nennt, hierbei handelt es sich um eine Art Recyclinghof für Proteine. Andere zelluläre Enzyme trimmen die Enden der Proteinfragmente in noch kleinere Peptide, von denen einige aus dem Zytoplasma ins ER transportiert werden, wo sie wiederum an die HLA Moleküle binden können. Sobald ein HLA Molekül ein Peptid gebunden hat, verlässt es das ER um zur Zelloberfläche zu gelangen, wo es darauf wartet von T-Killerzellen entdeckt zu werden.

Folie 14

Kommen wir auf den HLA Polymorphismus zurück. Wie uns von COVID-19 und der Grippe bereits wohlbekannt ist, sind Viren sehr geschickt darin, sich zu verändern um der Antikörperantwort zu entkommen (man denke an alpha, delta, omicron…). Um diese Möglichkeit für die T Zellantwort möglichst gering zu machen, präsentiert jedes der verschiedenen MHC Allele (also verschiedene MHC Gene) einen unterschiedlichen Satz an Peptiden. Es werden so viele Peptide in jeder Person präsentiert, dass die Evasion des Virus sehr erschwert wird. Durch die interindividuellen Unterschiede der HLA Typen wird sichergestellt, dass selbst wenn eine virale Evasion stattfindet, diese in der nächsten infizierten Person nicht erfolgreich sein wird. Wenn wir alle das gleiche HLA hätten, würde eine virale Evasion die gesamte Bevölkerung töten, während sie so «nur » ein paar Einzelne töten wird, deren HLA Moleküle nicht in der Lage sind, dem Immunsystem die viralen Peptide zu präsentieren. Der HLA Polymorphismus schützt also die Bevölkerung, der Einzelne ist nicht so wichtig. Dieses Konzept liefert eine überzeugende Erklärung für die Evolution des MHC Polymorphismus.

Folie 15

Leider sind das jedoch schlechte Nachrichten für diejenigen Leser, die zufällig ein oder zwei neue Organe benötigen. Der HLA Polymorphismus stärkt das Überleben der Artenvielfalt, nicht aber das eines Einzelnen mit einer Nierenerkrankung. Die Transplantatabstoßung ist die Folge davon, dass das Immunsystem das Spenderorgan mit einer virus-infizierten Zelle verwechselt und demzufolge das Organ angreift, was zur Abstoßung führt.

Folie 16

Hier ist eine wichtige allgemeine Lehre: nichts ist perfekt, auch das Immunsystem nicht! Apropos, lasst uns überlegen wie es T-Killerzellen gelingt, virus-infizierte Zellen schnell genug zu identifizieren um nützlich zu sein. Viren können sich sehr rasch vermehren, manchmal in nur wenigen Stunden. Daher würde es zu viel Zeit kosten darauf zu warten, dass virale Proteine am Ende ihres natürlichen Lebens zersetzt werden. Doch genau wie das Immunsystem selbst, ist auch die Proteinsynthese und mit ihr die Synthese viraler Proteine, alles andere als perfekt. Imperfekte Proteine, DRiPs genannt, werden augenblicklich zersetzt, wodurch sie den Beginn einer Virusinfektion mit der Antigenpräsentation koppeln und eine wirksame Immunkontrolle durch T-Killerzellen ermöglichen.

Folie 17

Also Schachmatt, liebes Immunsystem? Halt, nicht so voreilig! Manche schlauen Viren, insbesondere Herpesviren, haben Anpassungsmechanismen entwickelt, die mit der Antigenpräsentation interferieren. HCMV, das humane Zytomegalovirus, infiziert 60% aller Menschen und exprimiert eine Vielzahl von Proteinen (wie z.B. US2, US3, US6, US11, und US18), welche die Produktion von Peptiden einschränken oder mit der Funktion von HLA Klasse I Molekülen interferieren.

Folie 18

Heißt das also, dass manche HLA Allele besser dazu geeignet sind, mit bestimmten Infektionen fertig zu werden, als andere? Tatsächlich schützen manche HLA-B Allele vor HIV, andere sind besser gegen COVID. Die verschiedenen HLA Allele sind im Lauf von Äonen durch die Auseinandersetzung mit verschiedenen Pathogenen selektioniert worden. Beispielsweise findet sich HLA-A2 in 40% der Europäischen Bevölkerung, das ist die höchste Prävalenz eines HLA Alleles in einer bestimmten Gruppe. Dies spiegelt vermutlich die Tatsache wider, dass HLA-A2 irgendwann einmal den wirksamsten Schutz gegen ein Pathogen geboten hat, das möglicherweise heute gar kein wesentlicher Krankheitserreger mehr ist.

Folie 19

Es gibt jedoch Nebeneffekte. Man nehme z.B. das Allel HLA-B*27:05. Es kommt in 8% der kaukasischen Bevölkerung vor, aber 90% aller Patienten mit ankylosierender Spondylitis haben dieses Allel, welches vermutlich eine T-Zell-vermittelte Autoimmunreaktion in der Wirbelsäule verursacht. Das Immunsystem agiert auf einem schmalen Grat zwischen der Vermittlung von wirksamem Schutz gegen Pathogene und dem Vermeiden von Gewebeschäden durch Beschuss aus den eigenen Reihen.

Folie 20

Allerdings kann die T-Zell-vermittelte Autoimmunität auch ihr Gutes haben. Krebszellen zeichnen sich häufig durch Mutationen oder andere Veränderungen aus, die zur Produktion von Peptiden führen, welche sich von normalen zellulären Peptiden unterscheiden. In der Tumor-Immuntherapie werden dieselben Mechanismen genutzt die das Immunsystem dazu bringen, virale oder bakterielle Infektionen zu erkennen, um Krebszellen abzutöten.

Folie 21

Und wie steht es um die HLA-DR, -DQ und –DP MHC Klasse II Moleküle? Diese präsentieren pathogene Peptide an T-Helferzellen, welche wiederum Zytokine produzieren um B Zellen zu helfen, sich in Antikörper-produzierende Fabriken umzuwandeln. T-Heferzellen dienen auch der Optimierung von T-Killerzell Antworten. MHC Klasse II sind in ihrer Struktur den MHC Klasse I Molekülen sehr ähnlich, aber sie präsentieren längere Proteinfragmente, welche in Lysosomen generiert werden, das sind kleine Organellen die Proteine zersetzen, welche von außen in die Zelle aufgenommen wurden.

Folie 22

Wie machen sie das? MHC Klasse II Moleküle werden im ER hergestellt (ebenso wie alle anderen Proteine, die für die Expression auf der äußeren Zellmembran oder in den Lysosomen bestimmt sind). Dort binden sie ein anderes Protein, die «invariant chain », welche so aussieht wie ein Peptid und das MHC Klasse II Molekül in die Lysosomen leitet. Dort wird die «invariant chain » entfernt und gegen ein durch lysosomale Enzyme generiertes Peptid ausgetauscht. Dieser Prozess wird verfeinert durch einen weiteren Typ von MHC Molekülen (HLA-DM, welches so ähnlich aussieht wie die anderen MHC Klasse II Moleküle und das in manchen Zellen mit HLA-DO zusammenwirkt, ein anderes Klasse II-ähnliches Molekül. Die Evolution ist faul, wenn einmal ein Arbeitsmodell entwickelt wurde, wird dieses einfach kopiert und an andere Funktionen angepasst). Das Endresultat dieses komplexen Tanzes ist die Lieferung von MHC Klasse II Molekülen an die Zelloberfläche, welche mit Peptiden geladen sind, die die Aktivierung von T-Helferzellen ermöglichen.

Folie 23

Dieser Prozess der Pathogenerkennung durch das Immunsystem ist komplex… aber er ist auch relativ langsam. Wenn man das erste Mal mit einem Virus in Kontakt kommt, braucht das Immunsystem etwas Zeit, um die anti-virale Antwort hochzufahren. Wenn man Pech hat, kann dies zur Erkrankung oder sogar zum Tod durch unkontrollierte Virusvermehrung führen. Impfungen bereiten das Immunsystem auf eine Infektion vor, so dass diese im besten Fall ganz verhindert wird, zumindest aber eine raschere und wirksamere Antwort hervorgerufen wird, welche das Risiko einer schweren Infektion deutlich reduziert.

Folie 24

MHC Moleküle haben eine kritische Bedeutung bei Impfungen. Alle Impfstoffe bedienen sich der MHC Klasse II Moleküle um T-Helferzellen zu aktivieren, die für die Antikörperbildung gebraucht werden, und um die Proteine zu produzieren, gegen welche die Antikörperantworten gerichtet sind. Adenovirus und mRNA Impfstoffe bedienen sich auch der MHC Klasse II Moleküle, um T-Killerzellen zu aktivieren. T Zellen die im Rahmen einer Impfung aktiviert werden, verweilen für viele Jahre in unserem Körper, manchmal gar für Jahrzehnte, in Alarmbereitschaft für eine neue Infektion mit dem ursprünglichen Virus. Impfungen haben weitaus mehr Leben gerettet als alle anderen medizinischen Maßnahmen zusammengenommen. Verbreiten Sie also diese Information und nicht die Krankheit, lassen Sie sich impfen!

25 EPILOG

MHC Moleküle sind also in der Lage, Infektionen unter Kontrolle zu bringen und Immunantworten zu regulieren, und sie werden auf Grund dieser Eigenschaften heute dazu eingesetzt, Krebs zu heilen. Das wiegt ihre Nachteile der Autoimmunität und Transplantatabstoßung bei Weitem auf. Und sie sind auch der Grund warum Sie lieber Leser – obwohl Sie in einer Welt voller Pathogene leben – überlebt haben, so dass Sie dieses Karikaturbuch lesen konnten. Weitere Einzelheiten dazu, wie man noch besser überleben kann, finden Sie in den Literaturhinweisen 1-6.
