# Supplementary material for: HLA molecules in transplantation, autoimmunity and infection control: A comic book adventure
Source: HLA. 2022 May 15;100(4):301–11. doi: 10.1111/tan.14626 (PMC9545814; doi:10.1111/tan.14626)
Supplement: Supplementary file 1 — Supporting information. [file TAN-100-301-s001.zip › Supplementary files/PP_Greek_Thomaidou.1.pdf]

Μόρια HLA στη μεταμόσχευση, την αυτοανοσία και τον έλεγχο των λοιμώξεων: Μια περιπέτεια κόμικς

HLA molecules in transplantation, autoimmunity and infection control.
A comic Book adventure

by Eric Reits and Jacques Neefjes

Translated by Sofia Thomaidou. Original text : ***https://doi.org/10.1111/tan.14626***

Department of Cell and Chemical Biology, ONCODE Institute, Leiden University Medical Centre LUMC, The Netherlands

ΔΙΑΦΑΝΕΙΑ 1

Πριν από 1900 περίπου χρόνια, δύο Άραβες αδελφοί και γιατροί, ο Κοσμάς και ο Δαμιανός, πραγματοποίησαν την πρώτη γνωστή μεταμόσχευση, αντικαθιστώντας το γαγγραινώδες πόδι ενός εμπόρου με εκείνο του σκλάβου του. Η τύχη του σκλάβου είναι άγνωστη στην ιστορία, αλλά είναι απίθανο να επρόκειτο για εθελοντική δωρεά.

ΔΙΑΦΑΝΕΙΑ 2

Αυτή η "θαυματουργή" μεταμόσχευση έγινε η αιτία για την αγιοποίησή τους, με αποτέλεσμα να γίνουν οι προστάτες άγιοι των μεταμοσχεύσεων. Σε αυτό μάλλον συνέβαλε και το γεγονός ότι αποκεφαλίστηκαν λόγω της χριστιανικής τους πίστης, το οποίο πιθανώς αποκαταστάθηκε κατά την ανάληψή τους στον ουρανό.

ΔΙΑΦΑΝΕΙΑ 3

Γιατί η μεταμόσχευση είναι τόσο δύσκολη, ποιοι είναι οι εξελικτικοί παράγοντες; Ακόμη και ο Δαρβίνος πρέπει να αναρωτήθηκε... αλλά δεν γνώριζε για μια μοναδική κατηγορία πρωτεϊνών που εκφράζονται σχεδόν από όλους τους πολυκύτταρους ευκαρυωτικούς οργανισμούς.

ΔΙΑΦΑΝΕΙΑ 4

Ας ξεκινήσουμε με την τρέχουσα κατανόηση δύο μοναδικών κατηγοριών πρωτεϊνών στο σώμα μας, αυτών που έχουν τον υψηλότερο βαθμό πολυμορφισμού (διαφορές μεταξύ των ατόμων). Και αυτές είναι μοναδικές καθώς σχεδόν όλες οι άλλες πρωτεΐνες είναι σε μεγάλο βαθμό πανομοιότυπες μεταξύ των ανθρώπων. Αυτές οι πολυμορφικές πρωτεΐνες είναι τα "αντιγόνα μεταμόσχευσης" και γενικά ονομάζονται μόρια MHC τάξης I και MHC τάξης II. Στους ανθρώπους ονομάζονται HLA τάξης I και HLA τάξης II.

ΔΙΑΦΑΝΕΙΑ 5

Τα πιο σημαντικά μόρια HLA για τη μεταμόσχευση ονομάζονται HLA-A, HLA-B και HLA-C για την τάξη I MHC και HLA-DR, HLA-DQ και HLA-DP για την τάξη II MHC. Τα HLA-A, -B και -C είναι πρακτικά παρόντα σε όλα μας τα κύτταρα (εκτός από τα ερυθρά αιμοσφαίρια), ενώ τα HLA-DR, HLA-DQ και HLA-DP βρίσκονται κυρίως σε κύτταρα του ανοσοποιητικού συστήματος.

ΔΙΑΦΑΝΕΙΑ 6

Τα μόρια HLA είναι τόσο πολυμορφικά που οι έγκυες γυναίκες συχνά παράγουν αντισώματα έναντι των διαφορετικών τύπων HLA του πατέρα. Αυτό θα μπορούσε να χρησιμοποιηθεί για τον προσδιορισμό του πατέρα σε εποχές που δεν υπήρχαν διαθέσιμες γενετικές εξετάσεις. Αυτοί οι οροί των εγκύων γυναικών χρησιμοποιούνταν επίσης και για τη μεταμόσχευση ιστών. Σε επιστημονικές συναντήσεις, οι οροί αυτών των γυναικών ανταλλάσσονταν μεταξύ των εργαστηρίων και οι διαφορετικές ορολογικές αντιδράσεις προσδιορίζονταν στα λεγόμενα HLA σεμινάρια . Έτσι αναγνωρίστηκαν τα HLA-A, -B και -C, καθώς και οι διάφορες μορφές αυτών. Αυτές αριθμήθηκαν απλώς ως HLA-A1, η επόμενη HLA-A2 κ.λπ. Αυτό συνέβη και με τα μόρια HLA-DR, -DQ και -DP. Έτσι, οι ιστοί σας μπορεί να έχουν (για παράδειγμα) τις πρωτεΐνες HLA-A1, -B8, -Cw7, -DR3, -DQ2 και DPw1 από τη μητέρα σας και τις πρωτεΐνες HLA-A2, -B27, -Cw1, -DR4, -DQ3 και DPw4 από τον πατέρα σας.

ΔΙΑΦΑΝΕΙΑ 7

Σήμερα, η τυποποίηση HLA γίνεται συνήθως με αναλύσεις DNA. Υπάρχουν κάποιες ενδείξεις ότι οι γυναίκες μπορούν να ανιχνεύσουν διαφορές στους τύπους HLA των ανδρών με τη όσφρηση και ότι αυτό συμβάλλει στην επιλογή γενετικά διαφορετικών συντρόφων.

ΔΙΑΦΑΝΕΙΑ 8

Ενώ ο πολυμορφισμός HLA συμβάλλει στη διαφοροποίηση της ανθρωπότητας, αποτελεί τεράστιο εμπόδιο για την επιτυχή μεταμόσχευση οργάνων, η οποία απαιτεί την όσο το δυνατόν καλύτερη ταύτιση των τύπων HLA του λήπτη και του δότη. Ελλείψει τέλειας συμβατότητας, χρησιμοποιούνται δραστικά ανοσοκατασταλτικά φάρμακα για την πρόληψη της απόρριψης των οργάνων.

ΔΙΑΦΑΝΕΙΑ 9

Ο Δαρβίνος θα προβληματιζόταν. Σίγουρα, το να μυρίζεις το τέλειο ταίρι σου, να αποτρέπεις τη μεταμόσχευση ιστών ή να βρίσκεις τον πραγματικό πατέρα δεν μπορεί να είναι οι κύριοι εξελικτικοί λόγοι για τον πολυμορφισμό HLA.

ΔΙΑΦΑΝΕΙΑ 10

Υπάρχει όμως και ένας άλλος παράγοντας. Οι ιοί και άλλα μικροβιακά παθογόνα αφθονούν στη φύση. Ο κορωναίος, η γρίπη, ο 'εμπολα, η ευλογιά και πολλοί άλλοι ιοί χρησιμοποιούν τα κύτταρά μας για να δημιουργήσουν τις δικές τους οικογένειες. Ακόμη και οι "αυτοπεριοριζόμενες" λοιμώξεις θα ήταν θανατηφόρες χωρίς ανοσοποιητικό σύστημα. Και το ερώτημα είναι απλό: πώς μπορεί το ανοσοποιητικό σύστημα να ανιχνεύσει τους ιούς που κρύβονται μέσα στα κύτταρα για να τους σκοτώσει προτού μπορέσουν να μας σκοτώσουν;

ΔΙΑΦΑΝΕΙΑ 11

Για να περιορίσει τη βλάβη από τους ιούς, το ανοσοποιητικό σύστημα ανέπτυξε πολλά όπλα. Τα μακροφάγα τρώνε τα βακτήρια και τους ιούς, τα ουδετερόφιλα απελευθερώνουν ουσίες που σκοτώνουν τα βακτήρια, τα Β κύτταρα παράγουν αντισώματα, τα Τ-βοηθητικά βοηθούν τα Β και άλλα κύτταρα, τα Τ-κυτταροτοξικά (φονικά) σκοτώνουν τα μολυσμένα από ιούς κύτταρα (ακόμη και τα καρκινικά).

ΔΙΑΦΑΝΕΙΑ 12

Πώς όμως ένα Τ-φονικό κύτταρο ξέρει ποιον να σκοτώσει; Ο ιός, όντας μέσα στο κύτταρο, προστατεύεται από την ανίχνευση, ή μήπως όχι; Πράγματι, καθώς ο ιός πολλαπλασιάζεται, μικροσκοπικά κομμάτια των πρωτεϊνών του καταλήγουν σε μόρια HLA-A, -B ή -C, τα οποία τα μεταφέρουν στην επιφάνεια του κυττάρου. Το Τ-φονικό κύτταρο αναγνωρίζει αυτό το μικρό τμήμα στο πλαίσιο ΕΝΟΣ συγκεκριμένου μορίου HLA. Η ανακάλυψη αυτού του φαινομένου, που ονομάζεται HLA-περιορισμός, ήταν αρκετά σημαντική ώστε να αποσπάσει δύο βραβεία Νόμπελ. Κάθε διαφορετικός τύπος μορίου MHC τάξης I παρουσιάζει ένα διαφορετικό ρεπερτόριο πεπτιδίων, ώστε να δίνει στο ανοσοποιητικό σύστημα πολλούς στόχους για να σημαδέψει και να σκοτώσει τα κύτταρα που τους παράγουν.

ΔΙΑΦΑΝΕΙΑ 13

Πώς όμως δημιουργείται ένα θραύσμα ιού; Οι ιικές πρωτεΐνες -όπως και κάθε άλλη πρωτεΐνη μέσα στα κύτταρα- αποικοδομούνται. Οι πρωτεΐνες τεμαχίζονται από μια εκπληκτική νανομηχανή που ονομάζεται πρωτεάσωμα, η οποία είναι ουσιαστικά ένας κάδος απορριμμάτων για όλες τις πρωτεΐνες. Άλλα κυτταρικά ένζυμα κόβουν τα άκρα των θραυσμάτων σε μικρότερα πεπτίδια, μερικά από τα οποία μεταφέρονται από το κυτταρόπλασμα στο ενδοπλασματικό δίκτυο (Ε/Δ) όπου μπορούν να συνδέθουν με τα μόρια HLA. Μόλις ένα μόριο HLA δεσμεύσει ένα πεπτίδιο, φεύγει από το Ε/Δ για την επιφάνεια του κυττάρου όπου περιμένει την ανίχνευσή του από τα Τ-φονικά κύτταρα.

ΔΙΑΦΑΝΕΙΑ 14

Ας επιστρέψουμε στον πολυμορφισμό HLA. Όπως όλοι γνωρίζουμε από το COVID-19 και τη γρίπη, οι ιοί είναι πολύ καλοί στο να αλλάζουν για να ξεφύγουν από την αντίδραση των αντισωμάτων (σκεφτείτε άλφα, δέλτα, όμικρον....). Για να ελαχιστοποιηθεί αυτή η πιθανότητα για τα Τ κύτταρα, κάθε ένα από τα διαφορετικά αλληλόμορφα MHC (ποικιλίες γονιδίων) παρουσιάζει ένα διαφορετικό σύνολο πεπτιδίων. Παρουσιάζονται τόσα πολλά πεπτίδια σε ένα άτομο, ώστε να είναι δύσκολη η διαφυγή του ιού. Οι διαφορές στους τύπους HLA μεταξύ των ανθρώπων εξασφαλίζουν ότι ακόμη και αν αυτό συμβεί, ο ιός που ξεφεύγει δεν θα συνεχίσει την παραπλάνησή του στο επόμενο άτομο. Αν όλοι μας είχαμε πανομοιότυπα HLA, ένας ιός που διαφεύγει θα σκότωνε ολόκληρο τον πληθυσμό, τώρα θα σκοτώσει "μόνο" μερικά άτομα με μόρια HLA που δεν μπορούν να παρουσιάσουν τα ιικά πεπτίδια στο ανοσοποιητικό σύστημα. Ο πολυμορφισμός HLA προστατεύει έτσι σε επίπεδο πληθυσμού, το άτομο είναι λιγότερο σημαντικό. Αυτό παρέχει μια πειστική εξήγηση για την εξέλιξη του πολυμορφισμού MHC.

ΔΙΑΦΑΝΕΙΑ 15

Αλλά δυστυχώς, κακά τα νέα για σένα, αγαπητέ αναγνώστη, αν τυχαίνει να χρειάζεσαι ένα ή δύο νέα όργανα. Ο πολυμορφισμός HLA προάγει την επιβίωση του πληθυσμού ενός είδους, όχι ενός ατόμου με νεφροπάθεια. Η αποτυχία της μεταμόσχευσης είναι η συνέπεια του ανοσοποιητικού συστήματος που μπερδεύει ένα όργανο δότη με ένα όργανο μολυσμένο από ιό και έτσι αντιδρά αναλόγως, επιτίθεται στο όργανο με αποτέλεσμα την απόρριψη του μοσχεύματος.

ΔΙΑΦΑΝΕΙΑ 16

Ένα σημαντικό γενικό μάθημα: τίποτα, συμπεριλαμβανομένου του ανοσοποιητικού συστήματος, δεν είναι τέλειο! Μιλώντας γι' αυτό, ας σκεφτούμε πώς τα Τ-φονικά κύτταρα μπορούν να βρουν τα μολυσμένα από ιούς κύτταρα αρκετά γρήγορα ώστε να φανούν χρήσιμα. Οι ιοί μπορούν να παράγουν τους απογόνους τους πολύ γρήγορα, σε ορισμένες περιπτώσεις μέσα σε λίγες μόνο ώρες. Αυτό όμως δεν είναι αρκετά γρήγορο και θα πρέπει να περιμένουμε πολύ για να αποικοδομηθούν οι ιικές πρωτεΐνες στο τέλος της φυσικής τους ζωής. Αλλά όπως ακριβώς και το ίδιο το ανοσοποιητικό σύστημα, η σύνθεση των πρωτεϊνών, συμπεριλαμβανομένων και των ιικών πρωτεϊνών, απέχει πολύ από τη τελειότητα. Αυτές οι ατελείς πρωτεΐνες, που ονομάζονται DRiPs, αποικοδομούνται αμέσως, συνδυάζοντας την έναρξη της μόλυνσης από τον ιό με την παρουσίαση του αντιγόνου και επιτρέποντας την αποτελεσματική ανοσοεπιτήρηση από τα Τ-φονικά κύτταρα.

ΔΙΑΦΑΝΕΙΑ 17

Κίνηση ματ από το ανοσοποιητικό σύστημα; Όχι τόσο γρήγορα! Ορισμένοι έξυπνοι ιοί, ιδίως οι ερπητοϊοί, έχουν εξελιχθεί έτσι ώστε να παρεμποδίζουν την παρουσίαση των αντιγόνων. Ο ανθρώπινος κυτταρομεγαλοϊός HCMV, ο οποίος μολύνει το 60% της ανθρωπότητας, παράγει μια σειρά από πρωτεΐνες (US2, US3, US6, US11 και US18) που περιορίζουν την παραγωγή πεπτιδίων ή διαταράσσουν τη λειτουργία του HLA τάξης I.

ΔΙΑΦΑΝΕΙΑ 18

Είναι τότε δυνατόν ορισμένα αλληλόμορφα HLA να αντιμετωπίζουν καλύτερα τις λοιμώξεις από ιούς από ό,τι άλλα; Πράγματι, ορισμένα αλληλόμορφα HLA-B προστατεύουν καλύτερα από τον HIV, ενώ άλλα είναι καλύτερα για το Covid. Τα διαφορετικά αλληλόμορφα HLA έχουν επιλεγεί κατά τη διάρκεια των αιώνων για να αντιμετωπίζουν διαφορετικά παθογόνα. Για παράδειγμα, το HLA-A2 απαντάται στο 40% του ευρωπαϊκού πληθυσμού, και έχει την υψηλότερη συχνότητα αλληλόμορφου HLA σε μια δεδομένη ομάδα. Αυτό πιθανότατα προκύπτει από την ικανότητα του HLA-A2 να προστατεύει από ένα παθογόνο που εμφανίστηκε κάποτε στο παρελθόν, το οποίο μπορεί να μην αποτελεί πλέον κύρια αιτία ανθρώπινης ασθένειας.

ΔΙΑΦΑΝΕΙΑ 19

Αλλά υπάρχουν παράπλευρες συνέπειες. Για παράδειγμα, το αλληλόμορφο HLA-B*27:05, παρουσιάζεται στο 8% του καυκάσιου πληθυσμού και πάνω από το 90% των ασθενών με Αγκυλοποιητική Σπονδυλίτιδα έχουν αυτό το αλληλόμορφο, το οποίο πιθανότατα πυροδοτεί μια αυτοάνοση αντίδραση Τ-κυττάρων στη σπονδυλική στήλη. Το ανοσοποιητικό σύστημα λειτουργεί στην κόψη του ξυραφιού μεταξύ της παροχής αποτελεσματικής ανοσίας και της μη καταστροφής των ιστών από τα φιλικά πυρά.

ΔΙΑΦΑΝΕΙΑ 20

Η αυτοανοσία των T-κυττάρων μπορεί επίσης να είναι ευεργετική. Τα καρκινικά κύτταρα έχουν συνήθως πολλές μεταλλάξεις και άλλες αλλοιώσεις που οδηγούν στη δημιουργία πεπτιδίων που διαφέρουν από τα φυσιολογικά κυτταρικά πεπτίδια. Η ανοσοθεραπεία του καρκίνου εκμεταλλεύεται μηχανισμούς που χρησιμοποιεί το ανοσοποιητικό σύστημα στην αναγνώριση ιογενών και βακτηριακών λοιμώξεων για να σκοτώσει τα καρκινικά κύτταρα.

ΔΙΑΦΑΝΕΙΑ 21

Τι γίνεται όμως με τα μόρια HLA-DR, -DQ και -DP του MHC τάξης II; Αυτά τα μόρια παρουσιάζουν παθογόνα πεπτίδια στα Τ-βοηθητικά κύτταρα, τα οποία στη συνέχεια παράγουν κυτταροκίνες για να βοηθήσουν τα Β κύτταρα να διαφοροποιηθούν σε εργοστάσια παραγωγής αντισωμάτων. Τα Τ-βοηθητικά βοηθούν επίσης στη βελτιστοποίηση των αποκρίσεων των Τ-φονικών κυττάρων.

Τα MHC τάξης II μοιάζουν πολύ στο σχήμα με τα MHC τάξης I, αλλά παρουσιάζουν πρωτεϊνικά θραύσματα που είναι μακρύτερα και κατασκευάζονται στα λυσοσώματα, τα οποία είναι μικρά οργανίδια που αποδομούν τις πρωτεΐνες που αποκτώνται από το εξωτερικό των κυττάρων.

ΔΙΑΦΑΝΕΙΑ 22

Πώς το κάνουν αυτό; Τα μόρια MHC τάξης II παράγονται στο Ε/Δ (όπως και κάθε άλλη πρωτεΐνη που πρέπει να πάει στην εξωτερική μεμβράνη του κυττάρου ή στα λυσοσώματα), όπου συνδέονται με μια μη πολυμορφική αλυσίδα (invariant chain) που μιμείται ένα πεπτίδιο και συνοδεύει τα MHC τάξης II προς το λυσόσωμα. Εδώ, η μη πολυμορφική αλυσίδα απομακρύνεται και ανταλλάσσεται με ένα πεπτίδιο που δημιουργείται από τα λυσοσωμικά ένζυμα. Η διαδικασία αυτή βελτιστοποιείται από έναν ακόμη τύπο μορίου MHC (HLA-DM, το οποίο μοιάζει με το MHC τάξης II και σε ορισμένα κύτταρα λειτουργεί σε συνεργασία με το HLA-DO, ένα άλλο μόριο που μοιάζει με την τάξη II. Αυτό δείχνει ότι η εξέλιξη είναι οκνηρή, όταν έχει αναπτύξει ένα λειτουργικό μόριο, απλώς το αντιγράφει και το τροποποιεί για νέες λειτουργίες). Το καθαρό αποτέλεσμα αυτού του περίπλοκου χορού είναι η παράδοση των μορίων MHC τάξης II στην επιφάνεια των κυττάρων με πεπτίδια που επιτρέπουν την ενεργοποίηση των Τ-βοηθητικών κυττάρων.

ΔΙΑΦΑΝΕΙΑ 23

Αυτή η διαδικασία αναγνώρισης των παθογόνων από το ανοσοποιητικό σύστημα είναι πολύπλοκη… αλλά και σχετικά αργή. Την πρώτη φορά που συναντάτε έναν ιό, το ανοσοποιητικό σύστημα χρειάζεται χρόνο για να ενισχύσει την αντι-ιική του δράση. Αν είστε άτυχοι, αυτό μπορεί να οδηγήσει σε ασθένεια ή θάνατο από τον ανεξέλεγκτο πολλαπλασιασμό του ιού. Ο εμβολιασμός προετοιμάζει το ανοσοποιητικό σύστημα για μια λοίμωξη, επιτρέποντάς του σε ορισμένες περιπτώσεις να την αποτρέψει εντελώς, ενώ σε άλλες να ανταποκριθεί πιο γρήγορα και αποτελεσματικά και να μειώσει σημαντικά τις πιθανότητες μιας σοβαρής μορφής της.

ΔΙΑΦΑΝΕΙΑ 24

Τα μόρια MHC είναι σημαντικοί συμμετέχοντες στον εμβολιασμό. Όλα τα εμβόλια χρησιμοποιούν τα μόρια MHC τάξης II για την επαγωγή των Τ-βοηθητικών κυττάρων που απαιτούνται για τη χυμική αποκρίση και παράγουν τις πρωτεϊνες κατά των οποίων στρέφονται τα αντισώματα. Τα εμβόλια αδενοϊού και mRNA χρησιμοποιούν επίσης μόρια MHC τάξης I για να επάγουν τα Τ-φονικά κύτταρα. Τα Τ-κύτταρα που επάγονται από τα εμβόλια παραμένουν για πολλά χρόνια, ακόμη και δεκαετίες σε ορισμένες περιπτώσεις, σε επιφυλακή για μια νέα μόλυνση με τον αρχικό ιό. Τα εμβόλια έχουν σώσει πολύ περισσότερες ζωές από όλες τις άλλες ιατρικές παρεμβάσεις μαζί. Διαδώστε αυτό το μήνυμα, όχι την ασθένεια, εμβολιαστείτε!

ΕΠΙΛΟΓΟΣ

Έτσι, τα μόρια MHC ελέγχουν τις λοιμώξεις, ρυθμίζουν τις ανοσολογικές αποκρίσεις και τώρα βοηθούν και στη θεραπεία του καρκίνου. Αυτό αξίζει το τίμημα της αυτοανοσίας και της απόρριψης των μοσχευμάτων. Και αυτός είναι ο λόγος για τον οποίο εσείς -που ζείτε σε έναν κόσμο γεμάτο παθογόνους μικροοργανισμούς- επιζήσατε για να διαβάσετε αυτό το κόμικ. Για περισσότερες λεπτομέρειες σχετικά με το πώς να επιβιώσετε ακόμη καλύτερα, ανατρέξτε στις αναφορές 1-6.
