# Supplementary material for: HLA molecules in transplantation, autoimmunity and infection control: A comic book adventure
Source: HLA. 2022 May 15;100(4):301–11. doi: 10.1111/tan.14626 (PMC9545814; doi:10.1111/tan.14626)
Supplement: Supplementary file 1 — Supporting information. [file TAN-100-301-s001.zip › Supplementary files/PP_Haags.1.pptx]

## Slide 1
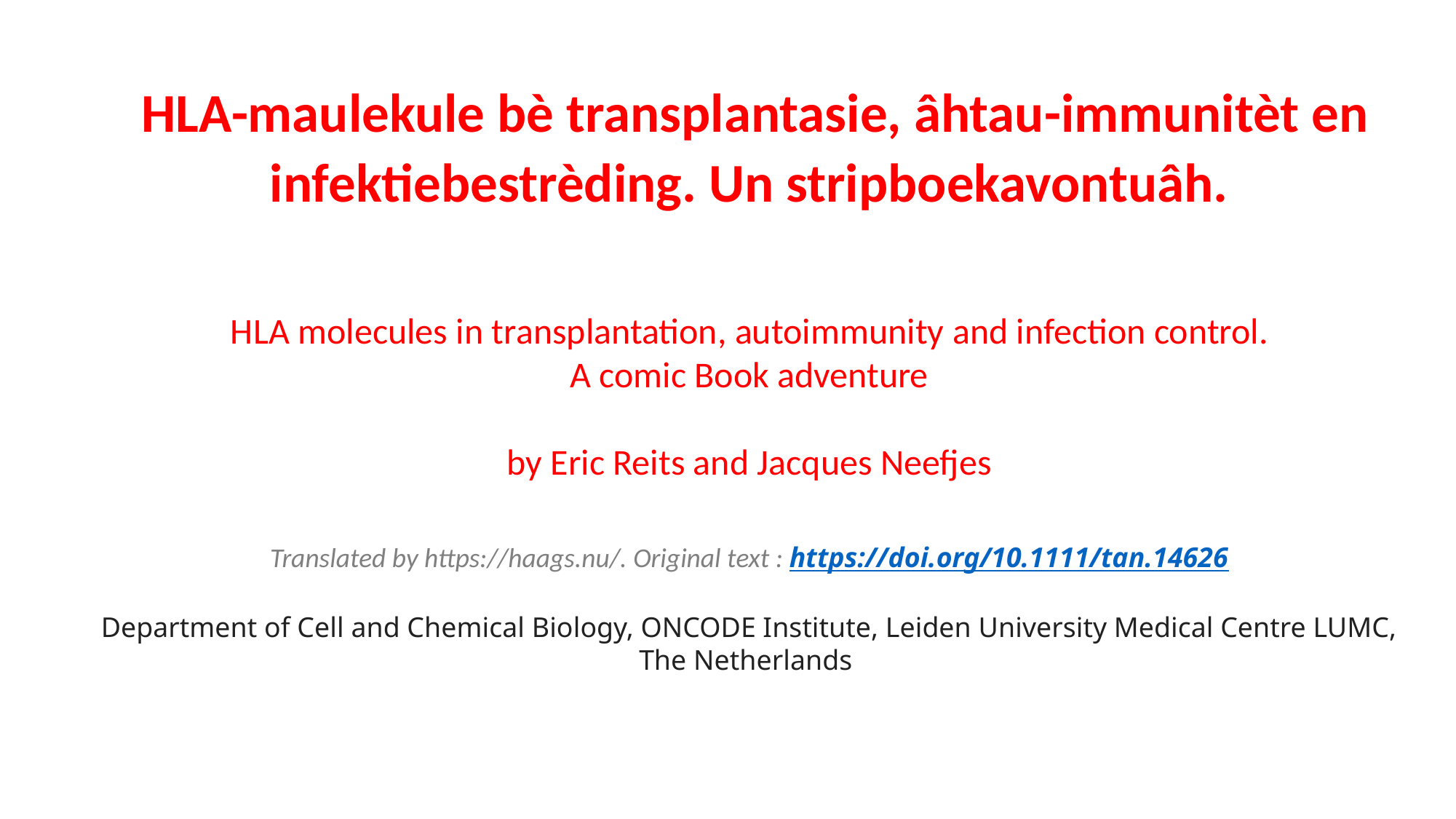

HLA-maulekule bè transplantasie, âhtau-immunitèt en infektiebestrèding. Un stripboekavontuâh.
HLA molecules in transplantation, autoimmunity and infection control.
A comic Book adventure
by Eric Reits and Jacques Neefjes
Translated by https://haags.nu/. Original text : https://doi.org/10.1111/tan.14626
Department of Cell and Chemical Biology, ONCODE Institute, Leiden University Medical Centre LUMC, The Netherlands

## Slide 2
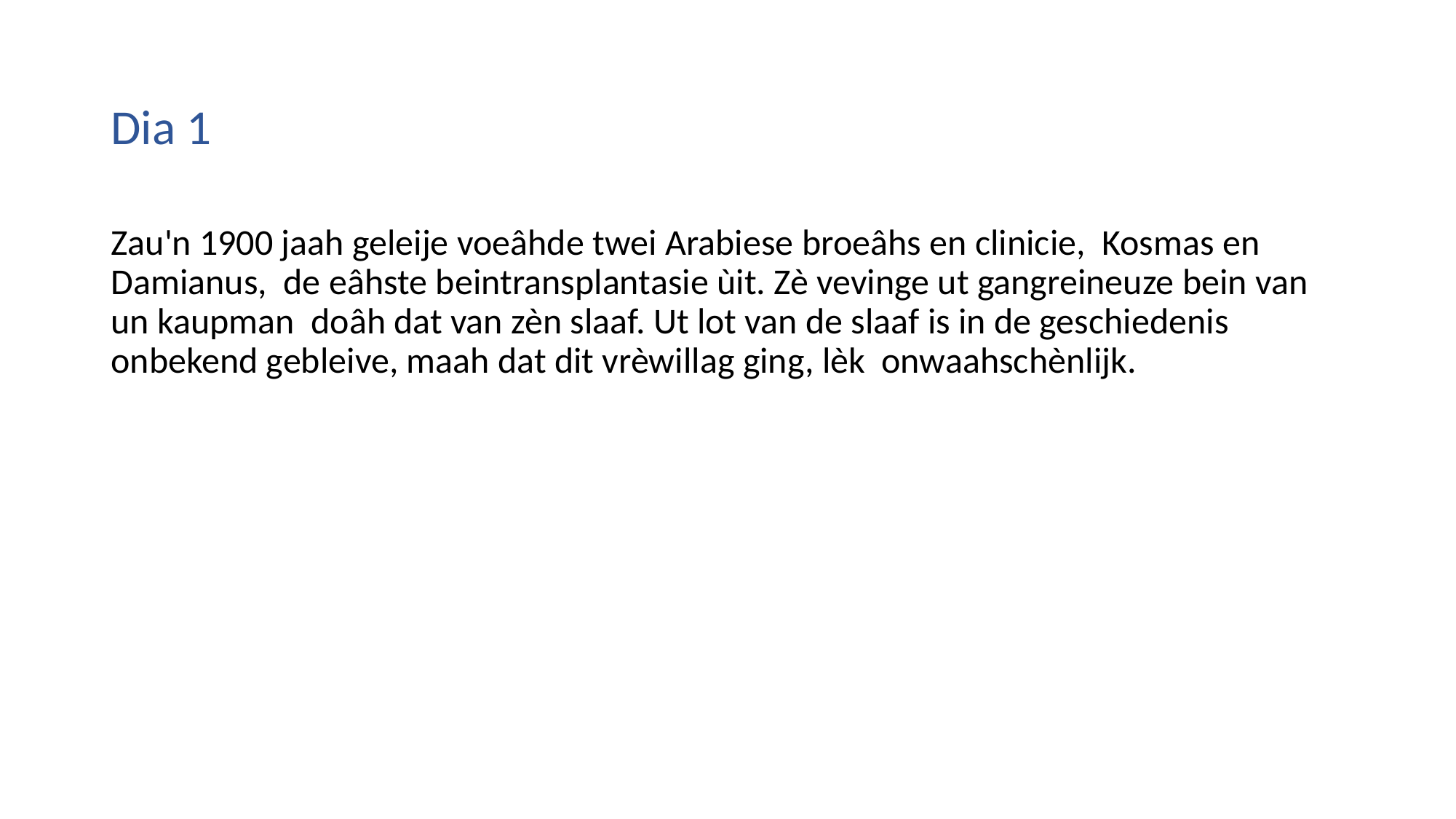

# Dia 1
Zau'n 1900 jaah geleije voeâhde twei Arabiese broeâhs en clinicie,  Kosmas en Damianus,  de eâhste beintransplantasie ùit. Zè vevinge ut gangreineuze bein van un kaupman  doâh dat van zèn slaaf. Ut lot van de slaaf is in de geschiedenis onbekend gebleive, maah dat dit vrèwillag ging, lèk  onwaahschènlijk.

## Slide 3
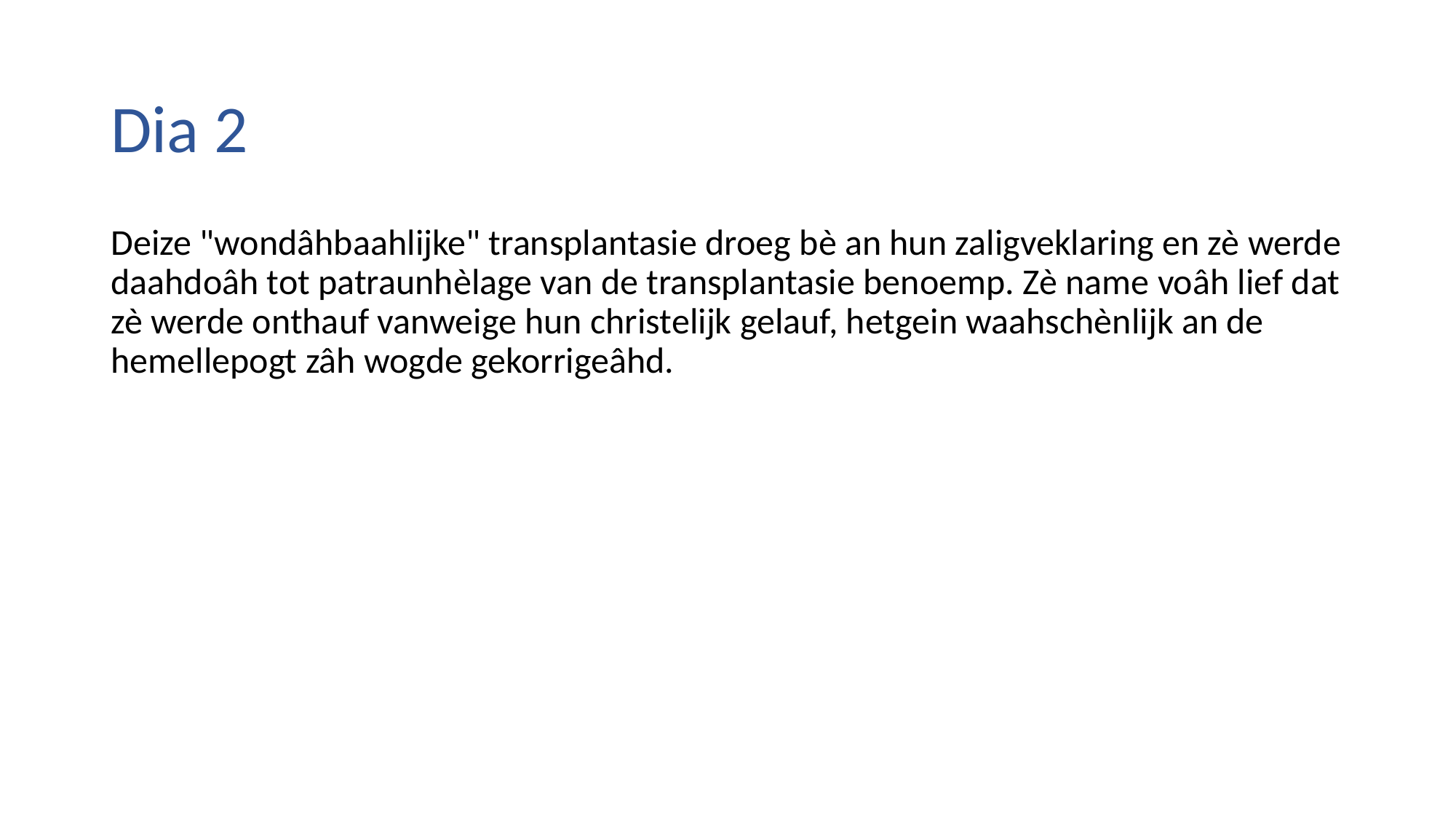

# Dia 2
Deize "wondâhbaahlijke" transplantasie droeg bè an hun zaligveklaring en zè werde daahdoâh tot patraunhèlage van de transplantasie benoemp. Zè name voâh lief dat zè werde onthauf vanweige hun christelijk gelauf, hetgein waahschènlijk an de hemellepogt zâh wogde gekorrigeâhd.

## Slide 4
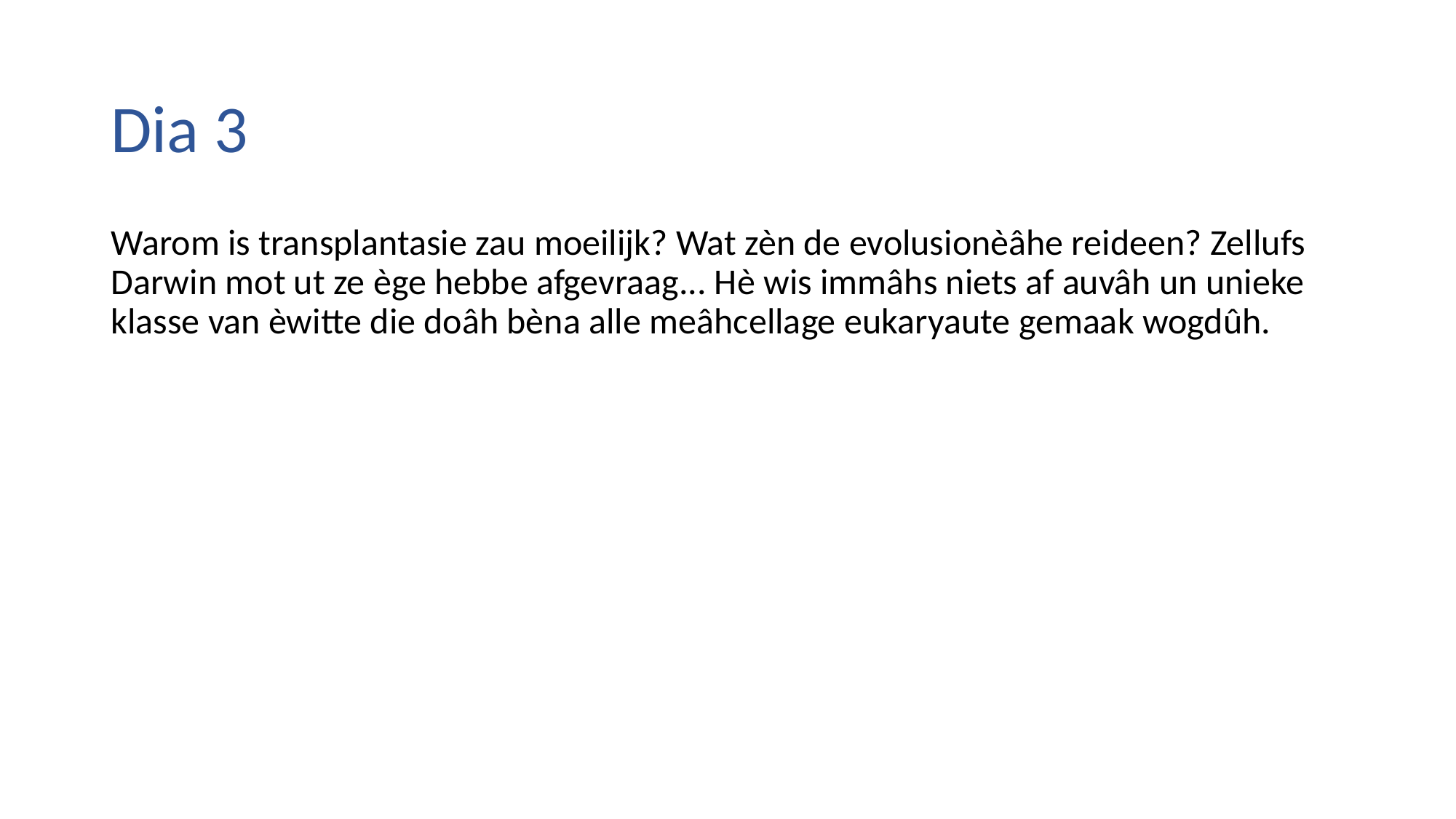

# Dia 3
Warom is transplantasie zau moeilijk? Wat zèn de evolusionèâhe reideen? Zellufs Darwin mot ut ze ège hebbe afgevraag... Hè wis immâhs niets af auvâh un unieke klasse van èwitte die doâh bèna alle meâhcellage eukaryaute gemaak wogdûh.

## Slide 5
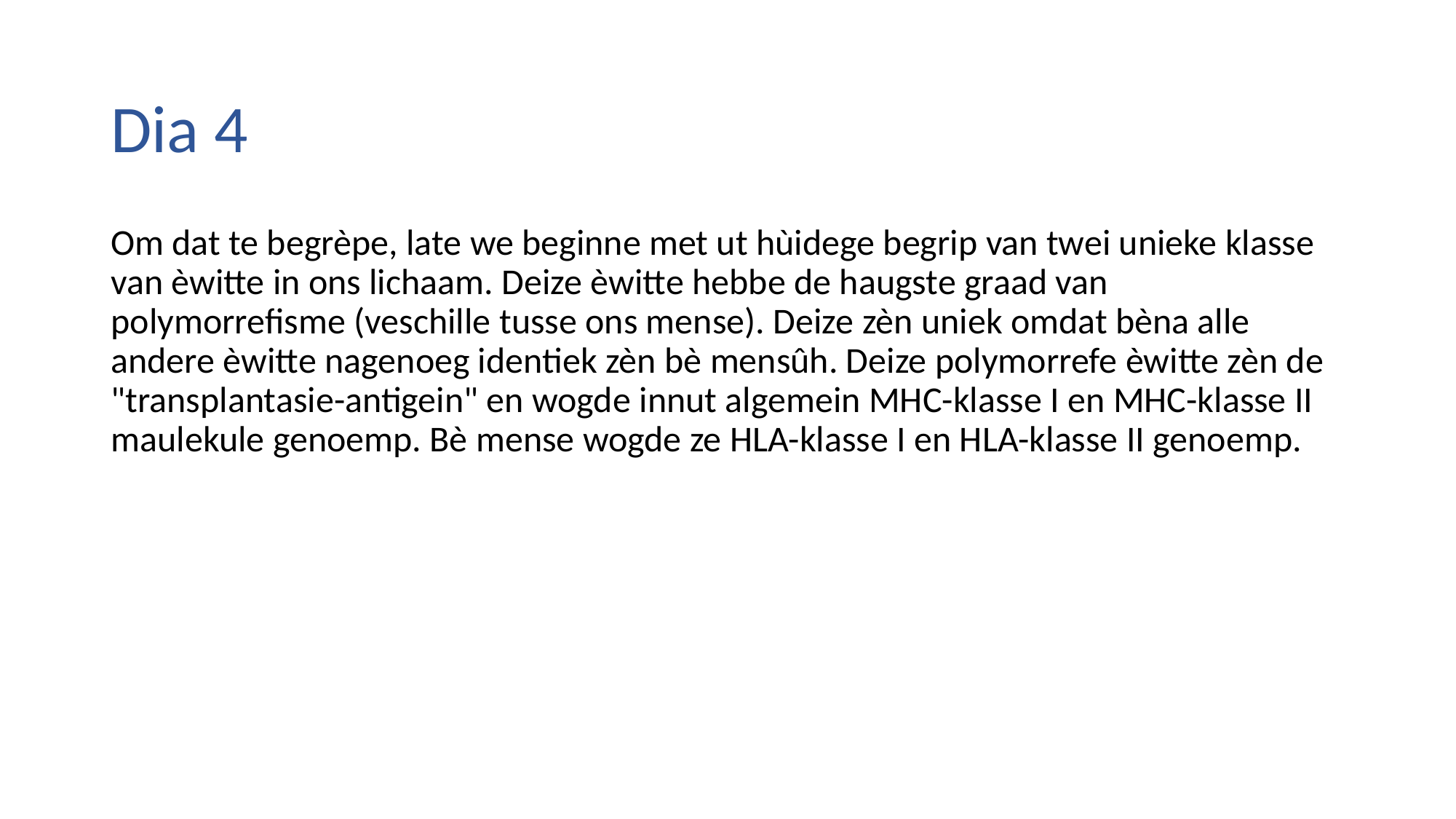

# Dia 4
Om dat te begrèpe, late we beginne met ut hùidege begrip van twei unieke klasse van èwitte in ons lichaam. Deize èwitte hebbe de haugste graad van polymorrefisme (veschille tusse ons mense). Deize zèn uniek omdat bèna alle andere èwitte nagenoeg identiek zèn bè mensûh. Deize polymorrefe èwitte zèn de "transplantasie-antigein" en wogde innut algemein MHC-klasse I en MHC-klasse II maulekule genoemp. Bè mense wogde ze HLA-klasse I en HLA-klasse II genoemp.

## Slide 6
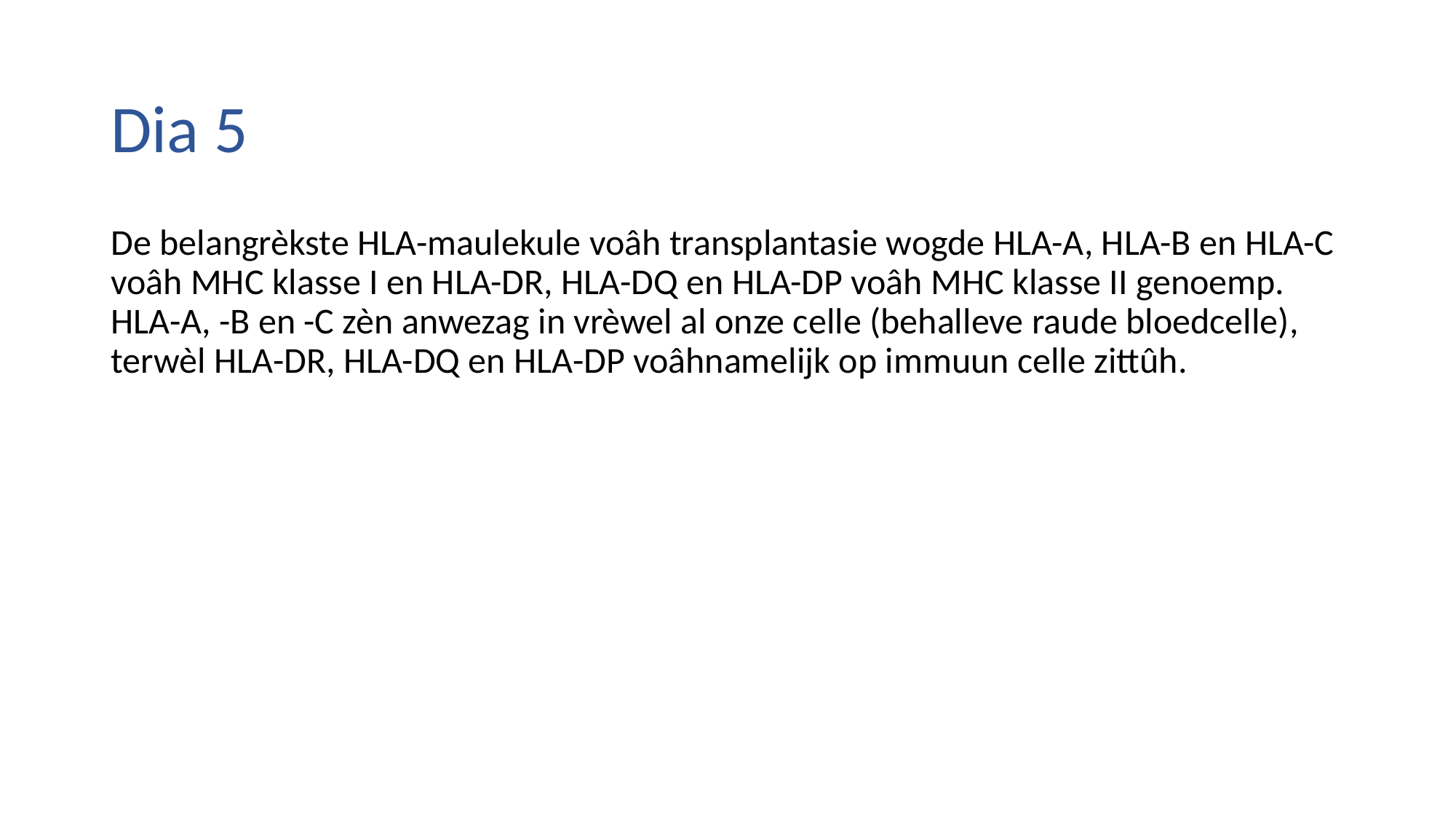

# Dia 5
De belangrèkste HLA-maulekule voâh transplantasie wogde HLA-A, HLA-B en HLA-C voâh MHC klasse I en HLA-DR, HLA-DQ en HLA-DP voâh MHC klasse II genoemp. HLA-A, -B en -C zèn anwezag in vrèwel al onze celle (behalleve raude bloedcelle), terwèl HLA-DR, HLA-DQ en HLA-DP voâhnamelijk op immuun celle zittûh.

## Slide 7
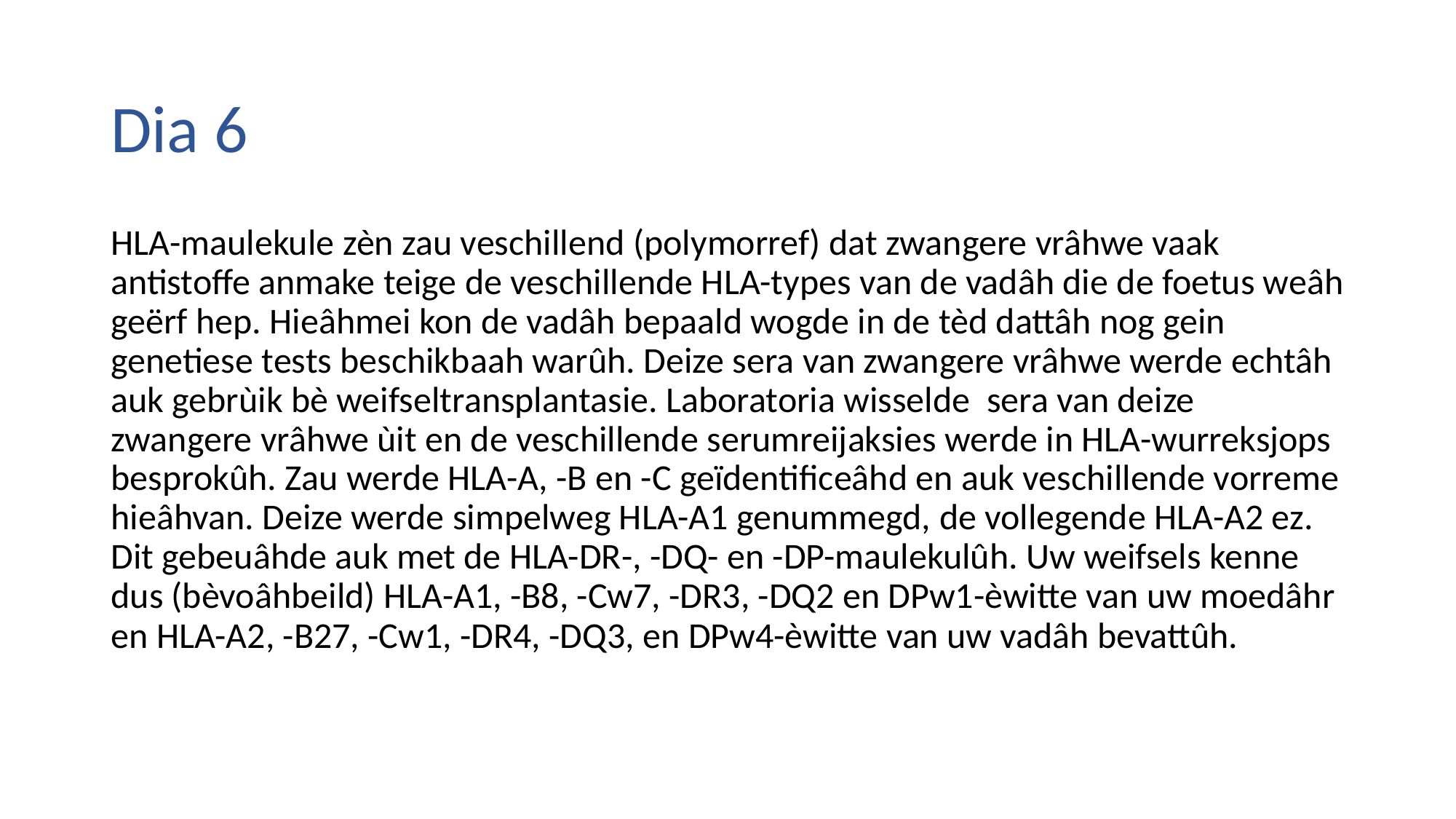

# Dia 6
HLA-maulekule zèn zau veschillend (polymorref) dat zwangere vrâhwe vaak antistoffe anmake teige de veschillende HLA-types van de vadâh die de foetus weâh geërf hep. Hieâhmei kon de vadâh bepaald wogde in de tèd dattâh nog gein genetiese tests beschikbaah warûh. Deize sera van zwangere vrâhwe werde echtâh auk gebrùik bè weifseltransplantasie. Laboratoria wisselde  sera van deize zwangere vrâhwe ùit en de veschillende serumreijaksies werde in HLA-wurreksjops besprokûh. Zau werde HLA-A, -B en -C geïdentificeâhd en auk veschillende vorreme hieâhvan. Deize werde simpelweg HLA-A1 genummegd, de vollegende HLA-A2 ez. Dit gebeuâhde auk met de HLA-DR-, -DQ- en -DP-maulekulûh. Uw weifsels kenne dus (bèvoâhbeild) HLA-A1, -B8, -Cw7, -DR3, -DQ2 en DPw1-èwitte van uw moedâhr en HLA-A2, -B27, -Cw1, -DR4, -DQ3, en DPw4-èwitte van uw vadâh bevattûh.

## Slide 8
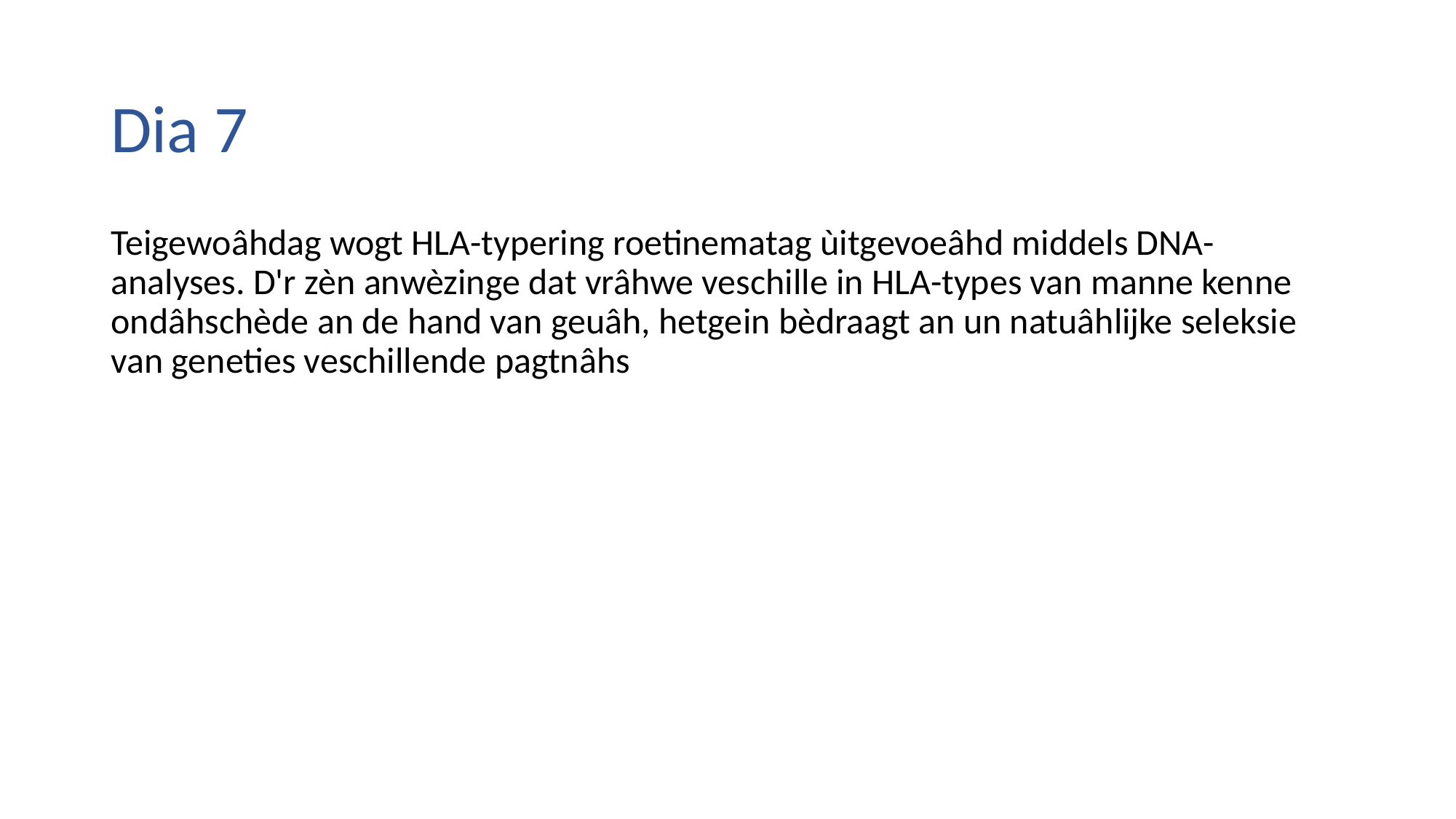

# Dia 7
Teigewoâhdag wogt HLA-typering roetinematag ùitgevoeâhd middels DNA-analyses. D'r zèn anwèzinge dat vrâhwe veschille in HLA-types van manne kenne ondâhschède an de hand van geuâh, hetgein bèdraagt an un natuâhlijke seleksie van geneties veschillende pagtnâhs

## Slide 9
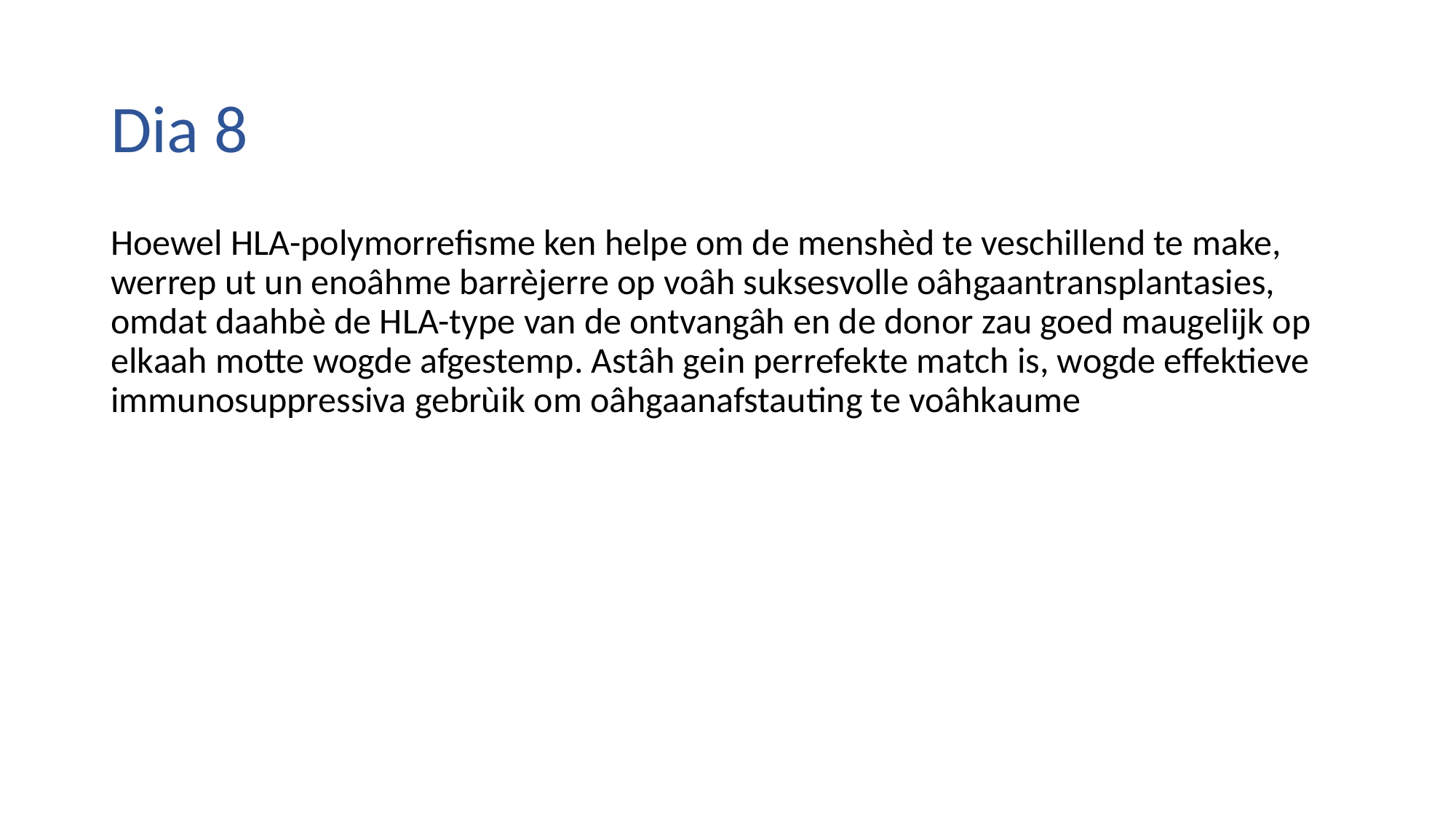

# Dia 8
Hoewel HLA-polymorrefisme ken helpe om de menshèd te veschillend te make, werrep ut un enoâhme barrèjerre op voâh suksesvolle oâhgaantransplantasies, omdat daahbè de HLA-type van de ontvangâh en de donor zau goed maugelijk op elkaah motte wogde afgestemp. Astâh gein perrefekte match is, wogde effektieve immunosuppressiva gebrùik om oâhgaanafstauting te voâhkaume

## Slide 10
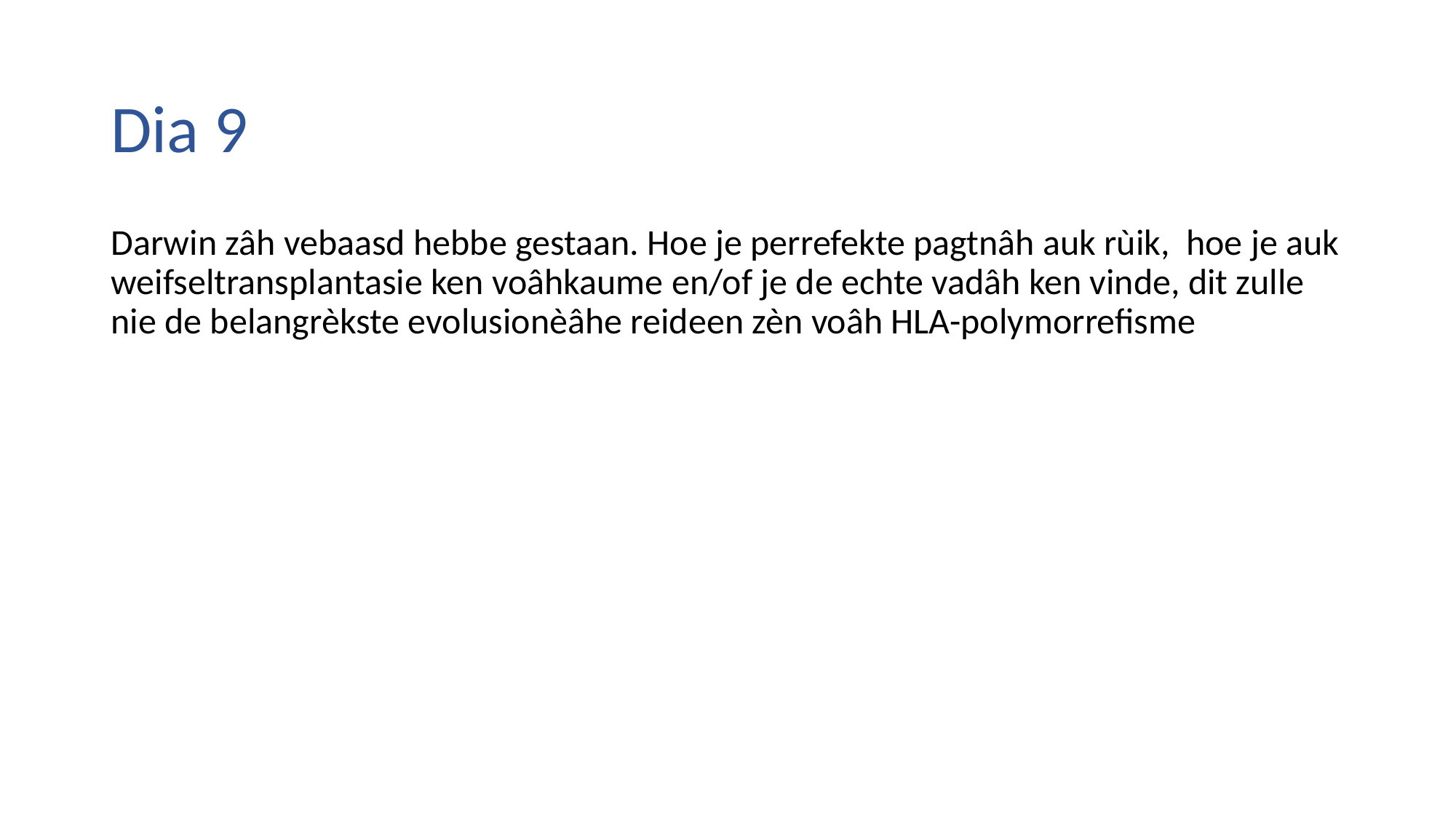

# Dia 9
Darwin zâh vebaasd hebbe gestaan. Hoe je perrefekte pagtnâh auk rùik,  hoe je auk weifseltransplantasie ken voâhkaume en/of je de echte vadâh ken vinde, dit zulle nie de belangrèkste evolusionèâhe reideen zèn voâh HLA-polymorrefisme

## Slide 11
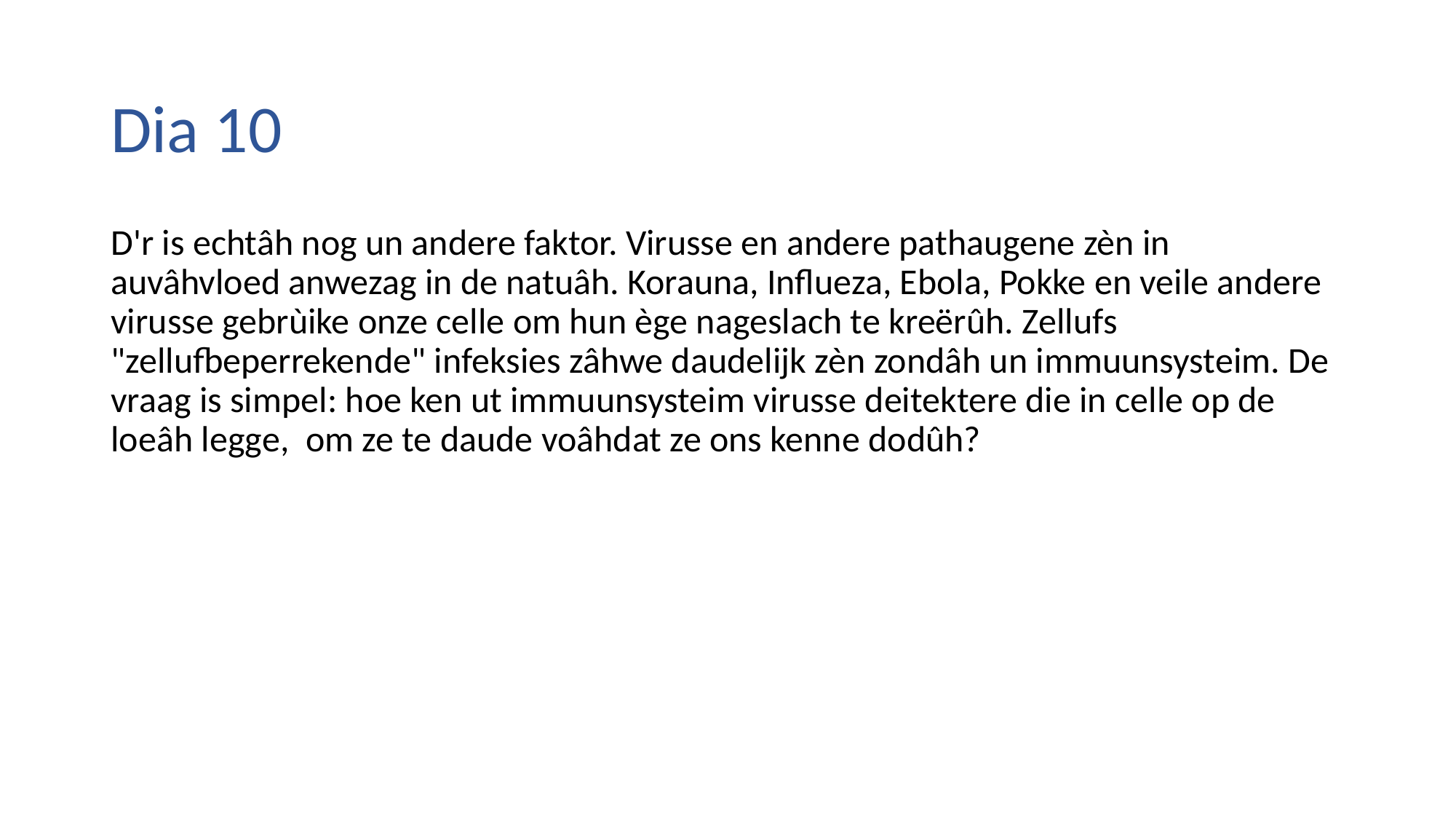

# Dia 10
D'r is echtâh nog un andere faktor. Virusse en andere pathaugene zèn in auvâhvloed anwezag in de natuâh. Korauna, Influeza, Ebola, Pokke en veile andere virusse gebrùike onze celle om hun ège nageslach te kreërûh. Zellufs "zellufbeperrekende" infeksies zâhwe daudelijk zèn zondâh un immuunsysteim. De vraag is simpel: hoe ken ut immuunsysteim virusse deitektere die in celle op de loeâh legge,  om ze te daude voâhdat ze ons kenne dodûh?

## Slide 12
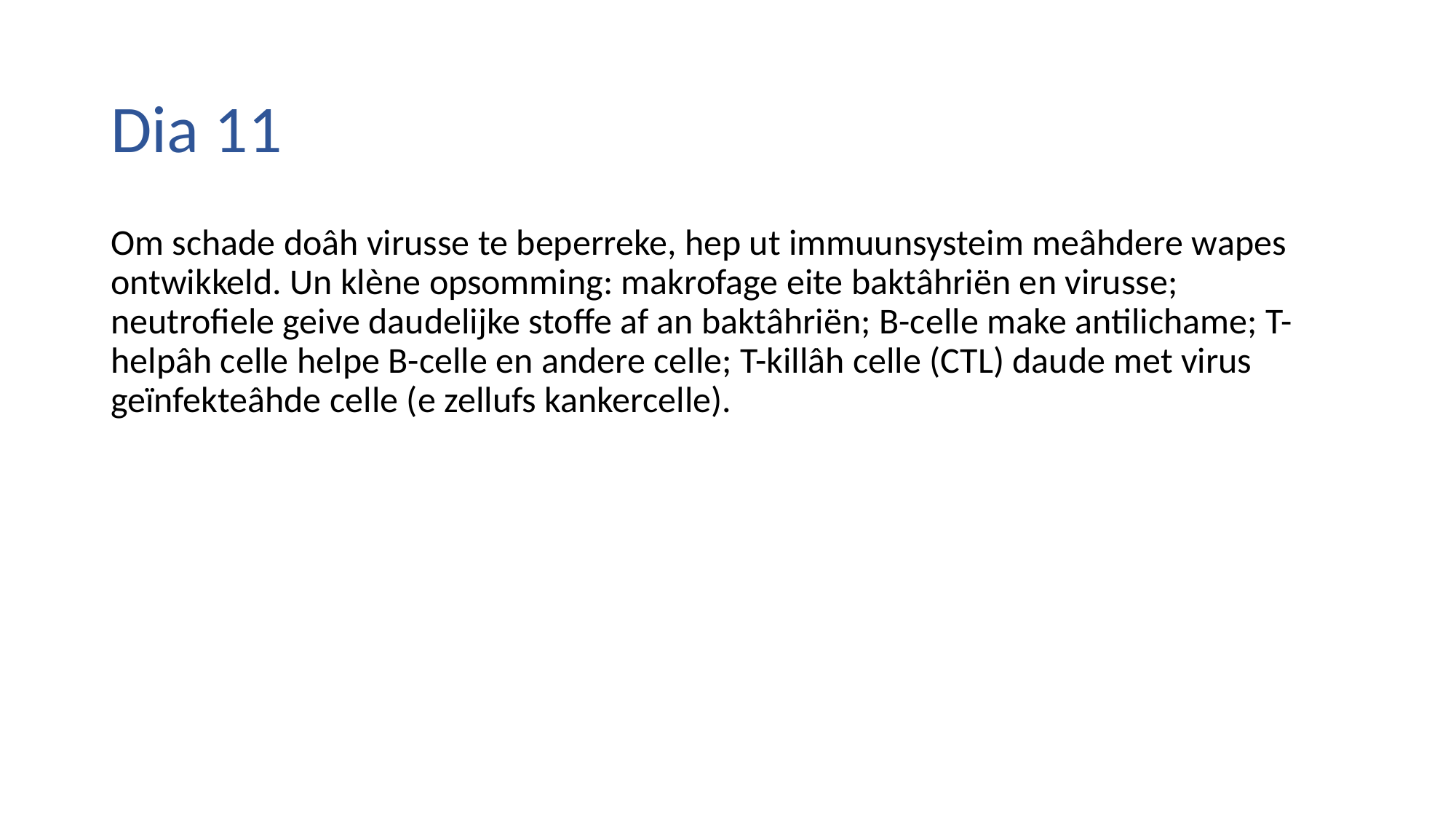

# Dia 11
Om schade doâh virusse te beperreke, hep ut immuunsysteim meâhdere wapes ontwikkeld. Un klène opsomming: makrofage eite baktâhriën en virusse; neutrofiele geive daudelijke stoffe af an baktâhriën; B-celle make antilichame; T-helpâh celle helpe B-celle en andere celle; T-killâh celle (CTL) daude met virus geïnfekteâhde celle (e zellufs kankercelle).

## Slide 13
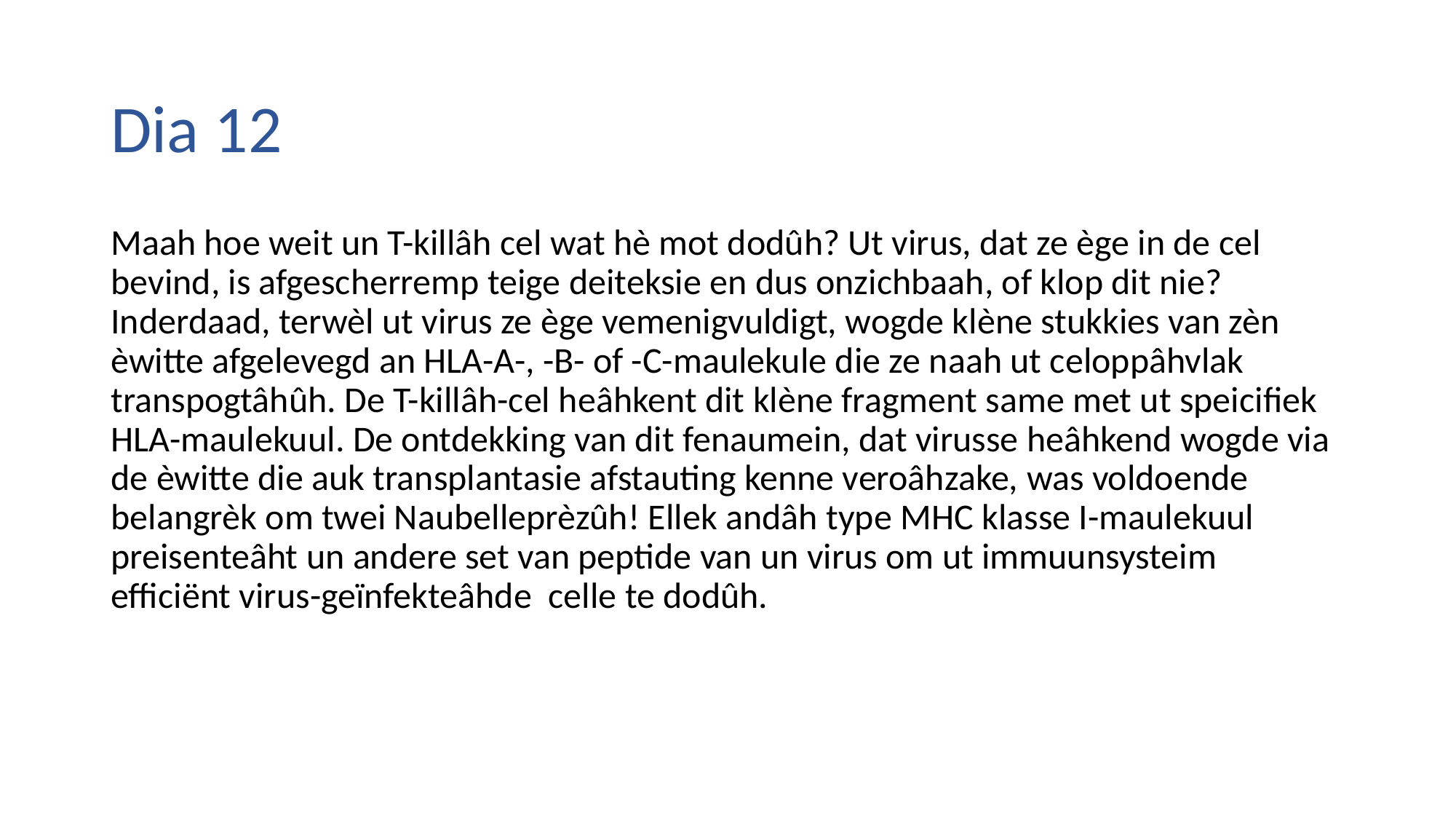

# Dia 12
Maah hoe weit un T-killâh cel wat hè mot dodûh? Ut virus, dat ze ège in de cel bevind, is afgescherremp teige deiteksie en dus onzichbaah, of klop dit nie? Inderdaad, terwèl ut virus ze ège vemenigvuldigt, wogde klène stukkies van zèn èwitte afgelevegd an HLA-A-, -B- of -C-maulekule die ze naah ut celoppâhvlak transpogtâhûh. De T-killâh-cel heâhkent dit klène fragment same met ut speicifiek HLA-maulekuul. De ontdekking van dit fenaumein, dat virusse heâhkend wogde via de èwitte die auk transplantasie afstauting kenne veroâhzake, was voldoende belangrèk om twei Naubelleprèzûh! Ellek andâh type MHC klasse I-maulekuul preisenteâht un andere set van peptide van un virus om ut immuunsysteim efficiënt virus-geïnfekteâhde  celle te dodûh.

## Slide 14
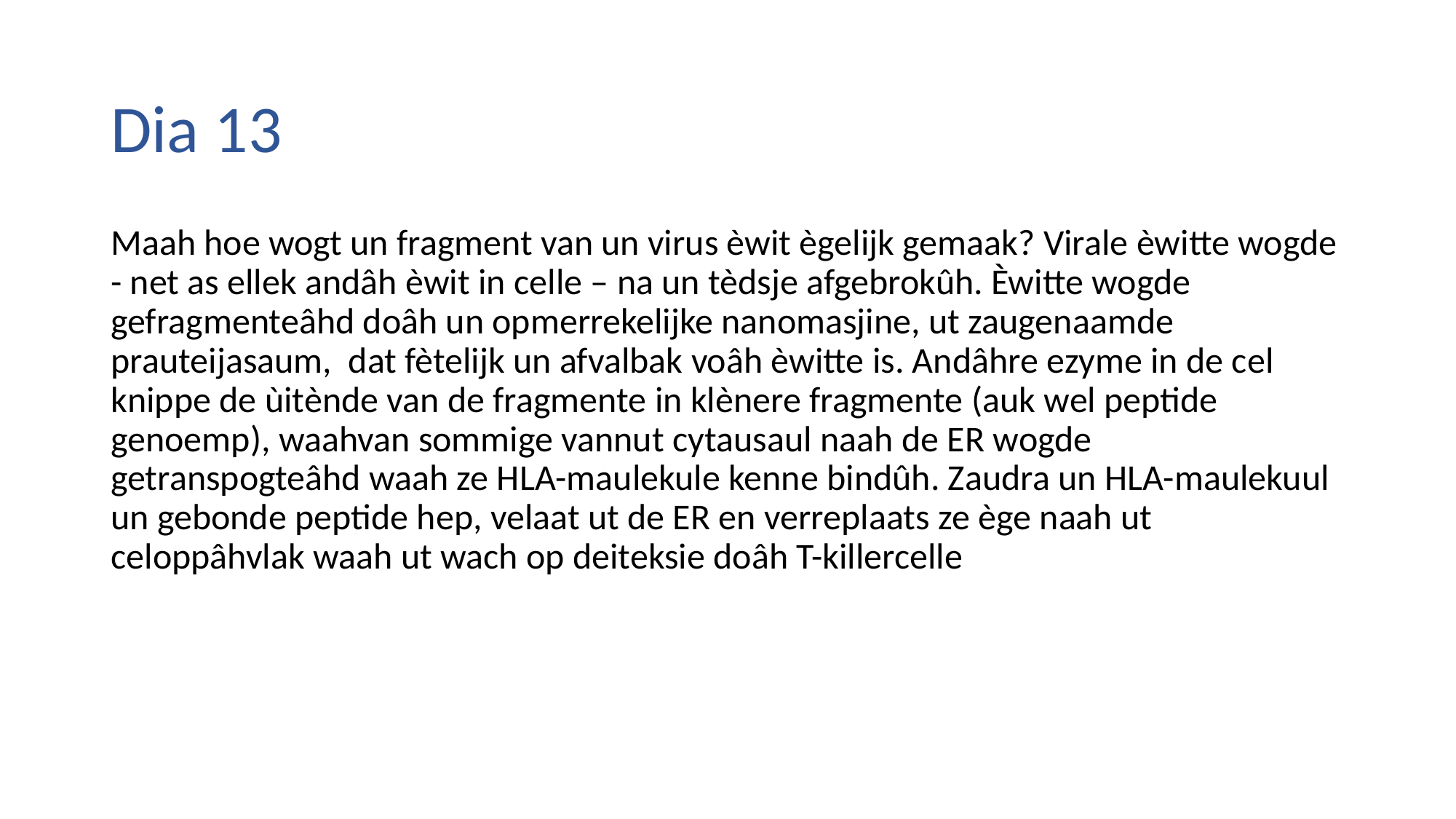

# Dia 13
Maah hoe wogt un fragment van un virus èwit ègelijk gemaak? Virale èwitte wogde - net as ellek andâh èwit in celle – na un tèdsje afgebrokûh. Èwitte wogde gefragmenteâhd doâh un opmerrekelijke nanomasjine, ut zaugenaamde prauteijasaum,  dat fètelijk un afvalbak voâh èwitte is. Andâhre ezyme in de cel knippe de ùitènde van de fragmente in klènere fragmente (auk wel peptide genoemp), waahvan sommige vannut cytausaul naah de ER wogde getranspogteâhd waah ze HLA-maulekule kenne bindûh. Zaudra un HLA-maulekuul un gebonde peptide hep, velaat ut de ER en verreplaats ze ège naah ut celoppâhvlak waah ut wach op deiteksie doâh T-killercelle

## Slide 15
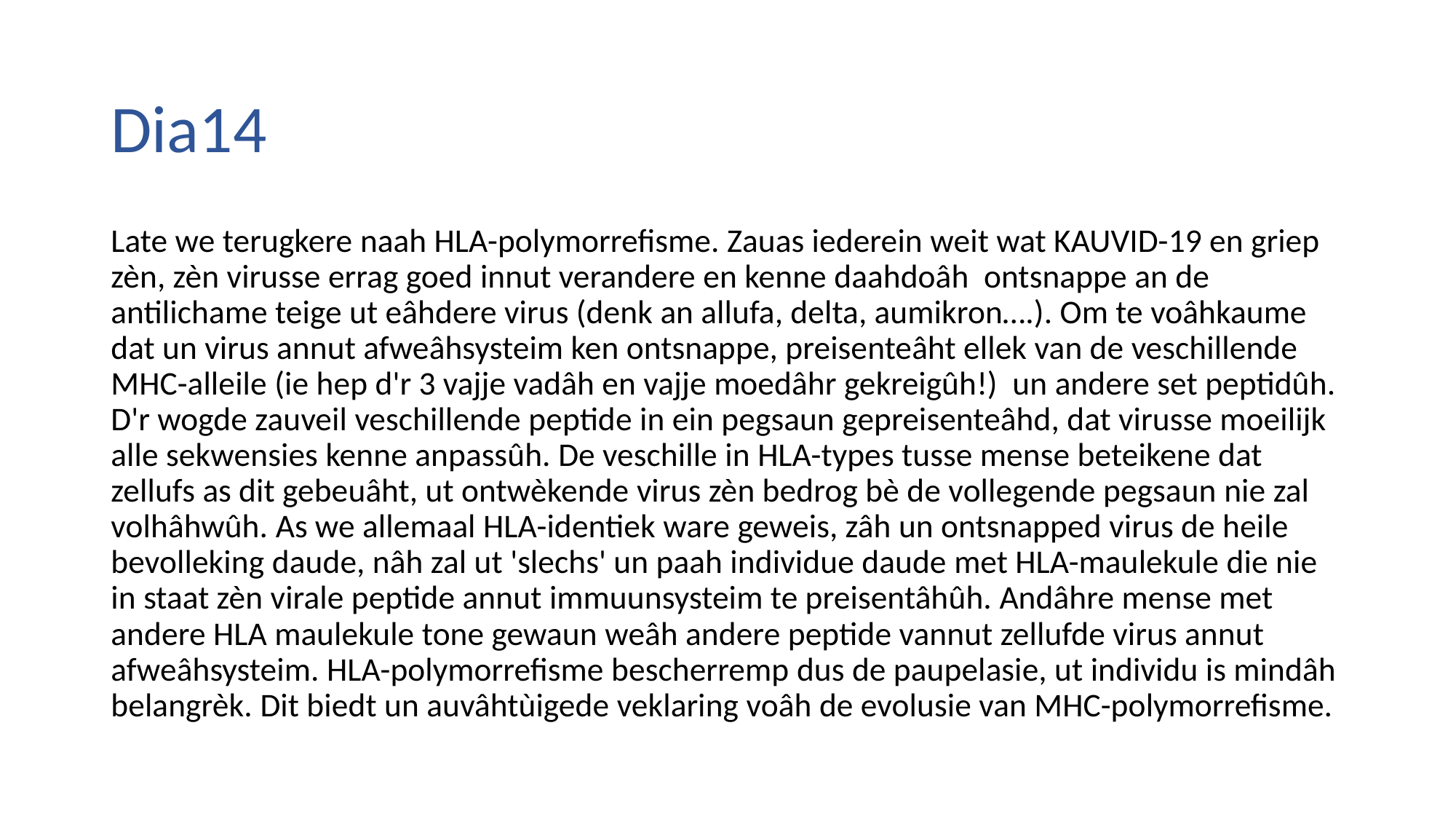

# Dia14
Late we terugkere naah HLA-polymorrefisme. Zauas iederein weit wat KAUVID-19 en griep zèn, zèn virusse errag goed innut verandere en kenne daahdoâh  ontsnappe an de antilichame teige ut eâhdere virus (denk an allufa, delta, aumikron….). Om te voâhkaume dat un virus annut afweâhsysteim ken ontsnappe, preisenteâht ellek van de veschillende MHC-alleile (ie hep d'r 3 vajje vadâh en vajje moedâhr gekreigûh!)  un andere set peptidûh. D'r wogde zauveil veschillende peptide in ein pegsaun gepreisenteâhd, dat virusse moeilijk alle sekwensies kenne anpassûh. De veschille in HLA-types tusse mense beteikene dat zellufs as dit gebeuâht, ut ontwèkende virus zèn bedrog bè de vollegende pegsaun nie zal volhâhwûh. As we allemaal HLA-identiek ware geweis, zâh un ontsnapped virus de heile bevolleking daude, nâh zal ut 'slechs' un paah individue daude met HLA-maulekule die nie in staat zèn virale peptide annut immuunsysteim te preisentâhûh. Andâhre mense met andere HLA maulekule tone gewaun weâh andere peptide vannut zellufde virus annut afweâhsysteim. HLA-polymorrefisme bescherremp dus de paupelasie, ut individu is mindâh belangrèk. Dit biedt un auvâhtùigede veklaring voâh de evolusie van MHC-polymorrefisme.

## Slide 16
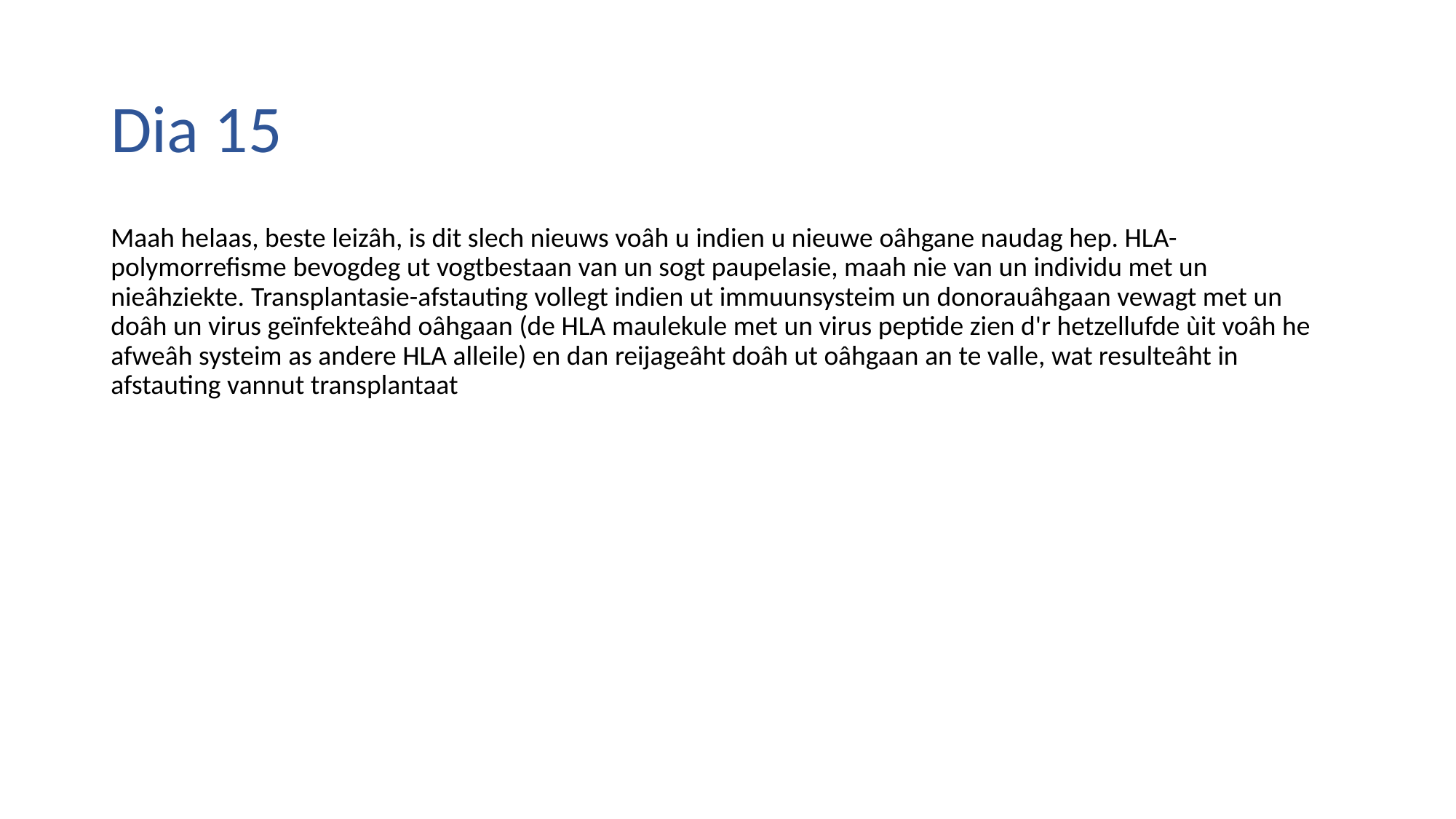

# Dia 15
Maah helaas, beste leizâh, is dit slech nieuws voâh u indien u nieuwe oâhgane naudag hep. HLA-polymorrefisme bevogdeg ut vogtbestaan van un sogt paupelasie, maah nie van un individu met un nieâhziekte. Transplantasie-afstauting vollegt indien ut immuunsysteim un donorauâhgaan vewagt met un doâh un virus geïnfekteâhd oâhgaan (de HLA maulekule met un virus peptide zien d'r hetzellufde ùit voâh he afweâh systeim as andere HLA alleile) en dan reijageâht doâh ut oâhgaan an te valle, wat resulteâht in afstauting vannut transplantaat

## Slide 17
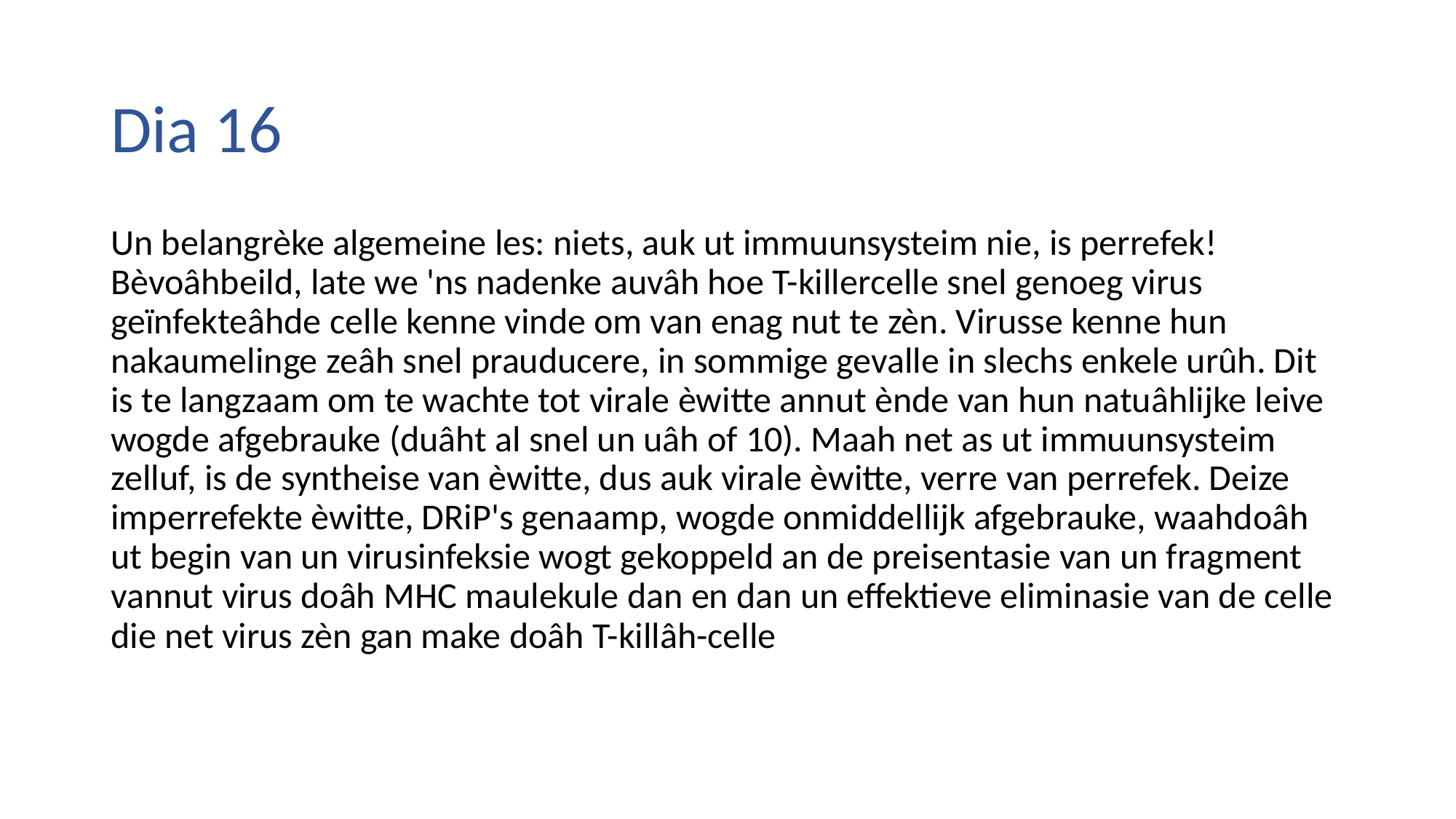

# Dia 16
Un belangrèke algemeine les: niets, auk ut immuunsysteim nie, is perrefek! Bèvoâhbeild, late we 'ns nadenke auvâh hoe T-killercelle snel genoeg virus geïnfekteâhde celle kenne vinde om van enag nut te zèn. Virusse kenne hun nakaumelinge zeâh snel prauducere, in sommige gevalle in slechs enkele urûh. Dit is te langzaam om te wachte tot virale èwitte annut ènde van hun natuâhlijke leive wogde afgebrauke (duâht al snel un uâh of 10). Maah net as ut immuunsysteim zelluf, is de syntheise van èwitte, dus auk virale èwitte, verre van perrefek. Deize imperrefekte èwitte, DRiP's genaamp, wogde onmiddellijk afgebrauke, waahdoâh ut begin van un virusinfeksie wogt gekoppeld an de preisentasie van un fragment vannut virus doâh MHC maulekule dan en dan un effektieve eliminasie van de celle die net virus zèn gan make doâh T-killâh-celle

## Slide 18
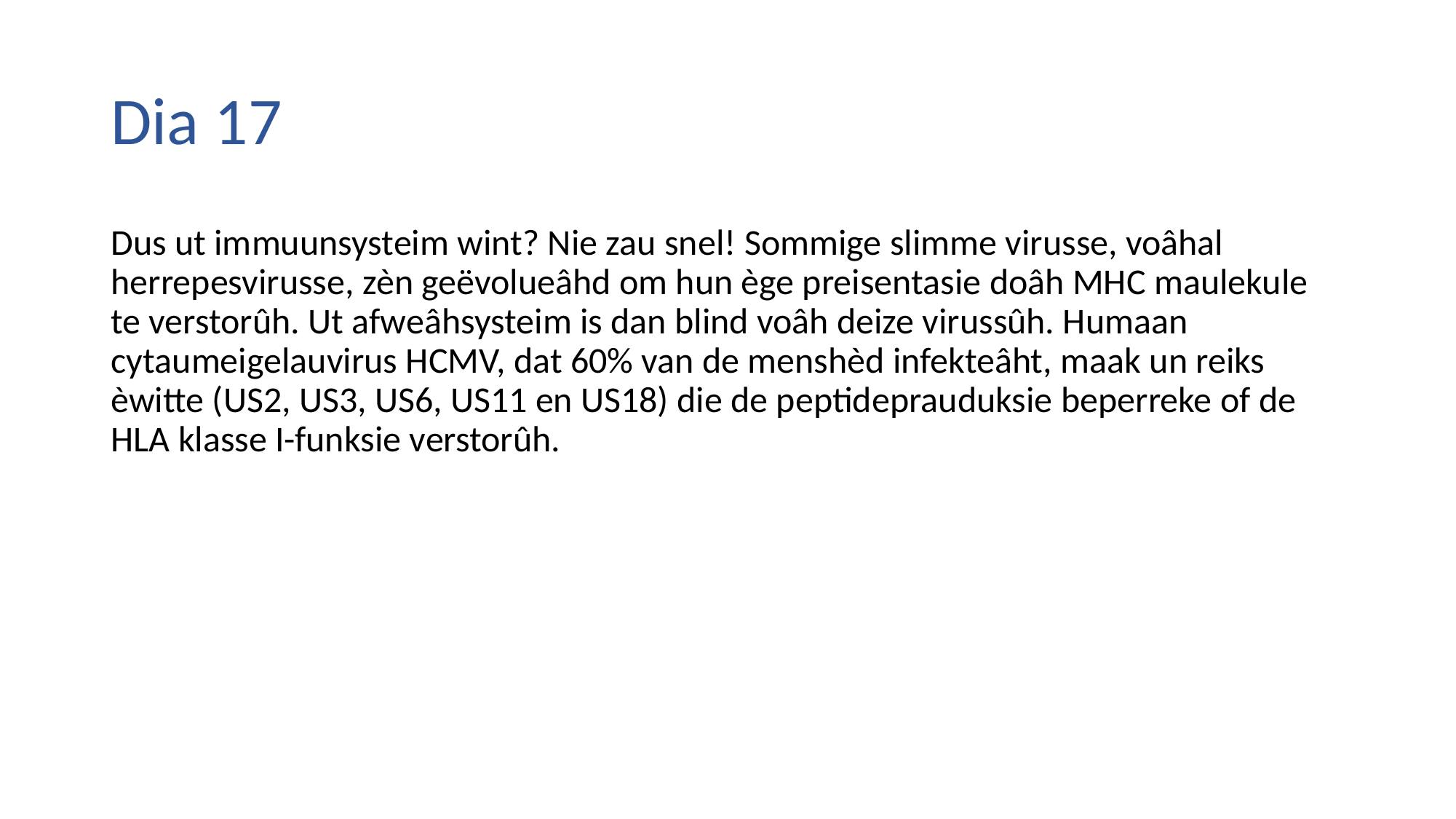

# Dia 17
Dus ut immuunsysteim wint? Nie zau snel! Sommige slimme virusse, voâhal herrepesvirusse, zèn geëvolueâhd om hun ège preisentasie doâh MHC maulekule te verstorûh. Ut afweâhsysteim is dan blind voâh deize virussûh. Humaan cytaumeigelauvirus HCMV, dat 60% van de menshèd infekteâht, maak un reiks èwitte (US2, US3, US6, US11 en US18) die de peptideprauduksie beperreke of de HLA klasse I-funksie verstorûh.

## Slide 19
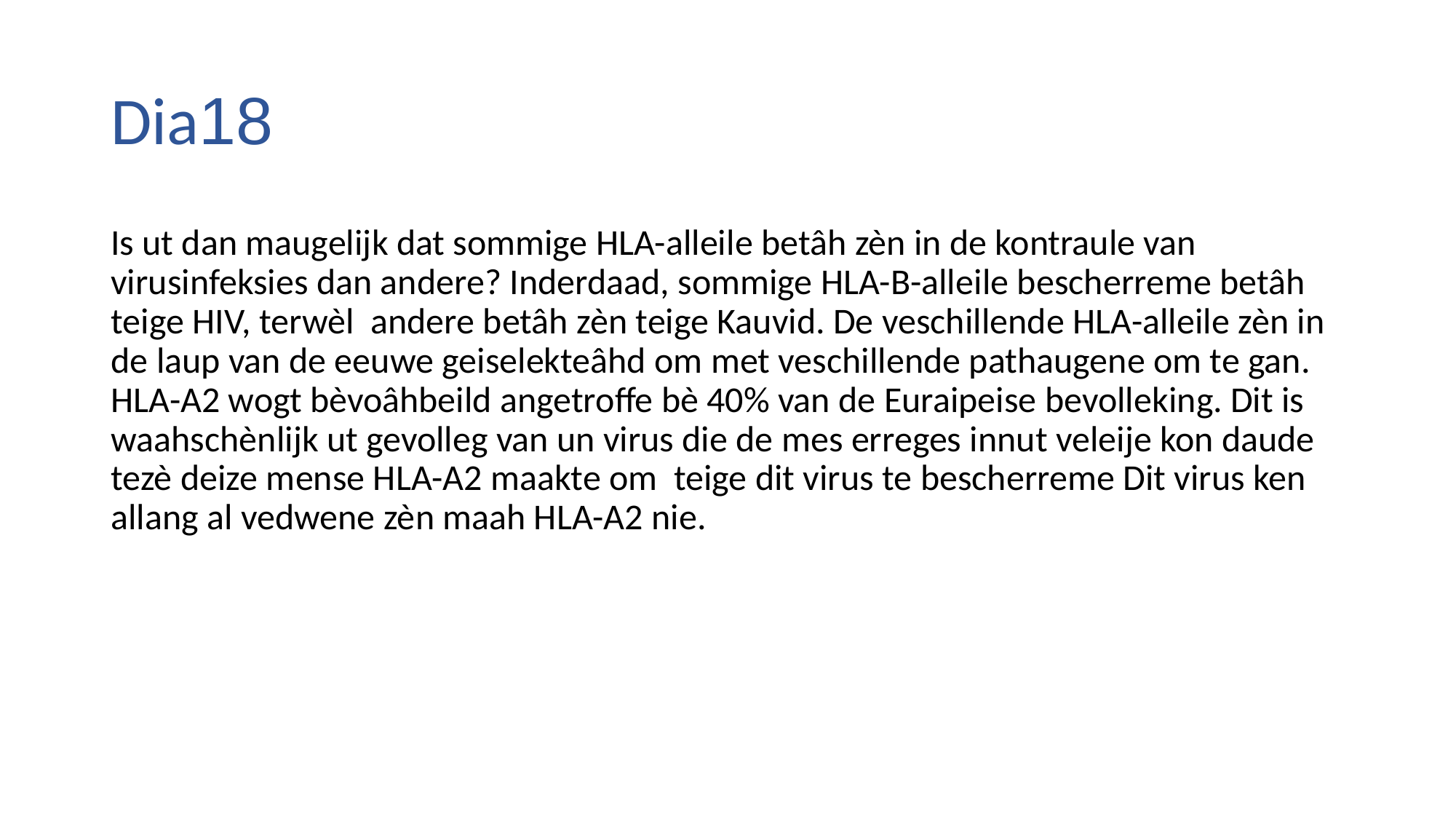

# Dia18
Is ut dan maugelijk dat sommige HLA-alleile betâh zèn in de kontraule van virusinfeksies dan andere? Inderdaad, sommige HLA-B-alleile bescherreme betâh teige HIV, terwèl  andere betâh zèn teige Kauvid. De veschillende HLA-alleile zèn in de laup van de eeuwe geiselekteâhd om met veschillende pathaugene om te gan. HLA-A2 wogt bèvoâhbeild angetroffe bè 40% van de Euraipeise bevolleking. Dit is waahschènlijk ut gevolleg van un virus die de mes erreges innut veleije kon daude tezè deize mense HLA-A2 maakte om  teige dit virus te bescherreme Dit virus ken allang al vedwene zèn maah HLA-A2 nie.

## Slide 20
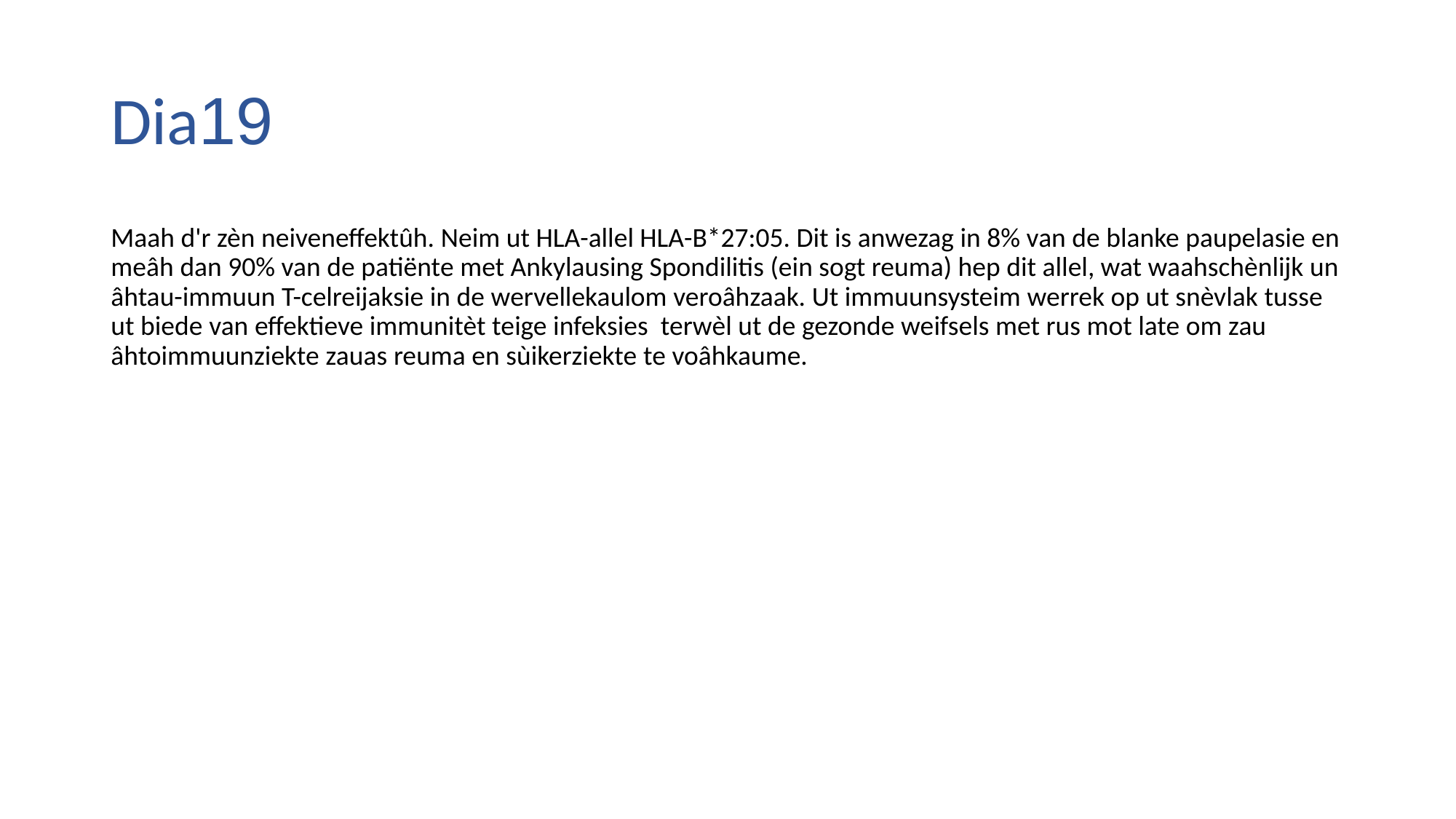

# Dia19
Maah d'r zèn neiveneffektûh. Neim ut HLA-allel HLA-B*27:05. Dit is anwezag in 8% van de blanke paupelasie en meâh dan 90% van de patiënte met Ankylausing Spondilitis (ein sogt reuma) hep dit allel, wat waahschènlijk un âhtau-immuun T-celreijaksie in de wervellekaulom veroâhzaak. Ut immuunsysteim werrek op ut snèvlak tusse ut biede van effektieve immunitèt teige infeksies  terwèl ut de gezonde weifsels met rus mot late om zau âhtoimmuunziekte zauas reuma en sùikerziekte te voâhkaume.

## Slide 21
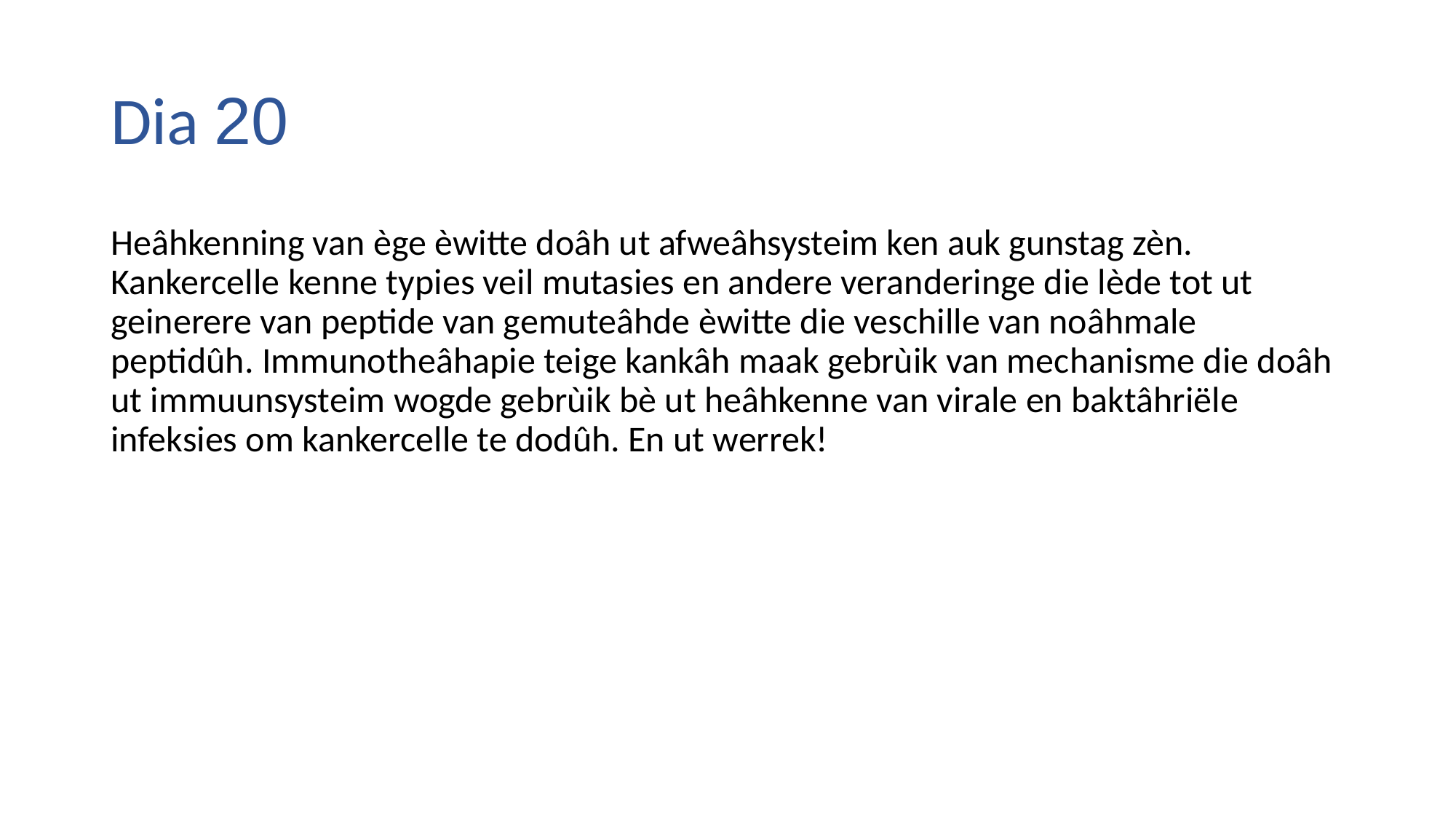

# Dia 20
Heâhkenning van ège èwitte doâh ut afweâhsysteim ken auk gunstag zèn. Kankercelle kenne typies veil mutasies en andere veranderinge die lède tot ut geinerere van peptide van gemuteâhde èwitte die veschille van noâhmale peptidûh. Immunotheâhapie teige kankâh maak gebrùik van mechanisme die doâh ut immuunsysteim wogde gebrùik bè ut heâhkenne van virale en baktâhriële infeksies om kankercelle te dodûh. En ut werrek!

## Slide 22
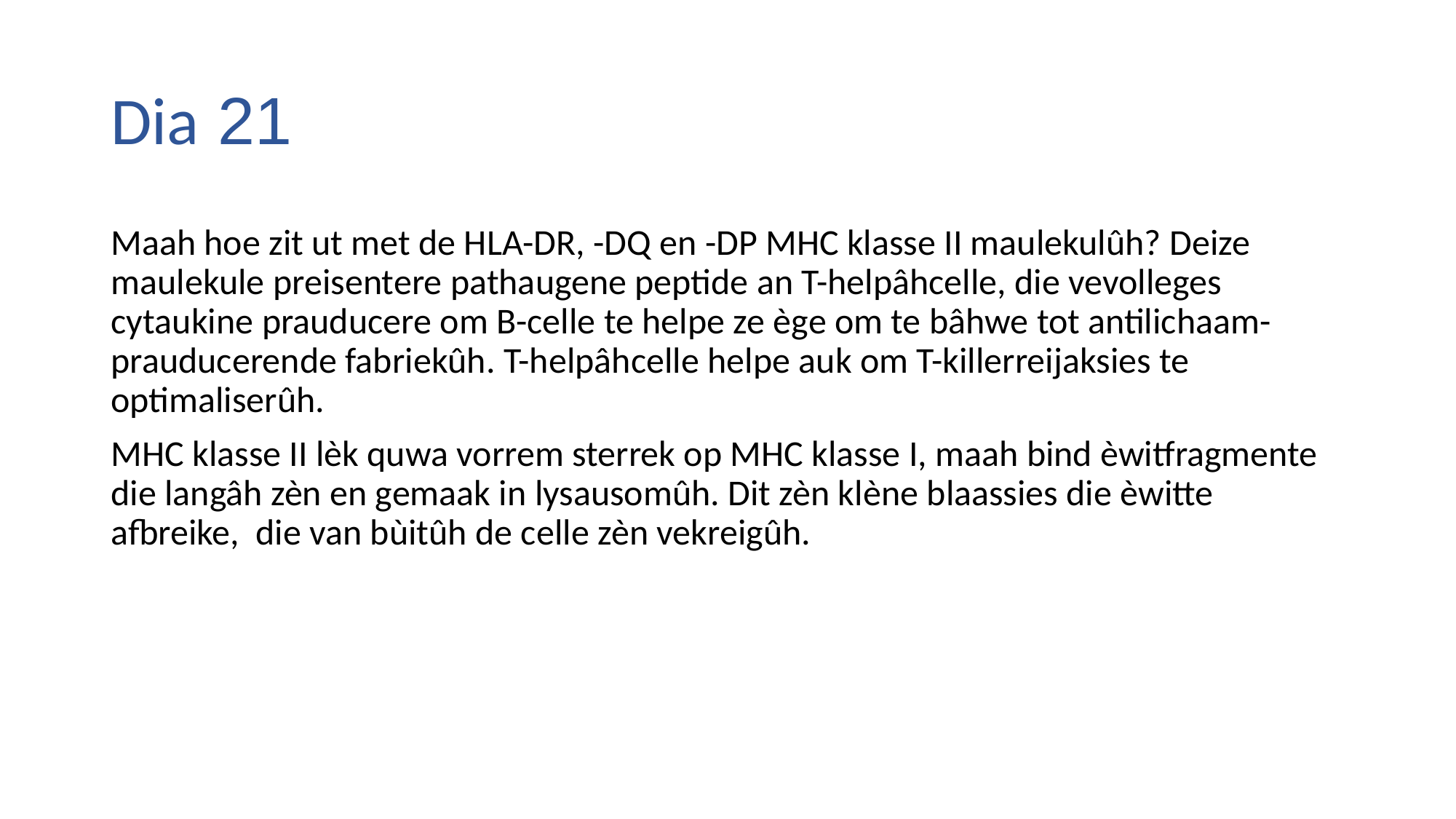

# Dia 21
Maah hoe zit ut met de HLA-DR, -DQ en -DP MHC klasse II maulekulûh? Deize maulekule preisentere pathaugene peptide an T-helpâhcelle, die vevolleges cytaukine prauducere om B-celle te helpe ze ège om te bâhwe tot antilichaam-prauducerende fabriekûh. T-helpâhcelle helpe auk om T-killerreijaksies te optimaliserûh.
MHC klasse II lèk quwa vorrem sterrek op MHC klasse I, maah bind èwitfragmente die langâh zèn en gemaak in lysausomûh. Dit zèn klène blaassies die èwitte afbreike,  die van bùitûh de celle zèn vekreigûh.

## Slide 23
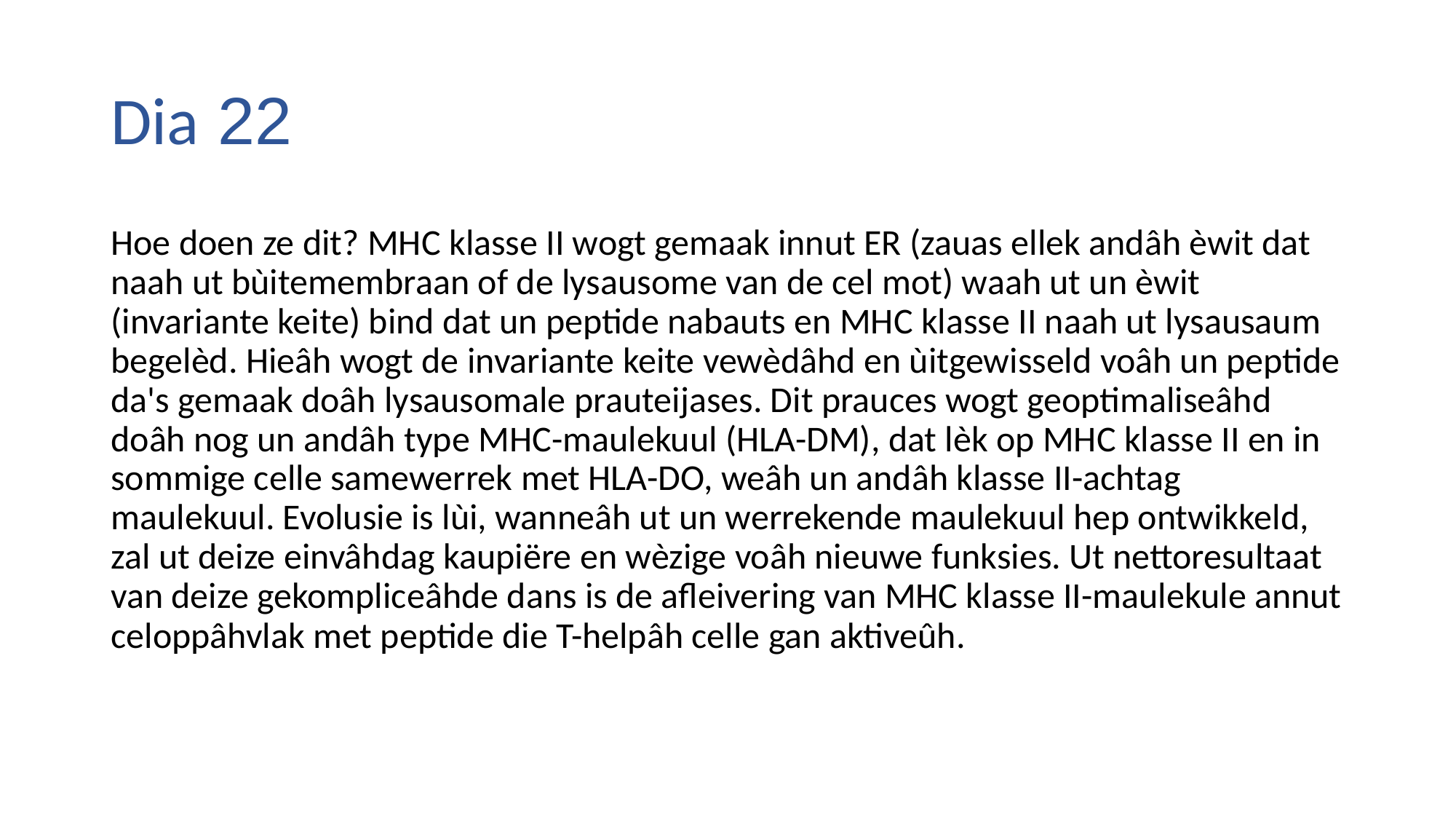

# Dia 22
Hoe doen ze dit? MHC klasse II wogt gemaak innut ER (zauas ellek andâh èwit dat naah ut bùitemembraan of de lysausome van de cel mot) waah ut un èwit (invariante keite) bind dat un peptide nabauts en MHC klasse II naah ut lysausaum begelèd. Hieâh wogt de invariante keite vewèdâhd en ùitgewisseld voâh un peptide da's gemaak doâh lysausomale prauteijases. Dit prauces wogt geoptimaliseâhd doâh nog un andâh type MHC-maulekuul (HLA-DM), dat lèk op MHC klasse II en in sommige celle samewerrek met HLA-DO, weâh un andâh klasse II-achtag maulekuul. Evolusie is lùi, wanneâh ut un werrekende maulekuul hep ontwikkeld, zal ut deize einvâhdag kaupiëre en wèzige voâh nieuwe funksies. Ut nettoresultaat van deize gekompliceâhde dans is de afleivering van MHC klasse II-maulekule annut celoppâhvlak met peptide die T-helpâh celle gan aktiveûh.

## Slide 24
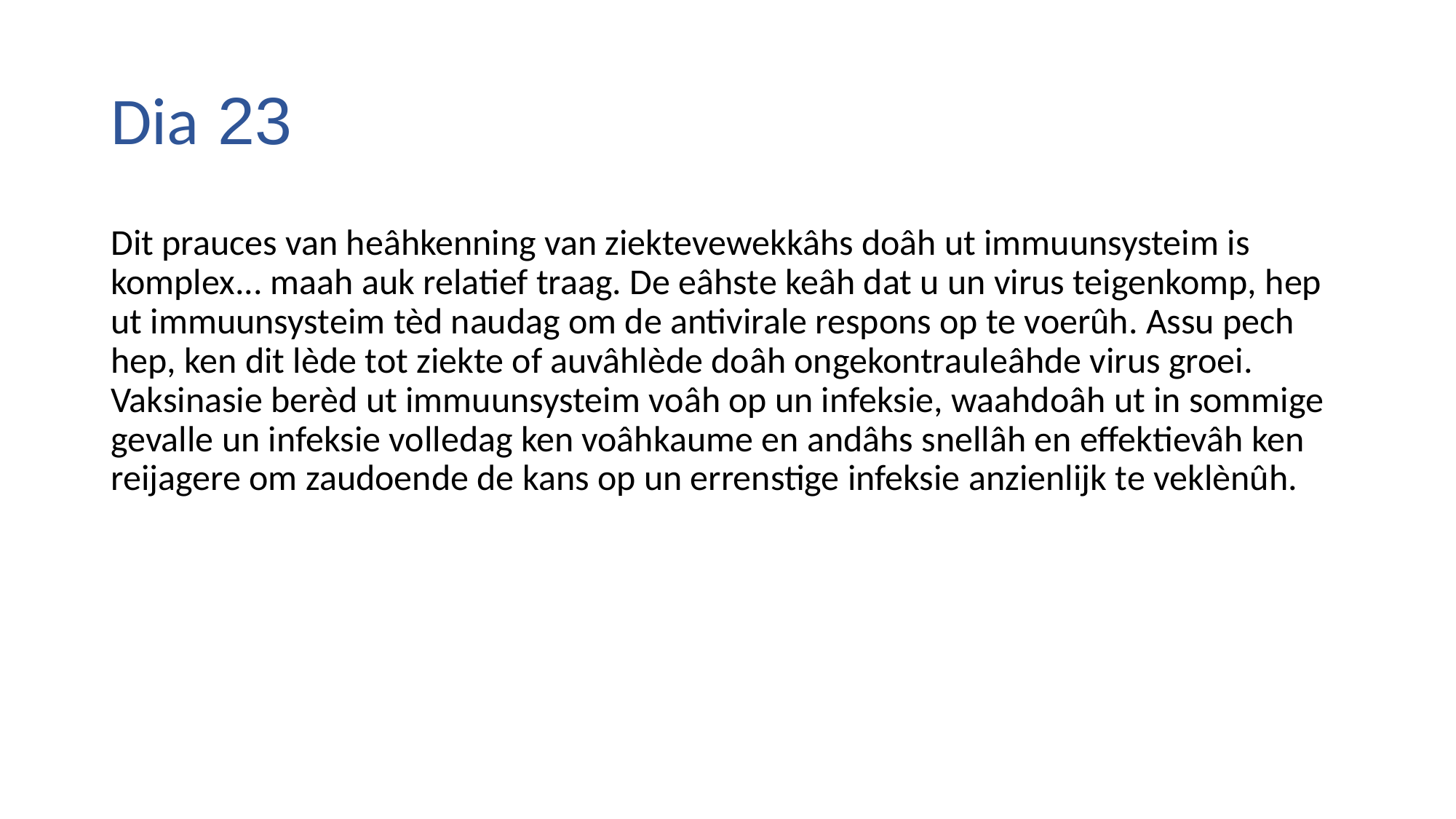

# Dia 23
Dit prauces van heâhkenning van ziektevewekkâhs doâh ut immuunsysteim is komplex... maah auk relatief traag. De eâhste keâh dat u un virus teigenkomp, hep ut immuunsysteim tèd naudag om de antivirale respons op te voerûh. Assu pech hep, ken dit lède tot ziekte of auvâhlède doâh ongekontrauleâhde virus groei. Vaksinasie berèd ut immuunsysteim voâh op un infeksie, waahdoâh ut in sommige gevalle un infeksie volledag ken voâhkaume en andâhs snellâh en effektievâh ken reijagere om zaudoende de kans op un errenstige infeksie anzienlijk te veklènûh.

## Slide 25
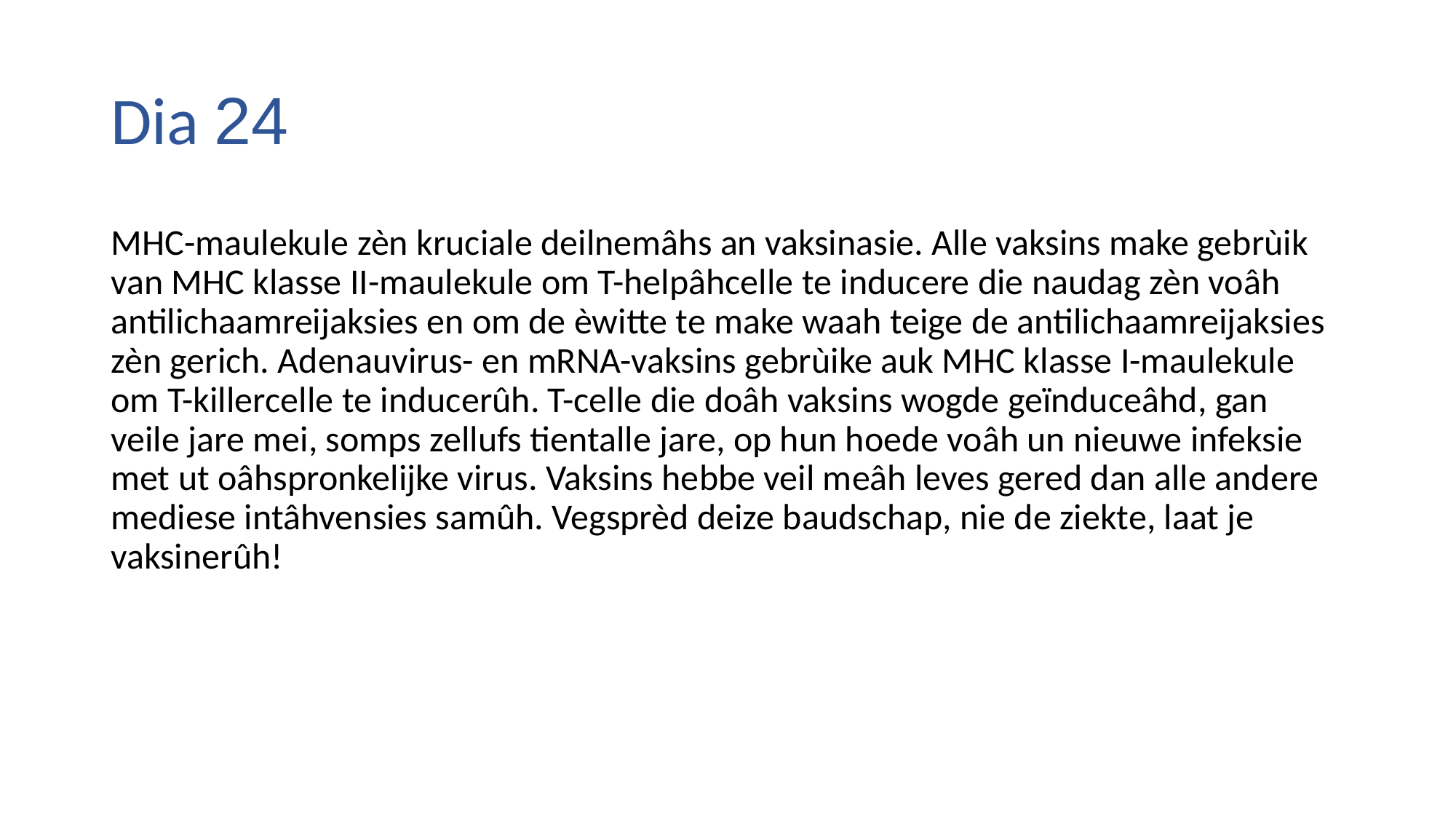

# Dia 24
MHC-maulekule zèn kruciale deilnemâhs an vaksinasie. Alle vaksins make gebrùik van MHC klasse II-maulekule om T-helpâhcelle te inducere die naudag zèn voâh antilichaamreijaksies en om de èwitte te make waah teige de antilichaamreijaksies zèn gerich. Adenauvirus- en mRNA-vaksins gebrùike auk MHC klasse I-maulekule om T-killercelle te inducerûh. T-celle die doâh vaksins wogde geïnduceâhd, gan veile jare mei, somps zellufs tientalle jare, op hun hoede voâh un nieuwe infeksie met ut oâhspronkelijke virus. Vaksins hebbe veil meâh leves gered dan alle andere mediese intâhvensies samûh. Vegsprèd deize baudschap, nie de ziekte, laat je vaksinerûh!

## Slide 26
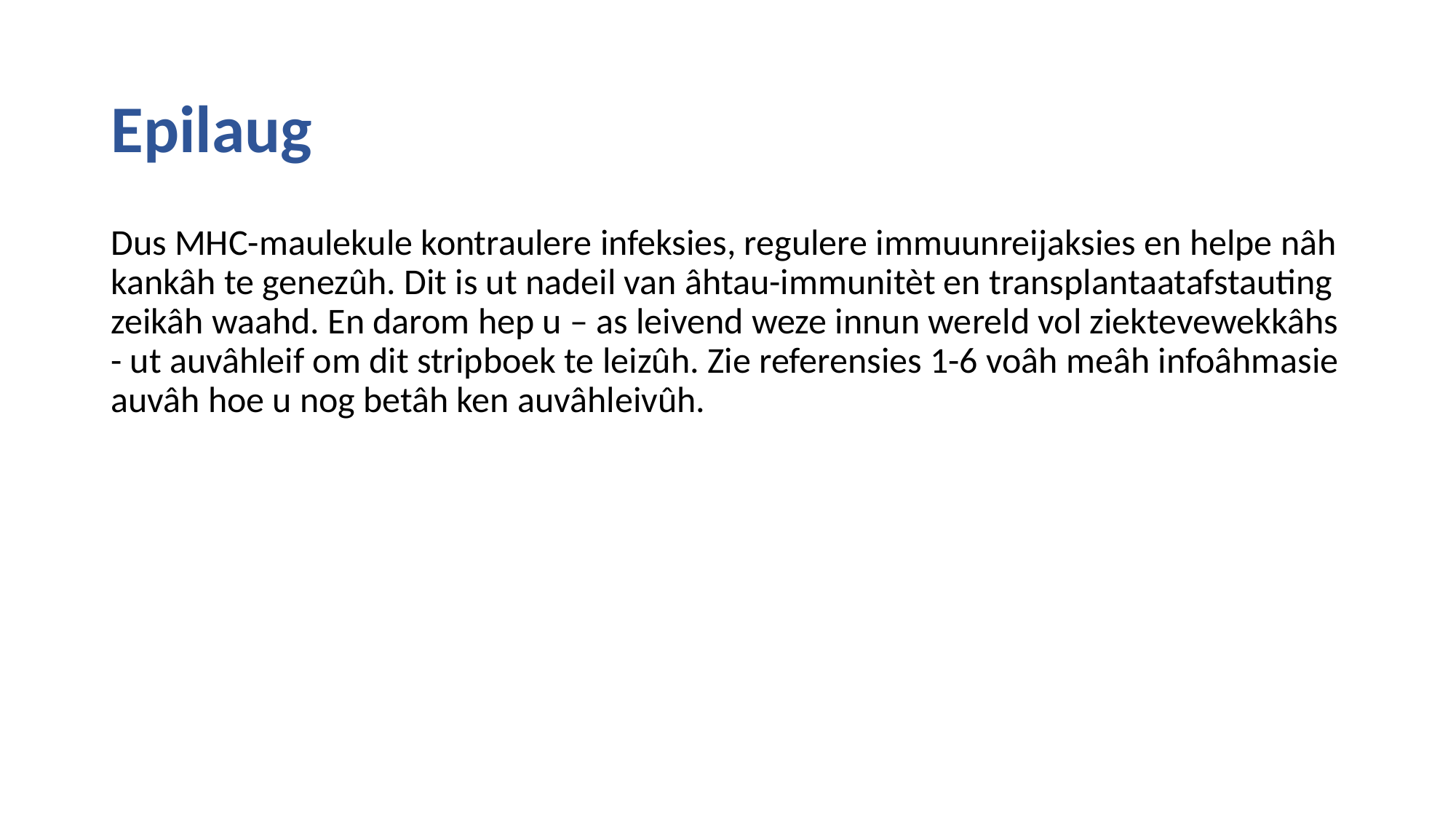

# Epilaug
Dus MHC-maulekule kontraulere infeksies, regulere immuunreijaksies en helpe nâh kankâh te genezûh. Dit is ut nadeil van âhtau-immunitèt en transplantaatafstauting zeikâh waahd. En darom hep u – as leivend weze innun wereld vol ziektevewekkâhs - ut auvâhleif om dit stripboek te leizûh. Zie referensies 1-6 voâh meâh infoâhmasie auvâh hoe u nog betâh ken auvâhleivûh.
