# Supplementary material for: HLA molecules in transplantation, autoimmunity and infection control: A comic book adventure
Source: HLA. 2022 May 15;100(4):301–11. doi: 10.1111/tan.14626 (PMC9545814; doi:10.1111/tan.14626)
Supplement: Supplementary file 1 — Supporting information. [file TAN-100-301-s001.zip › Supplementary files/PP_Hebrew_Admon Arie.1.pdf]

מעורבות מולקולות קומפלקס התאמת הרקמות בהשתלת רקמות, מחלות אוטואימוניות, ומניעת הדבקה פתוגנית: ספר הרפתקאות מצויר

HLA molecules in transplantation, autoimmunity and infection control.
A comic book adventure

by Eric Reits and Jacques Neefjes

Translated by Arie Admon. Original text : ***https://doi.org/10.1111/tan.14626***

Department of Cell and Chemical Biology, ONCODE Institute, Leiden University Medical Centre LUMC, The Netherlands

שקף 1:

לפני 1900 שנה, שני רופאים אחים, קוסמס ודמנוס, ביצעו את השתלת הרקמות הראשונה, ככל שידוע, הם החליפו את רגלו הנגועה בגנגרנה של סוחר, עם רגל שהסירו מהעבד שלו. אומנם, גורלו של העבד אינו ידוע להיסטוריונים, אך סביר שהוא לא התנדב למסור את רגלו בשמחה.

שקף 2:

בזכות השתלת הרקמות "הפלאית" הזו הם הוכרזו כקדושים המברכים את משתילי הרקמות, וזה למרות שהם הוצאו להורג בגלל השתייכותם לנצרות. חטא זה תוקן, כנראה, רק כאשר הגיעו לגן העדן.

שקף 3:

אך מדוע השתלת רקמות כל כך מסובכת? מה הם הלחצים האבולוציוניים המקשים עליה? אפילו דארווין וודאי תהה.. אבל, לצערו הוא לא הכיר משפחה חלבונים חשובה המבוטאים ברוב תאיהם של החולייתנים.

שקף 4:

הבה ונתחיל בתיאור מה שידוע לנו כעת על שתי משפחות חלבונים אלו בגופנו: חלבונים אלו הם הפולימורפיים ביותר מבין כל חלבוני גופנו (כלומר, כל אחד מהחלבונים שונה במידה ניכרת בין אנשים שונים). זאת בשונה ביחס לשאר סוגי החלבונים בגופנו, אשר כמעט זהים בין אנשים שונים. חלבונים אלו נקראים "קומפלקס התאמת הרקמות" ובלועזית HLA class I, שבנוסף נקראים בבני אדם MHC class II ו- MHC class I.

שקף 5:

החשובים בין חלבוני התאמת הרקמות, בבני האדם, הם HLA-A, HLA-B, HLA-C מבין חלבוני MHC class I, וכמו כן, HLA-DR, HLA-DQ, ו- HLA-DP מבין חלבוני MHC class II. החלבונים HLA-A, HLA-B, HLA-C מצויים כמעט בכל תאי הגוף השונים באדם, חוץ מאשר בתאי דם אדומים. לעומת זאת HLA-DR, HLA-DQ, ו- HLA-DP נמצאים בעיקר בתאי מערכת החיסון.

שקף 6:

חלבוני ה HLA הם כה פולימורפים ולכן זרים בין אדם לאדם, עד כי אצל נשים בהריון נוצרים נוגדנים כנגד חלבוני ה- HLA של העובר כאשר הם שונים מאלו של האם. שכמובן מקודדים על ידי העותקים של הגנים שהעובר קיבל מאביו. נוגדנים אלו אפשרו קביעת אבהות של הילוד, וזה עוד לפני שאנליזה גנטית הפכה להיות זמינה. בנוסף, הסרומים האלו של נשים בהריון שימשו גם כדי לבצע התאמת רקמות לצורך השתלה ותרומת מוח עצם. במפגשים מדעיים ורפואיים הוחלפו סרומים כאלו בין מעבדות, והתגובות של הנוגדנים שלהם שימשו כדי לקרוא בשמות ולזהות את הגנים והחלבונים HLA-A, HLA-B, HLA-C ובנוסף, לאפיין את התת-סוגים השונים שלהם. אלו קיבלו שמות ומספרים לפי סדר זיהוים, למשל HLA-A1, ואחריו HLA-A2, וכולי. כך גם זוהו וקיבלו שמות החלבונים HLA-DR, HLA-DQ, ו- HLA-DP. למשל, לאדם מסוים יהיה HLA-A1, B8-, Cw7, DR3, DQ2, ו- DPw4 שאותם קיבל מאמו, ובנוסף HLA-A2, B27-, Cw1-, DR4-, DQ3-, ו- DPw4- שאותם קיבל בתורשה מאביו.

שקף 7:

כיום, קביעת התאמת הרקמות מתבצעת באופן סדיר על ידי ריצוף DNA. קוריוז המעניין לציון, ישנן עדויות לכך שנשים יכולות לזהות גברים עם סוגי HLA השונים משלהן, ושתחושות אלו משפיעות על בחירת בני זוגן.

שקף 8:

למרות שהפולימורפיות הרבה מועילה ליצירת שונות באוכלוסיית בני האדם, היא מקשה מאוד על תרומת אברים ומוח עצם, מכוון שיש צורך להתאים ככל הניתן את ה- HLA השונים של התורם והמושתל. ככל שהתאמה זו אינה מושלמת, גדל הסיכון לדחיית הרקמה המושתלת.

שקף 9:

דארווין בוודאי היה מתפלא. כיצד בחירת בן זוג על ידי ריחו, דחיית רקמה מושתלת, וגילוי מי הוא האב של היילוד, יכולים להיות בעלי ערך אבולוציוני ולעודד את שימור הפולימורפיות הרבה של HLA באוכלוסייה האנושית.

שקף 10:

אבל, ישנו גורם אבולוציוני נוסף וחשוב: ווירוסים ופתוגנים חיידקיים נפוצים מאוד בטבע. ווירוסים כמו קורונה, שפעת, אבולה, ואבעבועות, כולם מדביקים את התאים שלנו ומשתלטים עליהם וגורמים להם לייצר עותקים נוספים של הווירוסים. אפילו הדבקה בווירוס או מיקרואורגניזם אחר, שבדרך כלל לא גורמים למחלה, יכולים להיות קטלנים אצל אנשים שמערכת החיסון שלהם אינה תקינה. לפיכך, נשאלת השאלה, כיצד מערכת החיסון יכולה לזהות ווירוסים שחדרו לתאים ולחסלם לפני שהם מתרבים והורגים אותנו?

שקף 11:

כדי להתגונן מפני פתוגנים, מערכת החיסון פיתחה מנגנוני התגוננות שונים: מאקרופאג'ים בולעים חיידקים ווירוסים ומפרקים אותם, נוטרופילים משחררים חומרים ההורגים חיידקים, תאי B מייצרים נוגדנים המזהים את הפתוגנים, תאי עוזר T מסייעים לתאי ה- B להתפתח ולהתרבות, ותאי קוטל T הורגים את תאי האדם שהודבקו בווירוסים (ואפילו יכולים להרוג תאי סרטן).

שקף 12:

כיצד תא T קטלן יודע את איזה תא עליו להרוג? הרי הווירוסים שמתרבים בתוך התאים לא חשופים לזיהוי על ידי מערכת החיסון, האומנם? אכן כן, כאשר הווירוסים משוכפלים בתוך התאים, שברי חלבונים קצרים מבין חלבוני הווירוסים מועברים אל חלבוני ה- HLA-A, HLA-B, או HLA-C, אשר נושאים אותם אל פני השטח של התאים המודבקים. תאי ה- T הקטלנים מזהים את שברי החלבונים האלו באופן ספציפי כאשר הם קשורים לאחד מבין חלבוני ה-HLA. הגילוי של תופעה זאת, הנקראת רסטריקציה של HLA הייתה כל כך חשובה, עד ששני פרסי נובל ניתנו לחוקרים שגילו את המנגנונים האלו. חשוב לציין שכל אחד מסוגי ה-HLA השונים הקיימים באוכלוסייה, קושרים מגוון שונה של מקטעי חלבון קצרים אלו, הנקראים פפטידים, ומציגים אותם על פני התאים. מגוון ענק זה של פפטידים המוצגים על ידי חלבוני HLA מספקים למערכת החיסון המון מטרות שונות שמשמשות לזיהוי התאים הנגועים ולהורגם.

שקף 13

- כיצד פפטידים, פיסות אלו של חלבוני ווירוסים, נוצרות בכלל בתאים. ובכן, חלק מחלבוני הווירוסים, כמו כל חלבוני התאים, מתפרקים לעיתים, וזה בעזרת מכונות פירוק ננו-מטריות הנקראות פרוטיאזום, שתפקידם הנורמאלי הוא לפרק חלבוני תא מיותרים. בתאים ישנם גם אנזימים נוספים שתפקידם לקצוץ את הפפטידים שנוצרים ולהתאימם לאורך הנחוץ לקשירה ל- HLA. חלק מהפפטידים שנוצרים מפירוק החלבונים נשלחים מהציטופלסמה לתוך הרישתית האנדופלסמטית בתוך התאים, ושם הם נקשרים לחלבוני ה- HLA המחכות להם. לאחר קשירת הפפטידים לחלבוני ה- HLA, אלו נשלחים אל פני התאים כדי לזיהוי על ידי תאי ה- T.

שקף 14:

בואו ונחזור לנושא של פולימורפיות של HLA. כפי שכל אחד יודע מווירוסים, כמו הקורונה והשפעת, הווירוסים מצליחים לעיתים לחמוק מזיהוי על ידי מערכת החיסון, והנוגדנים שנוצרים כנגדם (למשל אלפא, דלתא, אומיקרון, וכו'). כדי להקטין את מגוון האפשרויות שתאי ה- T צריכים להכיר, האללים השונים של ה- MHC (הווריאנטים של הגן) מבטאים חלבונים שונים במידה מסוימת (הנקראים אלוטיפים) שכל אחד מהם מציג רפרטואר שונה של פפטידים. אומנם כל ווריאנט של MHC מציג אלפי פפטידים רבים, אבל בגלל הפולימורפיות של MHC, רפרטוארים שונים של פפטידים מוצגים אצל כל אדם. מכוון שפפטידים שונים של הווירוסים מוצגים על ידי ה- MHC של אנשים שונים, קשה לווירוסים להתאים את עצמם ולהתחמק מהבקרה החיסונית על ידי הימנעות מביטוי של רצפים המעוררים את התגובה. לו היו כל ה MHC זהים בכל בני האדם, סביר שווירוסים היו מצליחים להתאים את עצמם, מדביקים והורגים את כל בני האדם. הפולימורפיות הרבה גורמת לכך שכאשר ווירוסים מצליחים להרוג את קרבנותיהם, אלו רק חלק מהאוכלוסייה, ואותם ששורדים, הם העמידים יותר. תופעה זו מסבירה את היתרון האבולוציוני המוקנה על ידי הפולימורפיות הרבה של MHC.

שקף 15:

אם כך, קורא יקר, המצב קשה אם לצערך אתה זקוק להשתלת רקמה אחת או שתיים. הפולימורפיות הרבה של ה-HLA משפרת את הסיכוי של מין בעלי חי מסוים לשרוד, אך מקשה על סיכויי ההישרדות של פרט הזקוק להשתלת כליה. דחיית רקמה מושתלת היא תוצאה של תגובה חיסונית שמזהה רקמה מושתלת כאילו היא רקמה הנגועה בווירוס, וכלן מגיבה ותוקפת את הרקמה המושתלת וגורמת לדחייתה.

שקף 16:

מסקנה חשובה ועקרונית נוספת: שום מערכת אינה מושלמת, אפילו לא מערכת החיסון! בואו ונחשוב כיצד תאי ה- T הקטלנים מצליחים לזהות את התאים הנגועים בווירוסים מספיק מהר כדי להביא תועלת ולמנוע את המשך תהליך ההדבקה. הווירוסים כמובן משתכפלים מהר מאוד, לעיתים תוך שעות בודדות הם כבר השתכפלו מספיק, כדי להשתחרר מהתאים ולהדביק תאים אחרים. זה מהר מדי מכדי שאפשר יהיה לחכות עד שחלבוני הווירוסים יתפרקו, ייווצרו פפטידים שיקשרו ל- MHC וישלחו לפני השטח של התאים, ויספיקו להפעיל את תאי ה- T כדי לעצור את תהליך ההדבקה. אבל, למזלנו, לא רק מערכת החיסון אינה מושלמת, גם תהליך הייצור של חלבוני הווירוסים אינו חף מתקלות. ואכן, חלק מחלבוני הווירוס החדשים שנוצרים, אינן תקינים לחלוטין, והם מתפרקים בתאים, כמו כל שאר חלבוני התא, וחלק מתוצרי הפירוק האלו (הנקראים DRiPs) מגיעים כפפטידים אל מולקולות ה MHC המחכות להם, נשלחים אל פני התאים ומפעילים את תאי ה- T, וכל זה תוך זמן קצר לאחר ההדבקה הוויראלית. לפיכך, חוסר יעילות, אפילו קל, בסינתזת חלבוני הווירוס, מספיק כדי להקנות למערכת החיסון יתרון במלחמה נגד ווירוסים.

שקף 17:

זהו? מערכת החיסון שלנו מנצחת תמיד? רגע, בואו ולא נהיה שאננים כל כך. ישנם וירוסים רבים, למשל ווירוסים ממשפחת ההרפס, שמשבשים את התהליך של הצגת אנטיגנים על ידי מערכת ה- MHC. לדוגמה, נגיף הציטומגלו (HCMV), בן למשפחה זו, שמדביק כ- 60% מבני האדם. הנגיף מייצר מספר חלבונים (US2, US3, US6, US11, ו- US18) שפוגמים בתהליך הכנת הפפטידים והצגתם על ידיו HLA class I וכך מאפשרים לווירוס לחמוק ממערכת החיסון.

שקף 18:

אם כך, האם ישנם אללים של HLA המקנים עמידות יותר גבוהה כנגד הדבקה וויראלית מסוימת? אכן כן, ישנם אללים של HLA-B המקנים עמידות כנגד HIV, ישנם אחרים המקנים עמידות לווירוס הקורונה. סביר גם כן שבאוכלוסיית בני האדם נוצרה כבר סלקציה של אלו הנושאים אללים המקנים עמידות לווירוסים מסוימים. למשל כ- 40% מאוכלוסיית אירופה נושאת את האלל HLA-A2 שזו הרמה הגבוהה ביותר של הימצאות אלל מסוים באוכלוסייה גדולה שכזאת. סביר מאוד שאלל זה הקנה בעבר הרחוק עמידות כנגד ווירוס מסוים, שיתכן ואפילו אינו קיים יותר, ולכן נפיצות האלל הזה רבה באוכלוסייה.

שקף 19:

מנגד, האם ישנן גם תוצאות לא רצויות לקיומו של אלל מסוים באוכלוסייה. אכן כן, למשל HLA-B*27:05 נמצא אצל כ 90% מהחולים במחלה קשה הנקראת
דלקת חוליות מקשחת שגורמת לדלקת כרונית הפוגעת בחוליות עמוד השדרה. לכן חושבים החוקרים שיתכן והמחלה נגרמת כתוצאה מתגובה חזקה מידי של תאי T כנגד ה- HLA הזה. אפשר לסכם, שמערכת החיסון צריכה לפעול בטווח הצר שבין הגנה כנגד ווירוסים ופתוגנים אחרים, וזאת מבלי לפגוע ברקמות ובתאים הבריאים שלא לצורך.

שקף 20:

תגובה חיסונית כנגד תאים הנמצאים בגוף יכולה גם להועיל. למשל, בתאי סרטן ישנן מוטציות, ונוצרים חלבונים רבים שאינם נורמאליים, שאינם קיימים בתאים בריאים. כתוצאה מכך, נוצרים בתאים גם שברי חלבונים (פפטידים) שמוצגים על ידי ה- HLA, וחלק מאלו שונים מהפפטידים שבתאים בריאים, ולכן יכולים לשמש לצורך פיתוח טיפול חיסוני (אימונותרפי) שמתבסס על הפעלת המערכת החיסון, המיועדת במקור להגנה נגד ווירוסים וחיידקים, וכך לתקוף באופן סלקטיבי את תאי הסרטן.

שקף 21:

ובכן, מה לגבי חלבוני MHC class II, HLA-DR, HLA-DQ, ו- HLA-DP ? מולקולות אלו מציגות גם פפטידים רבים שמקורם בחלבוני הפתוגנים המזוהים על ידי תאי העוזר T. תאים אלו משחררים ציטוקינים משפעלים את תאי ה- B לייצר נוגדנים בכמות גדולה, ובנוסף, מסייעים לתאי T הקטלנים להרוג את התאים המודבקים בווירוסים.

חלבוני MHC class II אומנם דומים במבנה שלהם במידה רבה לחלבוני MHC class I אך מציגים פפטידים ארוכים יותר. אלו נוצרים על ידי פירוק החלבונים בעזרת אנזימים המצויים בליזוזום, ולא על ידי הפרוטיאזום כמו הפפטידים של MHC class I. הליזוזומים הם אברונים תוך תאיים המסוגלים גם לקלוט חלבונים מחוץ לתאים ולפרקם בתוך התאים.

שקף 22:

כיצד מציגים חלבוני MHC class II פפטידים של הפתוגנים? החלבונים של MHC class II נוצרים ברשתית האנדופלזמטית (ER) כמו רוב החלבונים הממברנאלים והליזוזומאלים, ב- ER חלבונים אלו נקשרים לחלבון הנקרא invariant chain שנקשר לחלבון MHC class II בשקע על פניו המיועד לקשירת הפפטידים, וכך מונע את קישרת פפטידים בשלב הזה, חלבון זה גם מוביל את החלבון MHC class II לליזוזום. בליזוזום, שרשרת זו מסולקת, מה שמאפשר לחלבון MHC class II לקשור פפטידים שנוצרו מפירוק חלבוני הפתוגן שפורקו בליזוזום. מי שמסיע לתהליך העמסת הפפטידים בליזוזום הוא חלבון אחר במשפחה הנקרא HLA-DM הדומה ל HLA class II. לעיתים משתתף בתהליך גם HLA-DO שגם הוא משתייך למשפחת חלבוני MHC class II. מסתבר שהאבולוציה היא כנראה עצלה, וכאשר ישנה מערכת תאית הפועלת מספיק טוב, החלבונים האלו מועתקים ומשמשים למטרות דומות תוך שינויים קטנים. התוצאה הסופית של כל התהליך המורכב הזה הוא הצגה של פפטידים שמקורם בפתוגנים, על ידי תאי מערכת החיסון, לתאי העוזר T שמסיעים להפעלת התגובה החיסונית הנחוצה.

שקף 23:

התהליך הזה, של זיהוי פתוגנים על ידי מערכת החיסון, הוא תהליך איטי ומסורבל. בפעם הראשונה שבה אנו נתקלים בפתוגנן, כמו ווירוס למשל, לוקח זמן די רב ליצור את התגובה החיסונית הנחוצה ולהתגבר על המחלה. במידה וזה לוקח זמן רב מידי, יתכן שהמחלה תהיה אפילו קשה, ועלולה לגרום למוות כתוצאה מקצב התרבות גבוה מדי של הווירוסים. חיסון מונע יכול לצמצם מאוד את חומרת המחלה ולמנוע מוות במקרים קיצוניים, וזה על ידי הכנתה מראש של התגובה החיסונית כנגד הפתוגן, שמקטינה מאוד את חומרת המחלה ולעיתים אפילו מונעת הדבקה לחלוטין.

שקף 24:

מסתבר לכן שמולקולות MHC הן חיוניות לתגובה החיסונית. כל החיסונים מסתמכים על התגובה של MHC class II כדי להפעיל את תאי העוזר T, הנחוצים כדי לעורר יצירת נוגדנים המוכוונים כנגד חלבוני המטרה, ומאפשרים השגת תגובה חיסונית. חיסונים, כמו אלו המבוססים על ווירוס האדנו או על מולקולות RNA שליח, משתמשים גם ביצור של חלבוני פתוגן בתוך התאים, וכך מפעילים גם את תהליך הצגת האנטיגנים על MHC class I . בדרך זו הם גורמים להפעלת תאי T קטלנים שהורגים את התאים המודבקים ומונעים המשך התרבות הווירוסים. התרבות ויצירת מאגר של תאי זיכרון T ותאי B שנשמרים לאורך שנים בגוף, מקנה לרוב למחוסן עמידות בפני הדבקה חוזרת באותו פתוגן, עמידות שיכולה להישמר למשך שנים רבות, ואפילו לכל החיים. חיסונים הצילו עד כה את חייהם של יותר אנשים מאשר כל הטיפולים הרפואיים ביחד. אנא, הפיצו את המסר הזה, ובנוסף, אל תפיצו מחלות. התחסנו, ושמרו על חייכם.

לסיכום epilogue

מולקולות ה MHC מגינות מפני מחלות זיהומיות, מווסתות את התגובה החיסונית, וכיום אפילו מסייעות לרפא סרטן. תרומתם מועילה יותר מהנזק היכול להיגרם על ידי תגובה אוטואימונית והדחיה של הרקמות המושתלות. בזכות מולקולות אלו אנו שורדים בעולם מלא בפתוגנים, ואפילו זוכים לקרוא את הספר המצויר הזה. אם ברצונכם בהעשרה נוספת, קראו בבקשה את המאמרים הרשומים מטה.
