# Supplementary material for: HLA molecules in transplantation, autoimmunity and infection control: A comic book adventure
Source: HLA. 2022 May 15;100(4):301–11. doi: 10.1111/tan.14626 (PMC9545814; doi:10.1111/tan.14626)
Supplement: Supplementary file 1 — Supporting information. [file TAN-100-301-s001.zip › Supplementary files/PP_Hindi_Kumar Verma.1.pdf]

एचएलए प्रोटीन का अंग- प्रत्यारोपण, ऑटोइम्यूनिटी और संक्रमण नियंत्रण में महत्वपूर्ण भूमिका ।

अनोखे वैज्ञानिक खोज का एक मनोरंजक चित्रलेख !

HLA molecules in transplantation, autoimmunity and infection control.
A comic Book adventure

by Eric Reits and Jacques Neefjes

Translated by Amit Kumar Verma and Richa Tiwari. Original text : ***https://doi.org/10.1111/tan.14626***

Department of Cell and Chemical Biology, ONCODE Institute, Leiden University Medical Centre LUMC, The Netherlands

स्लाइड-१

लगभग 1900 साल पहले कॉसमॉस और दमनिक नामक दो अरब भाइयों ने, जो कि पेशे से चिकित्सक थे, दुनिया में सबसे पहला अंग-प्रत्यारोपण किया था। इस प्रक्रिया में इन्होंने एक व्यापारी के गैंगरियन बीमारी से ग्रसित पैर को उसके गुलाम के पैर के साथ बदल दिया था। इतिहास के पन्नों में गुलाम का क्या हुआ इसका तो उल्लेख नहीं है, लेकिन ये तो पक्का है की गुलाम ने खुद की इच्छा से अपने पैर का दान तो नहीं ही किया होगा।

स्लाइड-२

यह चमत्कारिक अंग-प्रत्यारोपण आगे चल कर सौंदर्य-वर्धन की नींव बन गई और इन भाइयों को अंग-प्रत्यारोपण का पिता कहा जाने लगा। मगर इसका फल उन भाइयों को यह मिला कि उनका सर काट कर उन्हें जान से मार दिया गया, इसका कारण था क्रिश्चियन धर्म का उल्लंघन। भगवान करे की इन भाइयों के इस नेक काम का स्वर्ग में कुछ तो इनाम मिले अगर स्वर्ग है तो!

स्लाइड-३

किन इवोल्यूशनरी कारण से अंग-प्रतिरोपण इतनी कठिन प्रकिया है, इसकी कल्पना शायद डार्विन ने भी की होगी! मगर उन्हें भी इस अनोखे एचएलऐ प्रोटीन, जो सभी बहूकोशिय जीवों में पाए जाते हैं, के बारे मे कोई जानकारी नहीं रही होगी।

स्लाइड-४

- चलिए हम समझने की कोशिश करते हैं, हमारे शरीर में पाए जाने वाले दो ऐसे प्रोटीन वर्ग की जो कि अंग- प्रत्यारोपण में अहम भूमिका निभाते हैं। इंसानों में इस प्रोटीन वर्ग में सबसे ज्यादा पॉलीमॉरफिस्म (अलग-अलग इंसानों के बीच ये कितना अलग है) देखा गया है। और ये अनूठा हैं कि इसके अलावा बाकि सभी प्रोटीन वर्ग इंसानों के बीच लगभग एक जैसे हैं। ये पोलीमॉर्फिक प्रोटीन अंग- प्रत्यारोपण एंटीजन हैं जो सामान्य तौर पर ऍमएचसी वर्ग-१ और ऍमएचसी वर्ग-२ के नाम से जाने जाते है। मनुष्य प्रजाति में इन्हे एचएलऐ वर्ग-१ और एचएलऐ वर्ग-२ कहा जाता है।

स्लाइड-५

अंग-प्रत्यारोपण के दृष्टिकोण से अगर हम एचएलऐ प्रोटीन का वर्गीकरण करें तो इन्हें दो वर्गों में बाँटा जा सकता है; एचएलऐ वर्ग-१ जिसके अंतर्गत आते हैं एचएलऐ -ऐ, बी और सी और एचएलऐ वर्ग-२ जिसके के अंतर्गत आते हैं एचएलऐ -डीआर, डीसी एवं डीपी। प्रथम वर्ग के एचएलऐ लाल रक्त कोशिकाओं को छोड़ कर बाकी सभी कोशिकाओं में पाए जाते हैं जबकि दूसरे वर्ग के एचएलऐ सिर्फ प्रतिरक्षा तंत्र की कोशिकााओं में पाए जाते हैं। (सेल्स को ही हिंदी में या कोशिका कहा जाता है)

स्लाइड-६

अचंभित करने वाली बात ये है कि एचएलऐ प्रोटीन इतने पॉलिमॉर्फिक हैं कि गर्भवती महिला, अपने बच्चे के एचएलऐ के विरुद्ध भी एंटीबॉडी बनाने लगती है जो कि उसके पिता से आये हैं । इसी खासियत का उपयोग पूराने ज़माने में, जब आनुवंशिक परीक्षण (जेनेटिक टेस्टिंग) की पद्धति उपलब्ध नहीं थी , तब असली पिता का पता लगाने के लिए किया जाता था। गर्भवती महिला के सेरा (रक्त से कोशिकाओं को हटा देने पर बचा रहने वाला तरल पदार्थ जिसमे एंटीबाडी रहती है) का इस्तेमाल टिश्यू (एक तरह के कोशिकाओं के समूह) प्रत्यारोपण में भी किया जाने लगा। विभिन्न वैज्ञानिक संस्थानों में इन सेरा का आदान-प्रदान किया जाने लगा । इन सेरा से होने वाले प्रतिक्रिया के आधार पर ही एचएलऐ प्रोटीन का वर्गीकरण किया गया। और इसी तारिके से एचएलऐ -ए, बी, सी या इसके विभिन्न प्रकारों का पता चला, जिनको आगे चल कर एचएलऐ -ए१ , ए२ और ए३ इत्यादि नाम दिए गए। ऐसा ही नामकरण एचएलऐ -डी , डीपी या डीक्यू प्रोटीन के साथ भी अपनाया गया। उदाहरण के लिए आपके शरीर की कोशिकाओं में एचएलऐ ए१ , बी८ , सीडब्ल्यू७ , डीआर३ , डीक्यू२ या डीपीडब्ल्यू१ आपकी माता से आए हैं और एचएलऐ ऐ२ , बी२७ , सीडब्ल्यू१, डीआर४, डीकिउ३ और डीपिडब्लू४ आपके पिता से आये है।

स्लाइड-७

आजकल तो एचएलऐ टाइपिंग में एक व्यक्ति के एचएलऐ के विभिन्न प्रकारों का पता डीएनऐ के विश्लेषण से हो जाता है। कुछ प्रमाण तो ये भी है की महिलाएँ पुरुषों के एचएलऐ की भिन्नता का पता उनके महक से ही लगा लेती हैं और इस तरह अनुवांशिक तौर पर अलग जोड़े को चुन पाती है।

स्लाइड-८

वैसे तो एचएलऐ की भिन्नता इंसानी विविधता के लिए महत्वपूर्ण हैं लेकिन ये अंगो के लेनदेन में बहुत मुश्किलें भी पैदा करती है, क्योंकि अंग देने और लेने वाले के बीच में एचएलऐ जितना ज्यादा मिले उतनी ही प्रत्यारोपण की प्रक्रिया सफल होना की संभावना रहती है। अगर अंग देने और लेने वाले के बीच में एचएलऐ की भिन्नता ज्यादा हो तो प्रत्यारोपण के दौरान अंग लेने वाले के शरीर की प्रतिरोधक क्षमता कम करने वाली दवाइयों का उपयोग किया जाता है ताकि शरीर नए अंग को अस्वीकार न करे।

स्लाइड-९

एचएलऐ को जान कर तो डार्विन भी हैरान होंगे कि सिर्फ सूंघ कर अपनी जोड़ीदार का पता लगाना, या अंग प्रत्यारोपण के दौरान अंग अस्वीकृति को रोकना, या एचएलऐ टाइपिंग से पिता का पता लगाना, एचएलऐ की उत्पत्ति का सिर्फ ऐवोलुशन सम्बन्धी कारण नहीं हो सकता। !!

स्लाइड-१०

एक और कारण भी है- हमारे आस-पास पाए जाने वाले रोगाणु (वायरस या बैक्टीरिया), कोरोना, इन्फ्लुएंजा, इबोला या अन्य कई वायरस हमारी कोशिकाओं का अपयोग करके फलते-फूलते हैं। ज़रुरी बात यह है कि अगर प्रतिरक्षा तंत्र न हो तो छोटे संक्रमण भी जानलेवा साबित हो सकते हैं। अब हमारे सामने सवाल बहुत आसन सा है की हमारा प्रतिरक्षा तंत्र कैसे हमारे कोशिकाओं के अंदर घुसने वाले वायरस को पहचानता है और उन्हे ख़त्म कर देता है, इससे पहले की वो कोशिकाओं को नुकसान पंहुचा पाए।

स्लाइड-११

यह अदभुत है कि प्रतिरक्षा तंत्र में समय के साथ बदलाव आया और रोगाणुओं का सामना करने के लिए प्रतिरक्षा तंत्र में कई नए हथियारों की उत्पत्ति हुई है । उदाहरण के लिए विशेष प्रकार की कोशिकाओं जैसे कि मैक्रोफेज, बैक्टीरिया या वायरस को खा के ख़त्म कर देता है, वहीं न्यूट्रोफिल्स के नाम से जाने जाने वाली कोशिकाएं बैक्टीरिया या वायरस पर जानेलेवा पदार्थ छोड़ कर उन्हें मार डालती है। बी-सेल्स नाम की कोशिकाएँ एंटीबॉडी बनती है , टी-हेल्पर सेल्स नामक कोशिकाएं बी-सेल्स और अन्य कोशिकाओं की मदद करती है। टी-किलर सेल्स, वायरस से ग्रसित सेल्स को मार डालती है और कैंसर वाले सेल्स को भी मार डालती है।

स्लाइड-१२

सवाल यह है कि हमारे कोशिकाओं को पता कैसा चलता है कि मारना किसे है, क्योकी वायरस तो खुद कोशिकाओं के अंदर छिपा बैठा है, है की नहीं? असल में होता यह है कि जब यह वायरस हमारे शरीर कि कोशिकाओं में खुद की संख्या बढ़ा रहा होता है, तभी इन के प्रोटीन के कुछ बहुत छोटे-छोटे टुकड़े (पेप्टाइड्स), एचएलऐ - ए, बी या सी को मिल जाते हैं। और ये एचएलऐ फिर इन प्रोटीन के टुकड़ो को कोशिकाओं के ऊपरी सतह (जिसे सेल मेम्ब्रेन कहते है) पर प्रदर्शित करता है। अब टी-किलर सेल इस छोटे से टुकड़े को एक विशेष प्रकार के अणु के तौर पर पहचान लेती है। इसे एचएलऐ रेस्ट्रिक्शन (प्रतिबंध) कहा जाता है यह और ये खोज इतनी महत्वपूर्ण हैं की इस पर दो नोबेल पुरस्कार दिए गए हैं । हर अलग प्रकार का एमएचसी वर्ग -१ अलग प्रकार के पेप्टाइड को प्रदर्शित करता है ताकि प्रतिरक्षा तंत्र को रोगाणु से भरे कोशिकाओं को निशाना बना कर मारने में आसानी हो।

स्लाइड-१३

अब आपके मन में ये सवाल होगा की वायरस के प्रोटीन के ये छोटे भाग जिन्हे वायरल पेप्टाइड कहा जाता है बनते कैसे है? एकदम वैसे ही बनते है जैसे कोशिकाओ के अंदर के प्रोटीन के टुकड़े होकर छोटे-छोटे पेप्टाइड्स बनते हैं। सभी कोशिकाओं में प्रोटीन को तोड़ने के के लिए (ताकी उन्हे कोशिकाओ से बहार निकला जा सके) एक ऐसी मशीन होती है जिसे प्रोटीयोसोम कहा जाता है। इस मशीन के द्वारा छोटे-छोटे टुकड़ों में टूटने के बाद कुछ और एंजाइम (और एक तराह के सूछम मशीन) इन छोटे पेप्टाइड्स के किनारे काट कर उन्हे और भी छोटा कर देते हैं, उनमे से कुछ पेप्टाइड्स कोशिकाओं के अंदर साइटोसोल (कोशिकाओं के अंदर रहने वाला तरल पदार्थ) से ई. आर. (एंडोप्लाज्मिक रेटिकुलम, कोशिकाओं के अंदर पाए जाने वाले विभिन्न प्रकार के कार्यकारी विभागों में से एक विभाग) में भेजे जाते हैं। जहाँ वे एचएलऐ से बंध जाते हैं और इ. आर. से बाहर निकल के के ऊपर आ जाते हैं, जहां ये इंतजार करते हैं टी-किलर सेल्स का, ताकि वो उन के पेप्टाइड्स को पहचान के उन्हें मार पाए, है ना मजेदार!

स्लाइड-१४

चलिए वापस आते हैं एचएलऐ के विभिन्नता पर। जैसा की हम सब जाते हैं ज्यादातर वायरस चाहे कोविड-19 हो या इन्फ्लुएंजा, प्रतिरखा तंत्र के एंटीबॉडी से अपने आप को बचाने के लिए अपने आप को बदलने में माहिर है (जिनके बदले भिन्न प्रकारों को हम अल्फा, डेल्टा, ओमाइक्रोन इत्यादि नाम से जानते हैं।) टी-सेल्स इस परेशानी को कम करने के लिए हर एक एमएचसी एलील (अलग-अलग प्रकार के पेप्टाइड समूह) को तैयार करती है। एक ही व्यक्ति के इतने सारे पेप्टाइड्स कोशिकाओ के सतह पर तैनात कर दिए जाते हैं कि प्रतिरक्षा तंत्र से भाग पाना इन वायरस के लिए बहुत ही कठिन हो जाता है। मजे की बात यह है की दो व्यक्ति के बीच में एचएलऐ की भिन्नता इतनी अधिक है की अगर वायरस एक व्यक्ति के एचएलऐ को चकमा दे भी दे तो वो दूसरे व्यक्ति को नहीं दे सकते। सोच कर देखो, अगर हम सब में एक ही प्रकर के एचएलऐ कण पाए जाते तो वायरस जो की एक व्यक्ति के प्रतिरछा तंत्र को धोखा दे गया वो सारी आबादी को ख़त्म करने के लिए काफी था। पर ऐसा ना होने की वजह से यह वायरस केवल कुछ ही लोगो को ही हानी पहुंचा [illegible]

स्लाइड-१५

यदि आपको अंग-प्रत्यारोपण की ज़रूरत है तो एचएलऐ की भिन्नता भले ही आपके लिए बुरी खबर हो मगर यही भिन्नता पूरे मनुष्य आबादी की सुरक्षा के लिए बहुत महत्वपूर्ण है। अंग आदान-प्रदान प्रतिक्रया , या दूसरे के अंग को स्वीकार नहीं करने कि प्रतिक्रया की समस्या इसलिए आती है क्योकि अंग लेने वाला के शरीर का प्रतिरक्षा तंत्र, दुसरे के अंग को वायरस से ग्रसित कोशिकाओं कि तरह समझता है और उस पर आक्रमण कर उसे हटाने कि कोशिश करता हैं , क्योकि उसके लिए दूसरे के अंग कि कोशिकाएं वैसे ही अजनबी हैं जैसे कि वायरस से ग्रसित कोशिकाएं।

स्लाइड-१६

एक छोटा सा पाठ ! इस दुनिया में कुछ भी त्रुटिहीन नहीं है, हमारा प्रतिरक्षा तंत्र भी नहीं। अगर हम उसी दिशा में सोचे तो हमें देखना चाहिए कि किस प्रकार टी-किलर सेल्स वायरस से ग्रसित कोशिकाओं का तुरंत पता लगाती है ताकि उन्हें ख़त्म कर सके। वायरस अपनी संख्या बहुत जल्दी बढ़ा लेते हैं, कई बार तो सिर्फ कुछ घंटों मे ही। इस्से ये समझ में आता है कि वाइरस के प्रोटीन के टूटने का इंतजार करना, उनके जीवन काल ख़त्म होने तक एक धीमी प्रक्रिया है। पर प्रतिरक्षा तंत्र की ही तरह प्रोटीन बनाए की प्रक्रिया साथ ही साथ वायरस के प्रोटीन बनने की प्रकिया भी दोशराहित नहीं है। इन त्रुटिपूर्ण प्रोटीन्स को ड्रिप्स कहा जाता है, ये तुरंत ही टुकड़े-टुकड़े में तोड़ दिए जाए है, और इस प्रकार जोड़ कर देखे तो वायरस के आक्रमण से एंटीजन के प्रदर्शित होने तक सब कुछ टी-किलर सेल्स की प्रतिरक्षा निगरानी के कारण ही होता है।

स्लाइड-१७

सावधान प्रतिरक्षा तंत्र! तुम इतने भी शातिर नहीं! कुछ चालक वायरस खासकर हर्पीज वायरस, अपना आप को इस तरह से बना कर रखें हैं कि वे एंटीजन की प्रस्तुति में बाधा डाल सकते हैं। मानव साइटोमेगालो वायरस, एचसीएमवी जो की लगभाग 60 प्रतिशत इंसानों को संक्रमित करता है, एक प्रकार के प्रोटीन (यूएस २ , यूएस ३ , यूएस ६ , यूएस ११ और यूएस ८) बनाता है जो की पेप्टाइड बनाने कि प्रक्रिया को, एचएलए वर्ग-१ के द्वारा एंटीजन की प्रस्तुति में छेड़खानी करते हैं।

स्लाइड-१८

तो क्या हम ये मान सकते हैं कि कुछ एचएलऐ एलील्स बाकियों की तुलना में बेहतर वायरल संक्रमण को सम्हाल पाए हैं। हाँ बिलकुल! कुछ एचएलऐ -बी एलील्स,एचआईवी के विरुद्ध बेहतर सुरक्षा प्रदान करते हैं और अन्य कोविड के विरुद्ध। यह कहा जा सकता है की भिन्न-भिन्न एचएलऐ एलील्स का हजारों सालो में अलग-अलग तरह के रोगाणुओं से लड़ने के लिए चुना गया है। उदाहरण के लिए के लिए एचएलऐ ए 2 लगभग 60 प्रतिशत यूरोपीय लोगों में देखा जा सकता है, जो की किसी भी एक समूह में सबसे ज्यादा फैला हुआ एचएलऐ है। ये शायद इस बात का भी नतीजा हो सकता है कि कई वर्षों पहले एचएलऐ ए 2 ने किसी रोगाणु से हमें सुरक्षा प्रदान की होगी और जिसके परिणाम से वो रोगाणु अब संक्रमण नहीं फैला सकता।

स्लाइड-१९

अगर हम अच्छे परिणामों की बात करें तो जैसा की हम एचएलऐ -बी*२७ :०५ को बारे में पता चला है , यह कोकशियस आबादी के ८ % लोगों में पाया जाता है और ९० % से भी अधिक अंकायलोसिस स्पोंडिओलिस से ग्रसित व्यक्तियों में भी इस एलील को देखा जा सकता है, जो की माना जाता है की रीढ़ में एक प्रकार की ऑटोइम्यून प्रतिक्रिया (जिसमें की प्रतिरक्षा तंत्र खुद के कोशिकाओं पे ही प्रतिक्रिया शुरू कर देता हैं उन्हें दुश्मन समझ कर) शुरू करता है। तो हम यह कह सकते हैं की प्रतिरक्षा तंत्र उपयुक्त सुरक्षा प्रदान करने और उस दौरान हमारे अपने कोशिकाओं में नुकसान पहुँचाने में दो धारी तलवार की तरह काम करता है।

स्लाइड-२०

पर मजे की बात ये है की टी-सेल्स के द्वारा उत्पन्न हुई ऑटोइम्यूनिटी के फायदेमंद भी हो सकते हैं। जैसे की कैंसर सेल्स के डीएनए में कुछ परिवर्तन (म्यूटेशंस) हो जाते हैं या अन्य वजहों से ये सेल्स ऐसे पेप्टाइड बनाने लगते हैं जो की सामान्य कोशिकाओं में नहीं पाए जाते हैं, और इसी का उपयोग कैंसर इम्मुनोथेरपी में उठाया जाता है। कैंसर इम्यूनोथेरेापी ने प्रतिरक्षा तंत्र द्वारा वायरस या बैक्टीरिया ग्रासित कोशिकाओ को पहचानने की प्रक्रिया का फायदा उठा कर कैंसर कोशिकाओं को मार पाने में कामयाबी प्राप्त की है।

स्लाइड-२१

हमने एचएलऐ-१ के बारे में इतनी बात की पर एचएलऐ डीआर, डीक्यू या डीपी एचएलऐ वर्ग-२ प्रोटीन का क्या? ये प्रोटीन वास्तव में रोगानुओ के पेप्टाइड्स को टी-सहायक या हेल्पर सेल को प्रदर्शित करते हैं। ये टी-हेल्पर सेल्स इसके बाद ऐसे साइटोकाइन बनाकर छोड़ती है जो की बी-सेल्स को एंटीबॉडी पैदा करने वाली कोशिकाओं में बदलने में मदद करती है। इसके अलावा टी-हेल्पर सेल्स टी-किलर सेल्स के काम को भी बेहतर बनाती है। ऍमएचसी वर्ग-२ देखने में ऍमएचसी वर्ग-१ की तरह होते हैं लेकिन वो ऐसे पेप्टाइड को प्रदर्शित करते हैं जो कि ऍमएचसी वर्ग-१ की तुलना में लंबा होता है और लाइसोसोम (कोशिकाओं के अंदर पाए जाने वाले विभिन्न प्रकार के कार्यकारी विभागों में से एक विभाग जहाँ पर बाहर से लाए गए प्रोटीन को तोड़ा जाता है) में बनाया जाता है ।

स्लाइड-२२

आप ज़रूर जानना चाहेंगे कि ये ये सब होता कैसे है? ऍमएचसी वर्ग-२ प्रोटीन्स ई. आर. द्वारा बनाये जाते हैं। यह सामान्य तौर पर हर उन प्रोटीन के साथ होता है जो कि कोशिकाओं के ऊपरी सतह (जिससे मेम्ब्रेन कहा जाता है) पर पाए जाते हैं या फिर लाइसोसोम में ले जाए जाते हैं। ई. आर. में ये प्रोटीन एक दूसरे प्रोटीन (इन्वैरिएंट चैन) के साथ जुड़ जाता है जो की एक पेप्टाइड का एक नमूना होता है। और ये ऍमएचसी अणु को लाइसोसोम तक लेकर जाता है। लाइसोसोम में यह पेप्टाइड का नमूना को ऍमएचसी से अलग किया जाता है। और इसके बदले एक ऐसे पेप्टाइड से जोड़ दिया जाता है जिसे लाइसोसोम के एक एंजाइम द्वारा बनाया गया है। इस प्रकिया का संचालन एचएलए-डीएम या कुछ कोशिकाओं में एचएलऐ-डीओ (एचएलए-डीएम और डीओ दोनों एक तरह के एचएलऐ वर्ग-२ प्रोटीन हैं) करते हैं। जब विभिन्न तरीकों के प्रोटीन से विभिन्न तरीकों का काम करने की बात हो तो इवोल्यूशन ने काफी आलस भरा काम किया है, बस नकल कर के प्रोटीन को बदल दिया है। इस पूरे जटिल नृत्य का नतीजा यह है कि ऍमएचसी वर्ग-२ प्रोटीन पेप्टाइड के साथ बांध कर कोशिकाओं के सतह पर ले जाता है जो कि टी-हेल्पर सेल्स को सक्रिय करता है।

स्लाइड-२३

यह सारी प्रक्रिया जिसके द्वारा रोगाणुओं को प्रतिरक्षा तंत्र में पहचाना जाता है बहुत उलझी हुई तो है ही, साथ ही साथ काफी धीमी भी है। पहली बार जब हम वायरस का सामना करते है, हमारा प्रतिरक्षा तंत्र उसके विरुद्ध तीव्र गति से काम करने के पहले कुछ समय लेता है। अगर आप बदनसीब हुए तो यह देरी आपके लिए जानलेवा हो सकती है या फिर बीमारी में भी बदल सकती है क्योंकि हमारा प्रतिरक्षा तंत्र वायरस के प्रजनन को नहीं रोक पाया और उनसे लड़ाई में हार गए। टीकाकरण प्रतिरक्षा तंत्र को किसी भी प्रकार के संक्रमण से लड़ने के लिए तैयार करके रखता है। और इस प्रकार टीकाकरण की सहायता से हमारा प्रतिरक्षा तंत्र संक्रमण को रोक पाता है या फिर तेज़ी से उसके खिलाफ लड़ने के लिये तैयार हो पाता है।

स्लाइड-२४

ऍमएचसी प्रोटीन्स टीकाकरण में मुख्य भूमिका निभाते हैं। सभी प्रकार के टीके इस बात का फायदा उठा कर बनाये जाते है की किस प्रकार ऍमएचसी वर्ग-२ प्रोटीन, टी-हेल्पर सेल्स को प्रेरित करते है। एडेनोवाइरस और ऍमआरएनए वाले टीके ऍमएचसी वर्ग -१ का भी इस्तेमाल कर के टी-किलर सेल्स को प्रेरित करते करते हैं। टी-सेल्स जिन्हें वैक्सीन के साथ प्रेरित किया गया हो वो सालों या कभी कभी दशकों तक तैनात रहते है उसी वाइरस से लड़ने की तैयारी में। कुल मिलकर टीकाकारण की प्रक्रिया ने हमें इतनी सुरक्षा प्रदान की है और इतने लोग की जान बचाई है जितना की आज तक सभी स्वास्थ्य उपलब्धियों ने मिला कर भी नहीं की । इसलिए कृपया कर इस सूचना को फैलायें, ग़लतफ़हमी या संक्रमण नहीं।

स्लाइड-२५

अंत में हम यह कह सकते हैं कि ऍमएच्चसी प्रोटीन्स हमारे प्रतिरक्षा तंत्र के साथ साथ संक्रमण को नियंत्रित करते हैं, और वर्तमान मे कैंसर के इलाज मे भी सहायता करते हैं। इसी वजह से रोगाणुओं से भरी हुई दुनिया मे रहने के बावजूद भी आप स्वस्थ हैं और इस अनोखे चित्रलेख का आनंद ले पा रहे हैं। यह जानने की लिए की हम और बेहतर कैसे जियें हमारे सन्दर्भ १-६ को देखना न भूलें।
