# Supplementary material for: HLA molecules in transplantation, autoimmunity and infection control: A comic book adventure
Source: HLA. 2022 May 15;100(4):301–11. doi: 10.1111/tan.14626 (PMC9545814; doi:10.1111/tan.14626)
Supplement: Supplementary file 1 — Supporting information. [file TAN-100-301-s001.zip › Supplementary files/PP_Italian_D'Amico_1.pdf]

Il ruolo delle molecole HLA nel trapianto, autoimmunità e controllo delle infezioni.

Un’avventura a fumetti

di Eric Reits e Jacques Neefjes

Translated by Francesca D’Amico. Original text : ***https://doi.org/10.1111/tan.14626***

Department of Cell and Chemical Biology, ONCODE Institute, Leiden University Medical Centre LUMC, The Netherlands

Diapositiva 1

Circa 1900 anni fa, due fratelli medici arabi Cosma e Damiano realizzarono il primo trapianto conosciuto nella storia, sostituendo la gamba in gangrena di un mercante con quella del suo schiavo. Le sorti dello Schiavo non sono note, ma probabilmente non si trattò di una donazione volontaria.

Diapositiva 2

Questo trapianto “miracoloso’’ contribuì alla loro beatificazione, fino a renderli Santi Patroni dei trapianti. Non rappresentò un problema il fatto di essere decapitati per via della loro fede cristiana, che fu presumibilmente corretta dopo la loro ascesa in Cielo.

Diapositiva 3

Perché il trapianto è così difficile, quali sono i fattori evolutivi? Persino Darwin deve esserselo domandato… ma non era a conoscenza di una particolare classe di proteine espresse da quasi tutti gli organismi multicellulari eucarioti.

Diapositiva 4

Iniziamo con il considerare quanto attualmente noto su due particolari classi di proteine presenti nel nostro corpo; quelle con il pi*ù* alto grado di polimorfismo (differenze tra individui). Queste proteine sono uniche dato che quasi tutte le altre nell’uomo sono praticamente identiche. Queste proteine polimorfiche sono gli ‘’antigeni del trapianto’’ e sono generalmente indicate come molecole MHC di classe I e classe II. Negli esseri umani sono chiamate HLA di classe I e II.

Diapositiva 5

Le pi*ù* importanti molecole HLA per i trapianti sono chiamate HLA-A, HLA-B e HLA-C per i geni MHC di classe I e HLA-DR, HLA-DQ e HLA-DP per i geni MHC di classe II. Le HLA-A,-B e -C sono virtualmente presenti in tutte le nostre cellule (ad eccezione dei globuli rossi), mentre le HLA-DR, HLA-DQ e HLA-DP si trovano esclusivamente nelle cellule immunitarie.

Diapositiva 6

Le molecole HLA sono così polimorfiche che le donne incinte spesso producono anticorpi per i diversi tipi di HLA del padre del feto. Questo fenomeno è stato utilizzato per determinare il padre del feto quando i test genetici non erano ancora disponibili. Inoltre, il siero delle donne incinte veniva anche utilizzato per il trapianto di tessuti. Nel corso di una serie di studi, il siero delle donne incinte veniva scambiato tra laboratori e le diverse risposte catalogate. In questo modo furono identificate HLA-A, -B e -C, insieme ad altre forme delle stesse. Queste furono semplicemente numerate HLA-A1, seguita da HLA-A2 ecc. Lo stesso successe per le molecole HLA-DR, -DQ, e -DP. Quindi, i tuoi tessuti potrebbero esprimere (per esempio) proteine HLA-A1, -B8, -Cw7, -DR3, -DQ2, e DPw1 derivanti da tua madre e proteine HLA-A2, -B27, -Cw1, -DR4, -DQ3, e DPw4 derivanti da tuo padre.

Diapositiva 7

Oggi, la determinazione delle HLA è regolarmente realizzata mediante DNA analisi. É stato riscontrato che le donne sono in grado di avvertire diversi tipi di HLA negli uomini tramite l'olfatto, e che questo contribuisce alla selezione genetica di determinati compagni.

Diapositiva 8

Il polimorfismo delle HLA potrebbe contribuire alla diversificazione dell’uomo, allo stesso tempo rappresenta un enorme svantaggio per il trapianto di organi, il quale richiede compatibilità, quanto pi*ù* possibile, tra i tipi di HLA del ricevente e del donatore. In assenza del match perfetto, farmaci immunosoppressivi efficaci sono usati per prevenire il rigetto d’organo.

Diapositiva 9

Darwin si sentirebbe confuso. Di sicuro, captare con l’ olfatto il tuo compagno perfetto, impedire il trapianto di tessuti, o conoscere il vero padre non possono essere i principali motivi evolutivi per cui esiste il polimorfismo delle HLA.

Diapositiva 10

C’è un altro fattore in gioco. Virus e altri microbi patogeni sono abbondanti in natura. Corona, influenza, Ebola, Vaiolo e molti altri virus sfruttano le nostre cellule per creare le proprie famiglie. Anche un’infezione auto-limitante potrebbe essere letale in assenza di un sistema immunitario. E la domanda che sorge è semplice: come fa il sistema immunitario a captare la presenza di virus in agguato nelle nostre cellule e a ucciderli prima che loro uccidano noi?

Diapositiva 11

Per limitare il danno provocato dai virus, il nostro sistema immunitario ha sviluppato diverse armi di difesa. I macrofagi divorano batteri e virus, i neutrofili rilasciano sostanze che uccidono I batteri, i linfociti B producono anticorpi, le cellule T-helper distruggono cellule infettate da virus (e addirittura cellule cancerogene).

Diapositiva 12

Ma come fa una cellula T-killer a sapere chi uccidere? Il virus, localizzato all'interno della cellula, non può essere rilevato, oppure si? In realtà, quando il virus si replica, piccole parti delle sue proteine sono presentate alle molecole HLA-A, -B, o -C che le espongono sulla superficie cellulare. Le cellule T-killer riconoscono questi piccoli frammenti nel contesto di un solo specifico tipo di HLA. La scoperta di questo fenomeno, chiamato restrizione HLA, fu talmente importante da aggiudicarsi due premi Nobel. Ogni tipo diverso di MHC di classe I presenta un diverso repertorio di peptidi per fornire al sistema immunitario vari target e uccidere le cellule che li producono.

Diapositiva 13

Ma come fa prima di tutto un frammento virale ad essere generato? Le proteine virali- così come ogni altra proteina cellulare- sono degradate. Le proteine sono frammentate da una efficiente nano-macchina chiamata proteasoma che è praticamente un sistema di smaltimento rifiuti per proteine. Altri enzimi cellulari tagliano le estremità dei frammenti in peptidi pi*ù* piccoli, alcuni dei quali sono trasportati dal citosol al RE dove possono legarsi alle molecole HLA. Una volta che le HLA sono legate al peptide, questo lascia il RE per raggiungere la superficie cellulare dove *può* essere rilevato dalle cellule T-killer.

Diapositiva 14

Ritorniamo al polimorfismo delle HLA. Come ben noto dal COVID-19 o l'influenza, i virus sono molto bravi a mutare per sfuggire all'azione degli anticorpi (pensa alle varianti alpha, delta, omicron). Per minimizzare questa possibilità per le cellule T, ognuno dei diversi alleli (varianti geniche) dei geni MHC presenta un diverso set di peptidi. Così tanti peptidi sono presentati in una sola persona, che l'evasione virale diventa difficoltosa. La differenza nei tipi di HLA nelle persone implica che anche se ciò accadesse, il virus evasore non riuscirebbe con successo nella prossima persona. Se fossimo stati tutti HLA-identici, un virus evasore ucciderebbe l'intera popolazione, invece in questo caso ucciderà "solo" un paio di individui con HLA incapaci di presentare peptidi virali al sistema immunitario. Il polimorfismo delle HLA quindi protegge la popolazione, il singolo individuo è meno importante. Ciò fornisce una spiegazione completa per l'evoluzione del polimorfismo delle MHC.

Diapositiva 15

Ma ahimè, brutte notizie, caro lettore nel caso in cui dovessi aver bisogno di uno o due organi. Il polimorfismo delle HLA promuove la sopravvivenza della popolazione di una specie, non quella di un individuo con una malattia renale. Il rigetto di trapianto è la conseguenza del sistema immunitario che confonde un organo donato con un organo infettato da un virus e risponde in maniera appropriata attaccando l'organo, il che risulta nel rigetto di trapianto.

Diapositiva 16

Un’importante lezione generale: nulla, incluso il sistema immunitario è perfetto! Parlando di ciò, pensiamo a come le cellule T-killer riescono a riconoscere cellule infettate da virus abbastanza velocemente da far sì che ciò sia utile. I virus possono produrre prole molto velocemente, in alcuni casi in appena qualche ora. Il processo è troppo lento per far sì che le proteine virali vengano degradate alla fine del loro ciclo vitale. Ma proprio come il sistema immunitario stesso, la sintesi di proteine, incluse le proteine virali, è tutt’altro che perfetta. Queste proteine imperfette, chiamate DriPs, sono degradate immediatamente, associando l’inizio dell’infezione virale alla presentazione dell’antigene e permettendo l’immunosorveglianza delle cellule T-killer effettive.

Diapositiva 17

Scacco matto, sistema immunitario? Non così velocemente! Alcuni virus particolarmente intelligenti, soprattutto gli herpesvirus, si sono evoluti al fine di interferire con la presentazione dell’antigene. Il citomegalovirus umano HCMV, il quale infetta il 60% dell’umanità, produce una suite di proteine (US2, US3, US6, US11, e US18) che limitano la produzione di peptidi o interferiscono con le funzioni delle HLA di classe I.

Diapositiva 18

É dunque possibile che alcuni alleli delle HLA sono migliori di altri nell'interfacciarsi con le infezioni virali? Infatti, alcuni alleli HLA-B conferiscono maggiore protezione nei confronti dell'HIV, altri sono migliori per combattere il Covid. I diversi alleli HLA sono stati selezionati nel corso di eoni per fronteggiare patogeni differenti. Per esempio, le HLA-A2 sono riscontrate nel 40% della popolazione europea, la più alta prevalenza di un allele HLA per un dato gruppo. Ciò risulta probabilmente dall'abilità delle HLA-A2 di conferire protezione contro un patogeno con cui si è stato precedentemente a contatto, che potrebbe non essere più una delle principali cause di malattie umane.

Diapositiva 19

Ma ci sono conseguenze collaterali. Consideriamo l'allele HLA-B*27:05. È presente nell'8% della popolazione Caucasica, oltre il 90% dei pazienti affetti da *Spondilite Anchilosante* ha questo allele, che probabilmente innesca una reazione autoimmune mediata dalle cellule T nella colonna vertebrale. Il sistema immunitario si trova sul filo del rasoio nel dover fornire un'immunità efficace senza danneggiare i propri tessuti.

Diapositiva 20

Le cellule T autoimmuni possono anche essere benefiche. Le cellule cancerogene tipicamente possiedono numerose mutazioni e altre alterazioni che conducono alla generazione di peptidi che differiscono dai normali peptidi cellulari. L'immunoterapia anti-cancro sfrutta meccanismi utilizzati dal sistema immunitario per riconoscere le infezioni virali e batteriche al fine di uccidere le cellule cancerogene.

Diapositiva 21

E per quanto riguarda le HLA-DR, -DQ e -DP delle molecole MHC di classe II? Queste molecole presentano peptidi patogeni alle cellule T-helper, che poi producono citochine per aiutare le cellule B a differenziarsi per la produzione di anticorpi. Le cellule T-helper aiutano anche a ottimizzare la risposta delle cellule T-killer. Le MHC di classe II hanno una forma molto simile alle MHC di classe I ma presentano frammenti proteici più lunghi e prodotti nei lisosomi, che sono piccoli organelli che degradano le proteine acquisite dall'esterno delle cellule.

Diapositiva 22

Come fanno a far ciò? Le MHC di classe II sono prodotte nel RE (come qualsiasi altra proteina destinata alla membrana esterna della cellula o ai lisosomi) dove si legano a una proteina (catena invariante) che mima un peptide e conduce l’MHC di classe II al lisosoma. Qui, la catena invariante viene rimossa e scambiata con un peptide creato da enzimi lisosomiali. Questo processo è ottimizzato da un altro tipo di molecola MHC (HLA-DM, che somiglia alle MHC di classe II, e in alcune cellule funziona in concerto con HLA-DO, un'altra molecola simile alla classe II. L'evoluzione è pigra, quando ha sviluppato un modulo funzionante, lo copierà e modificherà semplicemente per nuove funzioni). Il risultato netto di questa complicata danza è la consegna di molecole MHC di classe II sulla superficie cellulare con peptidi che consentono l'attivazione delle cellule T-helper.

Diapositiva 23

Questo processo di riconoscimento dei patogeni da parte del sistema immunitario è complesso... ma è anche relativamente lento. La prima volta che incontri un virus, il sistema immunitario impiega tempo per intensificare la risposta antivirale. Se sei sfortunato, ciò può risultare nel manifestarsi di malattia o morte a causa della replicazione virale incontrollata. La vaccinazione prepara il sistema immunitario a un'infezione, consentendo in alcuni casi di prevenire completamente l'infezione e, in altri casi, di rispondere più rapidamente ed efficacemente e di ridurre notevolmente le possibilità di un'infezione grave.

Diapositiva 24

Le molecole MHC sono partecipanti critici nella vaccinazione. Tutti i vaccini utilizzano molecole MHC di classe II per indurre le cellule T-helper necessarie per la risposta anticorpale e produrre le proteine contro le quali sono dirette le risposte anticorpali stesse. Anche i vaccini ad adenovirus e mRNA utilizzano molecole MHC di classe I per indurre le cellule T-killer. Le cellule T indotte dai vaccini durano molti anni, in alcuni casi anche decenni, in allerta per una nuova infezione con il virus originale. I vaccini hanno salvato molte più vite di tutti gli altri interventi medici messi insieme. Diffondi questo messaggio, non la malattia, fatti vaccinare!

Epilogo

Così le molecole MHC controllano le infezioni, regolano le risposte immunitarie e ora stanno aiutando a curare il cancro. Questo vale lo svantaggio dell'autoimmunità e del rigetto del trapianto. Ed è per questo che tu, che vivi in un mondo pieno di agenti patogeni, sei sopravvissuto per riuscire a leggere questo fumetto. Per maggiori dettagli su come sopravvivere ancora meglio, vedere le referenze 1–6.
