# Supplementary material for: HLA molecules in transplantation, autoimmunity and infection control: A comic book adventure
Source: HLA. 2022 May 15;100(4):301–11. doi: 10.1111/tan.14626 (PMC9545814; doi:10.1111/tan.14626)
Supplement: Supplementary file 1 — Supporting information. [file TAN-100-301-s001.zip › Supplementary files/PP_Norwegian_Bakke.1.pptx]

## Slide 1
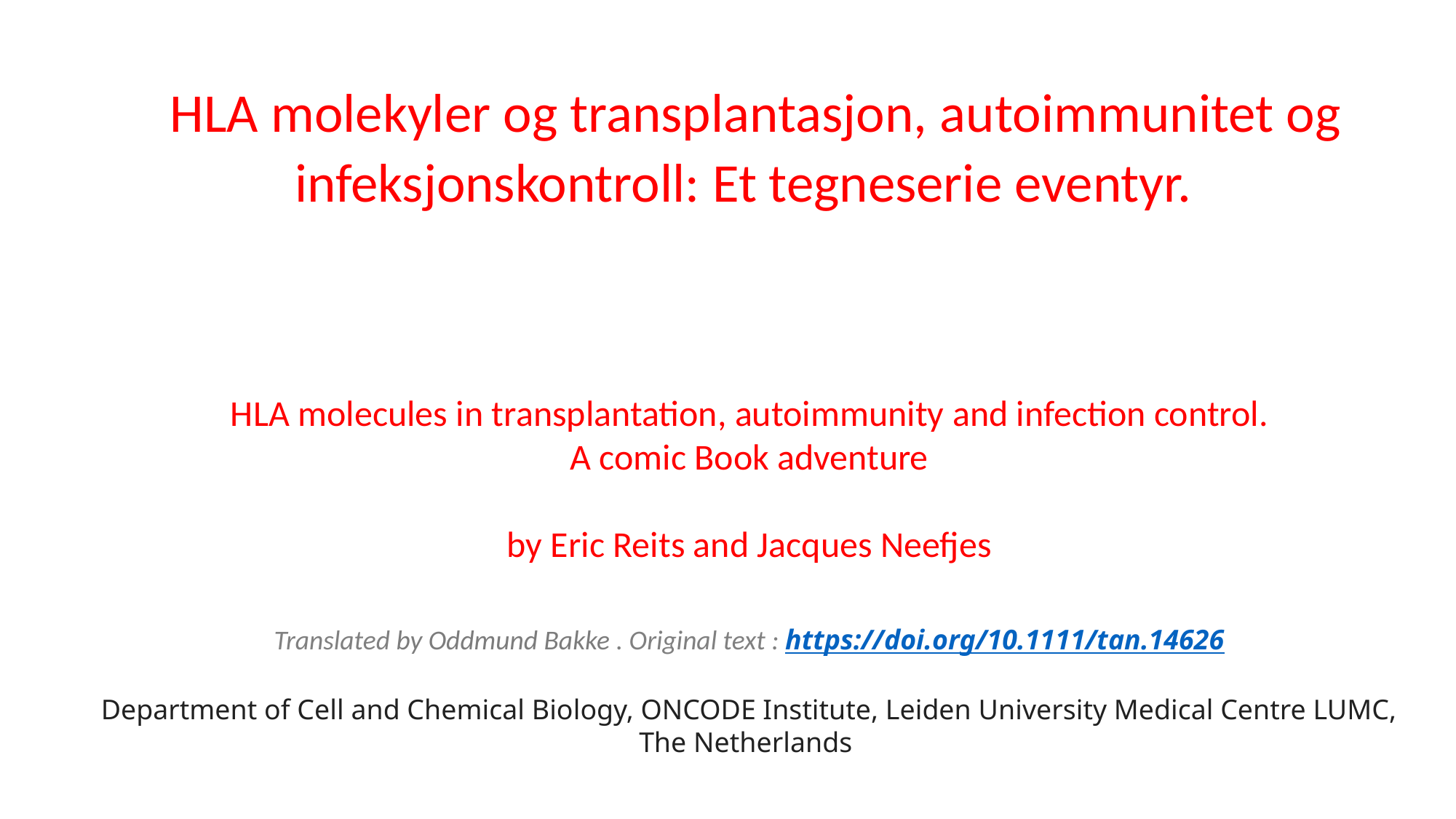

HLA molekyler og transplantasjon, autoimmunitet og infeksjonskontroll: Et tegneserie eventyr.
HLA molecules in transplantation, autoimmunity and infection control.
A comic Book adventure
by Eric Reits and Jacques Neefjes
Translated by Oddmund Bakke . Original text : https://doi.org/10.1111/tan.14626
Department of Cell and Chemical Biology, ONCODE Institute, Leiden University Medical Centre LUMC, The Netherlands

## Slide 2
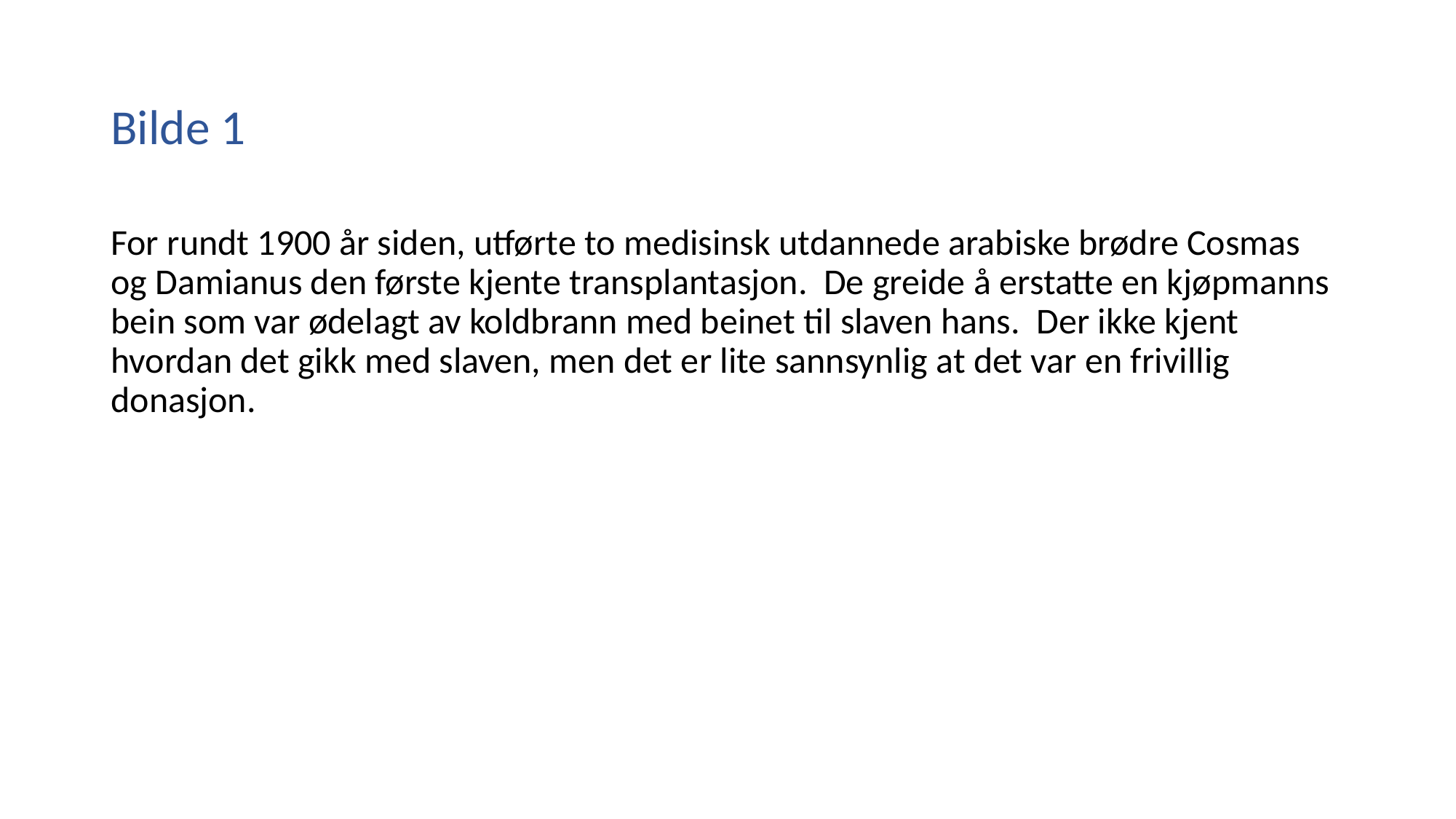

# Bilde 1
For rundt 1900 år siden, utførte to medisinsk utdannede arabiske brødre Cosmas og Damianus den første kjente transplantasjon. De greide å erstatte en kjøpmanns bein som var ødelagt av koldbrann med beinet til slaven hans. Der ikke kjent hvordan det gikk med slaven, men det er lite sannsynlig at det var en frivillig donasjon.

## Slide 3
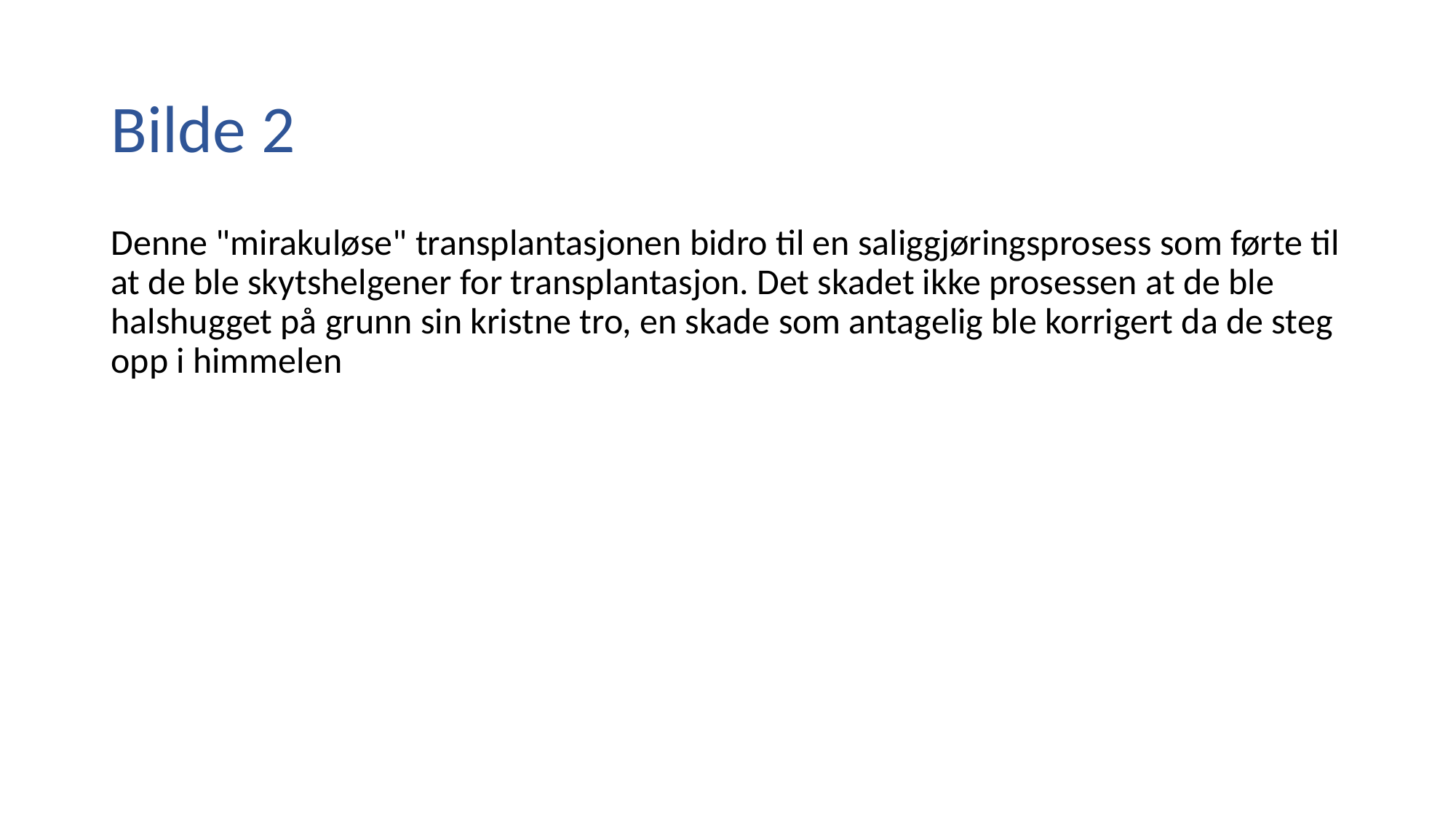

# Bilde 2
Denne "mirakuløse" transplantasjonen bidro til en saliggjøringsprosess som førte til at de ble skytshelgener for transplantasjon. Det skadet ikke prosessen at de ble halshugget på grunn sin kristne tro, en skade som antagelig ble korrigert da de steg opp i himmelen

## Slide 4
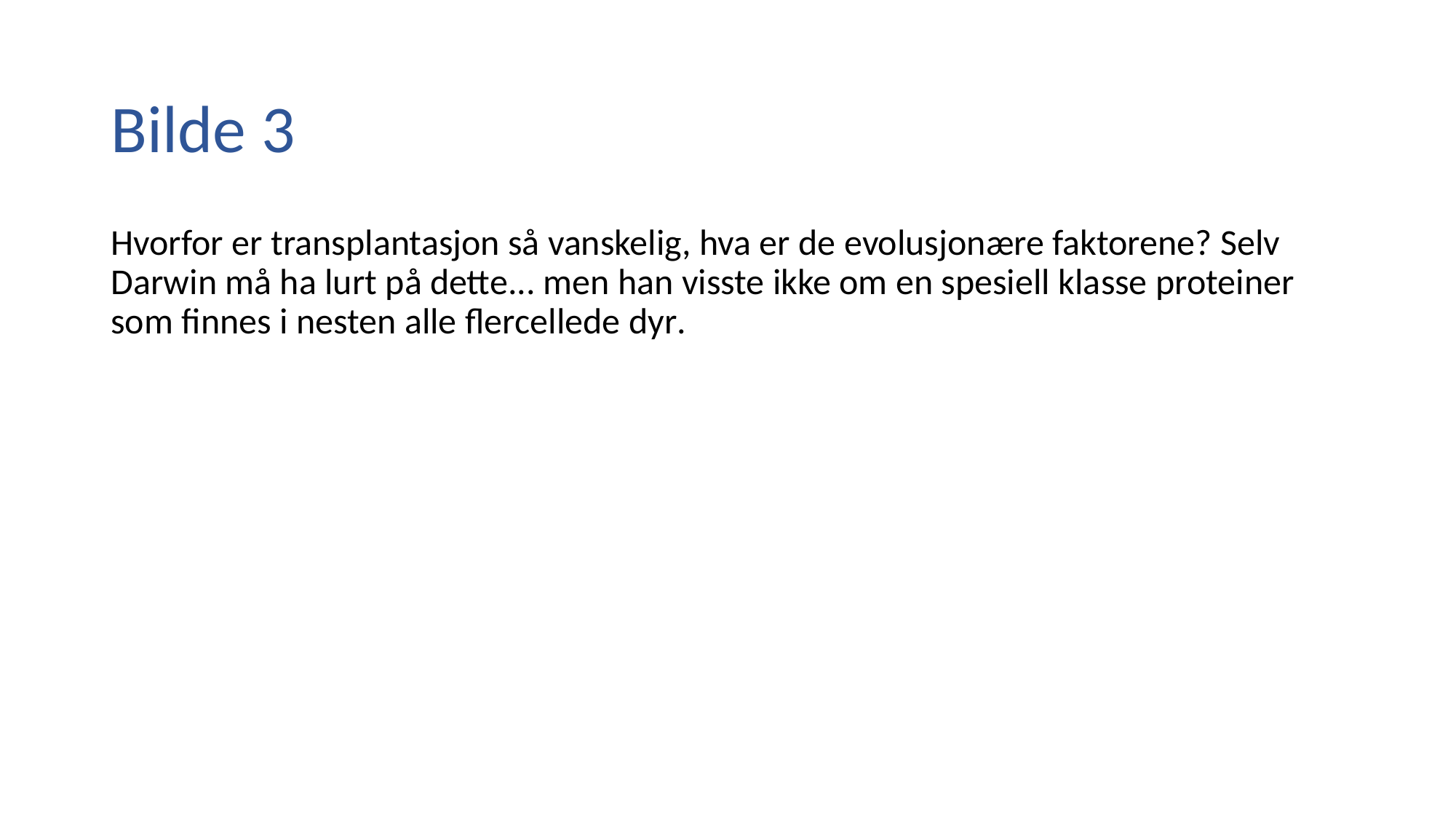

# Bilde 3
Hvorfor er transplantasjon så vanskelig, hva er de evolusjonære faktorene? Selv Darwin må ha lurt på dette... men han visste ikke om en spesiell klasse proteiner som finnes i nesten alle flercellede dyr.

## Slide 5
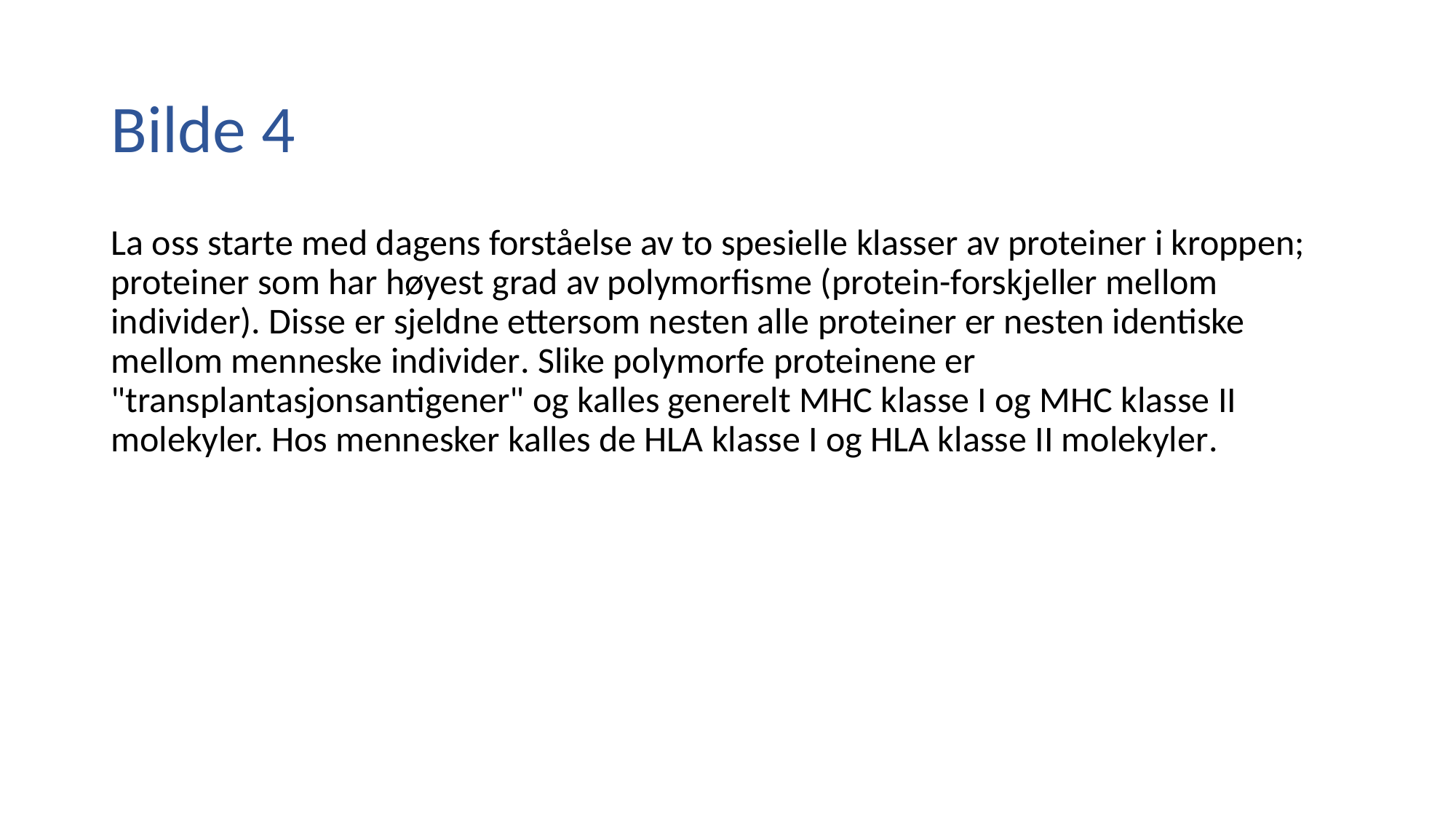

# Bilde 4
La oss starte med dagens forståelse av to spesielle klasser av proteiner i kroppen; proteiner som har høyest grad av polymorfisme (protein-forskjeller mellom individer). Disse er sjeldne ettersom nesten alle proteiner er nesten identiske mellom menneske individer. Slike polymorfe proteinene er "transplantasjonsantigener" og kalles generelt MHC klasse I og MHC klasse II molekyler. Hos mennesker kalles de HLA klasse I og HLA klasse II molekyler.

## Slide 6
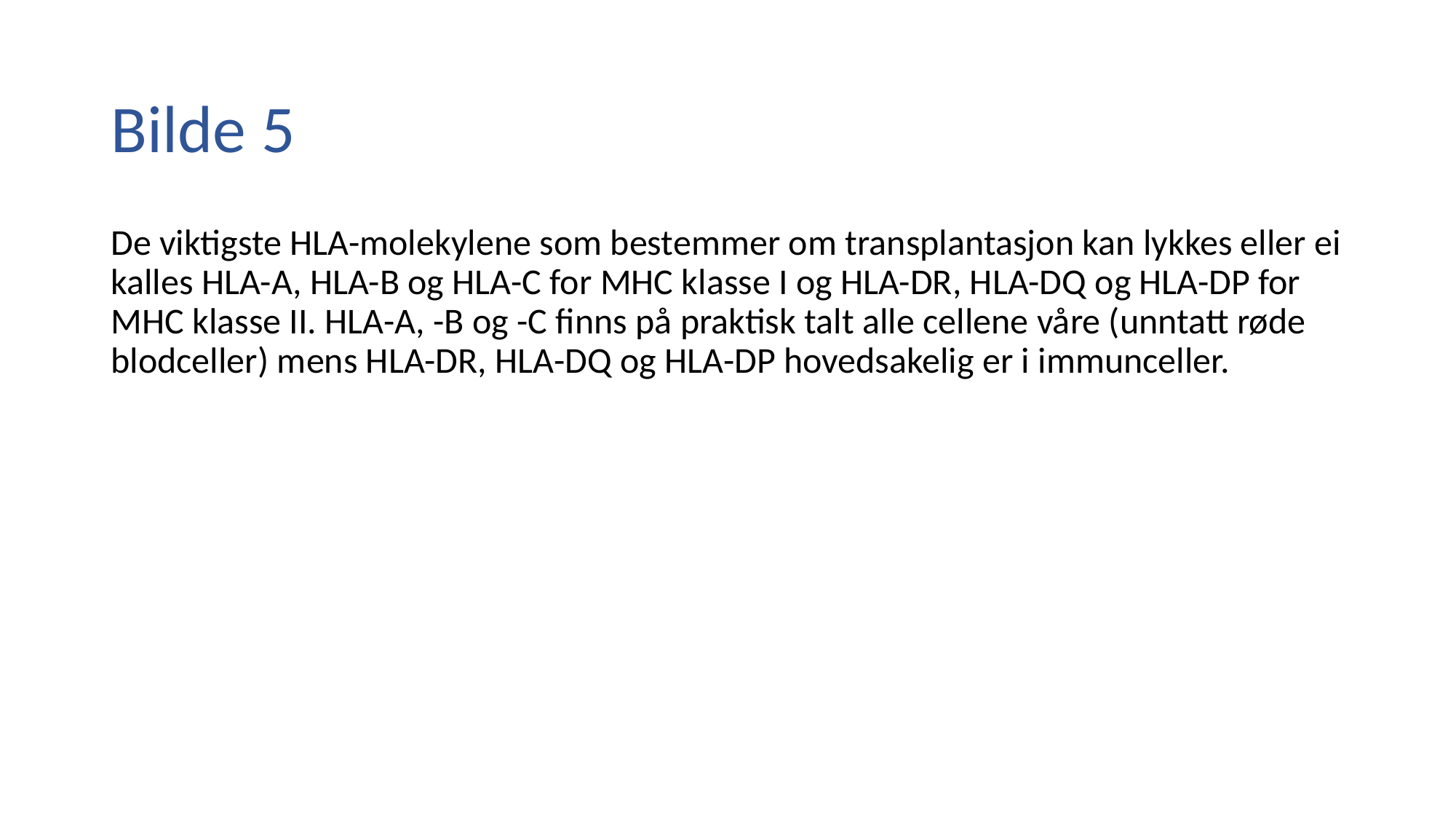

# Bilde 5
De viktigste HLA-molekylene som bestemmer om transplantasjon kan lykkes eller ei kalles HLA-A, HLA-B og HLA-C for MHC klasse I og HLA-DR, HLA-DQ og HLA-DP for MHC klasse II. HLA-A, -B og -C finns på praktisk talt alle cellene våre (unntatt røde blodceller) mens HLA-DR, HLA-DQ og HLA-DP hovedsakelig er i immunceller.

## Slide 7
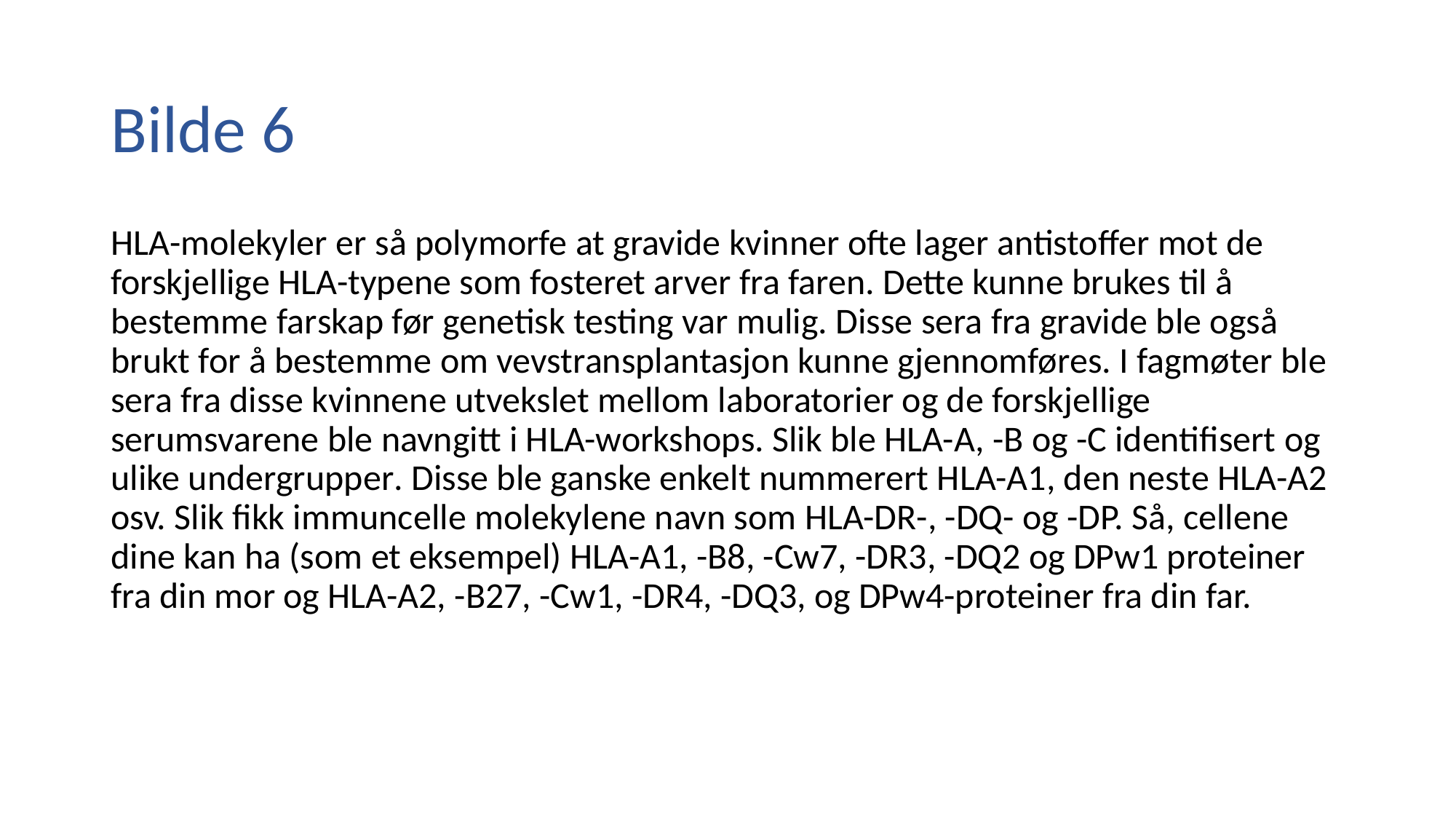

# Bilde 6
HLA-molekyler er så polymorfe at gravide kvinner ofte lager antistoffer mot de forskjellige HLA-typene som fosteret arver fra faren. Dette kunne brukes til å bestemme farskap før genetisk testing var mulig. Disse sera fra gravide ble også brukt for å bestemme om vevstransplantasjon kunne gjennomføres. I fagmøter ble sera fra disse kvinnene utvekslet mellom laboratorier og de forskjellige serumsvarene ble navngitt i HLA-workshops. Slik ble HLA-A, -B og -C identifisert og ulike undergrupper. Disse ble ganske enkelt nummerert HLA-A1, den neste HLA-A2 osv. Slik fikk immuncelle molekylene navn som HLA-DR-, -DQ- og -DP. Så, cellene dine kan ha (som et eksempel) HLA-A1, -B8, -Cw7, -DR3, -DQ2 og DPw1 proteiner fra din mor og HLA-A2, -B27, -Cw1, -DR4, -DQ3, og DPw4-proteiner fra din far.

## Slide 8
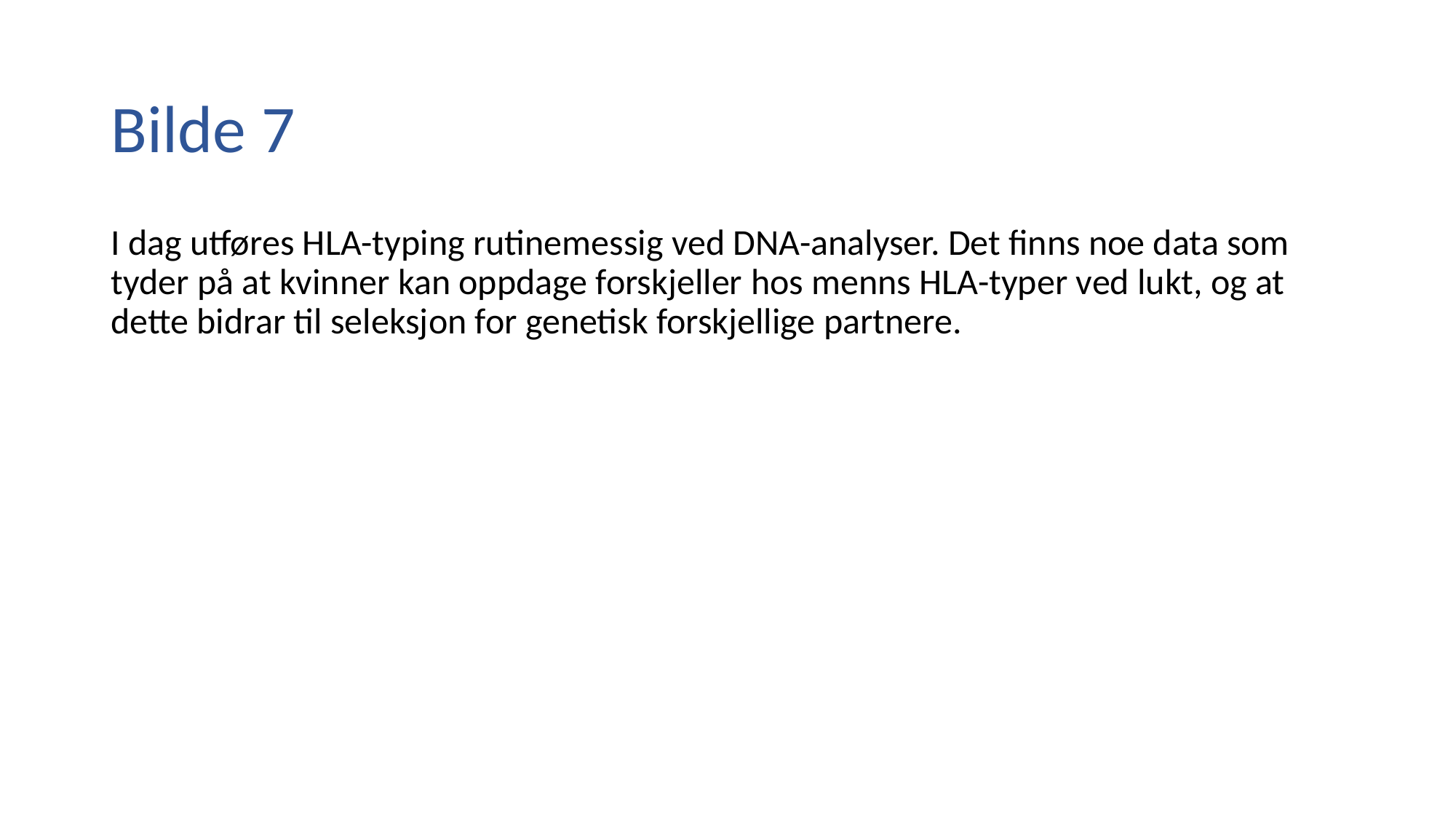

# Bilde 7
I dag utføres HLA-typing rutinemessig ved DNA-analyser. Det finns noe data som tyder på at kvinner kan oppdage forskjeller hos menns HLA-typer ved lukt, og at dette bidrar til seleksjon for genetisk forskjellige partnere.

## Slide 9
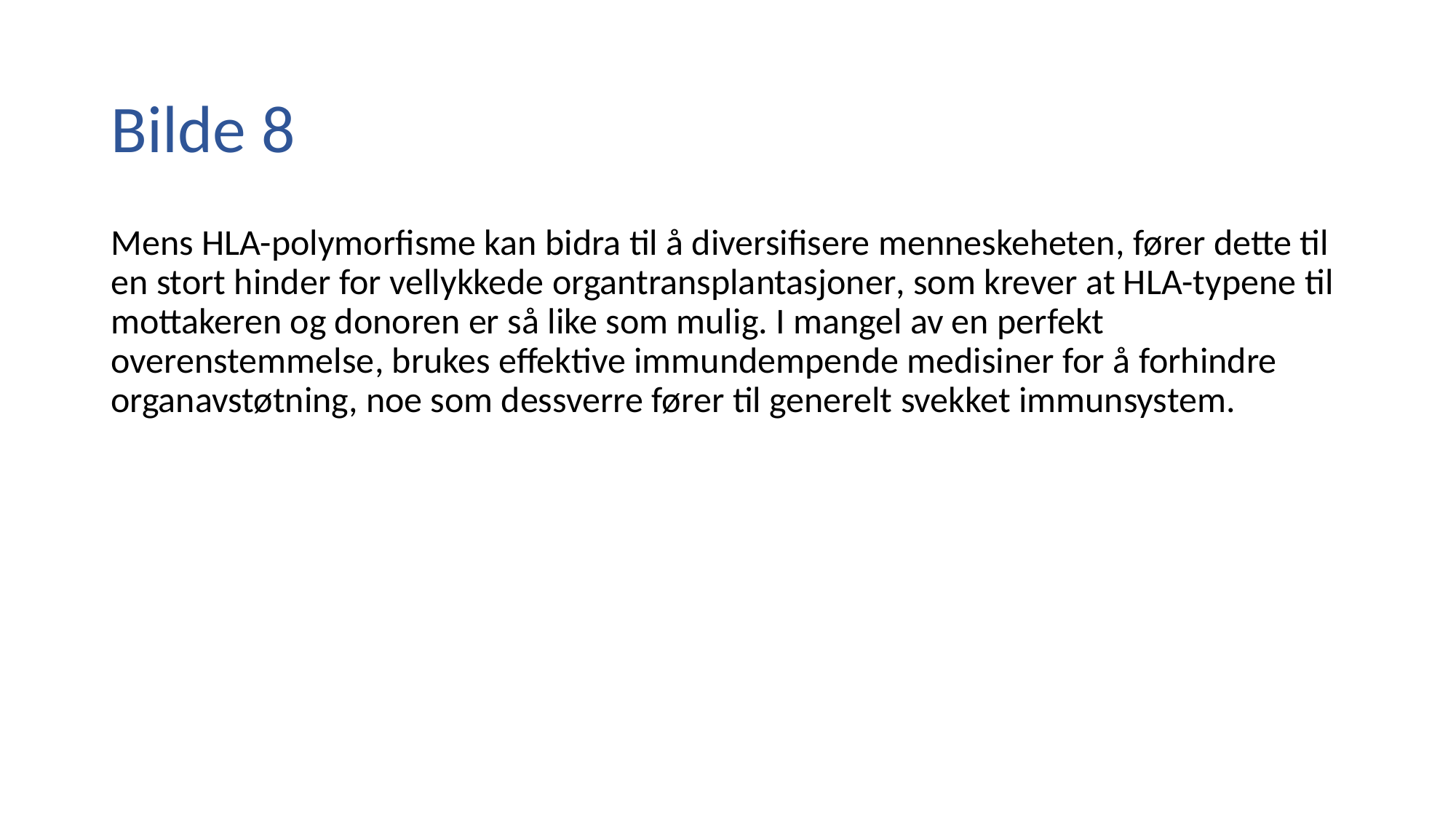

# Bilde 8
Mens HLA-polymorfisme kan bidra til å diversifisere menneskeheten, fører dette til en stort hinder for vellykkede organtransplantasjoner, som krever at HLA-typene til mottakeren og donoren er så like som mulig. I mangel av en perfekt overenstemmelse, brukes effektive immundempende medisiner for å forhindre organavstøtning, noe som dessverre fører til generelt svekket immunsystem.

## Slide 10
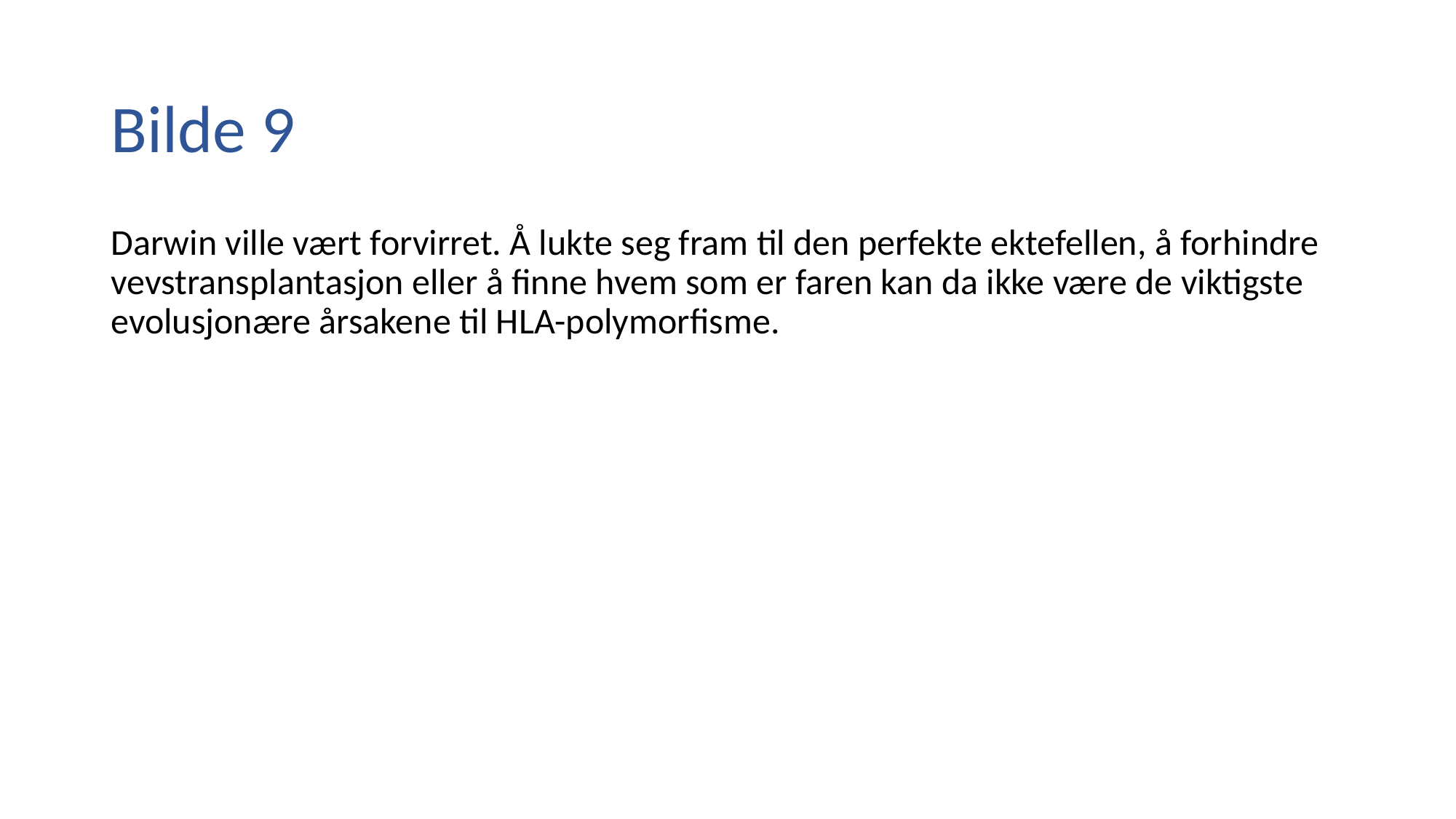

# Bilde 9
Darwin ville vært forvirret. Å lukte seg fram til den perfekte ektefellen, å forhindre vevstransplantasjon eller å finne hvem som er faren kan da ikke være de viktigste evolusjonære årsakene til HLA-polymorfisme.

## Slide 11
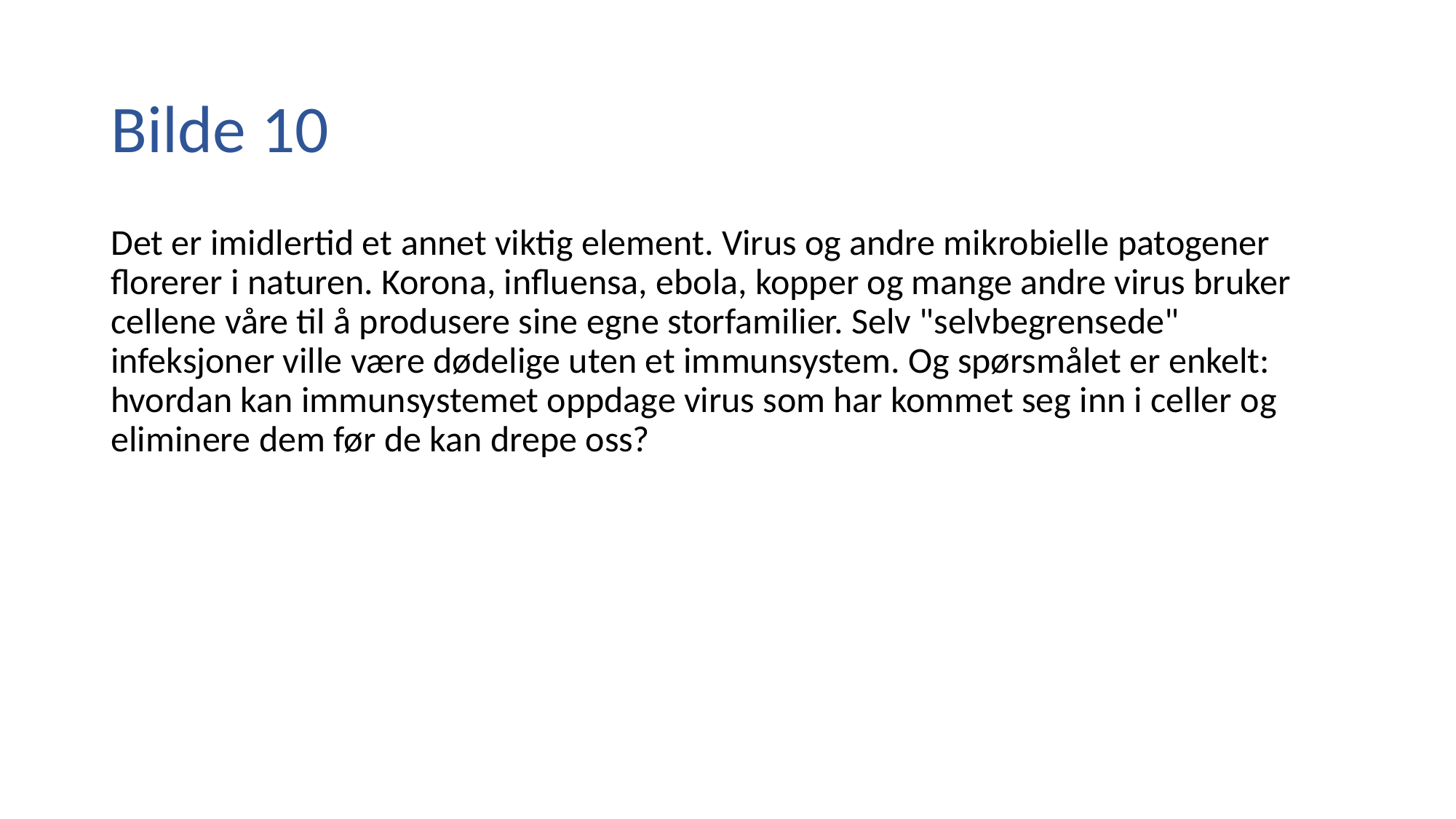

# Bilde 10
Det er imidlertid et annet viktig element. Virus og andre mikrobielle patogener florerer i naturen. Korona, influensa, ebola, kopper og mange andre virus bruker cellene våre til å produsere sine egne storfamilier. Selv "selvbegrensede" infeksjoner ville være dødelige uten et immunsystem. Og spørsmålet er enkelt: hvordan kan immunsystemet oppdage virus som har kommet seg inn i celler og eliminere dem før de kan drepe oss?

## Slide 12
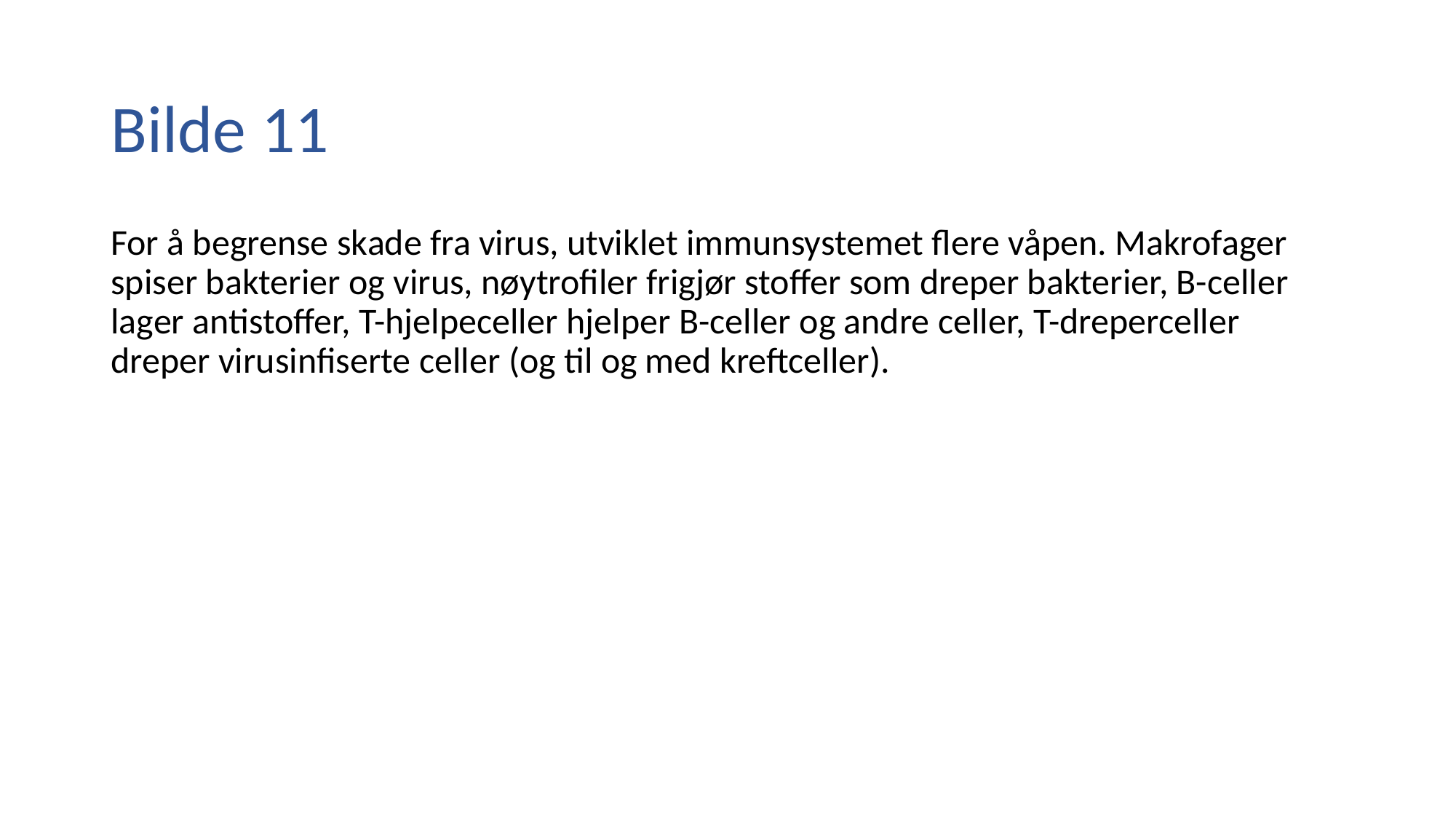

# Bilde 11
For å begrense skade fra virus, utviklet immunsystemet flere våpen. Makrofager spiser bakterier og virus, nøytrofiler frigjør stoffer som dreper bakterier, B-celler lager antistoffer, T-hjelpeceller hjelper B-celler og andre celler, T-dreperceller dreper virusinfiserte celler (og til og med kreftceller).

## Slide 13
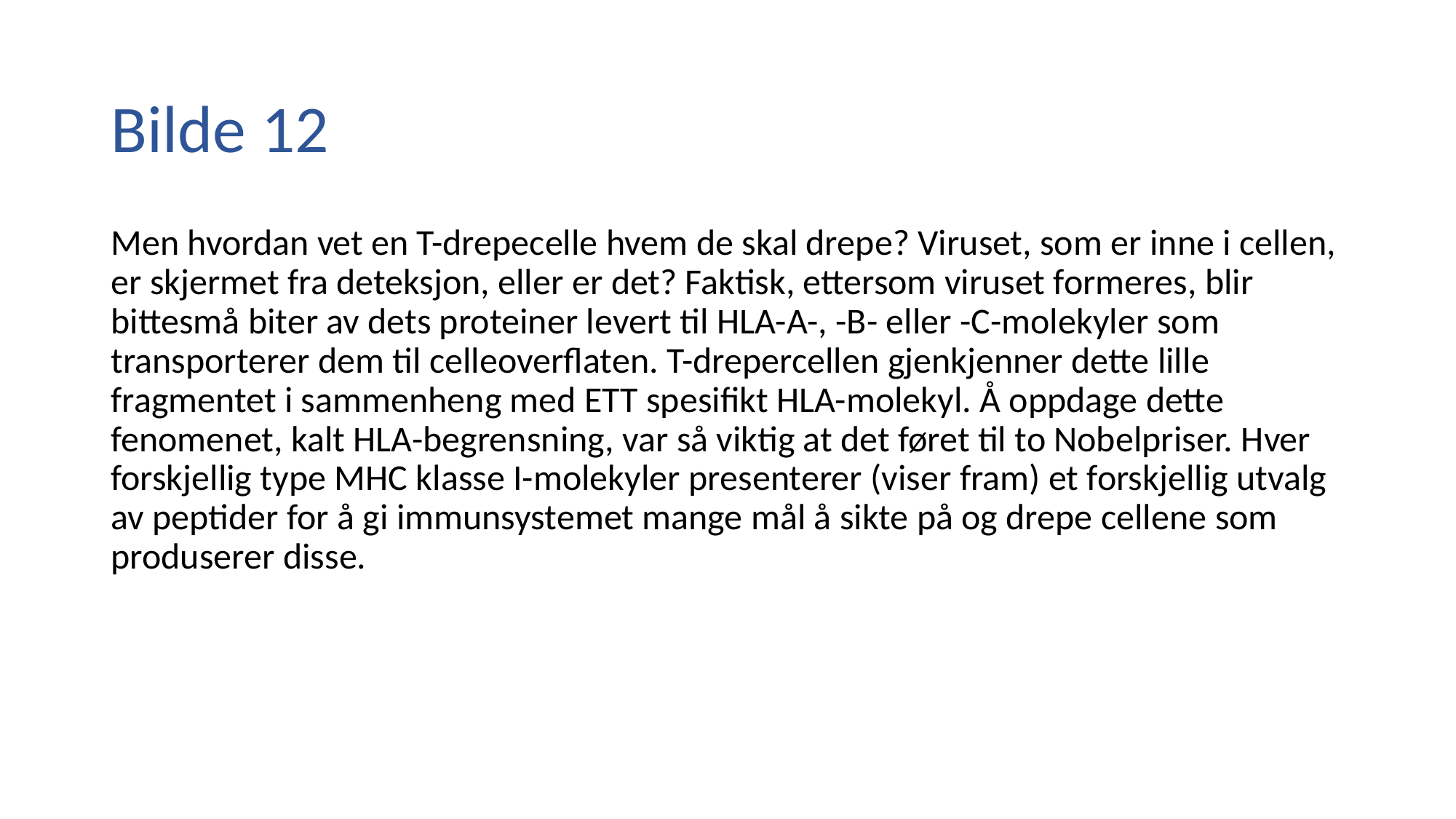

# Bilde 12
Men hvordan vet en T-drepecelle hvem de skal drepe? Viruset, som er inne i cellen, er skjermet fra deteksjon, eller er det? Faktisk, ettersom viruset formeres, blir bittesmå biter av dets proteiner levert til HLA-A-, -B- eller -C-molekyler som transporterer dem til celleoverflaten. T-drepercellen gjenkjenner dette lille fragmentet i sammenheng med ETT spesifikt HLA-molekyl. Å oppdage dette fenomenet, kalt HLA-begrensning, var så viktig at det føret til to Nobelpriser. Hver forskjellig type MHC klasse I-molekyler presenterer (viser fram) et forskjellig utvalg av peptider for å gi immunsystemet mange mål å sikte på og drepe cellene som produserer disse.

## Slide 14
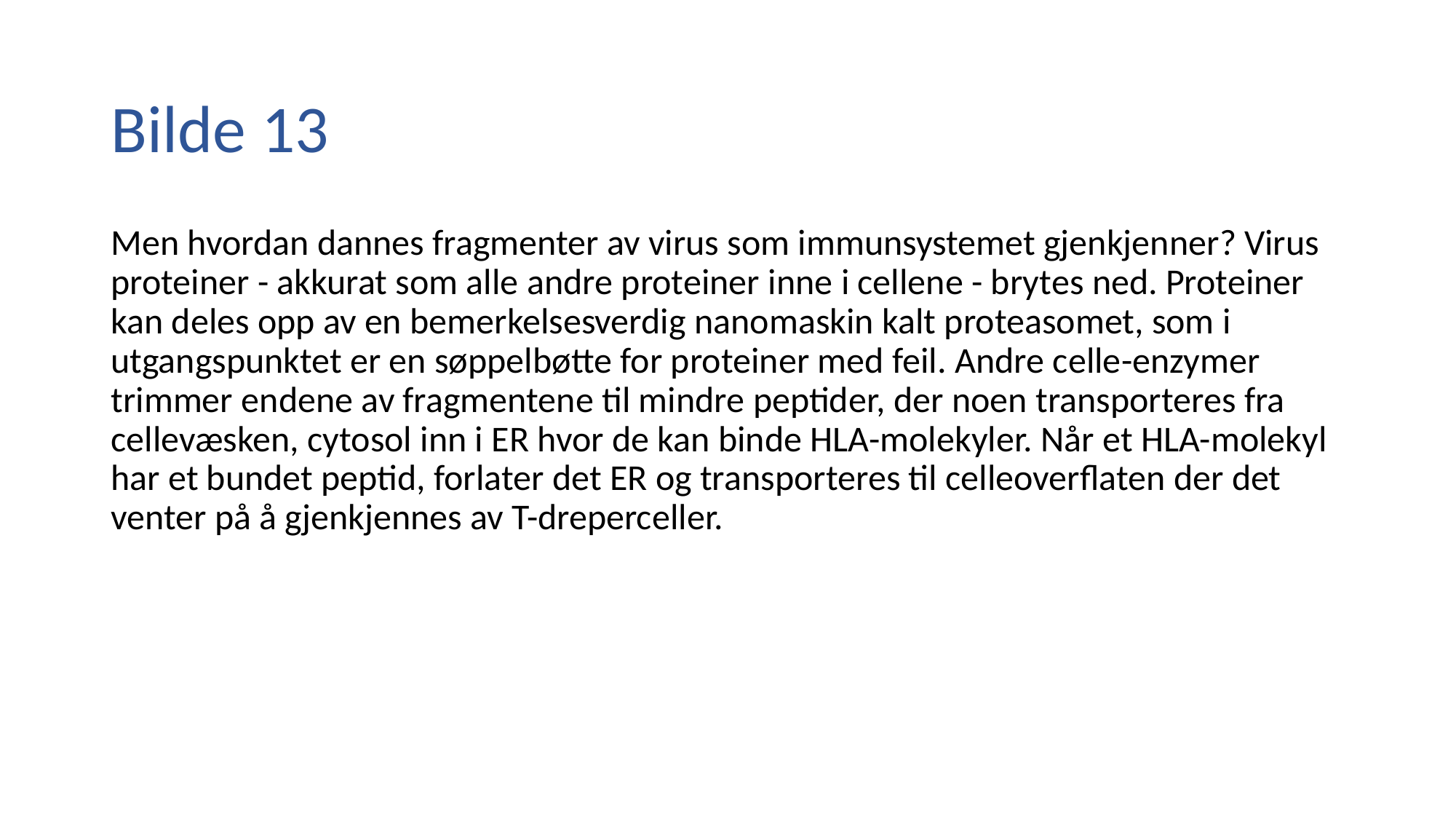

# Bilde 13
Men hvordan dannes fragmenter av virus som immunsystemet gjenkjenner? Virus proteiner - akkurat som alle andre proteiner inne i cellene - brytes ned. Proteiner kan deles opp av en bemerkelsesverdig nanomaskin kalt proteasomet, som i utgangspunktet er en søppelbøtte for proteiner med feil. Andre celle-enzymer trimmer endene av fragmentene til mindre peptider, der noen transporteres fra cellevæsken, cytosol inn i ER hvor de kan binde HLA-molekyler. Når et HLA-molekyl har et bundet peptid, forlater det ER og transporteres til celleoverflaten der det venter på å gjenkjennes av T-dreperceller.

## Slide 15
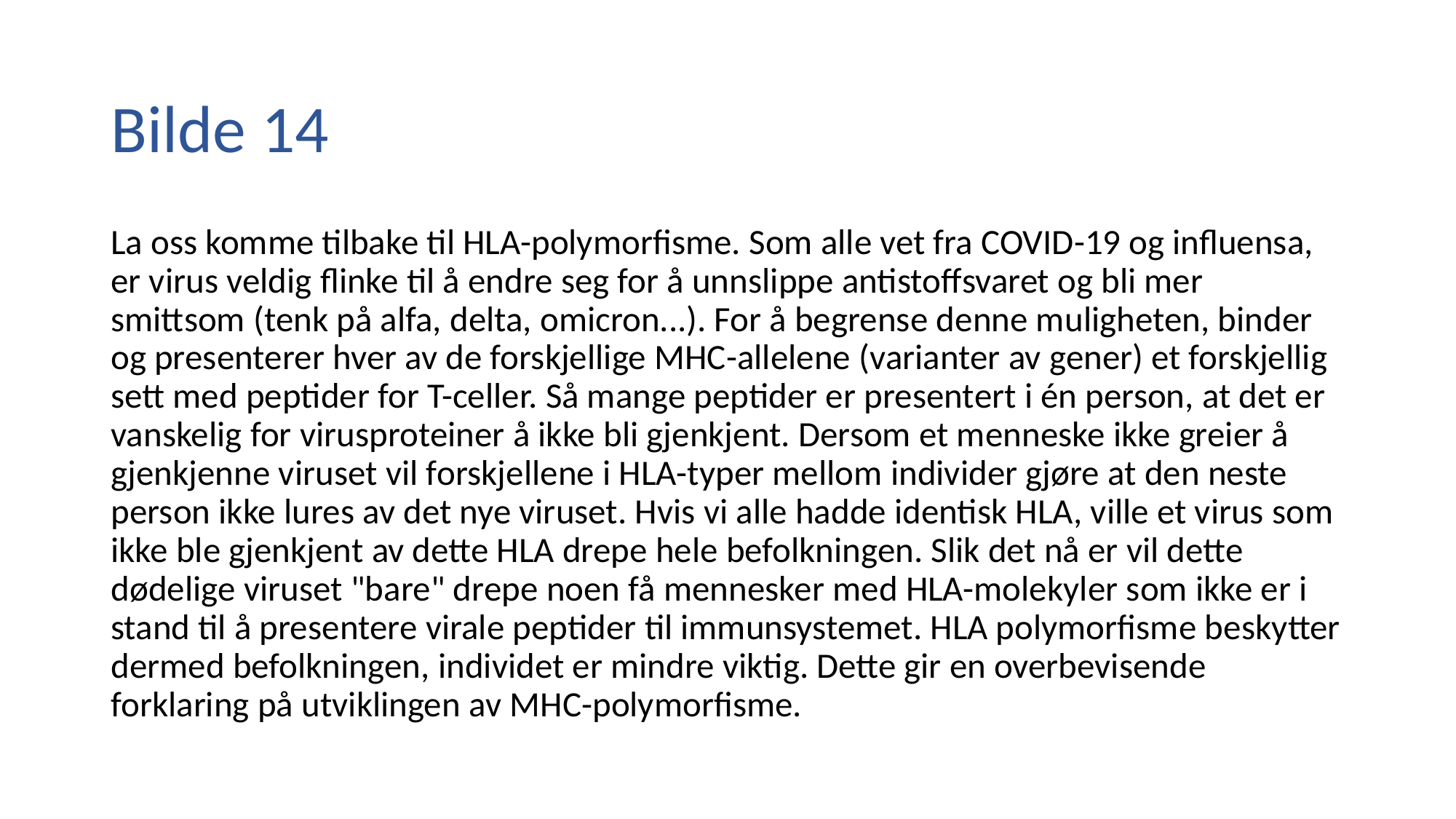

# Bilde 14
La oss komme tilbake til HLA-polymorfisme. Som alle vet fra COVID-19 og influensa, er virus veldig flinke til å endre seg for å unnslippe antistoffsvaret og bli mer smittsom (tenk på alfa, delta, omicron...). For å begrense denne muligheten, binder og presenterer hver av de forskjellige MHC-allelene (varianter av gener) et forskjellig sett med peptider for T-celler. Så mange peptider er presentert i én person, at det er vanskelig for virusproteiner å ikke bli gjenkjent. Dersom et menneske ikke greier å gjenkjenne viruset vil forskjellene i HLA-typer mellom individer gjøre at den neste person ikke lures av det nye viruset. Hvis vi alle hadde identisk HLA, ville et virus som ikke ble gjenkjent av dette HLA drepe hele befolkningen. Slik det nå er vil dette dødelige viruset "bare" drepe noen få mennesker med HLA-molekyler som ikke er i stand til å presentere virale peptider til immunsystemet. HLA polymorfisme beskytter dermed befolkningen, individet er mindre viktig. Dette gir en overbevisende forklaring på utviklingen av MHC-polymorfisme.

## Slide 16
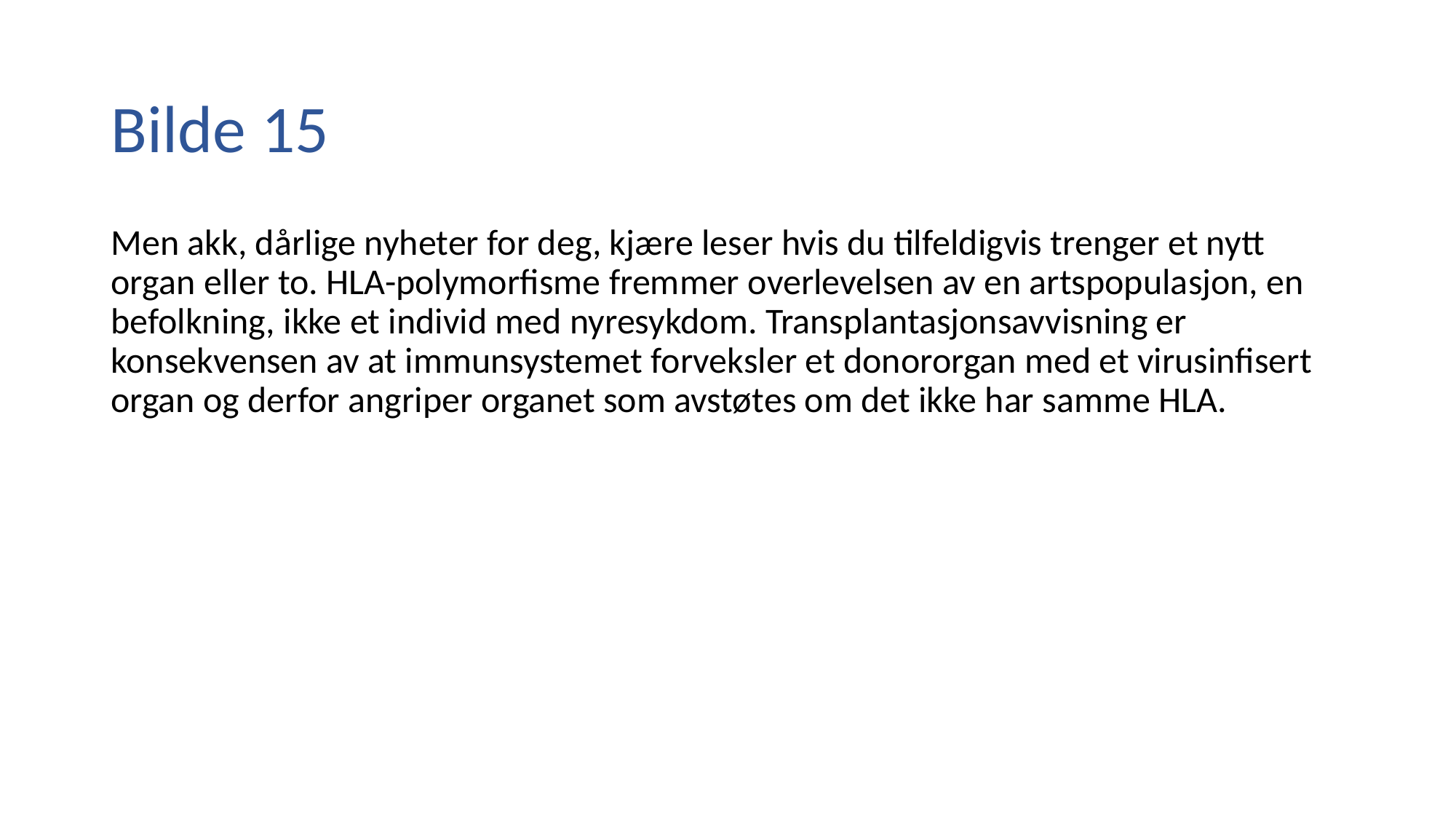

# Bilde 15
Men akk, dårlige nyheter for deg, kjære leser hvis du tilfeldigvis trenger et nytt organ eller to. HLA-polymorfisme fremmer overlevelsen av en artspopulasjon, en befolkning, ikke et individ med nyresykdom. Transplantasjonsavvisning er konsekvensen av at immunsystemet forveksler et donororgan med et virusinfisert organ og derfor angriper organet som avstøtes om det ikke har samme HLA.

## Slide 17
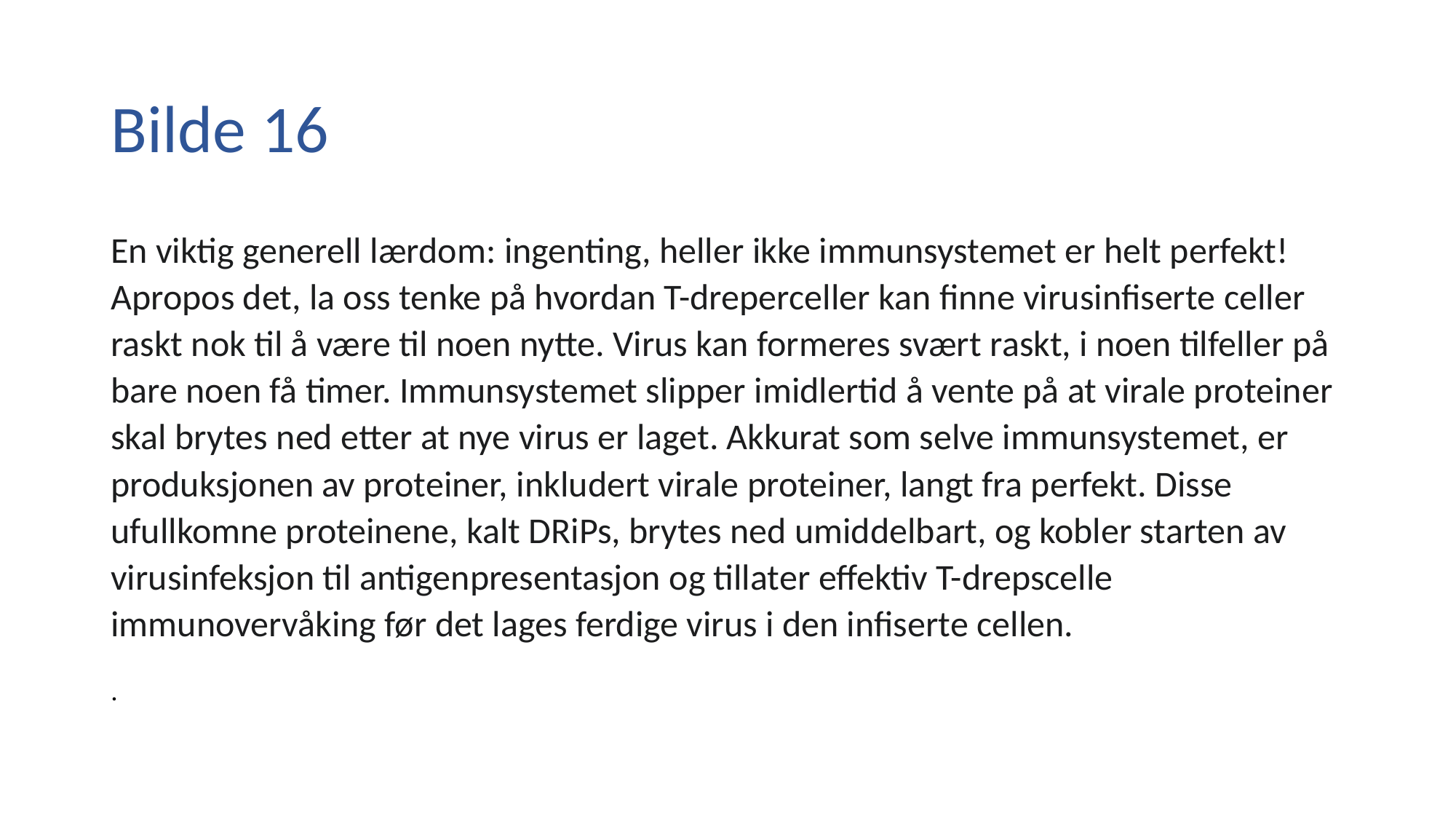

# Bilde 16
En viktig generell lærdom: ingenting, heller ikke immunsystemet er helt perfekt! Apropos det, la oss tenke på hvordan T-dreperceller kan finne virusinfiserte celler raskt nok til å være til noen nytte. Virus kan formeres svært raskt, i noen tilfeller på bare noen få timer. Immunsystemet slipper imidlertid å vente på at virale proteiner skal brytes ned etter at nye virus er laget. Akkurat som selve immunsystemet, er produksjonen av proteiner, inkludert virale proteiner, langt fra perfekt. Disse ufullkomne proteinene, kalt DRiPs, brytes ned umiddelbart, og kobler starten av virusinfeksjon til antigenpresentasjon og tillater effektiv T-drepscelle immunovervåking før det lages ferdige virus i den infiserte cellen.
.

## Slide 18
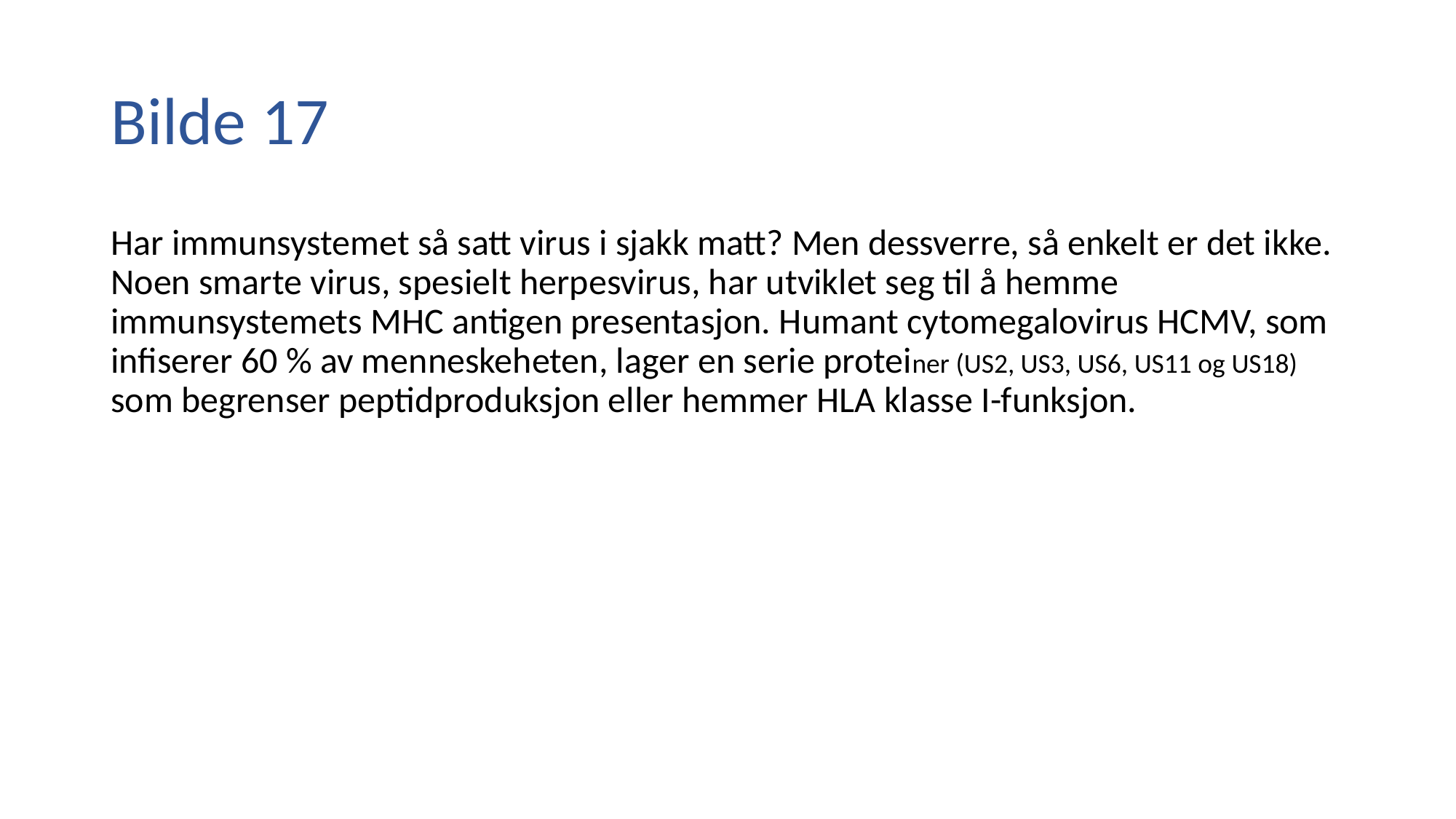

# Bilde 17
Har immunsystemet så satt virus i sjakk matt? Men dessverre, så enkelt er det ikke. Noen smarte virus, spesielt herpesvirus, har utviklet seg til å hemme immunsystemets MHC antigen presentasjon. Humant cytomegalovirus HCMV, som infiserer 60 % av menneskeheten, lager en serie proteiner (US2, US3, US6, US11 og US18) som begrenser peptidproduksjon eller hemmer HLA klasse I-funksjon.

## Slide 19
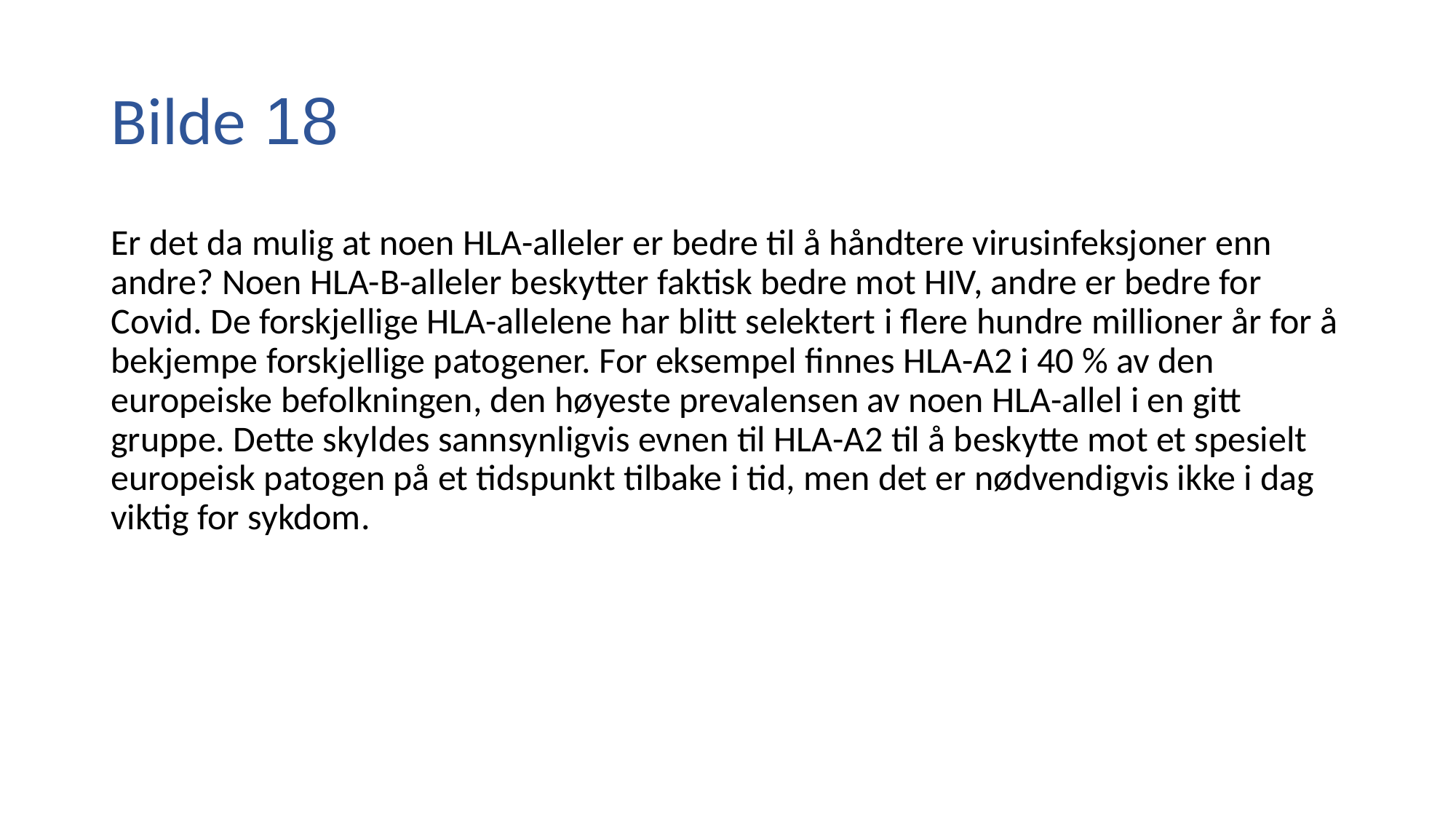

# Bilde 18
Er det da mulig at noen HLA-alleler er bedre til å håndtere virusinfeksjoner enn andre? Noen HLA-B-alleler beskytter faktisk bedre mot HIV, andre er bedre for Covid. De forskjellige HLA-allelene har blitt selektert i flere hundre millioner år for å bekjempe forskjellige patogener. For eksempel finnes HLA-A2 i 40 % av den europeiske befolkningen, den høyeste prevalensen av noen HLA-allel i en gitt gruppe. Dette skyldes sannsynligvis evnen til HLA-A2 til å beskytte mot et spesielt europeisk patogen på et tidspunkt tilbake i tid, men det er nødvendigvis ikke i dag viktig for sykdom.

## Slide 20
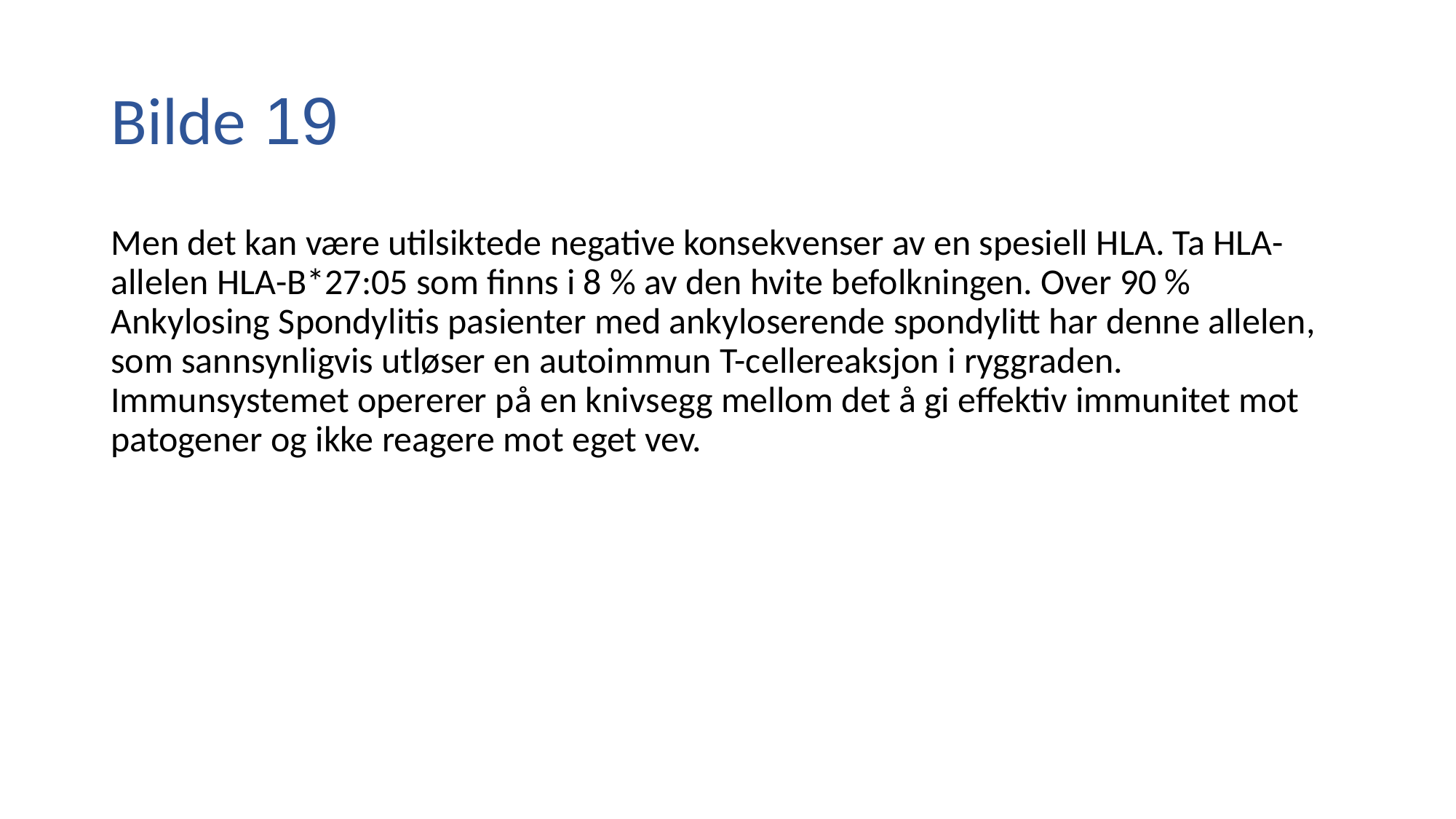

# Bilde 19
Men det kan være utilsiktede negative konsekvenser av en spesiell HLA. Ta HLA-allelen HLA-B*27:05 som finns i 8 % av den hvite befolkningen. Over 90 % Ankylosing Spondylitis pasienter med ankyloserende spondylitt har denne allelen, som sannsynligvis utløser en autoimmun T-cellereaksjon i ryggraden. Immunsystemet opererer på en knivsegg mellom det å gi effektiv immunitet mot patogener og ikke reagere mot eget vev.

## Slide 21
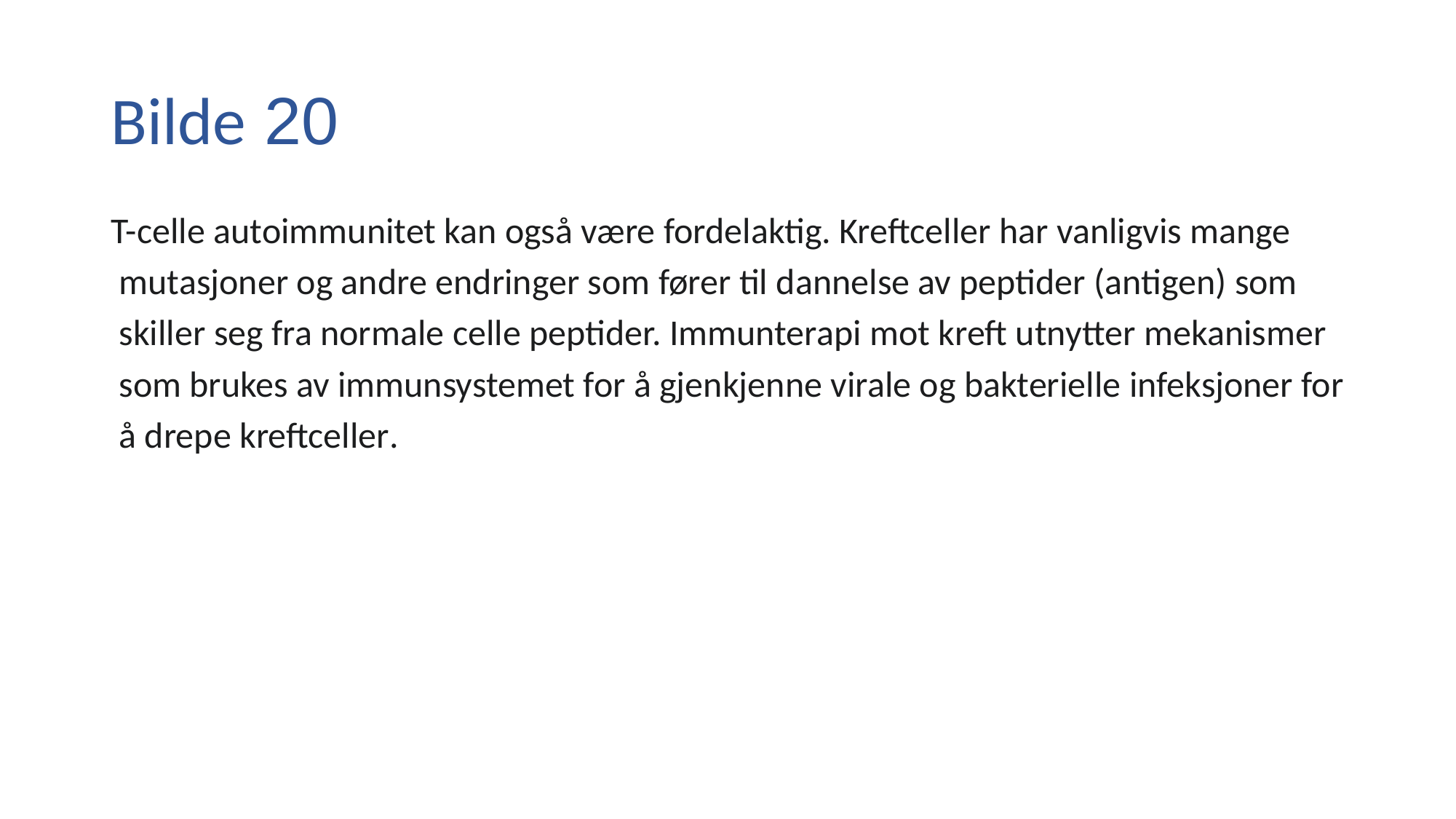

# Bilde 20
T-celle autoimmunitet kan også være fordelaktig. Kreftceller har vanligvis mange
 mutasjoner og andre endringer som fører til dannelse av peptider (antigen) som
 skiller seg fra normale celle peptider. Immunterapi mot kreft utnytter mekanismer
 som brukes av immunsystemet for å gjenkjenne virale og bakterielle infeksjoner for
 å drepe kreftceller.

## Slide 22
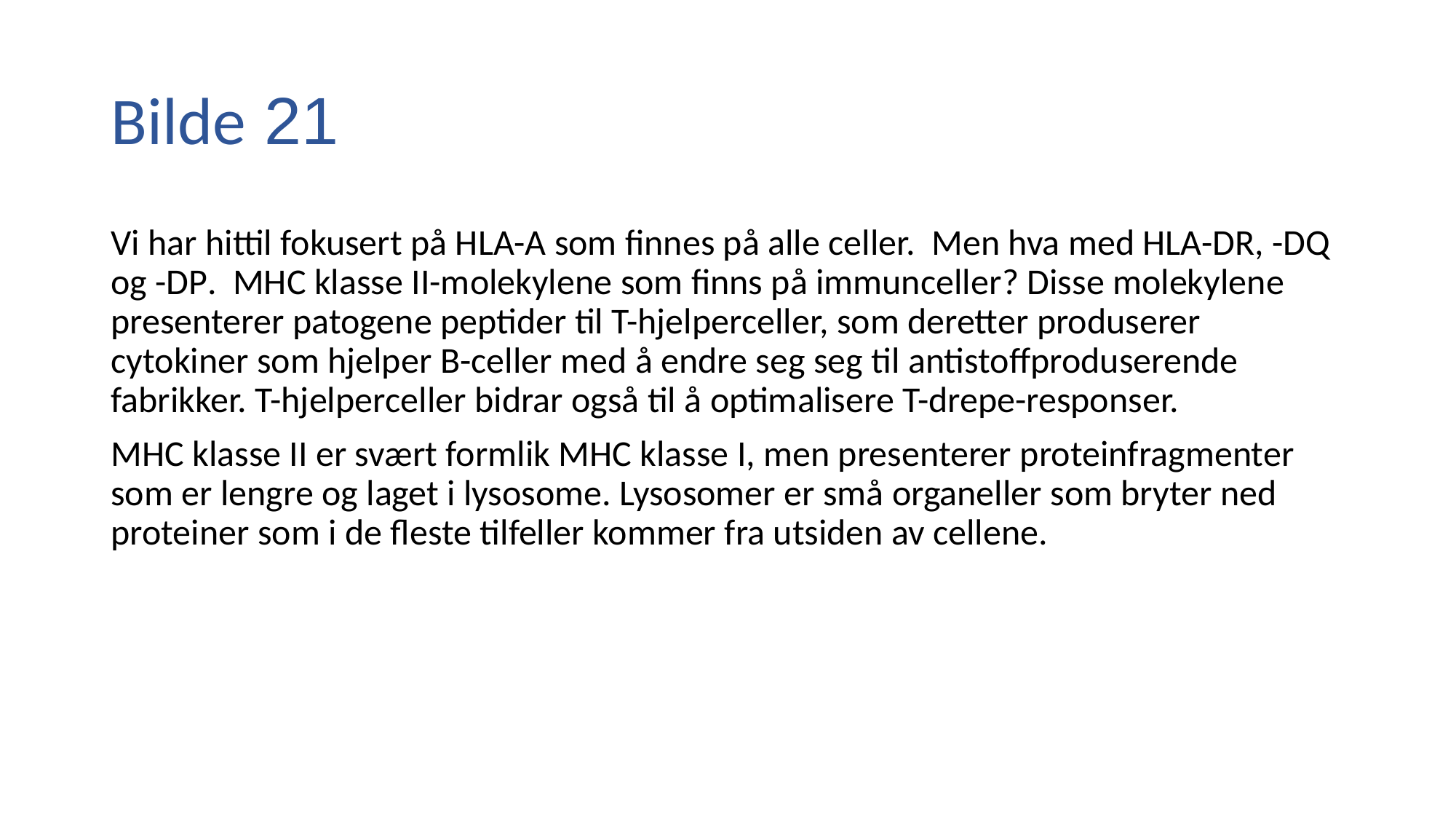

# Bilde 21
Vi har hittil fokusert på HLA-A som finnes på alle celler. Men hva med HLA-DR, -DQ og -DP. MHC klasse II-molekylene som finns på immunceller? Disse molekylene presenterer patogene peptider til T-hjelperceller, som deretter produserer cytokiner som hjelper B-celler med å endre seg seg til antistoffproduserende fabrikker. T-hjelperceller bidrar også til å optimalisere T-drepe-responser.
MHC klasse II er svært formlik MHC klasse I, men presenterer proteinfragmenter som er lengre og laget i lysosome. Lysosomer er små organeller som bryter ned proteiner som i de fleste tilfeller kommer fra utsiden av cellene.

## Slide 23
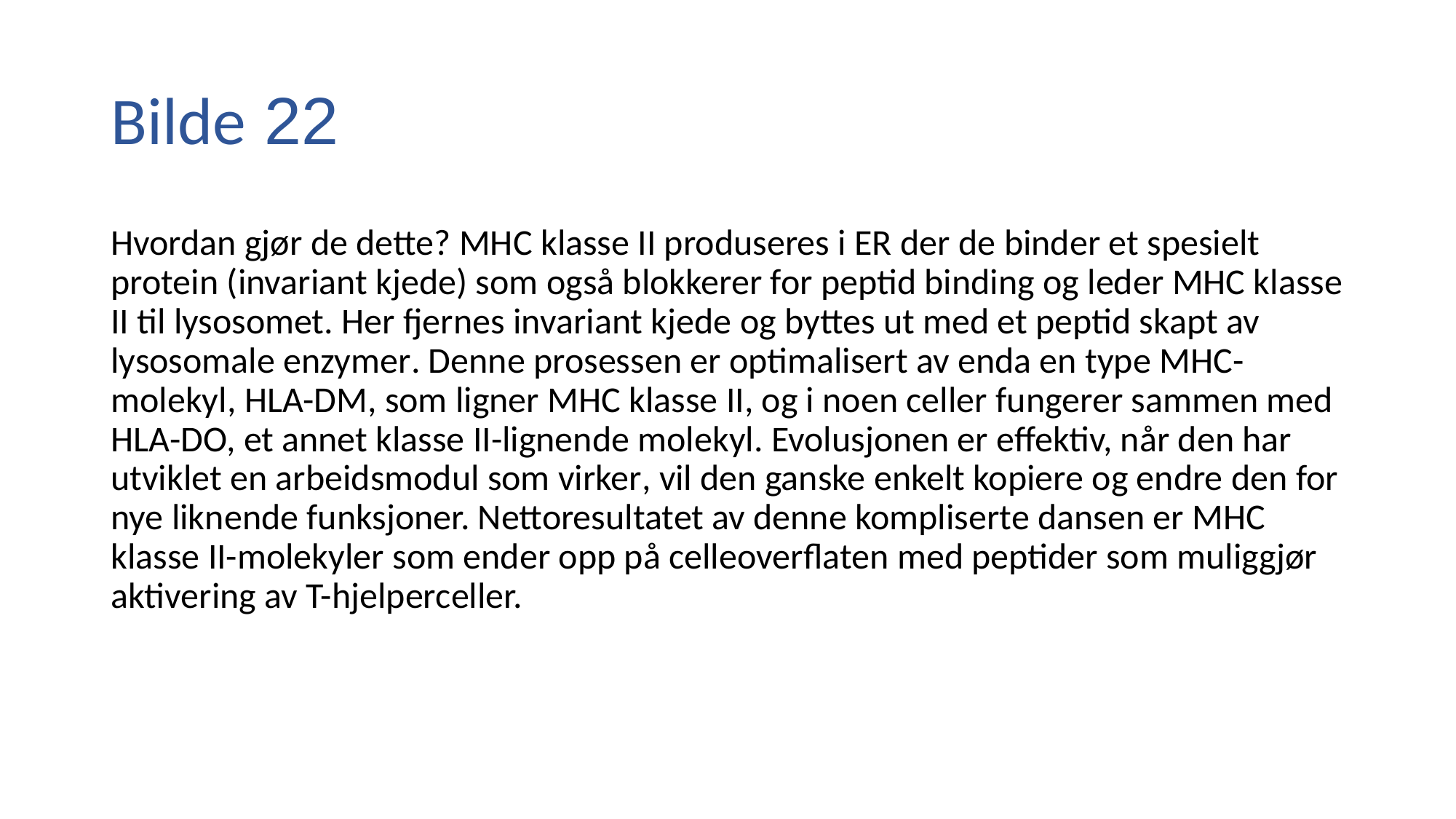

# Bilde 22
Hvordan gjør de dette? MHC klasse II produseres i ER der de binder et spesielt protein (invariant kjede) som også blokkerer for peptid binding og leder MHC klasse II til lysosomet. Her fjernes invariant kjede og byttes ut med et peptid skapt av lysosomale enzymer. Denne prosessen er optimalisert av enda en type MHC-molekyl, HLA-DM, som ligner MHC klasse II, og i noen celler fungerer sammen med HLA-DO, et annet klasse II-lignende molekyl. Evolusjonen er effektiv, når den har utviklet en arbeidsmodul som virker, vil den ganske enkelt kopiere og endre den for nye liknende funksjoner. Nettoresultatet av denne kompliserte dansen er MHC klasse II-molekyler som ender opp på celleoverflaten med peptider som muliggjør aktivering av T-hjelperceller.

## Slide 24
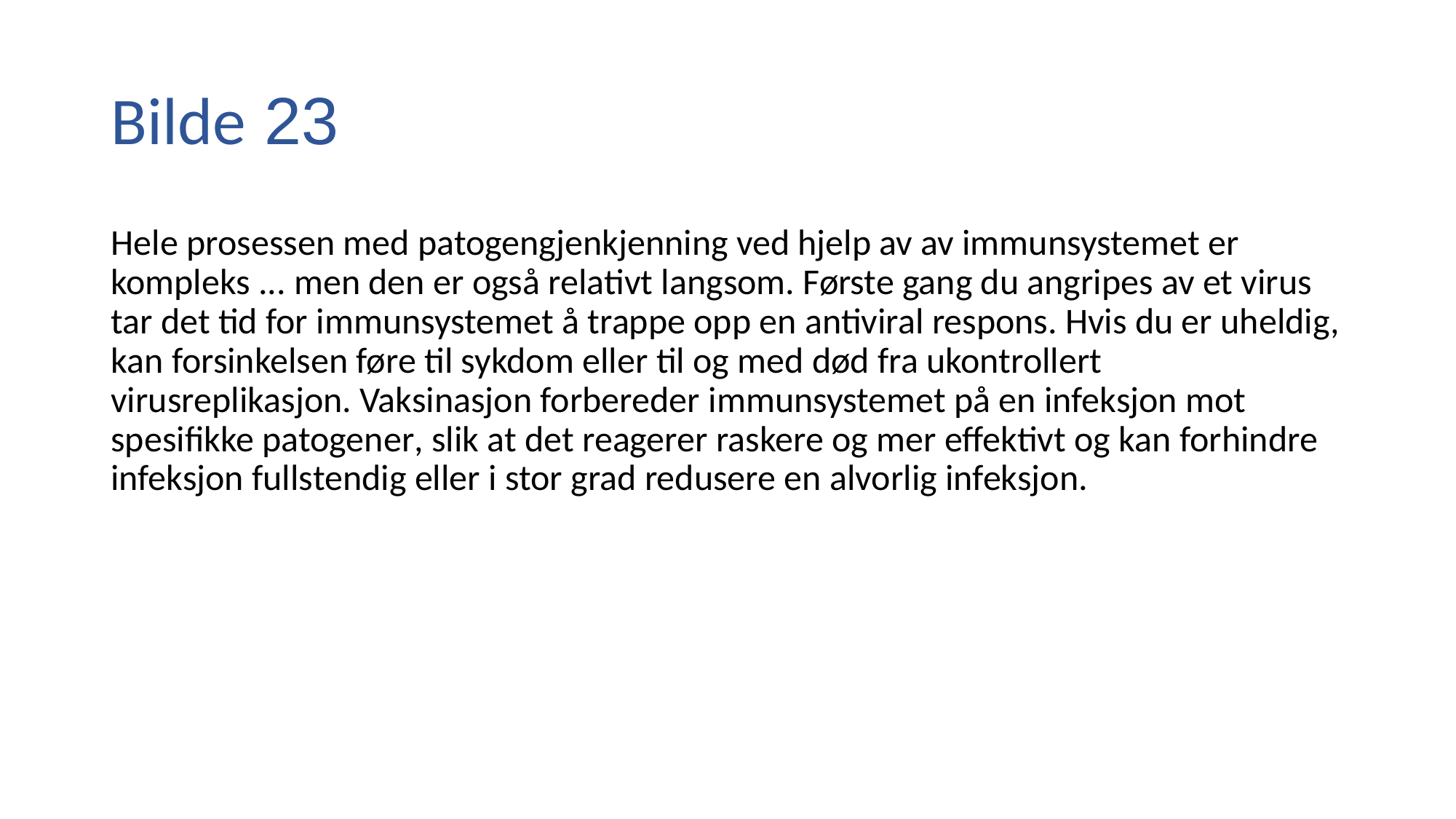

# Bilde 23
Hele prosessen med patogengjenkjenning ved hjelp av av immunsystemet er kompleks ... men den er også relativt langsom. Første gang du angripes av et virus tar det tid for immunsystemet å trappe opp en antiviral respons. Hvis du er uheldig, kan forsinkelsen føre til sykdom eller til og med død fra ukontrollert virusreplikasjon. Vaksinasjon forbereder immunsystemet på en infeksjon mot spesifikke patogener, slik at det reagerer raskere og mer effektivt og kan forhindre infeksjon fullstendig eller i stor grad redusere en alvorlig infeksjon.

## Slide 25
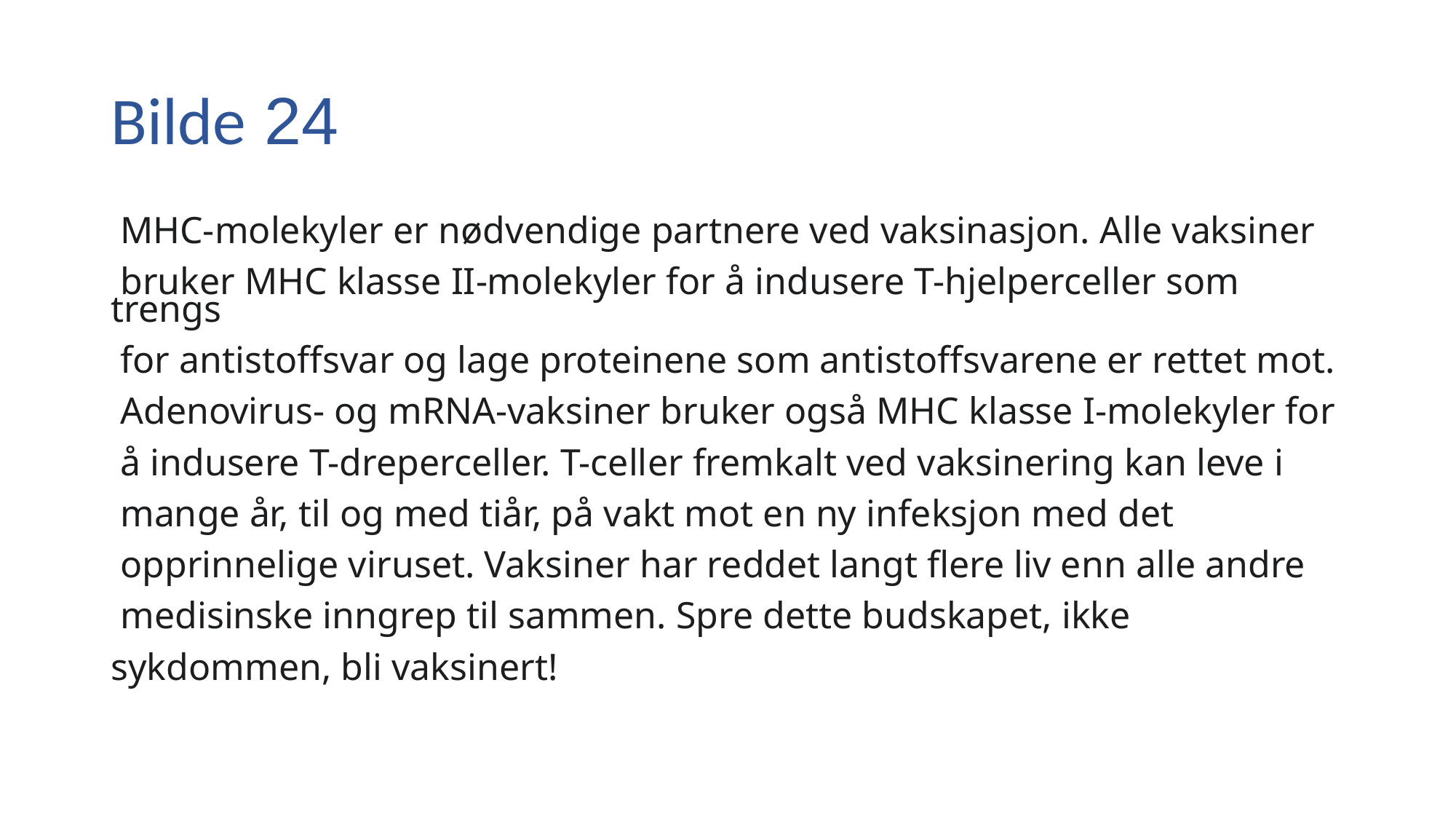

# Bilde 24
 MHC-molekyler er nødvendige partnere ved vaksinasjon. Alle vaksiner
 bruker MHC klasse II-molekyler for å indusere T-hjelperceller som trengs
 for antistoffsvar og lage proteinene som antistoffsvarene er rettet mot.
 Adenovirus- og mRNA-vaksiner bruker også MHC klasse I-molekyler for
 å indusere T-dreperceller. T-celler fremkalt ved vaksinering kan leve i
 mange år, til og med tiår, på vakt mot en ny infeksjon med det
 opprinnelige viruset. Vaksiner har reddet langt flere liv enn alle andre
 medisinske inngrep til sammen. Spre dette budskapet, ikke
sykdommen, bli vaksinert!

## Slide 26
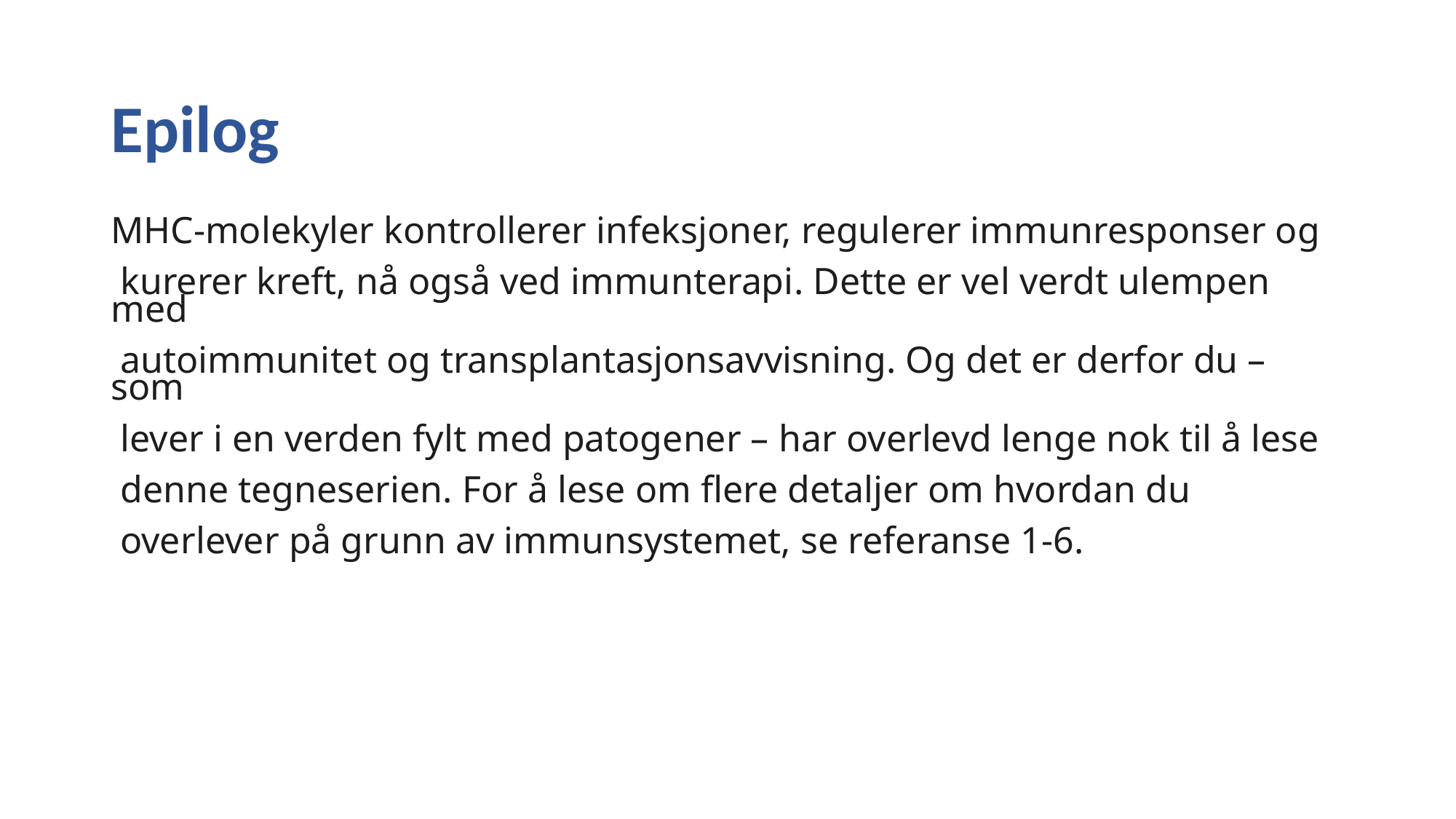

# Epilog
MHC-molekyler kontrollerer infeksjoner, regulerer immunresponser og
 kurerer kreft, nå også ved immunterapi. Dette er vel verdt ulempen med
 autoimmunitet og transplantasjonsavvisning. Og det er derfor du – som
 lever i en verden fylt med patogener – har overlevd lenge nok til å lese
 denne tegneserien. For å lese om flere detaljer om hvordan du
 overlever på grunn av immunsystemet, se referanse 1-6.
