# Supplementary material for: HLA molecules in transplantation, autoimmunity and infection control: A comic book adventure
Source: HLA. 2022 May 15;100(4):301–11. doi: 10.1111/tan.14626 (PMC9545814; doi:10.1111/tan.14626)
Supplement: Supplementary file 1 — Supporting information. [file TAN-100-301-s001.zip › Supplementary files/PP_Polish_Niemczura.1.pdf]

Rola białek układu HLA w transplantologii, zwalczaniu infekcji i rozwoju chorób autoimmunologicznych

HLA molecules in transplantation, autoimmunity and infection control.
A comic Book adventure

By Eric Reits and Jacques Neefjes

Translated by Magdalena Joanna Niemczura. Original text : ***https://doi.org/10.1111/tan.14626***

Department of Cell and Chemical Biology, ONCODE Institute, Leiden University Medical Centre LUMC, The Netherlands

1. SLAJD

Jakieś 1900 lat temu dwóch lekarzy, bracia Kosma i Damian, przeprowadziło pierwszą w historii transplantację. Odcięli oni zajętą gangreną nogę pewnego kupca i na jej miejsce przyszyli nogę jego niewolnika. Los niewolnika pozostaje nieznany, jednakże mało prawdopodobne, że był dobrowolnym dawcą

2. SLAJD

Cała operacja została uznana za „cud”, a obaj bracia zostali świętymi patronami transplantacji. Na ich korzyść w procesie beatyfikacji zadziałał również fakt, że zostali oni ścięci za wyznawanie wiary Chrześcijańskiej.

3. SLAJD

Dlaczego przeszczepy są tak trudne? Co w procesie ewolucji na to wpłynęło? Nawet Darwin pewnie się nad tym zastanawiał. A zastanawiał się dlatego, że nie wiedział o unikalnej klasie białek, które są obecne w niemal każdej komórce eukariotycznej.

4. SLAJD

Współcześnie wiemy, że w naszym organizmie znajdują się dwie klasy białek o wysokim stopniu polimorfizmu (to znaczy, że mocno się one różnią pomiędzy osobnikami). Czyni je to wyjątkowymi, ponieważ prawie wszystkie inne ludzkie białka są bardzo podobne pomiędzy poszczególnymi osobami. Te polimorficzne białka to antygeny zgodności tkankowej, nazywane MHC klasy I i MHC klasy II, a bardziej po ludzku: HLA I i HLA II.

5. SLAJD

Najważniejsze podczas przeszczepu są HLA-A, HLA-B z grupy białek MHC klasy I i HLA-DR, HLA-DQ i HLA-DP z grupy MHC kasy II. HLA-A, -B i –C są obecne praktycznie w każdej komórce organizmu (oprócz czerwonych krwinek), podczas gdy HLA-DR, HLA-DQ i HLA-DP znajdują się głównie na komórkach układu odpornościowego.

6. SLAJD

Polimorfizm antygenów zgodności tkankowej HLA jest tak wysoki, że kobiety w ciąży czasami wytwarzają przeciwciała przeciwko antygenom HLA ojca dziecka. Zjawisko to było wykorzystywane do określania ojcostwa w czasach kiedy nie słyszano jeszcze o testach genetycznych. Osocze pobrane od kobiet w ciąży było również używane przy przeszczepach tkanek. Różne laboratoria wymieniały próbki osocza od kobiet w ciąży między sobą nawzajem, opisując reakcje układu odpornościowego na poszczególne próbki. W ten sposób zidentyfikowano antygeny HLA-AA, -B i -C i ich podrodzaje. Zamiast oddzielnych nazw, nadano im po prostu kolejne numery, HLA-A1, HLA-A2 i tak dalej. Tak samo zrobiono z HLA-DR, -DQ i DP. Dlatego w twoich tkankach możesz mieć, np. HLA-A1, - B8, -Cw7, -DR3, -DQ2 i - DPw1 od twojej mamy i HLA-A2, -B27, -Cw1, -DR4, -DQ3 i DPw4 od twojego taty.

7. SLAJD

Dziś fenotypowanie pod kątem HLA wykonuje się poprzez analizę DNA. Istnieją dane mówiące, że kobiety są w stanie wyczuwać odmienne typy antygenów HLA u mężczyzn, co może podświadomie wpływać na wybór partnerów jak najbardziej różnych genetycznie od nich samych.

8. SLAJD

Polimorfizmy pomiędzy antygenami HLA zapewniają różnorodność wśród ludzi, jednak znacząco obniżają szanse przyjęcia się przeszczepu skoro potencjalny dawca jest tak różny od nas samych. Dlatego też tak ważne jest aby tak dopasować dawcę i biorcę pod względem antygenów HLA aby byli genetycznie tak blisko jak to tylko możliwe. Gdy znalezienie idealnego dawcy zawodzi, u biorcy stosuje się leki immunosupresyjne aby zminimalizować prawdopodobieństwo odrzucenia przeszczepu.

9. SLAJD

Darwin byłby zdezorientowany. Przecież wąchanie swojego potencjalnego partnera, zapobieganie odrzuceniu przeszczepów czy ustalenie ojcostwa nie może być głównym powodem powstania polimorfizmów w antygenach HLA.

10. SLAJD

I miałby rację. Istnieje jeszcze jeden bardzo wazny powód - wirusy i inne naturalnie wystepujące patogeny. Koronawirusy, grypa, ebola, ospa prawdziwa i wiele innych wirusów wykorzystują nasze komórki jako gospodarzy i używają naszej maszynerii komórkowej do wytwarzania kopii samych siebie. Nawet infekcje, które kończą się samoistnie byłyby zagrożeniem dla organizmu gdyby nie układ odpornościowy. Co nasuwa bardzo proste pytanie – jak układ odpornościowy rozpoznaje patogeny próbujące dostać się do organizmu zanim zdążą one wysłać nas na tamten świat?

11. SLAJD

Aby ograniczyć szkody, które w organizmie mogłyby wyrządzić wirusy, układ odpornościowy nabył drogą ewolucji kilka skutecznych typów broni. Makrofagi zjadają patogeny, neutrofile wypuszczają zabójcze dla mikrobów substancje, limfocyty B wytwarzają przeciwciała, a limfocyty T cytotoksyczne eliminują zainfekowane komórki (a nawet są w stanie wykryć i zniszczyć komórki rakowe).

12. SLAJD

Skąd taki limfocyt T wie którą komórkę zniszczyć? Przecież wirus, który sobie wesoło siedzi w komórce, jest dla nich niewidoczny, przykryty błoną komórkowa jak kołderką. Otóż wręcz przeciwnie – podczas gdy wirus beztrosko namnaża się w komórce, antygeny HLA-A, -B lub - C wychwytują fragmenty jego białek i niosą je na powierzchnię błony komórkowej. Widząc to, limfocyty T cytotoksyczne już wiedzą co mają dalej robić. Mechanizm ten jest tak specyficzny, że limfocyty T są w stanie rozpoznać tylko jeden konkretny typ antygenu układu MHC. Zjawisko to zostało nazwane restrykcją MHC i za jego odkrycie R.M. Zinkernagel i P.C. Doherty otrzymali w 1996 Nagrodę Nobla. Każdy z typów cząsteczek układu MHC klasy I ma swoisty repertuar wrogich peptydów, które są w stanie rozpoznać, co daje układowi odpornościowemu szeroki wachlarz potencjalnych celów.

13. SLAJD

Skąd jednak biorą się w komórce peptydy specyficzne dla wirusa? Otóż białka wirusowe, tak jak każde inne, kiedy są już bardzo stare i wypełnią swoje zadanie, są w komórce degradowane. Są cięte na kawałeczki przez maszynerię zwaną proteasomem, czyli taki komórkowy odpowiednik śmieciarki. Po przejściu przez proteasom, peptydy są dodatkowo atakowane przez różnorodne enzymy, które tu sobie coś odetną, tam pokroją, aż z białka zostają malutkie fragmenty, które są później transportowane do siateczki śródplazmatycznej, gdzie wyłapują je cząsteczki układu HLA. Następnie kompleks HLA + wirusowy peptyd zostaje przetransportowany na powierzchnię błony komórkowej, gdzie limfocyty T już czekają aby się nim zająć.

14. SLAJD

Wróćmy do polimorfizmów w układzie HLA. Jak wszyscy dobrze wiemy z epidemii covid-19 czy grypy, wirusy są doskonałe w unikaniu odpowiedzi immunologicznej organizmu dzięki umiejętności wprowadzania zmian we własnym materiale genetycznym (stąd warianty alfa, delta, omicron). Aby zminimalizować to ryzyko, każdy z alleli w białkach układu MHC jest w stanie prezentować inny rodzaj peptydów wirusowych. Liczba możliwych peptydów rozpoznawanych przez jednego człowieka jest tak duża, że wirusowi bardzo trudno jest się schować przed odpowiedzią immunologiczną. Jeśli jednak mu się to uda u jednej osoby, dzięki polimorfizmom pomiędzy białkami HLA, w organizmie innej zostanie wykryty. Gdyby ludzie byli bardziej jednorodni pod względem białek HLA, jeden wirus mógłby spowodować wymarcie całej populacji zamiast śmierci zaledwie kilku osobników, których białka HLA nie wykryły wirusa. Stąd można wysunąć hipotezę, że wysoki polimorfizm między białkami układu HLA u poszczególnych ludzi chroni populację, nie jednostkę.

15. SLAJD

Lecz niestety – mamy też złe wieści dla Ciebie, czytelniku, gdybyś potrzebował wymiany jakiegoś organu lub dwóch. HLA promuje przeżycie populacji, a niekoniecznie jednostki z, powiedzmy, rzadką chorobą nerek. Odrzucenie przeszczepu to nic innego jak tylko rozpoznawanie przez komórki organizmu organu dawcy jako coś wrogiego co trzeba zniszczyć.

16. SLAJD

Można stąd wyciągnąć ważną lekcję – nic, włączając ludzki układ odpornościowy, nie jest doskonałe. Ale jeśli już o układzie odpornościowym mowa, jak to się dzieje, że limfocyty T przybywają na miejsce infekcji na czas? Przecież wirusy namnażają się bardzo szybko, w niektórych przypadkach wystarcza im kilka godzin aby wyprodukować wiele kopii samych siebie. Organizm nie może sobie pozwolić na czekanie aż białka wirusowe się zestarzeją i zostaną zdegradowane. Z pomocą przychodzi tu fakt, że synteza białek, zupełnie tak jak układ odpornościowy, nie jest doskonała. Owe niedoskonałe białka, które zostały źle zsyntetyzowane, zwane DRiP, są natychmiast niszczone, uruchamiając mechanizm prezentacji peptydów wirusowych na powierzchni komórki przez białka układu HLA.

17. SLAJD

Gem, set, mecz dla układu odpornościowego? Niestety nie tak szybko. Niektóre wirusy, szczególnie z rodziny herpesviridae, są sprytne i wytworzyły mechanizm zakłócający prezentację antygenu przez układ HLA. Ludzki cytomegalowirus HCMV zamieszkujący 60% ludzkiej populacji stworzył pakiet białek (US2, US3, US6, US11, i US18), które ograniczają produkcję peptydów, bądź też zaburzają funkcjonowanie białek HLA klasy I.

18. SLAJD

Czy możliwym jest więc, żeby niektóre allele białek układu HLA były lepsze w radzeniu sobie z cząsteczkami wirusowymi niż inne? Owszem, niektóre allele HLA-B chronią lepiej przed HIV, a inne są lepsze w zwalczaniu COVID. Na drodze ewolucji poszczególne allele wyspecjalizowały się aby zwalczać specyficzny rodzaj wroga. Weźmy HLA-A2 jako przykład – jest on obecny u 40% Europejczyków, co sugeruje, że w pewnym momencie historii był on w stanie obronić posiadacza przed specyficzną chorobą, która eliminowała tych, który go nie mieli.

19. SLAJD

Oczywiście są też mniej przyjemne konsekwencje, choćby jak w przypadku allelu HLA-B*27:05. Obecny jest on u 8% osobników rasy białej, i... u 90% ludzi cierpiących na zesztywniające zapalenie stawów kręgosłupa – chorobę autoimmunologiczną objawiającą się tym, że limfocyty T chorego atakują jego kręgosłup. Układ odpornościowy zatem niczym linoskoczek na linie ciągle balansuje pomiędzy zapewnieniem organizmowi jak najlepszej ochrony a nieatakowaniem przez przypadek własnych tkanek.

20. SLAJD

Co ciekawe, autoagresja limfocytów T również może być dla organizmu korzystna. Komórki nowotworowe zazwyczaj nagromadzają taką ilość mutacji w swoim genomie, że wytwarzane przez nie peptydy są znacząco różne od typowych białek organizmu. Dziedzina nauki zwana immunoterapią nowotworową wykorzystuje ten mechanizm do ich zwalczania.

21. SLAJD

Ale co z innymi klasami białek układu HLA? HLA-DR, -DQ i-DP, czyli białka MHC klasy drugiej, specjalizują się w prezentacji antygenów limfocytom T pomocniczym, które następnie wytwarzają cytokiny, które dają znać limfocytom B, że trzeba wyprodukować przeciwciała. Limfocyty T pomocnicze zajmują się też optymalizacją odpowiedzi limfocytów T cytotoksycznych. Białka MHC klasy drugiej są bardzo podobne w budowie do tych klasy I, jednak różnią się tym, że potrafią prezentować dłuższe peptydy pochodzące z lizosomów (małych organelli degradujących białka, które komórka wchłonęła z zewnątrz), a nie z peroksysomów, jak MHC klasy I.

22. SLAJD

Jak to robią? MHC klasy II są wytwarzane na siateczce śródplazmatycznej (jak każde inne białka, które potem dostają sygnał aby migrować na powierzchnię błony komórkowej bądź do lizosomów), gdzie wiąże specyficzne białko, które odprowadza MHC klasy II do lizosomu. Tam białko to odłącza się, a do MHC II przyłącza się kolejne bialko, tym razem pochodzące z lizosomu. Tym procesem zarządza kolejny typ białek MHC (HLA-DM, który wyglądem przypomina MHC klasy II i w niektórych komórkach współpracuje z HLA-DO, kolejnym białkiem podobnym do MHC II. Ewolucja jest leniwym stworzeniem i jeśli już stworzy coś, co działa, będzie ten mechanizm kopiować i znajdować mu nowe zastosowania gdzie to tylko możliwe). W konsekwencji tego zawiłego tańca MHC klasy II zostaje dostarczone na powierzchnię błony komórkowej razem z peptydami umożliwiającymi aktywację limfocytów T pomocniczych.

23. SLAJD

Proces rozpoznawania patogenu przez układ odpornościowy jest skomplikowany i... dość powolny. Kiedy po raz pierwszy zetkniesz się z wirusem, układ odpornościowy nie spieszy się za bardzo aby wysłać kogoś na miejsce infekcji by sprawdził co się dzieje. Jeśli masz pecha, może to skutkować chorobą bądź nawet śmiercią. Szczepionki przygotowują układ odpornościowy na potencjalną infekcję, co w niektórych przypadkach pozwala całkowicie jej uniknąć, bądź też umożliwić szybszą odpowiedź immunologiczną, przez co przechorowanie infekcji nie jest dla organizmu aż tak dotkliwe.

24. SLAJD

Białka układu MHC są kluczowe w procesie nabywania odporności poprzez szczepienie. Wszystkie szczepionki wykorzystują MHC klasy II aby aktywować limfocyty T pomocnicze potrzebne do wytworzenia przeciwciał przeciwko patogenowi. Szczepionki adenowirusowe i mRNA również wykorzystują MHC, tym razem klasy I, do aktywowania limfocytów T cytotoksycznych. Limfocyty T aktywowane przez szczepionki pozostają w organizmie przez długi czas, w niektórych przypadkach nawet kilkadziesiąt lat, i tylko czekają na patogen aby go zniszczyć. Szczepienia ocaliły życie większej ilości ludzi niż wszystkie inne terapie razem wzięte. Dlatego tak ważne jest by się szczepić!

25. EPILOG

Podsumowując, miałka MHC zapobiegają infekcjom, regulują odpowiedź immunologiczną, a nawet pomagają w leczeniu nowotworów. Cena jaką musimy za to zapłacić, w postaci okazjonalnej autoagresji układu w stosunku do organizmu czy możliwości odrzucenia przeszczepu wydaje się relatywnie niska. Jest to bardzo prawdopodobne, że to właśnie dzięki układowi MHC byłeś w stanie przetrwać w świecie pełnym patogenów wystarczająco długo by przeczytać ten komiks! Po więcej wskazówek jak najlepiej przetrwać, odsyłamy do przypisów 1-6.
