# Supplementary material for: HLA molecules in transplantation, autoimmunity and infection control: A comic book adventure
Source: HLA. 2022 May 15;100(4):301–11. doi: 10.1111/tan.14626 (PMC9545814; doi:10.1111/tan.14626)
Supplement: Supplementary file 1 — Supporting information. [file TAN-100-301-s001.zip › Supplementary files/PP_Portuguese_Pedroso_Neves Costa.1.pdf]

O papel das moléculas HLA nos transplantes, na autoimunidade e no controlo de infeções. Uma aventura em banda desenhada.

HLA molecules in transplantation, autoimmunity and infection control.
A comic Book adventure

by Eric Reits and Jacques Neefjes

Translated by Dora Pedroso and Ana Neves-Costa. Original text : ***https://doi.org/10.1111/tan.14626***

Department of Cell and Chemical Biology, ONCODE Institute, Leiden University Medical Centre LUMC, The Netherlands

Slide 1

Há cerca de 1900 anos, dois médicos Árabes, os irmãos Cosme e Damião, realizaram o primeiro transplante de que há conhecimento. A perna gangrenosa de um comerciante foi substituída pela perna de um seu escravo, numa doação muito provavelmente involuntária, ainda que desse escravo não reze a história.

Slide 2

Este “milagre” contribuiu para que se tornassem os Santos Padroeiros dos transplantes. Para a beatificação dos irmãos, também terá ajudado terem sido decapitados por seguirem a fé Cristã. Após ascensão ao Céu, assume-se que as suas cabeças tenham sido reposicionadas.

Slide 3

Porque são os transplantes tão difíceis e qual o papel da evolução? Até Darwin se deve ter questionado... mas ele desconhecia uma classe de proteínas muito especiais que são expressas em quase todos os organismos eucariontes multicelulares.

Slide 4

Comecemos por tentar compreender duas classes de proteínas presentes no nosso corpo e que se destacam por serem muito polimórficas (ou seja, diferem consideravelmente de pessoa para pessoa). Esta característica é incomum, já que a maioria das proteínas são quase idênticas entre indivíduos. Estas proteínas polimórficas são consideradas “antigénios de compatibilidade” e são chamadas moléculas MHC classe I e MHC classe II. Nos seres humanos em particular, denominam-se HLA classe I e HLA classe II.

Slide 5

Nos transplantes, as moléculas HLA mais relevantes são as HLA-A, HLA-B e HLA-C dentro da classe I, e HLA-DR, HLA-DQ e HLA-DP dentro da classe II. Enquanto que as moléculas HLA-A, -B e -C estão em todas as nossas células (a única exceção são os glóbulos vermelhos), as HLA-DR, -DQ e -DP encontram-se principalmente em células do sistema imunitário.

Slide 6

As moléculas HLA são tão polimórficas que é frequente as grávidas produzirem anticorpos contra os diferentes tipos de HLA do pai do bebé. Antes dos testes genéticos, estes anticorpos eram utilizados em testes de paternidade. O soro de mulheres grávidas era também usado em transplantes e partilhavam-se soros entre diferentes laboratórios com vista a estabelecer os diferentes tipos de respostas geradas. Foi assim que se identificaram as moléculas HLA-A, -B e -C, assim como as suas variantes, que se passaram simplesmente a chamar HLA-A1, depois HLA-A2, etc. O mesmo aconteceu para as HLA-DR, -DQ e -DP. Assim, as nossas células podem ter, por exemplo, proteínas HLA-A1, -B8, -Cw7, -DR3, -DQ2 e DPw1 herdadas da nossa mãe, mas também HLA-A2, -B27, -Cw1, -DR4, -DQ3 e DPw4 herdadas do nosso pai.

Slide 7

Hoje em dia, a genotipagem de HLA é um teste de rotina quando se fazem análises genéticas ao ADN. Existem também evidências de que algumas mulheres conseguem detetar o tipo de HLA de um homem pelo cheiro, o que poderá contribuir para a escolha de parceiros geneticamente diferentes.

Slide 8

Se por um lado os polimorfismos das proteínas HLA ajudam a diversificar a população humana, são também uma enorme barreira ao transplante de órgãos, já que é necessário assegurar o máximo de correspondência possível entre dador e recetor. Quando não existe coincidência perfeita, recorre-se a medicamentos imunossupressores para evitar a rejeição do transplante.

Slide 9

Darwin teria ficado intrigado; decerto que cheirar o parceiro ideal, impedir o transplante de órgãos ou desvendar a paternidade não são razões suficientes para a evolução ter aperfeiçoado o polimorfismo das moléculas HLA.

Slide 10

Mas há outras razões: vírus e outros micróbios patogénicos abundam na natureza. Corona, Influenza, Ébola, Varíola e muitos outros vírus utilizam as nossas células para se multiplicarem. Mesmo as infeções “auto-limitadas” seriam letais se não tivéssemos sistema imunitário. Então, como é que o sistema imunitário consegue detetar a presença de vírus quando estes estão escondidos dentro das nossas células, de modo a poder matá-los antes que eles nos matem a nós?

Slide 11

O sistema imunitário desenvolveu variadas armas para limitar os estragos causados pelos vírus: os macrófagos comem bactérias e vírus; os neutrófilos libertam substâncias tóxicas para as bactérias; os linfócitos B produzem anticorpos; os linfócitos T-auxiliares ajudam os linfócitos B e outras células; e os T-citotóxicos matam células infetadas por vírus (e podem mesmo matar células cancerosas).

Slide 12

Como é que os linfócitos T-citotóxicos sabem quem matar se dentro das células os vírus estão a salvo - ou não estarão? Na verdade, à medida que os vírus se replicam, pequenos fragmentos de proteínas virais são transferidos para as moléculas HLA-A, -B ou -C, que por sua vez os transportam para a superfície da célula. Os linfócitos T-citotóxicos reconhecem estes péptidos em complexo com UMA molécula HLA específica. A este fenómeno chama-se restrição HLA e a sua descoberta rendeu dois prémios Nobel. Cada tipo de molécula MHC classe I apresenta um repertório distinto de péptidos, o que resulta numa imensidão de alvos para reconhecimento pelos linfócitos T, que podem assim destruir as células que os apresentam.

•

Slide 13

Mas como é que um fragmento viral é gerado? As proteínas virais sofrem degradação, à semelhança de qualquer outra proteína no interior das células. A fragmentação das proteínas realiza-se por intermédio de uma nano-máquina notável, o proteassoma, que é basicamente um EliminaTudo para proteínas. Outras enzimas celulares aparam as extremidades dos fragmentos, originando péptidos menores, alguns dos quais são transportados do citosol para o retículo endoplasmático (RE), onde se podem ligar a moléculas HLA. Assim que uma molécula HLA se liga a um péptido, ela sai do RE e dirige-se à superfície celular, onde aguarda deteção por linfócitos T-citotóxicos.

Slide 14

Voltemos ao polimorfismo HLA. Como é sabido de COVID-19 e Influenza, os vírus são excelentes a mudar para escapar à resposta dos anticorpos (pense em alfa, delta, ómicron…). Para minimizar esta possibilidade para os linfócitos T, cada um dos diferentes alelos (variantes de genes) MHC apresenta um conjunto diferente de péptidos. Em cada pessoa, há tantos péptidos apresentados que a evasão do vírus se torna difícil. Existem tantos tipos de HLA diferentes que, mesmo que um vírus se evada, não conseguirá manter o seu embuste na pessoa seguinte. Se fôssemos todos idênticos quanto a HLA, um vírus que escapasse mataria toda a população, mas assim matará "apenas" alguns indivíduos com moléculas HLA incapazes de apresentar péptidos virais ao sistema imunitário. O polimorfismo HLA protege, portanto, a população, sendo cada indivíduo menos importante. Assim se explica como este polimorfismo terá evoluído.

Slide 15

Mas infelizmente, más notícias para si, caro leitor, se precisar de um ou dois órgãos novos. O polimorfismo HLA promove a sobrevivência de uma população, não de um individuo com doença renal. A rejeição de um transplante é consequência do sistema imunitário confundir um órgão de um dador com um órgão infetado por um vírus e responder em concordância, atacando o órgão, o que resulta na rejeição do transplante.

Slide 16

Uma lição importante: nada é perfeito, incluindo o sistema imunitário! A propósito, pensemos como os linfócitos T-citotóxicos conseguem encontrar células infetadas por vírus suficientemente depressa para haver alguma utilidade. Os vírus podem produzir a sua descendência muito depressa, em certos casos em algumas horas apenas. Esperar que as proteínas virais fossem degradadas levaria demasiado tempo. Mas, à semelhança do próprio sistema imunitário, a síntese de proteínas, incluindo as proteínas virais, está longe da perfeição. Estas proteínas imperfeitas, chamadas DRiPs, são degradadas de imediato, associando o início da infeção viral à apresentação de antigénios e permitindo uma eficiente imunovigilância pelos linfócitos T-citotóxicos.

Slide 17

Xeque-mate, sistema imunitário? Não tão depressa! Alguns vírus espertos, especialmente os herpesvírus, evoluíram de forma a interferirem com a apresentação de antigénios. O citomegalovírus humano, HCMV, que infeta 60% da humanidade, produz uma gama de proteínas (US2, US3, US6, US11 e US18) que limita a produção de péptidos ou interfere com a função de moléculas HLA classe I.

Slide 18

Será então possível que alguns alelos HLA sejam mais eficientes a lidar com infeções virais que outros? Com efeito, alguns alelos HLA-B protegem melhor do HIV, outros são melhores para Covid. Os diferentes alelos HLA foram selecionados durante eões para lidar com diferentes agentes patogénicos. Por exemplo, o HLA-A2 está presente em 40% da população Europeia, sendo esta a prevalência mais elevada de qualquer alelo HLA em determinado grupo. Isto resulta provavelmente da capacidade do HLA-A2 de proteger, a dada altura no passado, de um agente patogénico que pode muito bem já não ser atualmente uma causa importante de doença humana.

Slide 19

Mas não esqueçamos os danos colaterais! Consideremos o alelo HLA-B*27:05. Encontra-se presente em 8% da população Caucasiana e mais de 90% dos pacientes com espondilite anquilosante possuem este alelo, que provavelmente desencadeia uma reação autoimune dos linfócitos T na coluna vertebral. O sistema imunitário está sobre o fio da navalha, assegurando imunidade eficaz sem lesar os órgãos com o seu fogo amigável.

Slide 20

A autoimunidade dos linfócitos T pode também ser benéfica. As células cancerosas geralmente possuem muitas mutações e outras alterações conducentes à produção de péptidos diferentes dos péptidos celulares normais. A imunoterapia do cancro explora mecanismos usados pelo sistema imunitário no reconhecimento de infeções virais e bacterianas para matar células cancerosas.

Slide 21

Mas então e as moléculas MHC classe II HLA-DR, -DQ e -DP? Estas moléculas apresentam péptidos que são patogénicos para os linfócitos T-auxiliares, que por sua vez produzem citocinas para ajudarem os linfócitos B a se diferenciarem em fábricas produtoras de anticorpos. Os linfócitos T-auxiliares também ajudam a otimizar as respostas T-citotóxicas. As moléculas MHC classe II são muito similares em formato às MHC classe I mas apresentam fragmentos de proteína que são mais longos e produzidos nos lisossomas, que são pequenos organelos que degradam proteínas adquiridas do exterior das células.

Slide 22

Como é que que elas fazem isto? As moléculas MHC classe II são produzidas no RE (tal como qualquer outra proteína que tenha que se dirigir para a membrana celular ou para os lisossomas), onde se ligam a uma proteína (de cadeia invariável) que se assemelha a um péptido e acompanha a molécula MHC classe II ao lisossoma. Aqui, a cadeia invariável é removida e substituída por um péptido produzido por enzimas lisossomais. Este processo é otimizado por meio de um outro tipo de molécula MHC (HLA-DM), que se assemelha a MHC classe II e que em algumas células funciona em concertação com HLA-DO, outra molécula análoga às de classe II. A evolução é preguiçosa e, depois de desenvolver um módulo funcional, simplesmente copia-o e modifica-o para novas funções. Esta dança complicada culmina com o transporte para a superfície celular de moléculas MHC classe II com péptidos que permitem a ativação de linfócitos T-auxiliares.

Slide 23

Este processo de reconhecimento de agentes patogénicos pelo sistema imunitário é complexo…mas também é relativamente lento. Na primeira vez que encontramos um vírus, o sistema imunitário demora a intensificar a resposta anti-viral. Se tivermos azar, isto pode resultar em doença ou morte por replicação viral descontrolada. A vacinação prepara o sistema imunitário para uma infeção, permitindo que em certos casos evite por completo a infeção, ou que responda mais rápida e eficientemente, reduzindo assim bastante a possibilidade de uma infeção severa.

Slide 24

As moléculas MHC são críticas na vacinação. Todas as vacinas recorrem a moléculas MHC classe II para induzir linfócitos T-auxiliares necessários a respostas de anticorpos e para produzir as proteínas contra as quais estas respostas são dirigidas. As vacinas de adenovírus e mRNA também utilizam moléculas MHC classe I para induzir linfócitos T-citotóxicos. Os linfócitos T induzidos pelas vacinas persistem por muitos anos, décadas em alguns casos, em alerta para uma nova infeção pelo vírus original. As vacinas têm salvo muito mais vidas que quaisquer outras intervenções médicas combinadas. Espalhe esta mensagem, não a doença, vacine-se!

EPÍLOGO

Então, as moléculas MHC controlam infeções, regulam as respostas imunitárias e estão agora a ajudar a curar o cancro, o que compensa largamente as desvantagens da autoimunidade e de rejeição de transplantes. E é por isto que você – vivendo num mundo repleto de agentes patogénicos – sobreviveu para ler esta banda desenhada. Para mais pormenores de como sobreviver ainda melhor, por favor veja as referências 1-6.
