# Supplementary material for: HLA molecules in transplantation, autoimmunity and infection control: A comic book adventure
Source: HLA. 2022 May 15;100(4):301–11. doi: 10.1111/tan.14626 (PMC9545814; doi:10.1111/tan.14626)
Supplement: Supplementary file 1 — Supporting information. [file TAN-100-301-s001.zip › Supplementary files/PP_Spanish_Pérez Berrocal_1.pdf]

Moléculas HLA en trasplantes, autoinmunidad y control de infecciones: Una aventura animada

HLA molecules in transplantation, autoimmunity and infection control.
A comic Book adventure

by Eric Reits and Jacques Neefjes

Translated by David Aureliano Pérez Berrocal. *Original text :* ***https://doi.org/10.1111/tan.14626***

Department of Cell and Chemical Biology, ONCODE Institute, Leiden University Medical Centre LUMC, The Netherlands

Diapositiva 1

Hace unos 1900 años, los dos hermanos médicos árabes, Cosmas y Damianus realizaron el primer trasplante conocido, cambiando la pierna gangrenosa de un mercante con la de su esclavo. La suerte del esclavo es desconocida en la historia, pero seguramente no fue una donación voluntaria.

Diapositiva 2

El trasplante “milagroso” contribuyó a su beatificación, llevándolo a convertirse al Santo Patrón de los trasplantes. No dolió que fueran decapitados debido a su fe cristiana, ya que esto se corregiría al ascender a los cielos.

Diapositiva 3

¿Por qué los trasplantes son tan difíciles de realizar, cuáles son los factores evolutivos? Incluso Darwin debió haberse preguntado...pero él no conocía una clase única de proteínas que están expresadas por casi todos los organismos eucariotas multicelulares.

Diapositiva 4

Comencemos con la comprensión actual de dos clases de proteínas únicas en nuestro cuerpo; las que tienen el mayor grado de polimorfismo (diferencias entre individuos). Y estas son únicas al igual que casi todas las demás proteínas son casi idénticas entre los humanos. Estas proteínas polimórficas son los "antígenos de trasplante" y se denominan generalmente como moléculas MHC de clase I y MHC de clase II. En humanos se llaman HLA de clase I y HLA de clase II.

Diapositiva 5

Las moléculas HLA más importantes para los trasplantes estan denominadas HLA-A, HLA-B y HLA-C para los MHC de clase I y HLA-DR, HLA-DQ y HLA-DP para los MHC de clase II. Las HLA-A, -B y -C están presentes en prácticamente todas nuestras células (excepto en los glóbulos rojos) mientras que las HLA-DR, HLA-DQ y HLA-DP se encuentran principalmente en las células inmunes.

Diapositiva 6

Las moléculas de HLA son tan polimórficas que las mujeres embarazadas a menudo producen anticuerpos contra los diferentes tipos de HLA del padre. Esto podía usarse para determinar quien era el padre en los tiempos previos a que las pruebas genéticas estuvieran disponibles. Pero estos sueros de mujeres embarazadas también fueron utilizadas para el trasplante de tejidos. En talleres, se intercambiaban sueros de estas mujeres entre los laboratorios y las diferentes respuestas serológicas nombradas en talleres HLA. Así es como HLA-A, -B y -C fueron identificadas y sus diferentes formas también. Estas fueron simplemente numeradas HLA-A1, la siguiente HLA-A2 y así sucesivamente. Esto también ocurrió con las moléculas HLA-DR, -DQ y -DP. Así que sus tejidos pueden tener (por ejemplo) proteínas HLA-A1, -B8, -Cw7,-DR3, -DQ2 y DPw1 de su madre y proteínas HLA-A2, -B27, -Cw1, -DR4, -DQ3 y DPw4 de su padre.

Diapositiva 7

Hoy en día, la tipificación de HLA se lleva a cabo de manera rutinaria mediante análisis de ADN. Hay algunas evidencias que las mujeres pueden detectar diferencias en las HLA masculinas a través del olfato, y que esto contribuye a la selección para parejas genéticamente diferentes.

Diapositiva 8

Mientras que el polimorfismo de HLA puede ayudar a la diversificación de la humanidad, es una inmensa barrera para el éxito de trasplantes de órganos, que requiere compatibilidad, tan similar como sea posible, entre los tipos de HLA del donante y el receptor. En la ausencia de tal compatibilidad, medicamentos inmunosupresores efectivos son usados para prevenir rechazo del órgano trasplantado.

Diapositiva 9

Darwin estaría desquiciado. Seguramente, oler a tu pareja perfecta, prevenir trasplantes de tejidos, o buscar al padre verdadero no puede ser la principal razón evolutiva para el polimorfismo de HLA.

Diapositiva 10

Pero hay otro factor: los virus y otros patógenos microbianos que abundan en la naturaleza. Corona, Gripe, Ébola, Viruela y otros muchos virus utilizan nuestras células para crear sus propias familias. Incluso infecciones autolimitadas serían letales sin un sistema inmune. Y la pregunta es bastante simple: ¿Cómo puede el sistema inmune detectar a los virus que acechan dentro de las células para matarlos antes de que ellos nos maten a nosotros?

Diapositiva 11

Para limitar el daño de los virus, el sistema inmune desarrolló evolutivamente múltiples armas. Los macrófagos se comen bacterias y virus, neutrófilos liberan substancias asesinas para bacterias, las células B producen anticuerpos, células T colaboradoras ayudan a las células B y otras más, y las células T citotóxicas matan a las células infectadas por virus (e incluso a células cancerígenas).

Diapositiva 12

¿Pero cómo una célula T citotóxica pueda saber a quién asesinar? El virus, al estar dentro de la célula, escapa a la detección, o ¿no es así? En efecto, cuando el virus se replica, pequeños trozos de sus proteínas son enviados a las moléculas HLA-A, -B, -C que las transportan hasta la superficie celular. La célula T citotóxica reconoce este pequeño fragmento en el contexto de una molécula específica HLA. El descubrimiento de este fenómeno, denominado restricción de HLA, fue suficientemente importante para obtener dos premios Nobel. Cada tipo diferente de moléculas MHC clase I presenta un repertorio diferente de péptidos para otorgar al sistema inmune numerosos objetivos para apuntar y matar las células que las producen.

Diapositiva 13

¿Pero cómo es generado el fragmento de un virus en primer lugar? Las proteínas virales al igual que cualquier proteína dentro de las células son degradadas. Las proteínas son fragmentadas por una increíble nano-máquina llamada el proteosoma, que es básicamente un contenedor para proteínas. Otras enzimas celulares cortan los extremos de los fragmentos en péptidos mas cortos, alguno de los cuales son transportados desde el citosol al Retículo Endoplasmático (RE) donde pueden unirse a moléculas HLA. Una vez una molécula de HLA tiene un péptido unido, esta abandona el RE- hacia la superficie celular donde espera ser detectada por células T citotóxicas.

Diapositiva 14

Vayamos de vuelta al polimorfismo de HLA. Como todos conocemos sobre el COVID-19 y la Gripe, los virus son muy buenos mutando para escapar la respuesta de anticuerpos(recordad la alpha, delta, ómicron…). Para minimizar esta posibilidad para las células T citotóxicas, cada uno de los diferentes alelos MHC (variedad de genes) presenta un conjunto diferente de péptidos. Si muchos péptidos son presentados en una persona, la evasión del virus es complicada. Las diferencias en los tipos de HLA entre personas significa que si incluso esto ocurre, el virus evasor no engañará a la siguiente persona. Si todos fuéramos idénticos en nuestros HLA, un virus evasor aniquilaría a la población entera, sino solo “mataría” a algunos individuos con moléculas HLA incapaces de presentar los péptidos virales al sistema inmune. El polimorfismo de HLA protege así a la población, mientras que es menos importante hacia el individuo. Esto provee una explicación convincente para la evolución del polimorfismo de MHC.

Diapositiva 15

Desafortunadamente querido lector, hay malas noticias para usted si usted necesita un órgano o dos. El polimorfismo de HLA promueve la supervivencia de la población de una especie, no al individuo con una enfermedad renal. Rechazo de un trasplante es la consecuencia del sistema inmune confundiendo el órgano donante con un órgano infectado por virus and responde adecuadamente mediante ataques al órgano resultando en rechazo del órgano trasplantado.

Diapositiva 16

Una lección general importante: ¡nada, incluyendo el sistema inmune es perfecto! Y hablando del cual, pensemos sobre como las células T citotóxicas pueden encontrar células infectadas víricamente de una manera suficientemente rápida, en algunos casos en tan solo algunas horas. Esto es demasiado lento para esperar a que las proteínas sean degradadas al final de su vida natural. Pero al igual que el sistema inmune, la síntesis de proteínas incluyendo proteínas virales, esta lejos de ser perfecta. Estas proteínas imperfectas, denominadas DRiPs, son degradadas inmediatamente, acoplando el comienzo de la infección vírica con la presentación antigénica y permitiendo una inmunovigilancia efectivas por parte de las células T citotóxicas.

Diapositiva 17

Jaque mate sistema inmune… ¡No tan rápido! Algunos virus inteligentes, especialmente virus del herpes, han evolucionado para interferir con la presentación de antígenos. Cytomegalovirus humano (HCMV), que infecta al 60% de la humanidad hace un conjunto de proteínas (US2, US3, US6, US11, and US18) que limita la producción de péptidos o interfiere con la función de HLA clase I.

Diapositiva 18

¿Es entonces posible qué algunos de los alelos de HLA son mejores en manejar infecciones virales que otros? En efecto, algunos alelos HLA-B protegen mejor contra el VIH, otros son mejores para el Covid. Los diferentes alelos HLA han sido seleccionados a través de eones para lidiar con diferentes patógenos. Por ejemplo, HLA-A2 se encuentra en el 40% de la población europea, la mayor prevalencia de todos los alelos HLA en cualquier grupo. Esto probablemente es consecuencia de la habilidad de HLA-A2 para proteger contra un patógeno en algún momento específico en el tiempo, que podría buenamente no ser una causa mayor de enfermedad a día de hoy.

Diapositiva 19

Pero hay consecuencias colaterales, atendiendo al alelo HLA-B*27:05, presente en el 8% de la población Caucásica, sobre el 90% de los pacientes de espondilitis anquilosante presentan este alelo, que seguramente produce una reacción autoinmune celular tipo T citotóxica en la espina dorsal. El sistema inmune opera en el filo de un cuchillo entre proveer una inmunidad efectiva sin dañar tejidos por fuego amigo.

Diapositiva 20

La autoinmunidad de las células T también puede ser beneficiosa. Las células cancerígenas típicamente tienen muchas mutaciones y otras alteraciones que pueden llevar a la generación de péptidos que difieren de péptidos de células normales. La inmunoterapia para combatir el cáncer suele explotar mecanismos usados por el sistema inmune reconociendo infecciones virales y bacterianas para matar a las células cancerígenas.

Diapositiva 21

¿Pero entonces qué ocurre con las moléculas MHC de clase II HLA-DR y -DP? Estas moléculas presentan péptidos de patógenos a las células T auxiliares que a su vez producen citoquinas para ayudar a la diferenciación de las células B hacia fábricas productoras de anticuerpos. Las moléculas MHC de clase II son extremadamente similares en forma a las de clase I pero presentan fragmentos proteicos que son más largos y generados en los lisosomas, que son orgánulos que degradan proteínas adquiridas del exterior de las células.

Diapositiva 22

¿Cómo ocurre esto? Las moléculas MHC de clase II son generadas en el RE(cómo cualquier otra proteína que tiene que ir al exterior de la membrana celular o a los lisosomas) donde se une a proteínas (cadena invariante) que simulan a un péptido y acompaña a moléculas MHC de clase II al lisosoma. Aquí, la cadena invariante es expulsada e intercambiada por un péptido creado por las enzimas lisosomales. Este proceso es optimizado por incluso, otro tipo de moléculas MHC ((HLA-DM, que se asemejan en forma a las MHC de clase II, y en algunas células trabajan al unísono junto con HLA-DO, otra molécula de clase II. La evolución es perezosa, cuando ha desarrollado algo que funciona, simplemente lo copia y modifica para nuevas funciones. El resultado neto de este complicado baile es el envío de moléculas MHC de clase II a la superficie celular con péptidos que permiten la activación de las células T auxiliares.

Diapositiva 23

El proceso de reconocimiento de patógenos por el sistema inmune es complejo… pero también relativamente lento. La primera vez que usted se encuentra con un virus, el sistema inmune tarda en preparar la respuesta antiviral. Si usted es desafortunado, esto puedo traducirse en enfermedades o la muerte por culpa de replicación viral sin revisar. La vacunación prepara al sistema inmune para una infección, permitiéndole en algunos casos prevenirla completamente, y de esta manera responde de una manera más rápida y efectiva reduciendo notablemente la posibilidad de sufrir una infección severa.

Diapositiva 24

Las moléculas MHC son participantes imprescindibles en la vacunación. Todas las vacunas hacen uso de las MHC de clase II para inducir la respuesta de anticuerpos mediante las células T auxiliares y generan las proteínas contra las que las respuesta de anticuerpos es dirigida. Las células T que son inducidas por las vacunas se mantienen en alerta por una nueva infección por el virus original durante muchos años, incluso décadas en algunos casos. Las vacunas han salvado muchas más vidas que cualquiera de las intervenciones medicas combinadas. Difunde este mensaje, no la enfermedad, ¡Vacúnate!

Epilogo

Las moléculas MHC controlan infecciones, regulan la respuesta inmune y están ahora ayudando curar el cáncer. También acarrean algunos riesgos como la autoinmunidad y rechazo de órganos. Y es por eso que usted-viviendo en un mundo lleno de patógenos- ha sobrevivido para leer este Comic. Para mas detalles de como sobrevivir incluso mejor, por favor vea las referencias 1-6
