# Supplementary material for: HLA molecules in transplantation, autoimmunity and infection control: A comic book adventure
Source: HLA. 2022 May 15;100(4):301–11. doi: 10.1111/tan.14626 (PMC9545814; doi:10.1111/tan.14626)
Supplement: Supplementary file 1 — Supporting information. [file TAN-100-301-s001.zip › Supplementary files/PP_Swedish_Lindsen.1.pdf]

HLA molekyler inom transplantation, autoimmunitet och infektioner: ett serieäventyr

HLA molecules in transplantation, autoimmunity and infection control.
A comic Book adventure

by Eric Reits and Jacques Neefjes

Translated by Kristina Lindsten. Original text : ***https://doi.org/10.1111/tan.14626***

Department of Cell and Chemical Biology, ONCODE Institute, Leiden University Medical Centre LUMC, The Netherlands

Bild 1

För ungefär 1900 år sedan genomförde två arabiska bröder och läkare, Cosmas och Damianus, den första kända transplantationen då de ersatte en köpmans kallbrandsangripna ben med hans slavs ben. Slavens öde är okänt men det var sannolikt inte en frivillig donation.

Bild 2

Denna ”mirakulösa” transplantation bidrog till deras saligförklaring och de utsågs till transplantationernas skyddshelgon. De blev halshuggna på grund av sin kristna tro, något som antagligen korrigerades vid deras himmelsfärd.

Bild 3

Varför är transplantationer så svåra, vad är de evolutionära orsakerna? Även Darwin måste ha undrat… men han kände inte till en klass proteiner som uttrycks på nästan alla flercelliga eukaryoter.

Bild 4

Låt oss börja med den nuvarande kännedomen om två unika klasser av proteiner i vår kropp; de som har den allra högsta graden av polymorfism (skillnader mellan individer). Och dessa är unika eftersom nästan alla andra proteiner är nästan identiska emellan människor. Dessa proteiner är ”transplantationsantigen” och kallas generellt MHC klass I och MHC klass II molekyler. I människor kallas de HLA klass I och HLA klass 2.

Bild 5

De viktigaste HLA molekylerna för transplantation kallas HLA-A, HLA-B and HLA-C för MHC klass 1 och HLA-DR, HLA-DQ och HLA-DP för MHC klass II. HLA-A, -B och -C finns på praktiskt taget alla våra celler (utom röda blodkroppar) medan HLA-DR, HLA-DQ och HLA-DP framför allt finns på immunceller.

Bild 6

HLA molekyler är så polymorfa att gravida kvinnor ofta utvecklar antikroppar mot pappans HLA-typ. Innan det fanns genetisk testning använde man detta fenomen för faderskapsbestämning. Men serum från dessa kvinnor användes även för transplantation. Serum från dessa kvinnor byttes mellan olika labb och olika serum-svar namngavs. Detta är hur HLA-A, -B och -C identifierades och även ytterligare varianter. Dessa namngavs helt enkelt HLA-A1, nästa HLA-A2 etc. Detta hände även med HLA-DR, -DQ och -DP molekylerna. Således, din vävnad kan ha (som ett exempel) HLA-A1, -B8, -Cw7, -DR3, -DQ2 och DPw1 proteiner från din mamma och HLA-A2, -B27, -Cw1, -DR4, -DQ3 and DPw4 proteiner från din pappa.

Bild 7

Idag görs HLA typning rutinmässigt med DNA analys. Det finns vissa bevis att kvinnor kan urskilja mäns olika HLA-typ genom luktsinnet och att detta bidrar till urvalet av en genetiskt olik matchning.

Bild 8

Medan HLA polymorfism kan hjälpa till att diversifiera mänskligheten så är det en enorm barriär för lyckad organtransplantation eftersom den kräver så nära matchning som möjligt av mottagare och donator. I brist på perfekt matchning används immunsuppresiva läkemedel för att förhindra bortstötning av organ.

Bild 9

Darwin skulle varit förbryllad. Säkerligen så är inte möjligheten att känna doften av din perfekta partner, undvika organtransplantation eller att hitta din biologiska pappa de principiella evolutionära anledningarna till HLA polymorfism.

Bild 10

Men det finns ytterligare en faktor. Virus och andra mikrobiella patogener finns i överflöd i naturen. Corona, Influensa, Ebola, smittkoppor och många andra virus använder våra celler för att föröka sig. Även infektioner som begränsar sig själva skulle vara dödliga utan ett immunsystem. Och frågan är enkel: hur kan immunsystemet upptäcka virus som lurar i celler och döda dem innan viruset dödar oss?

Bild 11

För att minska skador av virus har immunsystemet utvecklat flera olika vapen. Makrofager äter bakterier och virus, neutrofiler släpper ut substanser som dödar bakterier, B-celler gör antikroppar, T-hjälparceller hjälper B-celler och andra celler, T-mördarceller dödar virus-infekterade celler (och även cancerceller).

Bild 12

Men hur vet en T-mördarcell vem den ska döda? Viruset som är inne i cellen, är skyddat från att bli upptäckt, eller hur är det egentligen? I själva verket är det så att när viruset replikerar (förökar sig) så levereras små bitar av virusets proteiner (peptider) till HLA-A, -B eller -C-molekyler som tar dem till cellytan. T-mördarcellen känner igen detta lilla peptid-fragment i tillsammans med EN specifik HLA-molekyl. Upptäckten av detta fenomen som kallas HLA-restriktion, var tillräckligt viktig för att få två Nobelpris. Varje typ av MHC klass I-molekyl presenterar en annan uppsättning av peptider för att ge immunsystemet massor av mål att sikta på och döda cellerna som producerar dessa.

Bild 13

Men hur bildas ett virusfragmenten egentligen? Virala proteiner – precis som vilka andra proteiner som helst inuti cellen – bryts ner. Proteiner fragmenteras av en nano-maskin som kallas proteasomen, som i princip funkar som en soptunna för proteiner. Andra cellulära enzymer trimmar ändarna av fragmenten till mindre peptider, varav vissa transporteras från cytosolen till endoplasmatiska retikulet (ER) där de kan binda till HLA molekyler. När en HLA-molekyl har bundit en peptid lämnar den ER och går till cellytan där den inväntar upptäckt av T-mördarceller.

Bild 14

Låt oss gå tillbaka till HLA-polymorfismen. Det är välkänt från COVID-19 och influensa att virus kan förändra sig och undkomma upptäckt av antikroppar (tänk alfa, delta, omicron…). För att minimera denna möjlighet för T-celler så presenterar de olika MHC allelerna (genvarianter) olika set av peptider. Så många peptider presenteras i en individ vilket gör det svårt att undslippa immunförsvaret. Skillnaden i HLA-typ mellan individer innebär att även om detta händer så kommer viruset inte kunna upprätthålla sitt bedrägeri i nästa individ. Om vi alla hade haft identisk HLA-typ så skulle ett virus kunna undkomma och döda hela populationen, nu dödar det ”bara” några individer med HLA molekyler som inte presenterar virala peptider för immunförsvaret. HLA-polymorfism skyddar på så sätt populationen, den enskilda individen är mindre viktig. Sammantaget ger detta en övertygande förklaring till utvecklingen av MHC-polymorfism.

Bild 15

Men tyvärr, dåliga nyheter för dig kära läsare om du råkar behöva ett nytt organ eller två. HLA-polymorfism främjar överlevnaden av en art, inte en individ med njursjukdom. Avstötning av ett transplanterat organ är konsekvensen av att immunsystemet blandar ihop det donerade organet med ett virusinfekterat organ och svarar därmed genom att attackera organet, vilket i sin tur resulterar i avstötning.

Bild 16

En viktig allmän läxa: inget, inte ens immunsystemet är perfekt! På tal om det, låt oss fundera på hur T-mördarceller kan hitta virusinfekterade celler tillräckligt snabbt för att vara till någon nytta. Virus kan föröka sig snabbt, i vissa fall bara på några timmar. Det tar för lång tid att vänta på att de virala proteinerna ska brytas ner i slutet av sin livstid. Men precis som immunsystemet självt så är syntesen av proteiner, även de virala proteinerna, långt ifrån perfekt. Dessa bristfälliga proteiner, kallade DRiPs, bryts genast ner och kopplar därmed starten av virusinfektionen till antigenpresentation och T-mördarcellernas immunövervakning.

Bild 17

Schackmatt till immunsystemet? Inte så snabbt! Vissa smarta virus, särskilt herpesvirus, har utvecklat mekanismer för att påverka immunsystemet. Humant cytomegalovirus, HCMV, som infekterar 60% av alla människor gör en rad proteiner (US2, US3, US6, US11 och US18) som begränsar peptidproduktionen och påverkar funktionen av HLA klass I.

Bild 18

Är det då möjligt att vissa HLA alleler är bättre på att hantera virusinfektioner än andra? Ja, vissa HLA-B alleler skyddar bättre mot HIV, andra bättre mot Covid. De olika HLA allelerna har selekterats över eoner för att hantera olika patogener. Till exempel HLA-A2 finns hos 40% av alla Européer, den högsta prevalensen av en allel i en viss population. Detta beror på förmågan hos HLA-A2 att skydda mot patogener vid något tillfälle långt bak i tiden som mycket väl inte behöver utgöra någon större anledning till sjukdom idag.

Bild 19

Men det finns indirekta konsekvenser. Ta allelen HLA-B*27:05. Denna finns hos 8% av alla kaukasier, över 90% av patienter med ankyloserande spondylit har denna allel som sannolikt triggar en autoimmun T-cellsreaktion i ryggraden. Immunsystemet balanserar på en knivsegg mellan att tillhandahålla effektiv immunitet utan att skada vävnader mot vådabeskjutning.

Bild 20

T-cells autoimmunitet kan också vara av nytta. Cancerceller har ofta många mutationer och andra förändringar som leder till att peptiderna avviker från det normala. Immunterapi mot cancer utnyttjar mekanismer som används av immunsystemet för att känna igen virala och bakteriella infektioner, för att istället döda cancerceller.

Bild 21

Men hur är det med HLA-DR, -DQ och -DP MHC klass II molekyler? Dessa molekyler presenterar patogena peptider till T-hjälparceller som i sin tur producerar cytokiner som stimulerar B-celler att differentiera till antikroppsproducerande fabriker. T-hjälparceller hjälper också till med att optimera T-mördarcellssvar. MHC klass II är till formen väldigt lika MHC klass I, men de presenterar peptider som är lite längre och som kommer från lysosomer som är små organeller som bryter ner proteiner som kommer utifrån cellerna.

Bild 22

Hur gör de detta? MHC klass II produceras i ER (så som vilket annat protein som helst som ska till cellytan eller till lysosomerna) där de binder ett protein (invariant chain) som härmar en peptid och hjälper MHC klass II till lysosomen. Här byts invariant chain ut mot en peptid som genererats av lysosomala enzymer. Denna process optimeras av ännu en typ av MHC molekyl (HLA-DM, som liknar MHC klass II och i vissa celler fungerar i samförstånd med HLA-DO, en annan klass II-liknande molekyl. Evolutionen är lat, när den väl utvecklat en fungerande modell, så kopierar den helt enkelt den för nya funktioner). Summan av denna komplicerade dans är att MHC klass II molekylen kommer till cellytan med peptider som gör det möjligt att aktivera T-hjälparceller.

Bild 23

Denna process om hur immunsystemet känner igen patogener är komplex… men den är också relativt långsam. Den första gången du stöter på ett virus så tar det ett tag för immunsystemet att dra igång det anti-virala svaret. Om du har otur så resulterar detta i sjukdom eller död på grund av okontrollerad virusreplikering. Vaccination förbereder immunsystemet för en infektion och gör att man ibland kan undvika infektion helt och hållet eller svara snabbare och effektivare och starkt reducera risken för en allvarlig infektion.

Bild 24

MHC molekyler är kritiska deltagare vid vaccination. Alla vacciner utnyttjar MHC klass II molekyler för att inducera T-hjälparceller som behövs för att producera antikroppssvar. Adenovirus and mRNA vaccin använder också MHC klass I molekyler för att inducera T-mördarceller. T-celler som induceras av vaccin håller i många år, även årtionden i vissa fall, och är på sin vakt inför nya infektioner med det ursprungliga viruset. Vacciner har räddad långt fler liv än alla andra medicinska ingripanden sammanlagt. Sprid detta meddelande, inte sjukdomen, vaccinera dig!

Epilog

Så, MHC molekyler kontrollerar infektion, reglerar immunsvar och hjälper till att bota cancer. Allt detta väger upp mot nackdelen av autoimmunitet och avstötning vid transplantation. Och det är därför som du – som lever i en värld full av patogener – har överlevt och kan läsa detta serieäventyr. För mer detaljer om hur man kan överleva ännu bättre se referenserna 1-6.
