# Supplementary material for: HLA molecules in transplantation, autoimmunity and infection control: A comic book adventure
Source: HLA. 2022 May 15;100(4):301–11. doi: 10.1111/tan.14626 (PMC9545814; doi:10.1111/tan.14626)
Supplement: Supplementary file 1 — Supporting information. [file TAN-100-301-s001.zip › Supplementary files/PP_Thai_Kittisares_1.pdf]

โมเลกุล HLA ในการปลูกถ่ายอวัยวะ ภาวะภูมิคุ้มกันต้านตนเอง และการควบคุมการติดเชื้อ: การผจญภัยในรูปแบบหนังสือการ์ตูน

HLA molecules in transplantation, autoimmunity and infection control.
A comic Book adventure

by Eric Reits and Jacques Neefjes

Translated by Kulvara Kittisares. Original text : ***https://doi.org/10.1111/tan.14626***

Department of Cell and Chemical Biology, ONCODE Institute, Leiden University Medical Centre LUMC, The Netherlands

1 | สไลด์ 1

เมื่อประมาณ 1900 ปีที่แล้ว พี่น้องผู้เป็นแพทย์ชาวอาหรับสองคนชื่อคอสมาสและดาเมียนุส ได้ทำการปลูกถ่ายอวัยวะที่เป็นที่รู้จักเป็นครั้งแรก โดยเป็นการปลูกถ่ายขาที่เน่าเปื่อยของพ่อค้าด้วยขาของทาสของเขา ชะตากรรมของทาสผู้นั้นไม่เป็นที่ทราบในประวัติศาสตร์ แต่ไม่น่าจะเป็นการบริจาคอวัยวะโดยสมัครใจ

2| สไลด์ 2

การปลูกถ่าย “ปาฏิหาริย์” ในครั้งนี้มีส่วนในการประสาทพรให้กับทั้งสองและนำไปสู่การเป็นนักบุญอุปถัมภ์แห่งการปลูกถ่ายอวัยวะ ดังนั้นการที่พวกเขาถูกตัดศีรษะเนื่องจากความเชื่อในคริสเตียนจึงไม่เป็นเรื่องน่าเจ็บปวดเพราะเชื่อกันว่าจะพวกเขาน่าจะได้รับการแก้ไขเมื่อขึ้นสวรรค์

3 | สไลด์ 3

เหตุใดการปลูกถ่ายจึงเป็นเรื่องยากและปัจจัยของวิวัฒนาการที่เกี่ยวข้องคืออะไร?
แม้แต่ดาร์วินยังต้องสงสัย... แต่ดาร์วินยังไม่รู้เกี่ยวกับโปรตีนประเภทหนึ่งที่มีลักษณะเฉพาะซึ่งแสดงออกโดยสิ่งมีชีวิตชนิดยูคาริโอตหลายเซลล์เกือบทั้งหมด

4 | สไลด์ 4

เรามาเริ่มต้นกันด้วยความเข้าใจในปัจจุบันเกี่ยวกับโปรตีนสองประเภทในร่างกายของเรา ซึ่งมีระดับความหลากหลายมากที่สุด (แตกต่างกันไปประหว่างบุคคล) ซึ่งทำให้โปรตีนสองชนิดนี้มีลักษณะจำเพาะเนื่องจากโปรตีนอื่นๆเกือบทั้งหมดมีความคล้ายคลึงกันระหว่างมนุษย์แต่ละคน โปรตีนเหล่านี้ถือเป็น "แอนติเจนของการปลูกถ่ายอวัยวะ" และโดยทั่วไปเรียกว่าโมเลกุล MHC class I และ MHC class II ส่วนในมนุษย์เรียกว่า HLA class I และ HLA class II

5 | สไลด์ 5

โมเลกุล HLA ที่สำคัญที่สุดสำหรับการปลูกถ่ายในกลุ่ม MHC class I คือ HLA-A, HLA-B และ HLA-C ส่วนในกลุ่ม MHC class II คือ HLA-DR, HLA-DQ และ HLA-DP โมเลกุล HLA-A, -B และ -C มีอยู่ในแทบทุกเซลล์ของเรา (ยกเว้นเซลล์เม็ดเลือดแดง) ในขณะที่ HLA-DR, HLA-DQ และ HLA-DP ส่วนใหญ่จะอยู่ในเซลล์ภูมิคุ้มกัน

6 | สไลด์ 6

โมเลกุล HLA นั้นมีความหลากหลายมากจนทำให้หญิงตั้งครรภ์มักจะสร้างแอนติบอดีต่อ HLA ของพ่อของเด็กในครรภ์ที่แตกต่างไปจากของตนเอง โดยการสร้างแอนติบอดีของหญิงตั้งครรภ์นี้สามารถนำไปใช้เพื่อตรวจสอบความเป็นพ่อในอดีตก่อนที่จะมีการทดสอบทางพันธุกรรม นอกจากนั้นซีรั่มของหญิงตั้งครรภ์เหล่านี้ยังถูกนำมาใช้สำหรับการตรวจเนื่อเยื่อในการปลูกถ่ายอวัยวะ โดยมีการแลกเปลี่ยนซีรั่มจากหญิงตั้งครรภ์ระหว่างห้องปฏิบัติการในการประชุมเชิงปฏิบัติการ และในการประชุมเชิงปฏิบัติการ HLA ได้มีการตั้งชื่อการตอบสนองของซีรั่มที่แตกต่างกันไป และนี่ก็คือการที่โมเลกุล HLA-A, -B และ -C ได้ถูกค้นพบ รวมถึงการค้นพบชนิดย่อยต่างๆของ HLA ด้วยเช่นเดียวกัน โดยชนิดย่อยต่างๆของ HLA ได้ถูกตั้งชื่ออย่างง่ายๆโดยใส่หมายเลขตามไปเป็น HLA-A1 และลำดับถัดไปเป็น HLA-A2 ไปเรื่อย ๆ โดยในการตั้งชื่อ HLA-DR, -DQ และ -DP ก็เป็นเช่นเดียวกัน ดังนั้นเนื้อเยื่อของคุณอาจมี (ตัวอย่างเช่น) โปรตีน HLA-A1, -B8, -Cw7, -DR3, -DQ2 และ DPw1 จากแม่ของคุณ และโปรตีน HLA-A2, -B27, -Cw1, -DR4, -DQ3 และ DPw4 จากพ่อของคุณ

7 | สไลด์ 7

ในทุกวันนี้การตรวจชนิดของ HLA นั้นโดยทั่วไปทำโดยการวิเคราะห์ดีเอ็นเอ มีหลักฐานบางอย่างที่แสดงว่าผู้หญิงสามารถตรวจจับความแตกต่างของชนิดHLAของผู้ชายได้ด้วยดมกลิ่นและสิ่งนี้มีส่วนในการเลือกคู่ครองที่แตกต่างกันทางพันธุกรรม

8 | สไลด์ 8

แม้ว่าความหลากหลายของ HLA อาจช่วยกระจายความหลากหลายของมนุษยชาติได้ แต่ก็เป็นอุปสรรคใหญ่ต่อความสำเร็จในการปลูกถ่ายอวัยวะ ซึ่งต้องมีการจับคู่ชนิดของHLA ของผู้รับและผู้บริจาคให้ใกล้เคียงกันที่สุด ในกรณีที่ไม่มีคู่ที่สมบูรณ์แบบจะมีการใช้ยากดภูมิคุ้มกันที่มีประสิทธิภาพเพื่อป้องกันการปฏิเสธอวัยวะ

9 | สไลด์ 9

แต่ก็ยังคงเป็นปริศนาสำหรับดาร์วิน แน่นอนว่าทั้งการดมกลิ่นคู่ครองของคุณ การป้องกันการปลูกถ่ายเนื้อเยื่อ หรือการค้นหาพ่อที่แท้จริง ไม่ใช่สาเหตุหลักของวิวัฒนาการของ HLA

10 | สไลด์ 10

แต่ยังมีปัจจัยอีกอย่างคือไวรัสและจุลินทรีย์ก่อโรคอื่น ๆนั้นมีอยู่มากมายในธรรมชาติ ทั้งโคโรนา ไข้หวัดใหญ่ อีโบลา ไข้ทรพิษ และไวรัสอื่นๆอีกมากต่างก็ใช้เซลล์ของเราเพื่อสร้างครอบครัวของตัวเอง และหากปราศจากระบบภูมิคุ้มกันแม้แต่การติดเชื้อที่โดยปกติหายได้เองก็อาจทำให้ถึงแก่ชีวิตได้

และคำถามง่ายๆก็คือ : ระบบภูมิคุ้มกันสามารถตรวจจับไวรัสที่แฝงตัวอยู่ภายในเซลล์เพื่อฆ่าพวกมันก่อนที่มันจะฆ่าเราได้อย่างไร?

11 | สไลด์ 11

ระบบภูมิคุ้มกันได้พัฒนาอาวุธหลายชนิดเพื่อจำกัดความเสียหายจากไวรัส อันได้แก่ แมคโครฟาจกินแบคทีเรียและไวรัส นิวโทรฟิลปล่อยสารฆ่าแบคทีเรีย เซลล์ B สร้างแอนติบอดี เซลล์ T-helper ช่วยเซลล์ B และเซลล์อื่นๆ เซลล์ T-killer ฆ่าเซลล์ที่ติดไวรัส (และแม้กระทั่งเซลล์มะเร็ง)

12 | สไลด์ 12

แต่เซลล์ T-killer รู้ได้อย่างไรว่าจะต้องฆ่าใคร? ไวรัสที่อยู่ภายในเซลล์ถูกป้องกันจากการตรวจพบหรือไม่?

ที่จริงแล้ว เมื่อไวรัสแบ่งตัว จะมีการส่งโปรตีนชิ้นเล็ก ๆ ไปยังโมเลกุล HLA-A, -B หรือ -C ซึ่งนำพาพวกมันไปยังผิวเซลล์ เซลล์ T-killer รับรู้ถึงชิ้นส่วนเล็กๆนี้ในบริบทของโมเลกุล HLA จำเพาะหนึ่งโมเลกุล การค้นพบปรากฏการณ์นี้ซึ่งเรียกว่าความจำเพาะต่อ HLA (HLA-restriction) มีความสำคัญมากพอที่ทำให้ได้รับรางวัลโนเบลสองรางวัล โมเลกุล MHC class I แต่ละประเภทต่างแสดงสายเปปไทด์ที่แตกต่างกันเพื่อให้เป้าหมายจำนวนมากแก่ระบบภูมิคุ้มกันในการเล็งและฆ่าเซลล์ที่ผลิตสิ่งเหล่านี้

13 | สไลด์ 13

แต่ชิ้นส่วนไวรัสเกิดขึ้นได้อย่างไรตั้งแต่แรก? โปรตีนจากไวรัสซึ่งเหมือนกับโปรตีนอื่นๆที่อยู่ภายในเซลล์มีการหมดอายุขัยและถูกแยกเป็นชิ้นส่วนเล็กๆโดยเครื่องจักรขนาดนาโนซึ่งเรียกว่า proteosome ซึ่งจริงๆแล้วจะกำจัดโปรตีนทุกชนิด ต่อมาจะมีเอนไซม์ในเซลล์อีกชนิดจะตัดแต่งปลายของชิ้นส่วนของโปรตีนให้เป็นเปปไทด์ที่มีขนาดเล็กลง บางส่วนของเปปไทด์นี้จะถูกลำเลียงจากไซโตซอลไปยัง ER ซึ่งพวกมันสามารถจับโมเลกุล HLA ได้ เมื่อโมเลกุล HLA มีเปปไทด์ที่จับอยู่ มันจะออกจาก ER ไปที่ผิวเซลล์และรอการตรวจหาโดยเซลล์ T-killer ที่นั่น

14 | สไลด์ 14

กลับไปที่ความหลากหลายของ HLA อย่างที่เราทราบกันดีจาก COVID-19 และไข้หวัดใหญ่ว่าไวรัสมีการเปลี่ยนแปลงเพื่อหลีกเลี่ยงการตอบสนองของแอนติบอดีได้เป็นอย่างดี (ลองนึกถึงอัลฟา เดลต้า โอไมครอน….) ดังนั้นเพื่อเป็นการลดโอกาสที่จะหลบเลี่ยงของไวรัสจากเซลล์ T แต่ละอัลลีล (ยีนที่หลากหลาย) ของ MHC จะแสดงชุดเปปไทด์ที่แตกต่างกัน และในคนๆเดียวจะมีเปปไทด์ที่แสดงเป็นจำนวนมากทำให้การหลีกเลี่ยงภูมิคุ้มกันไวรัสนั้นทำได้ยาก และหากว่ามีไวรัสที่หลบเลี่ยงจากภูมิคุ้มกันเกิดขึ้นในบุคคลคนหนึ่ง ความแตกต่างของชนิด HLA ระหว่างบุคคลทำให้ไวรัสที่หลบเลี่ยงมาได้นั้นจะไม่สามารถหลบเลี่ยงได้เหมือนเดิมในอีกบุคคลหนึ่ง ดังนั้นไวรัสสามารถฆ่าคนเพียงไม่กี่คนที่มีโมเลกุล HLA ที่ไม่สามารถแสดงเปปไทด์ของไวรัสต่อระบบภูมิคุ้มกันได้ แต่หากเราทุกคนต่างก็มี HLA เหมือนกันไวรัสที่หลบเลี่ยงมาได้ก็จะฆ่าประชากรทั้งหมด ความหลากหลายของ HLA จึงเป็นการปกป้องระดับประชากรและมีความสำคัญน้อยกว่าในระดับปัจเจกบุคคล และสิ่งนี้เองเป็นคำอธิบายที่น่าสนใจสำหรับวิวัฒนาการของความหลากหลายของ MHC

15 | สไลด์ 15

แต่อนิจจา ข่าวร้ายสำหรับคุณผู้อ่านที่รัก หากคุณต้องการอวัยวะใหม่หนึ่งหรือสองอวัยวะ ความหลากหลายของ HLA นั้นส่งเสริมการอยู่รอดของประชากรทั้งสปีชีส์แต่ไม่ใช่ปัจเจกบุคคลที่เป็นโรคไต การปฏิเสธการปลูกถ่ายเป็นผลของระบบภูมิคุ้มกันที่สับสนระหว่างอวัยวะของผู้บริจาคกับอวัยวะที่ติดเชื้อไวรัสและตอบสนองตามนั้นโดยการโจมตีอวัยวะซึ่งส่งผลให้เกิดการปฏิเสธการปลูกถ่าย

16 | สไลด์ 16

บทเรียนทั่วไปที่สำคัญ: ไม่มีอะไรรวมทั้งระบบภูมิคุ้มกันที่สมบูรณ์แบบ! เมื่อพูดถึงเรื่องนี้ ลองคิดดูว่าเซลล์ T-killer สามารถค้นหาเซลล์ที่ติดไวรัสได้เร็วพอที่จะนำไปใช้ได้อย่างไร ไวรัสสามารถให้กำเนิดลูกหลานได้เร็วมากในบางกรณีภายในเวลาเพียงไม่กี่ชั่วโมง ซึ่งการที่จะรอให้โปรตีนจากไวรัสหมดสภาพเมื่อสิ้นสุดชีวิตตามธรรมชาตินั้นช้าเกินไป แต่ก็เหมือนกับระบบภูมิคุ้มกันที่การสังเคราะห์โปรตีน รวมทั้งโปรตีนจากไวรัสยังห่างไกลจากความสมบูรณ์แบบ โปรตีนที่ไม่สมบูรณ์เหล่านี้เรียกว่า DRiP จะมีการเสื่อมสภาพในทันที โดยเชื่อมโยงการเริ่มต้นของการติดเชื้อไวรัสกับการแสดงตัวของแอนติเจนและช่วยให้การเฝ้าระวังภูมิคุ้มกันของเซลล์ T-killerมีประสิทธิภาพ

17 | สไลด์ 17

ภูมิคุ้มกันรุกฆาตแล้วหรือ? ไม่เร็วขนาดนั้น! ไวรัสที่ฉลาดบางตัวโดยเฉพาะไวรัสเริม (Herpesvirus) ได้วิวัฒนาการมาขัดขวางการแสดงตัวของแอนติเจน อย่างเช่นเชื้อไวรัสไซโตเมกาโลไวรัสมนุษย์ หรือ HCMV ซึ่ง 40% ของมนุษย์ติดเชื้อนี้ สร้างชุดของโปรตีน (US2, US3, US6, US11 และ US18)ที่จำกัดการผลิตเปปไทด์หรือรบกวนการทำงานของ HLA class I

18 | สไลด์ 18

เป็นไปได้ไหมที่อัลลีล HLA บางตัวสามารถจัดการกับการติดไวรัสได้ดีกว่าอัลลีลอื่น? อันที่จริงมี HLA-Bบางอัลลีลสามารถป้องกันเชื้อ HIV ได้ดีกว่า ส่วนบางอัลลีลป้องกันเชื้อ Covid ได้ดีกว่า โดยอัลลีล HLA ที่แตกต่างกันได้รับการคัดเลือกในช่วงเวลาต่างกันเพื่อจัดการกับเชื้อโรคชนิดที่ต่างกัน ตัวอย่างเช่น มีการพบ HLA-A2 ใน 60% ของประชากรยุโรป ซึ่งเป็นอัลลีล HLA ที่มีความชุกสูงสุดในกลุ่ม ซึ่งอาจเป็นผลมาจาก ความสามารถของ HLA-A2 ในการป้องกันเชื้อโรคชนิดหนึ่งในช่วงเวลาหนึ่งในอดีต ที่ปัจจุบันนี้ไม่ใช่เชื้อโรคที่เป็นสาเหตุหลักของโรคในมนุษย์อีกต่อไป

19 | สไลด์ 19

แต่ก็มีผลที่ตามมาด้วยเช่นกัน ตัวอย่างเช่นอัลลีล HLA-B*27:05 ซึ่งมีอยู่ใน 8%ของประชากรคอเคเซียนนั้น พบว่ามากกว่า 90% ของผู้ป่วยโรคกระดูกสันหลังอักเสบ ancylosing spondylitis มีอัลลีลนี้อยู่ ซึ่งอัลลีลนี้น่าจะกระตุ้นปฏิกิริยต่อต้านตนเองของเซลล์ T ในกระดูกสันหลัง ระบบภูมิคุ้มกันนั้นเป็นการปฏิบัติการอยู่บนคมมีด ระหว่างการให้ภูมิคุ้มกันที่มีประสิทธิภาพหรือการสร้างความเสียหายจากเนื้อเยื่อ

20 | สไลด์ 20

ภูมิคุ้มกันต่อต้านตนเองของเซลล์ T ก็มีประโยชน์เช่นกัน เซลล์มะเร็งมักจะมี การกลายพันธุ์และการดัดแปลงอื่นๆมากมายที่นำไปสู่การสร้างเปปไทด์ที่แตกต่างจากเปปไทด์ในเซลล์ปกติ โดยการรักษามะเร็งโดยภูมิคุ้มกันบำบัดใช้ประโยชน์ของกลไกที่ระบบภูมิคุ้มกันใช้ในการจดจำการติดเชื้อไวรัสและ แบคทีเรีย เพื่อใช้ฆ่าเซลล์มะเร็ง

21 | สไลด์ 21

แล้วโมเลกุล HLA-DR, -DQ และ -DP MHC class II ล่ะ? โมเลกุลเหล่านี้แสดงเปปไทด์ที่ทำให้เกิดโรคต่อเซลล์ T-helper ซึ่งผลิตไซโตไคน์ เพื่อช่วยให้เซลล์ B พัฒนาไปเป็นเซลล์ที่เป็นโรงงานผลิตแอนติบอดี เซลล์ T-helper ยังช่วยเพิ่มประสิทธิภาพการตอบสนองของ T-killer ด้วยเช่นกัน MHC class II มีรูปร่างคล้ายกันมากกับ MHC class I แต่มีชิ้นส่วนโปรตีนที่ ยาวกว่าและสร้างขึ้นในไลโซโซม (lysosome) ซึ่งเป็นออร์แกเนลล์ขนาดเล็กที่ย่อยสลายโปรตีนที่ได้มาจากภายนอกเซลล์

22 | สไลด์ 22

มันทำได้อย่างไร? MHC class II ถูกสร้างขึ้นใน ER (เช่นเดียวกับโปรตีนอื่น ๆ ที่ไปอยู่ที่ด้านนอกของเยื่อหุ้มเซลล์หรือไลโซโซม) ที่นั่นโมเลกุลจะจับโปรตีนชนิดหนึ่งคือโปรตีนสายคงที่ (invariant chain) ซึ่งเลียนแบบเปปไทด์และนำโมเลกุล MHC class II เข้าสู่ไลโซโซม ที่ไลโซโซมโปรตีนสายคงที่จะถูกเอาออกและแลกเปลี่ยนเป็นเปปไทด์ที่สร้างขึ้นโดยไลโซโซม กระบวนการนี้ได้รับการปรับให้เหมาะสมโดยโมเลกุล MHC อีกประเภทหนึ่ง (คือ HLA-DM ซึ่งมีลักษณะคล้ายกับ MHC class II และในบางเซลล์ก็ทำงานร่วมกับ HLA-DO ซึ่งเป็นโมเลกุลคล้าย MHC class II อีกชนิดหนึ่ง ดูเหมือนว่าวิวัฒนาการนั้นมีความเกียจคร้าน เมื่อพัฒนาโมดูลที่ทำงานได้แล้วมันจะแค่คัดลอกและปรับเปลี่ยนสำหรับฟังก์ชันใหม่) ผลลัพธ์สุทธิของกระบวนการที่ซับซ้อนนี้คือการนำส่งโมเลกุล MHC class II ไปยังพื้นผิวเซลล์ที่มีเปปไทด์ที่จะไปกระตุ้นเซลล์ T- helper ทำงาน

23 | สไลด์ 23

กระบวนการตรวจหาเชื้อโรคโดยระบบภูมิคุ้มกันนี้ซับซ้อน...แต่ก็ค่อนข้างช้าเช่นกัน ครั้งแรกที่คุณพบไวรัสระบบภูมิคุ้มกันต้องใช้เวลาเพื่อพัฒนาการตอบสนองต่อไวรัส หากคุณโชคร้ายอาจส่งผลให้เกิดโรคหรือเสียชีวิตจากการแบ่งตัวไวรัสที่ไม่ถูกตรวจพบ การฉีดวัคซีนช่วยเตรียมระบบภูมิคุ้มกันสำหรับการติดเชื้อ ทำให้ในบางกรณีสามารถป้องกันการติดเชื้อได้อย่างสมบูรณ์หรือไม่เช่นนั้นก็เพื่อตอบสนองได้อย่างรวดเร็วและมีประสิทธิภาพยิ่งขึ้นและลดโอกาสของการติดเชื้อรุนแรงได้อย่างมาก

24 | สไลด์ 24

โมเลกุล MHC มีบทบาทสำคัญเป็นอย่างยิ่งในการฉีดวัคซีน วัคซีนทุกชนิดจะใช้โมเลกุล MHC class II เพื่อกระตุ้นเซลล์ T-helper ที่จำเป็นสำหรับการตอบสนองของแอนติบอดีและโปรตีนที่เป็นเป้าของการตอบสนองโดยแอนติบอดี วัคซีนชนิด Adenovirus และ mRNA ยังใช้โมเลกุล MHC class I เพื่อกระตุ้นเซลล์ T-killer เซลล์ T ที่เกิดจากวัคซีนมีอายุนานหลายปีหรือแม้แต่หลายสิบปีในบางกรณี เพื่อเตรียมพร้อมสำหรับการติดเชื้อใหม่ด้วยไวรัสดั้งเดิม วัคซีนช่วยชีวิตคนได้มากกว่าการรักษาทางการแพทย์อื่นๆรวมกัน โปรดเผยแพร่ข้อความนี้ ไม่ใช่แพร่โรค รับวัคซีนกันเถอะ!

บทส่งท้าย

ดังนั้นโมเลกุล MHC จึงควบคุมการติดเชื้อ ควบคุมการตอบสนองของภูมิคุ้มกัน และขณะนี้กำลังช่วยรักษามะเร็ง ซึ่งก็คุ้มค่ากับกับด้านร้ายที่ทำให้เกิดาวะภูมิคุ้มกันต่อต้านตนเองและการปฏิเสธการปลูกถ่ายอวัยวะ และนั่นก็คือเหตุผลที่คุณ ผู้ซึ่งอาศัยอยู่ในโลกที่เต็มไปด้วยเชื้อโรครอดชีวิตมาอ่านหนังสือการ์ตูนเล่มนี้ได้

สำหรับรายละเอียดเพิ่มเติมเกี่ยวกับวิธีเอาตัวรอดให้ดียิ่งขึ้น โปรดดูเอกสารอ้างอิง 1–6
