# Supplementary material for: HLA molecules in transplantation, autoimmunity and infection control: A comic book adventure
Source: HLA. 2022 May 15;100(4):301–11. doi: 10.1111/tan.14626 (PMC9545814; doi:10.1111/tan.14626)
Supplement: Supplementary file 1 — Supporting information. [file TAN-100-301-s001.zip › Supplementary files/PP_Turkish_Cabukusta.1.pdf]

Organ Nakli, Otoimmünite ve Enfeksiyon Kontrolünde HLA Molekülleri: Bir Çizgi Roman Macerası

HLA molecules in transplantation, autoimmunity and infection control.
A comic Book adventure

by Eric Reits and Jacques Neefjes

Translated by Birol Çabukusta. Original text : https://doi.org/10.1111/tan.14626

Department of Cell and Chemical Biology, ONCODE Institute, Leiden University Medical Centre LUMC, The Netherlands

Slayt 1

Yaklaşık 1900 yıl önce, iki Arap kardeş ve doktor Cosmas ve Damianus, bir tüccarın kangrenli bacağını kölesininkiyle değiştirerek bilinen ilk organ naklini gerçekleştirdiler. Kölenin akıbeti tarihte bilinmemekle birlikte, gönüllü bir bağış olması pek olası değildir.

Slayt 2

Bu “mucizevi” nakil, onların, nakillerin Koruyucu Azizleri ilan edilmelerine yol açtı. Hıristiyan inançları nedeniyle başlarının kesilmesi göğe çıktıklarında muhtemelen düzeltilerek onların canlarını yakmadı.

Slayt 3

Organ nakli neden bu kadar zor ve evrimsel faktörleri nelerdir? Darwin bile merak etmiş olmalı... ama o bile tüm çok hücreli ökaryotlarda bulunan benzersiz bir protein sınıfını bilmiyordu.

Slayt 4

Vücudumuzdaki iki benzersiz protein sınıfının güncel anlayışıyla başlayalım; bunlar bireyler arasında en çok farklılık gösteren, bir diğer deyişle en yüksek polimorfizm derecesine sahip olan proteinlerdir. Bu polimorfizm gösteren proteinler “nakil antijenleri”dir ve genel olarak sınıf I ve sınıf II MHC molekülleri olarak adlandırılırlar. İnsanlarda is HLA sınıf I ve HLA sınıf II olarak adlandırılırlar.

Slayt 5

Nakil için en önemli HLA molekülleri, sınıf I için HLA-A, HLA-B ve HLA-C ve sınıf II için HLA-DR, HLA-DQ ve HLA-DP olarak adlandırılır. HLA-A, HLA-B ve HLA-C kırmızı kan hücreleri hariç tüm hücrelerimizde bulunurken, HLA-DR, HLA-DQ ve HLA-DP esas olarak bağışıklık hücrelerinde bulunur.

Slayt 6

HLA molekülleri o kadar polimorfizm gösterirler ki hamile kadınlar genellikle bebeğin babasının farklı HLA tiplerine karşı antikorlar üretir. Bu, genetik test yapılmadan önceki zamanlarda babayı belirlemek için kullanılırdı. Hamile kadınların bu kan serumları doku nakli için de kullanılırdı. Bilimsel kongrelerde, bu kadınlardan alınan serumlar, laboratuvarlar arasında değiş tokuş edilirdi ve bu şekilde farklı serum yanıtları HLA araştırmalarında adlandırıldı. HLA-A, HLA-B, HLA-C ve ayrıca bunların farklı formları bu şekilde tanımlanmıştır. Bu farklı formlar sadece HLA-A1, HLA-A2 vb. şeklinde olarak numaralandırıldı. Bu numaralandırma HLA-DR, -DQ ve -DP moleküllerinde de oldu. Örneğin, dokularınız annenizden gelen HLA-A1, HLA-B8, HLA-Cw7, HLA-DR3, HLA-DQ2 ve HLA-DPw1 proteinlerine ve babanızdan gelen HLA-A2, HLA-B27, HLA-Cw1, HLA-DR4, HLA-DQ3, ve HLA-DPw4 taşıyor olabilir.

Slayt 7

Günümüzde HLA tiplemesi rutin olarak DNA analizleri ile yapılmaktadır. Kadınların erkeklerin HLA tiplerindeki farklılıkları koku yoluyla tespit edebildiğine ve bunun genetik olarak farklı eşler için seçimine katkıda bulunduğuna dair bazı kanıtlar vardır.

Slayt 8

HLA polimorfizmi insanlığı çeşitlendirmeye yardımcı olabilirken, organ alıcı ve vericinin HLA tiplerini mümkün olduğunca yakın eşleşmesi başarılı organ nakli için gereken büyük bir engeldir. Mükemmel bir eşleşme olmadığında organ reddini önlemek için etkili immünosupresif ilaçlar kullanılır.

Slayt 9

Bu bilgiler, bugün hayatta olsaydı Darwin'in kafasını karıştırırdı. Elbette mükemmel eşinizi koklamak, doku naklini engellemek veya gerçek babayı bulmak HLA polimorfizminin temel evrimsel nedenleri olamaz.

Slayt 10

Elbette bunun altında yatan başka bir faktör var. Virüsler ve diğer mikrobik patojenler doğada bol miktarda bulunur. Korona, grip, ebola, çiçek hastalığı ve diğer birçok virüs, vücut hücrelerimizi çoğalmak ve kendi ailelerini oluşturmak için kullanırlar. Bağışıklık sistemi olmadan en küçük enfeksiyonlar bile ölümcül olabilir. Bu çok basit bir soruyu ortaya atar: Bağışıklık sistemi, hücrelerin içinde gizlenen virüsleri, onlar bizi öldürmeden önce virüsleri öldürmek için nasıl algılayabilir?

Slayt 11

Virüslerden kaynaklanan hasarı sınırlamak için bağışıklık sistemi birden fazla silah geliştirmiştir. Makrofajlar bakteri ve virüsleri yer; nötrofiller bakteriler için öldürücü maddeler salgılar; B hücreleri antikorlar üretir; yardımcı T hücreleri, B hücrelerine ve diğer hücrelere yardım eder; öldürücü T hücreleri ise virüs bulaşmış vücut hücrelerini (ve hatta kanser hücrelerini) öldürür.

Slayt 12

Ama bir öldürücü T hücresi, kimi öldüreceğini nasıl bilir? Virüs, hücrenin içinde olduğu için algılanmaya karşı korunmuş olmuyor mu? Tabi virüs çoğalırken, proteinlerinin küçük parçaları onları hücre yüzeyine taşıyan HLA-A, HLA-B veya HLA-C moleküllerine iletilir. Öldürücü T hücresi, bu küçük virüs parçasını HLA molekülü üzerinde tanır. HLA kısıtlaması olarak adlandırılan bu fenomeni keşfetmek, iki Nobel Ödülü almaya yetecek kadar önemliydi. Her farklı tipteki sınıf I MHC molekülü, bağışıklık sistemine bunları üreten hücreleri hedeflemesi ve öldürmesi için birçok hedef vermek amacıyla farklı bir peptit repertuarı sunar.

Slayt 13

Ancak bir virüs proteininin parçası ilk etapta nasıl oluşturulur? Virüs proteinleri -tıpkı hücre içindeki diğer proteinler gibi- parçalanır. Proteinler, temelde proteinler için bir “yok et” anlamına gelen proteazom adı verilen olağanüstü bir nano-makine tarafından parçalanır. Diğer hücre enzimleri, proteinleri endoplazmik retikulumda HLA moleküllerine bağlanabilecekleri daha küçük peptitlere dönüştürür. Bir HLA molekülü bağlı bir peptit içerdiğinde, endoplazmik retikulumdan öldürücü T hücreler tarafından saptanmayı bekleyeceği hücre yüzeyine bırakır.

Slayt 14

HLA polimorfizmine geri dönelim. Herkesin COVID-19 ve gripten bildiği gibi, virüsler antikor tepkisinden kaçmakta çok iyidir (alfa, delta, omikron varyantlarını düşünün). T hücreleri için bu olasılığı en aza indirmek için, farklı MHC allellerinin (gen çeşitlerinin) her biri farklı bir peptit seti sunar. Bir kişide o kadar çok peptit sunulur ki, virüsün kaçması zorlaşır. Kaçabilse bile, insanlar arasındaki HLA tiplerindeki farklılıklar, kaçan virüsün bir sonraki kişide aldatmacasını sürdüremeyeceği anlamına gelir. Hepimiz HLA özdeş olsaydık, kaçan bir virüs tüm popülasyonu öldürürdü, şimdi ise HLA molekülleri bağışıklık sistemine viral peptitleri sunamayan "sadece" birkaç kişiyi öldürecek. Böylece, HLA polimorfizmi popülasyonu korur, birey daha az önemlidir. Bu, MHC polimorfizminin evrimi için ilginç bir açıklama sağlar.

Slayt 15

Ama ne yazık ki, bu sizin için, sevgili okuyucu, yeni bir veya iki organa ihtiyacınız varsa kötü haber. HLA polimorfizmi, böbrek hastalığı olan bir bireyin değil, bir popülasyonunun hayatta kalmasını destekler. Nakil reddi, bağışıklık sisteminin nakledilen organı, virüs bulaşmış bir organla karıştırmasının ve buna göre organa saldırarak yanıt vermesinin sonucudur.

•

Slayt 16

Önemli bir genel ders: Bağışıklık sistemi dahil hiçbir şey mükemmel değildir! Bundan bahsetmişken, öldürücü T hücrelerin virüs bulaşmış hücreleri herhangi bir işe yarayacak kadar hızlı nasıl bulabildiğini düşünelim. Virüsler, yavrularını çok hızlı bir şekilde üretebilir, bu bazen sadece birkaç saat alır. Bu süre, virüs proteinlerinin doğal yaşamlarının sonunda bozulmasını beklemek için çok yavaştır. Ancak tıpkı bağışıklık sisteminin kendisi gibi, virüs proteinlerinin sentezi de mükemmel değildir. DRiP'ler olarak adlandırılan bu kusurlu proteinler hemen parçalanır. Bu parçalanmış proteinler, virüs enfeksiyonunun başlangıcını antijen sunumuna bağlarlar ve etkili öldürücü T hücre bağışıklık gözetimini sağlarlar.

Slayt 17

…şah ve mat, bağışıklık sistemi, değil mi? O kadar hızlı değil! Bazı akıllı virüsler, özellikle herpes virüsleri, antijen sunumuna müdahale edecek şekilde evrimleşmiştir. İnsanlığın %60'ını enfekte eden insan sitomegalovirüsü HCMV, peptit üretimini sınırlayan veya HLA sınıf I işlevine müdahale eden bir dizi protein (US2, US3, US6, US11 ve US18) üretir.

Slayt 18

O halde bazı HLA alellerinin virüs enfeksiyonlarına karşı diğerlerinden daha iyi olması mümkün müdür? Gerçekten de bazı HLA-B alelleri HIV'e karşı daha iyi korurken, diğerleri COVID'e karşı daha etkilidir. Farklı patojenlerle başa çıkmak için çağlar boyunca farklı HLA alelleri seçilmiştir. Örneğin, HLA-A2, Avrupa popülasyonunun %40'ında bulunur ve bu belirli bir grupta herhangi bir HLA alelinin en yüksek yaygınlığıdır. Bu muhtemelen HLA-A2'nin geçmişte bir noktada hastalığa neden olan fakat bugün artık çok mühim olmayan bir patojene karşı koruma yeteneğinden kaynaklanmaktadır.

Slayt 19

Ama bunun ikincil sonuçları da var. HLA aleli HLA-B*27:05'i örnek alın. Kafkas popülasyonunun %8'inde mevcut olan Ansilozan Spondilit hastalarının %90'ından fazlası, muhtemelen omurgada bir otoimmün T hücre reaksiyonunu tetikleyen bu alele sahiptir. Bağışıklık sistemi, dost ateşinden dolayı dokulara zarar vermeden etkili bağışıklık sağlamak arasında bıçak sırtında çalışır.

Slayt 20

T hücresi otoimmünitesi faydalı da olabilir. Kanser hücreleri tipik olarak normal hücresel peptitlerden farklı peptitlerin üretilmesine yol açan birçok mutasyona ve diğer değişikliklere sahiptir. Kanser immünoterapisi, kanser hücrelerini öldürmek için virüs ve bakteriyel enfeksiyonları tanımada bağışıklık sistemi tarafından kullanılan mekanizmalardan yararlanır.

Slayt 21

Peki ya HLA-DR, HLA-DQ ve HLA-DP sınıf II MHC molekülleri? Bu moleküller, daha sonra B hücrelerinin antikor üreten fabrikalara farklılaşmasına yardımcı olmak için sitokinler üreten yardımcı T hücrelere patojen kökenli peptitler sunar. Yardımcı T hücreler ayrıca öldürücü T hücrelerin tepkilerini optimize etmeye yardımcı olur. sınıf II MHC, şekil olarak sınıf I MHC'ye oldukça benzer, ancak hücrelerin dışından alınan proteinleri parçalayan küçük organeller olan lizozomlarda yapılan daha uzun protein parçacıklarını sunar.

Slayt 22

Bunu nasıl yapıyorlar? sınıf II MHC (hücrenin dış zarına veya lizozomlarına gitmesi gereken diğer herhangi bir protein gibi) endoplasmik retikulumda yapılır. Burada bir peptidi taklit eden ve sınıf II MHC'yi lizozoma yönelten bir proteine (değişmez zincir'e) bağlanır. Değişmez zincir lizozomda çıkarılır ve lizozom enzimleri tarafından oluşturulan bir peptit ile değiştirilir. Bu süreç, başka bir MHC molekülü türü tarafından optimize edilir. Bu molekül sınıf II MHC'ye benzeyen HLA-DM'dir ve bazı hücrelerde başka bir sınıf II benzeri molekül olan HLA-DO ile uyum içinde çalışır. Evrim tembeldir, çalışan bir mekanizma geliştirdiğinde, yeni işlevler için basitçe bu mekanizmaları kopyalar. Bu karmaşık dansın net sonucu, sınıf II MHC moleküllerinin yardımcı T hücre aktivasyonunu sağlayan peptitlerle hücre yüzeyine verilmesidir.

Slayt 23

Bağışıklık sistemi tarafından patojen tanıma süreci karmaşıktır... ama aynı zamanda nispeten yavaştır. Bir virüsle ilk karşılaştığınızda, bağışıklık sisteminin anti-virüs yanıtı hızlandırması zaman alır. Şanssızsanız, bu, kontrolsüz virüs çoğalmasından dolayı hastalık veya ölümle sonuçlanabilir. Aşı, bağışıklık sistemini bir enfeksiyona hazırlar, bazı durumlarda enfeksiyonu tamamen önlemesini ve aksi takdirde daha hızlı ve etkili bir şekilde yanıt vermesini sağlar ve ciddi bir enfeksiyon olasılığını büyük ölçüde azaltır.

Slayt 24

MHC molekülleri aşılarda kritik rolü vardır. Tüm aşılar, antikor yanıtları için gerekli olan yardımcı T hücrelerini indüklemek ve antikor yanıtlarının yönlendirildiği proteinleri yapmak için sınıf II MHC moleküllerini kullanır. Adenovirüs ve mRNA aşıları da öldürücü T hücreleri indüklemek için sınıf I MHC moleküllerini kullanırlar. Aşıların neden olduğu T hücreleri, orijinal virüsle yeni bir enfeksiyona karşı tetikte olarak yıllarca, hatta bazı durumlarda on yıllarca süregelirler. Aşılar, diğer tüm tıbbi müdahalelerin toplamından çok daha fazla hayat kurtardı. Hastalığı değil bu mesajı yayın, aşı olun!

Slayt 25

Böylece MHC molekülleri enfeksiyonları kontrol eder, bağışıklık tepkilerini düzenler ve de kanseri iyileştirmeye yardımcı olur. Bu, otoimmünite ve nakil reddinin dezavantajlarına değer. İşte bu yüzden patojenlerle dolu bir dünyada yaşayan siz bu Çizgi Romanı okumak için hayatta kaldınız. Nasıl daha da iyi hayatta kalınacağına dair daha fazla ayrıntı için lütfen 1-6 alıntılarına bakın.
