# Supplementary material for: HLA molecules in transplantation, autoimmunity and infection control: A comic book adventure
Source: HLA. 2022 May 15;100(4):301–11. doi: 10.1111/tan.14626 (PMC9545814; doi:10.1111/tan.14626)
Supplement: Supplementary file 1 — Supporting information. [file TAN-100-301-s001.zip › Supplementary files/PP_Ukrainian_ Stoianova.1.pdf]

Молекули HLA в трансплантації, автоімунітеті та інфекційному контролі: пригодницький комікс

HLA molecules in transplantation, autoimmunity and infection control.
A comic Book adventure

by Eric Reits and Jacques Neefjes

Translated by cand. med Anastasiia Stoianova. *Original text :* ***https://doi.org/10.1111/tan.14626***

Department of Cell and Chemical Biology, ONCODE Institute, Leiden University Medical Centre LUMC, The Netherlands

Slide 1

Близько 1900 років тому двоє братів-лікарів з Аравії Косма і Даміан провели одному купцю з гангреною першу відому в світі трансплантацію і пересадили йому ногу його раба. Доля раба залишилась в історії невідомою, але навряд чи це була добровільна пожертва.

Slide 2

Ця «чудесна» трансплантація сприяла зарахуванню братів до лику блаженних і надала статус покровителей трансплантації.

Що, однак, не зберегло їм голову, яку відтяли за християнську віру і, звичайно, повернули на місце після вознесіння на небо.

Slide 3

Чому трансплантація така складна, які тут існують еволюційні фактори?

Навіть Дарвін, мабуть, ставив собі ці питання…

Однак він і гадки не мав про унікальний клас білків, який містять у собі майже всі багатоклітинні еукаріотичні організми.

Slide 4

Почнімо з теперішнього розуміння двох унікальних класів протеїнів в нашому організмі; саме тих, що мають найбільший ступінь *поліморфізму* (варіацій між особинами).

Саме в цьому і полягає їхня винятковість, оскільки більшість інших білків схожі між собою у різних людей.

Ці поліморфні білки є «трансплантаційними антигенами» і називаються молекулами головного класу гістосумісності I та II класів (англ. major histocompatibility complex, MHC).

У людей їх називають лейкоцитарні антигени людини класу 1 і HLA класу 2 (англ. human leukocyte antigen, HLA; в обох випадках далі збережена англомовна абревіатура).

Slide 5

Для трансплантації найважливішими молекулами HLA класу 1 є HLA-A, HLA-B і HLA-C, а HLA класу 2 -- HLA-DR, HLA-DQ і HLA-DP.

HLA-A, -B і -C присутні практично на всіх наших клітинах (крім без`ядерних еритроцитів), тоді як HLA-DR, HLA-DQ і HLA-DP розташовані в основному на представниках імунітету.

Slide 6

Молекули HLA настільки поліморфні, що вагітні жінки часто виробляють антитіла до незнайомих їм HLA-антигенів батька.

Це можна було б використати для визначення батьківства задовго до того, як генетичне тестування стало мейнстримом.

Раніше сироватки вагітних використовували у трансплантології тканин. На наукових зустрічах лабораторії обмінювались цими сироватками і згодом називали різні сироваткові реакції.

Так були ідентифіковані HLA-A, -B і -C, а також їхні субформи. На той час їх просто пронумерували починаючи з HLA-A1, далі HLA-A2 тощо. Це також трапилось з молекулами другого класу: HLA-DR, -DQ та -DP.

Text on the slide: і батьком в нас буде…

Slide 7

Сьогодні HLA-типування зазвичай проводиться за допомогою аналізу ДНК. Існують наукові свідчення, що жінки можуть розрізняти різноманітні HLA чоловіків за запахом, що сприяє відбору генетично відмінних партнерів.

Text on the slide:

- *Я HLA-A2, 3, B7, 44, Cw5, w7, DR4, 15, DQ3, 6, DPw3, w4*
- *але я HLA-A1, 3, B8, 65, Cw7, w8, DR1, 3, DQ2, 5, DPw1, w3*

Slide 8

Хоча поліморфізм HLA допомагає урізноманітнити геном людства, він є величезним бар’єром для успішної трансплантації органів, яка вимагає якомога точнішого збігу HLA-антигенів реципієнта та донора.

За відсутності ідеального збігу застосовуються ефективні імуносупресивні препарати для запобігання відторгнення органу.

Text on the slide:

- *Я HLA-A2, B7, Cw7, DR11, DQ7, DPw4*
- *але я HLA-A1, B8,Cw7, DR3, DQ2, DPw1*

Slide 9

Так, Дарвін був би збитий з пантелику.

Звичайно, зустріч з другою половинкою, труднощі при трансплантації тканин чи встановлення батьківства не можуть бути основними еволюційними причинами поліморфізму HLA.

- *Text on the slide: острів трансплантацій*

Slide 10

Але є ще один фактор. Вірусів та інших мікробних патогенів багато в природі. Корона, грип, Ебола, віспа та багато інших вірусів використовують наші клітини як домівку для створення власних родин.

Навіть інфекції зі здатністю до самообмеження були б смертельними без імунної системи.

Питання просте: як імунна система може виявити віруси, що ховаються всередині клітин, щоб вбити їх, перш ніж вони знищать нас?

Slide 11

Щоб не дати вірусам нашкодити нашому організму, імунна система розробила кілька видів зброї.

Макрофаги поїдають бактерії та віруси, нейтрофіли виділяють хімічні речовини, що вбивають бактерії, В-клітини виробляють антитіла, Т-хелпери координують імунну відповідь (в тому числі гуморальну), Т-кілери вбивають інфіковані вірусом клітини (і навіть ракові клітини).

Slide 12

Але як Т-клітина-вбивця знає, в кого цілитись?

Вірус, перебуваючи всередині клітини, ніби хитро замаскувався, чи не так?

Насправді під час реплікації вірусу крихітні шматочки його білків доставляються до молекул HLA-A, -B або -C, які вирушають з ними на мембрану клітини. Т-клітина-кілер може розпізнати цей невеликий фрагмент на поверхні лише СВОЄЇ специфічної для пептиду молекули HLA.

Відкриття цього явища, названого HLA-рестрикцією, було досить важливим, щоб отримати дві Нобелівські премії.

Кожен різний тип молекули MHC класу 1 презентує імунним клітинам різний репертуар пептидів і таким чином дає імунній системі безліч мішеней для прицілу і пострілу.

Text on the slide:

- *чим це пахне?*

Slide 13

Але як спочатку створюються такі шматочки вірусу?

Вірусні білки, як і будь-який інший білок всередині клітин, руйнуються.

Білки нарізаються за допомогою чудової наномашини під назвою *протеасома*, яка в основному виконує команду “ЗнищитиВсе” щодо білків.

Інші ферменти клітини фрагментують білки на ще менші пептиди, а ті транспортуються з цитозолю в ендоплазматичний ретикулум і зв’язуються з молекулами HLA.

Як тільки молекула HLA зловила пептид, вона покидає ендоплазматичну сітку і прямує на поверхню клітини, де очікує на візит Т-клітин-вбивць (також названих цитотоксичними клітинами, ЦТК).

Text on the slide:

- *protein* — білок, *proteasome — протеасома, peptides*
- —пептиди, *MHC class I* — МНС класу 1, *TAP (stays the same), CTL* — ЦТК

Slide 14

Повернемося до поліморфізму HLA.

Як добре відомо з пандемій COVID-19 та грипу, віруси постійно мутують, щоб уникнути реакції антитіл (згадайте альфа, дельта, омікрон та інші варіанти SARS-CoV-2…).

Щоб мінімізувати цей неприємний ефект для Т-клітин, кожен з різних алелей MHC (алель — один з варіантів гена) представляє різний набір пептидів.

В організмі однієї людини презентується стільки різних білків, що вірусу важко уникнути атаки імунної системи.

Таке варіювання HLA між людьми означає, що навіть дуже кмітливий вірус не завжди зможе обдурити організм наступної людини.

Якби ми всі мали ідентичні HLA, ось такий хитромудрий знищив би всю популяцію, але в реальності він вб’є «лише» кількох людей з молекулами HLA, які не можуть представити вірусні пептиди імунній системі. Таким чином, поліморфізм HLA захищає саму популяцію, а не конкретну особину. Це переконливе пояснення еволюції поліморфізму MHC, погодьтесь.

Slide 15

На жаль, є і погані новини для вас, шановний читачу, у випадку якщо вам знадобиться той чи інший новий орган. Поліморфізм HLA сприяє виживанню популяції, однак зовсім не конкретного пацієнта з захворюванням нирок.

Відторгнення трансплантата – це наслідок того, що імунна система плутає донорський орган з інфікованим вірусом органом і реагує відповідно до ситуації, що призводить до втрати трансплантата.

Slide 16

Важливий урок життя: ніщо у світі, імунітет включно, не є ідеальним!

Тут буде якраз доречно подумати про те, як Т-кілери можуть знаходити інфіковані вірусом клітини достатньо прудко, щоб це мало сенс для виживання.

Віруси можуть розмножуватись блискавично швидко, в деяких випадках всього за кілька годин.

Однак це занадто повільно, щоб чекати, поки вірусні білки зруйнуються і стануть доступні імунітету.

На наше щастя, синтез вірусних білків, як і сама імунна система, далекий від досконалості.

Ці неповноцінні білки, які називаються дефектними продуктами рибосом (англ. defective ribosomal products, DRiPs), утворюються миттєво, що на самому початку вірусної інфекції дає можливість презентувати вірусні антигени та дати Т-кілерам виконувати свою роботу.

Slide 17

Шах і мат, думає імунна система? Не так швидко! Деякі розумні віруси, особливо герпесвіруси, вибудували еволюційні механізми, що заважають презентації антигену. Цитомегаловірус (CMV), який носять в собі 60% людства, створює набір білків (US2, US3, US6, US11 і US18), які обмежують вироблення пептидів або заважають функції HLA I класу.

Slide 18

Чи можливо тоді, що носії певних алелів HLA краще справляються з вірусними інфекціями, ніж інші?

Так! Дійсно, деякі алелі HLA-B краще захищають від ВІЛ-інфекції, інші — від SARS-CoV-2. Еволюційна селекція алелів HLA для боротьби з різними патогенами тривала протягом багатьох років.

Наприклад, найбільш поширений у своїй групі алель HLA-A2 зустрічається у 40% населення Європи.

Це, ймовірно, є результатом здатності HLA-A2 захищати від якогось збудника хвороб минулого, який тепер може і не бути основною причиною захворювань людини.

Slide 19

Але є і інший бік медалі. Візьмемо алель HLA-B*27:05. Наявний тільки у 8% європейців, він зустрічається майже у 90% пацієнтів з анкілозивним спондилітом (хворобою Бехтерєва) і, ймовірно, провокує автоімунну реакцію Т-клітин в хребті.

Імунна система ходить по лезу, намагаючись зберегти ефективну імунну відповідь і уникнути пошкодження тканин від дружнього вогню.

Slide 20

Автоімунна відповідь Т-клітин також може бути корисною.

Ракові клітини зазвичай містять багато мутацій та володіють іншими трюками, які призводять до утворення пептидів, відмінних від звичайних клітинних білків.

В імунотерапії раку для знищення пухлинних клітин використовуються якраз таки звичні механізми імунного розпізнавання.

Slide 21

Але як щодо молекул HLA-DR, -DQ та -DP, що відносяться до MHC класу 2?

Ці молекули представляють бактеріальні антигени Т-хелперам (помічникам та посередникам), які потім виробляють сигнальні молекули (цитокіни).

Ці молекули допомагають В-клітинам диференціюватися у фабрики, що виробляють антитіла.

Т-хелпери також допомагають оптимізувати відповідь Т-кілерів.

MHC класу 2 дуже подібні за формою до MHC I класу, але вони презентують довші фрагменти білків, утворені в лізосомах.

Лізосоми є дрібними органелами, які розкладають білки, що потрапили у клітину ззовні.

Slide 22

Як вони це роблять?

Молекули MHC класу 2 виробляються в ендоплазматичному ретикулумі (як і будь-який інший білок, який надходить до зовнішньої мембрани клітини або лізосоми).

Там з MHC-молекулою зв’язується білок під назвою “інваріантний ланцюг” (invariant chain), який виконує роль тимчасового антигену і супроводжує (з франц. chaperone — супроводжувати) MHC II класу дорогою до лізосоми.

Там інваріантний ланцюг видаляється і замінюється на пептид, утворений завдяки лізосомним ферментам.

Цей процес оптимізується ще одним типом молекули MHC (HLA-DM, яка виглядає схожою на MHC II класу, а в деяких клітинах працює разом з HLA-DO, іншою молекулою класу 2).

Еволюція лінива: коли вона розробила робочий алгоритм, вона просто копіює та модифікує його. Кінцевим результатом цього складного танцю є доставлення комплексу з MHC класу 2 та пептиду на поверхню клітини, що активує Т-клітин-хелпери.

Text on the slide: extracellular antigens — позаклітинні антигени, presentation — презентування, B-cell — В-клітина, *MHC class II —* MHC класу 2, *Golgi — апарат Гольджи*

Slide 23

Цей процес розпізнавання патогенів імунною системою є складним... і відносно повільним. Коли ви вперше стикаєтеся з вірусом, імунній системі потрібен час, щоб створити противірусну імунну відповідь. І якщо вам не пощастить, ця затримка може призвести до захворювання або смерті в разі неконтрольованої реплікації вірусу.

Вакцинація готує імунну систему до інфекції, що дає змогу в деяких випадках повністю запобігти зараженню, а в інших — реагувати швидше та ефективніше і значно знизити ймовірність серйозної інфекції.

Slide 24

Молекули MHC беруть важливу участь у відповіді на вакцинацію. Усі вакцини використовують молекули MHC класу 2 для стимуляції Т-клітин-хелперів, необхідних для гуморальної імунної відповіді і створення білків-мішеней для антитіл.

Аденовірусні та мРНК-вакцини також використовують молекули MHC I класу для стимуляції Т-клітин-кілерів.

Т-клітини пам’яті, що відреагували на вакцинацію, зберігаються в організмі багато років, у деяких випадках навіть протягом десятиліть, насторожі нового зараження вірусом, який був в нашому організмі давним-давно.

Вакцини врятували набагато більше життів, ніж усі інші медичні втручання разом узяті.

Поширюйте це повідомлення, а не хворобу, вакцинуйтеся!

Epilogue

Таким чином, молекули MHC контролюють інфекцію, регулюють імунні реакції і тепер допомагають вилікувати рак.

Це окупає інший бік медалі з автоімунними хворобами та відторгненням трансплантата.

І саме тому ви — у світі, наповненому патогенами — вижили, щоб прочитати цей комікс.

Щоб дізнатися більше про те, як вижити ще краще, перегляньте посилання.
