# Supplementary material for: HLA molecules in transplantation, autoimmunity and infection control: A comic book adventure
Source: HLA. 2022 May 15;100(4):301–11. doi: 10.1111/tan.14626 (PMC9545814; doi:10.1111/tan.14626)
Supplement: Supplementary file 1 — Supporting information. [file TAN-100-301-s001.zip › Supplementary files/PP_Welsh_Willams.1.pptx]

## Slide 1
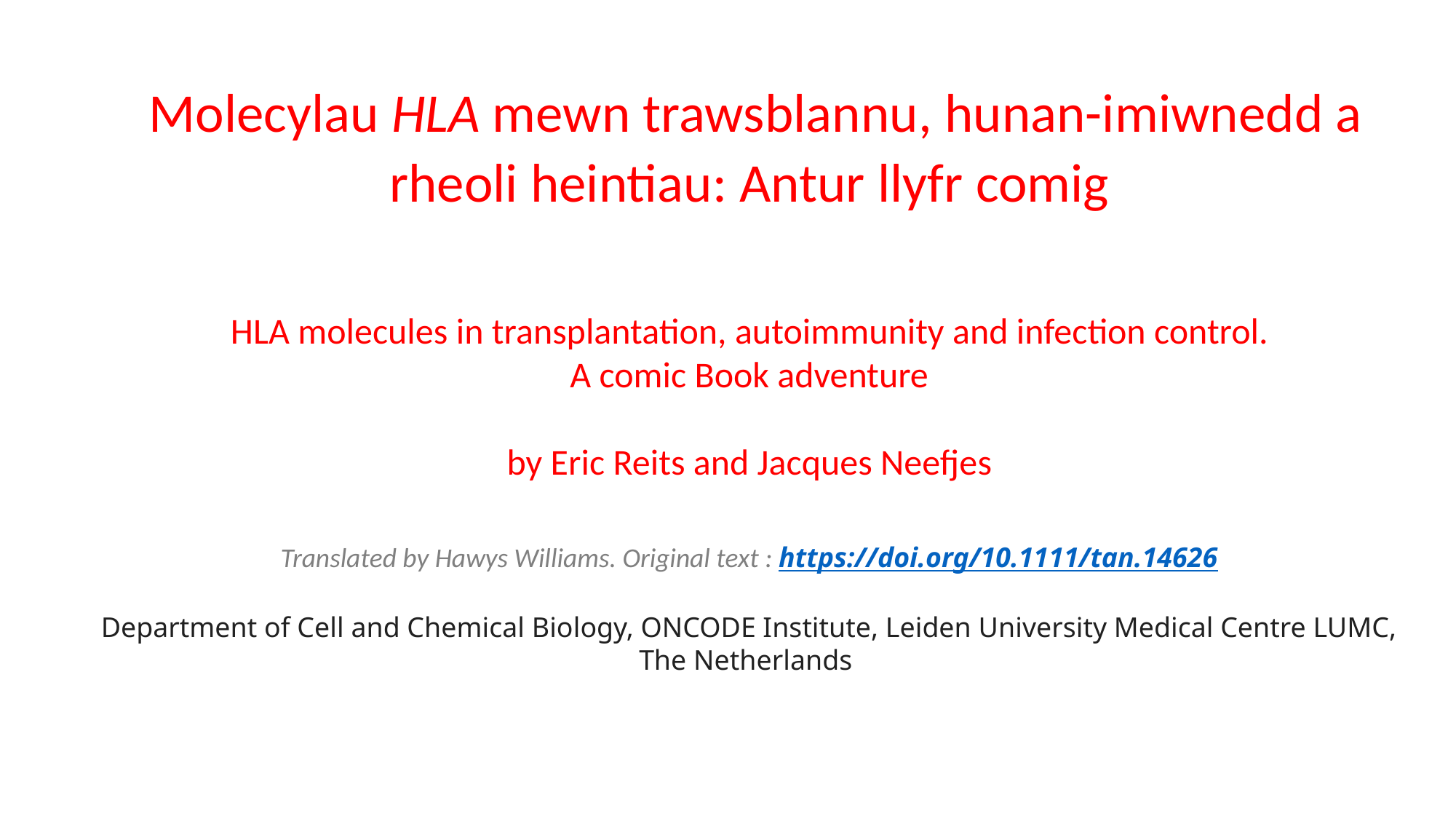

Molecylau HLA mewn trawsblannu, hunan-imiwnedd a rheoli heintiau: Antur llyfr comig
HLA molecules in transplantation, autoimmunity and infection control.
A comic Book adventure
by Eric Reits and Jacques Neefjes
Translated by Hawys Williams. Original text : https://doi.org/10.1111/tan.14626
Department of Cell and Chemical Biology, ONCODE Institute, Leiden University Medical Centre LUMC, The Netherlands

## Slide 2
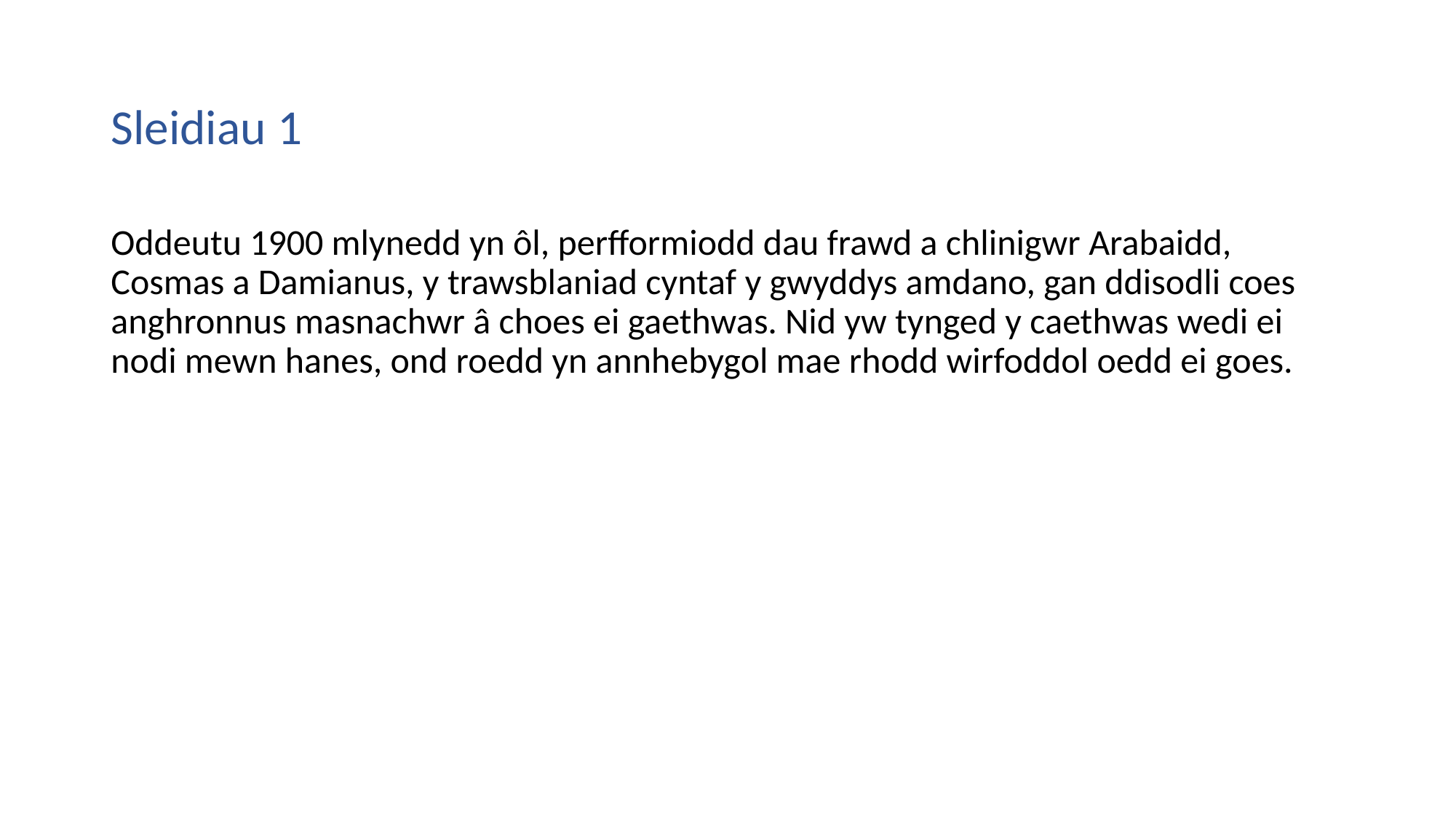

# Sleidiau 1
Oddeutu 1900 mlynedd yn ôl, perfformiodd dau frawd a chlinigwr Arabaidd, Cosmas a Damianus, y trawsblaniad cyntaf y gwyddys amdano, gan ddisodli coes anghronnus masnachwr â choes ei gaethwas. Nid yw tynged y caethwas wedi ei nodi mewn hanes, ond roedd yn annhebygol mae rhodd wirfoddol oedd ei goes.

## Slide 3
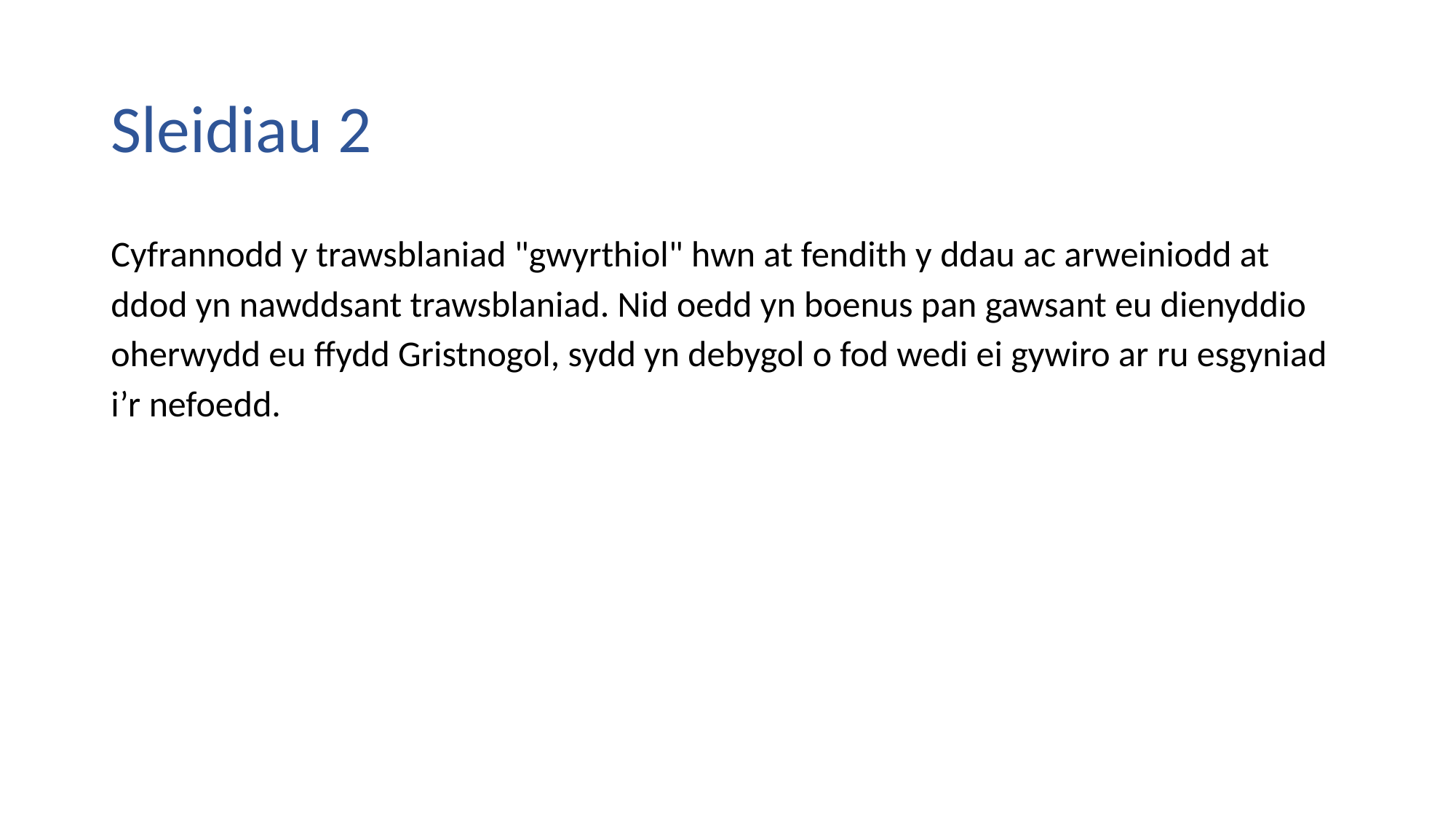

# Sleidiau 2
Cyfrannodd y trawsblaniad "gwyrthiol" hwn at fendith y ddau ac arweiniodd at ddod yn nawddsant trawsblaniad. Nid oedd yn boenus pan gawsant eu dienyddio oherwydd eu ffydd Gristnogol, sydd yn debygol o fod wedi ei gywiro ar ru esgyniad i’r nefoedd.

## Slide 4
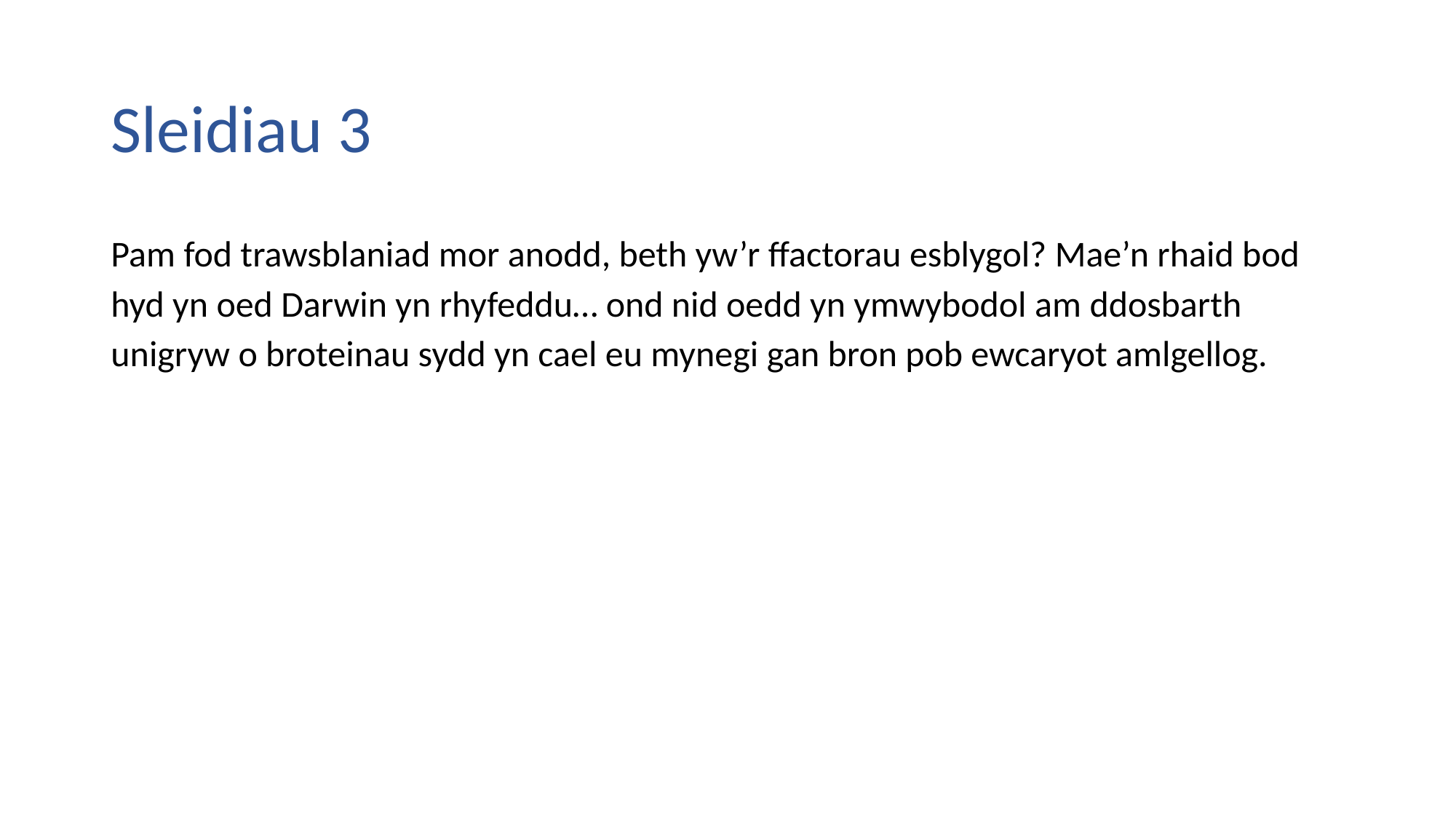

# Sleidiau 3
Pam fod trawsblaniad mor anodd, beth yw’r ffactorau esblygol? Mae’n rhaid bod hyd yn oed Darwin yn rhyfeddu… ond nid oedd yn ymwybodol am ddosbarth unigryw o broteinau sydd yn cael eu mynegi gan bron pob ewcaryot amlgellog.

## Slide 5
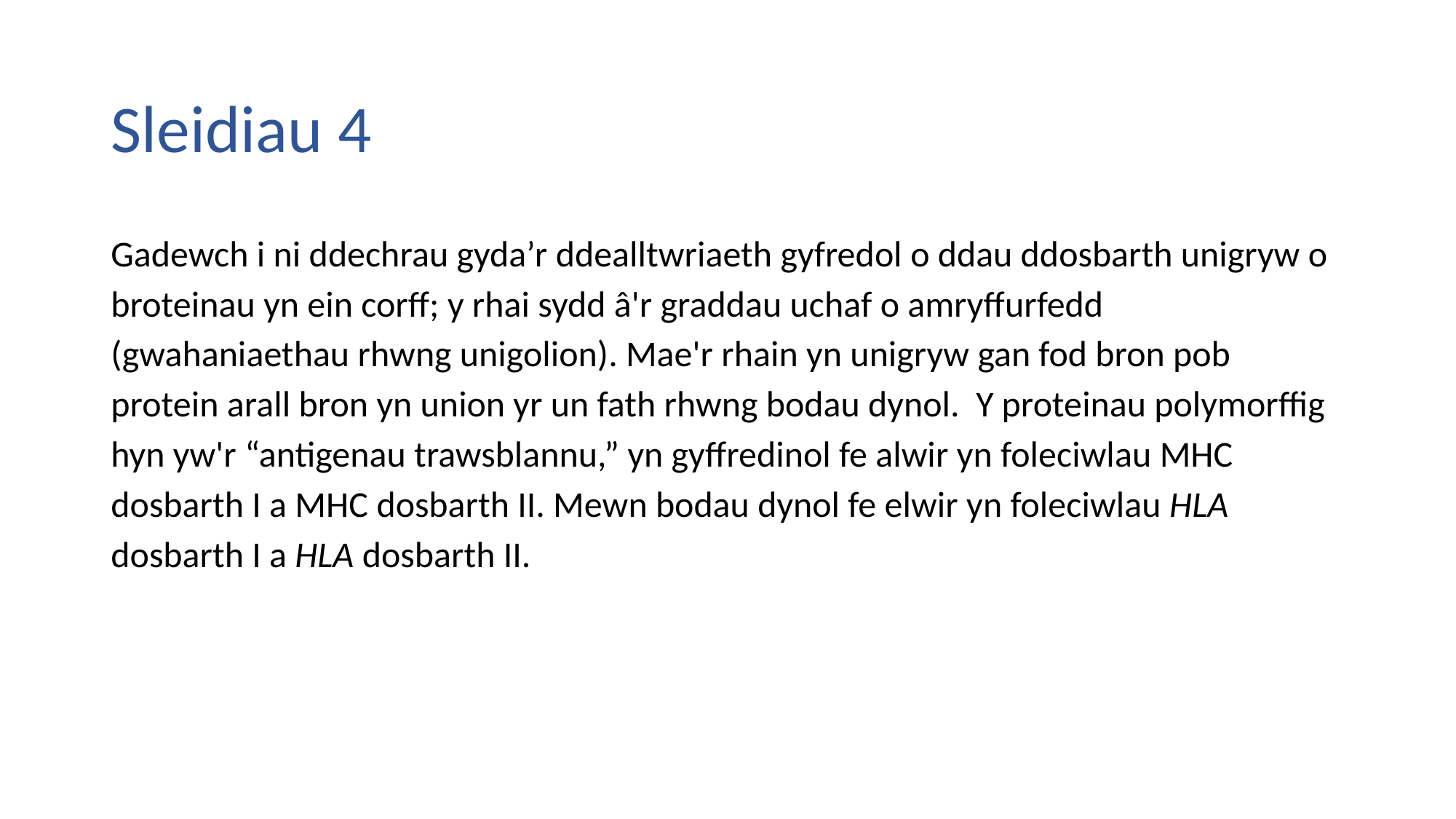

# Sleidiau 4
Gadewch i ni ddechrau gyda’r ddealltwriaeth gyfredol o ddau ddosbarth unigryw o broteinau yn ein corff; y rhai sydd â'r graddau uchaf o amryffurfedd (gwahaniaethau rhwng unigolion). Mae'r rhain yn unigryw gan fod bron pob protein arall bron yn union yr un fath rhwng bodau dynol. Y proteinau polymorffig hyn yw'r “antigenau trawsblannu,” yn gyffredinol fe alwir yn foleciwlau MHC dosbarth I a MHC dosbarth II. Mewn bodau dynol fe elwir yn foleciwlau HLA dosbarth I a HLA dosbarth II.

## Slide 6
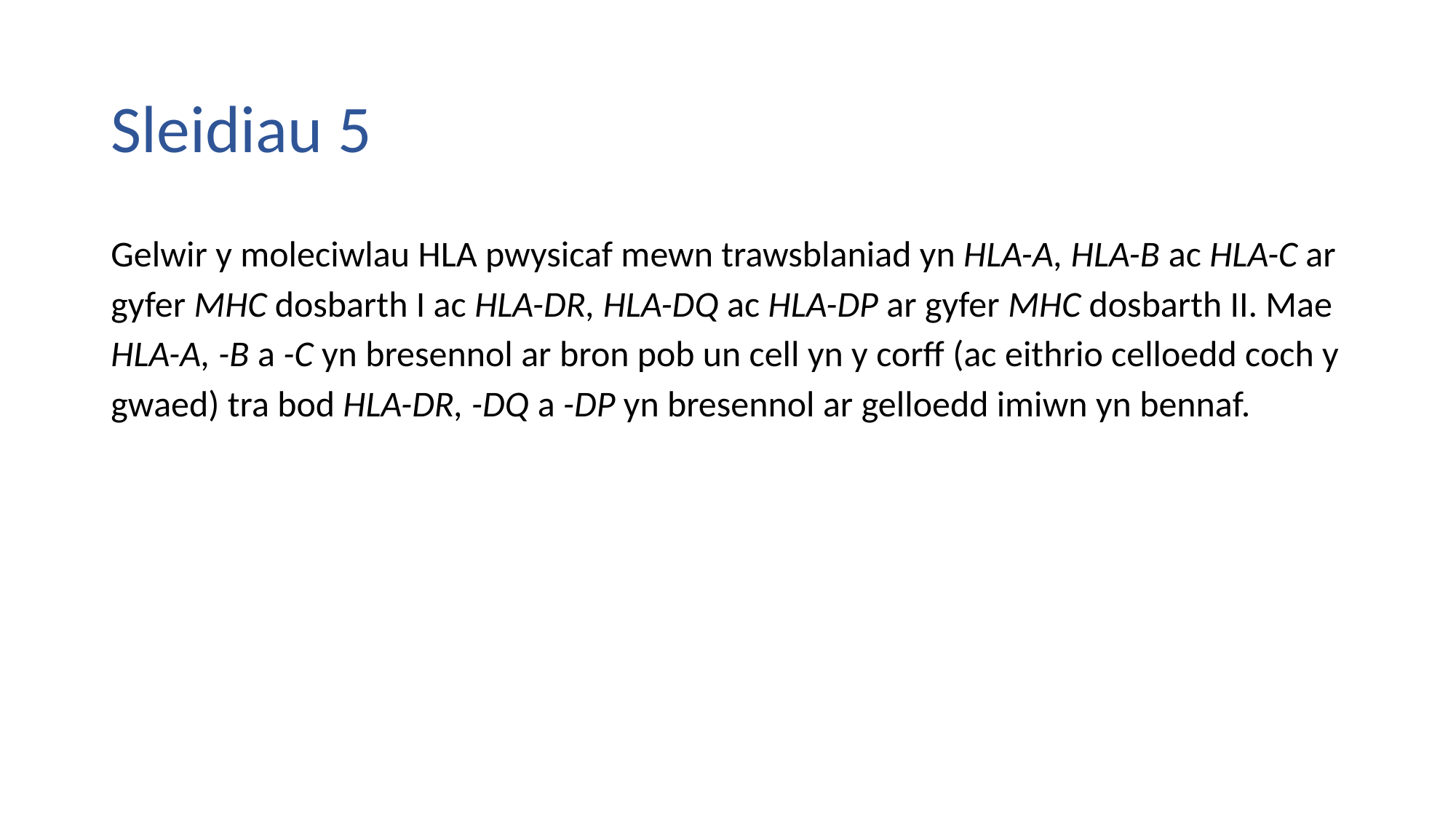

# Sleidiau 5
Gelwir y moleciwlau HLA pwysicaf mewn trawsblaniad yn HLA-A, HLA-B ac HLA-C ar gyfer MHC dosbarth I ac HLA-DR, HLA-DQ ac HLA-DP ar gyfer MHC dosbarth II. Mae HLA-A, -B a -C yn bresennol ar bron pob un cell yn y corff (ac eithrio celloedd coch y gwaed) tra bod HLA-DR, -DQ a -DP yn bresennol ar gelloedd imiwn yn bennaf.

## Slide 7
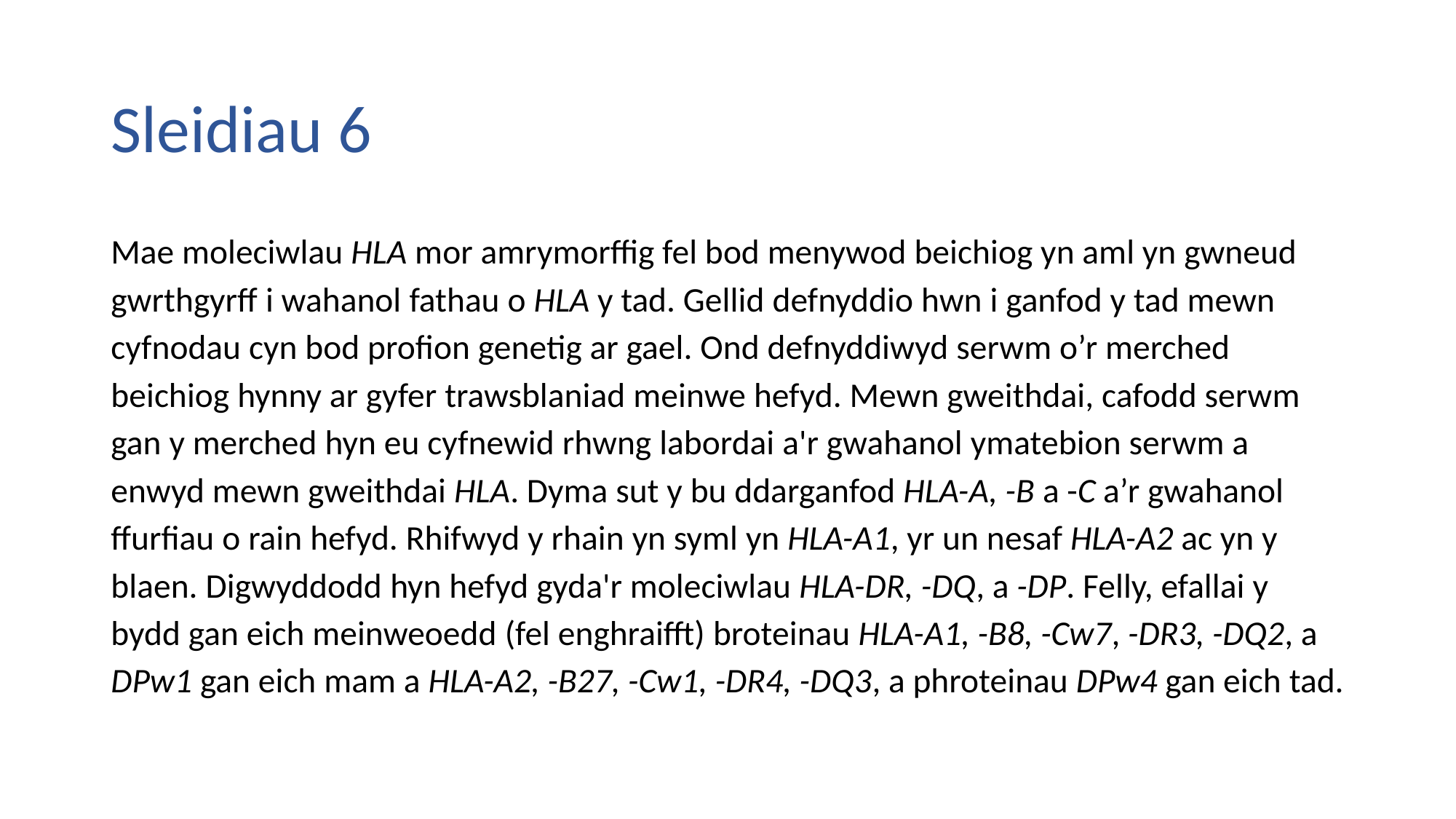

# Sleidiau 6
Mae moleciwlau HLA mor amrymorffig fel bod menywod beichiog yn aml yn gwneud gwrthgyrff i wahanol fathau o HLA y tad. Gellid defnyddio hwn i ganfod y tad mewn cyfnodau cyn bod profion genetig ar gael. Ond defnyddiwyd serwm o’r merched beichiog hynny ar gyfer trawsblaniad meinwe hefyd. Mewn gweithdai, cafodd serwm gan y merched hyn eu cyfnewid rhwng labordai a'r gwahanol ymatebion serwm a enwyd mewn gweithdai HLA. Dyma sut y bu ddarganfod HLA-A, -B a -C a’r gwahanol ffurfiau o rain hefyd. Rhifwyd y rhain yn syml yn HLA-A1, yr un nesaf HLA-A2 ac yn y blaen. Digwyddodd hyn hefyd gyda'r moleciwlau HLA-DR, -DQ, a -DP. Felly, efallai y bydd gan eich meinweoedd (fel enghraifft) broteinau HLA-A1, -B8, -Cw7, -DR3, -DQ2, a DPw1 gan eich mam a HLA-A2, -B27, -Cw1, -DR4, -DQ3, a phroteinau DPw4 gan eich tad.

## Slide 8
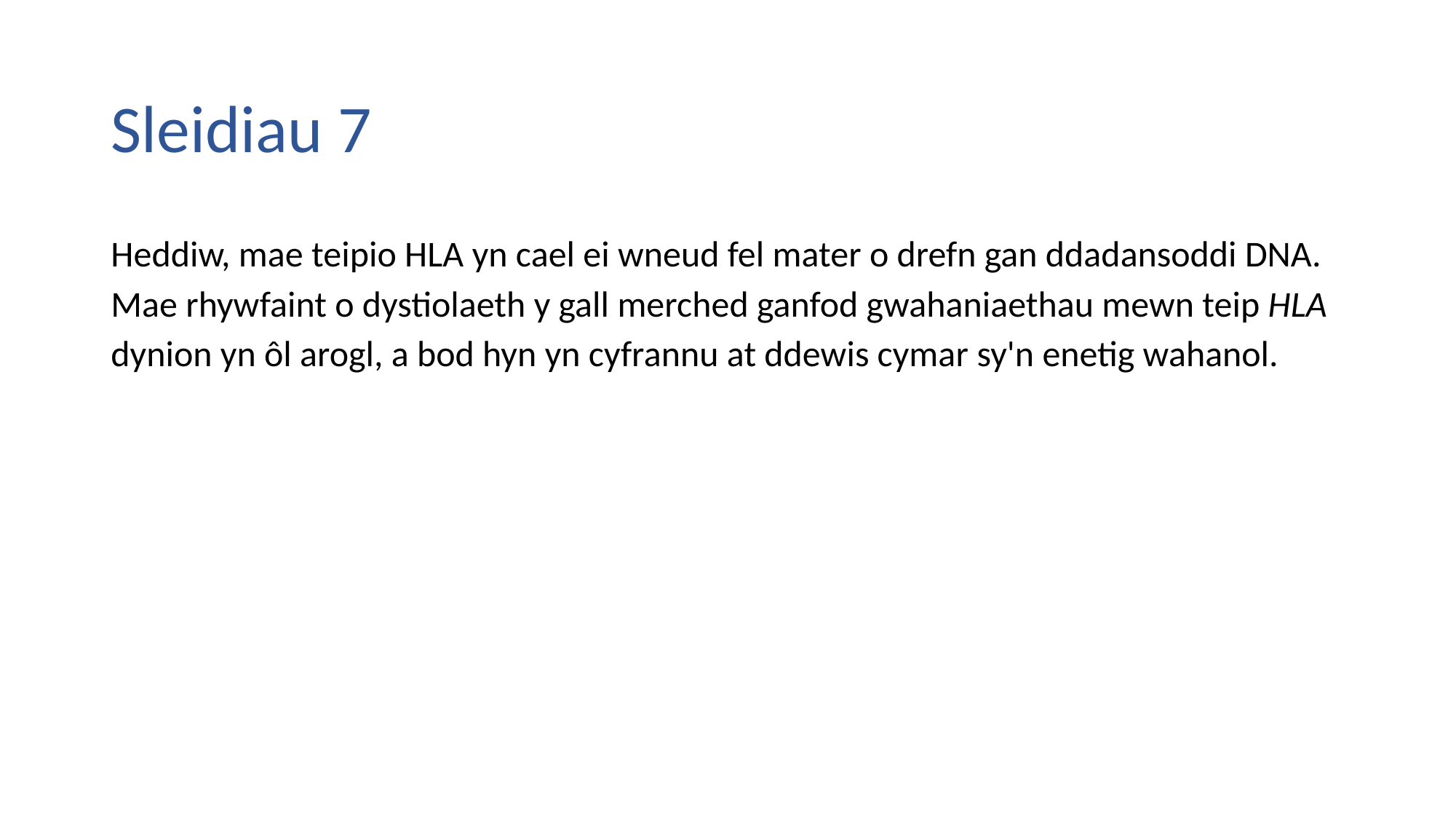

# Sleidiau 7
Heddiw, mae teipio HLA yn cael ei wneud fel mater o drefn gan ddadansoddi DNA. Mae rhywfaint o dystiolaeth y gall merched ganfod gwahaniaethau mewn teip HLA dynion yn ôl arogl, a bod hyn yn cyfrannu at ddewis cymar sy'n enetig wahanol.

## Slide 9
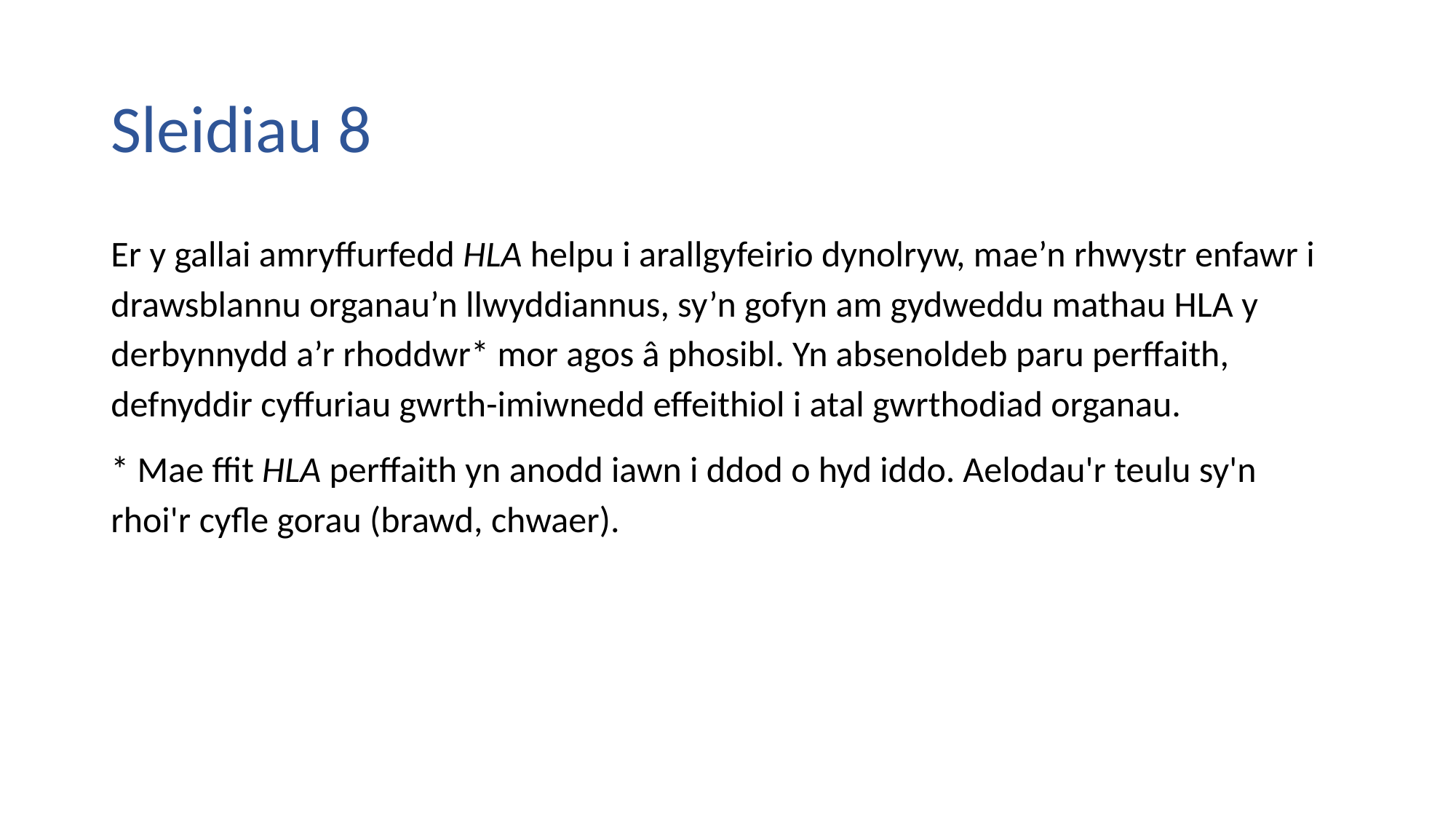

# Sleidiau 8
Er y gallai amryffurfedd HLA helpu i arallgyfeirio dynolryw, mae’n rhwystr enfawr i drawsblannu organau’n llwyddiannus, sy’n gofyn am gydweddu mathau HLA y derbynnydd a’r rhoddwr* mor agos â phosibl. Yn absenoldeb paru perffaith, defnyddir cyffuriau gwrth-imiwnedd effeithiol i atal gwrthodiad organau.
* Mae ffit HLA perffaith yn anodd iawn i ddod o hyd iddo. Aelodau'r teulu sy'n rhoi'r cyfle gorau (brawd, chwaer).

## Slide 10
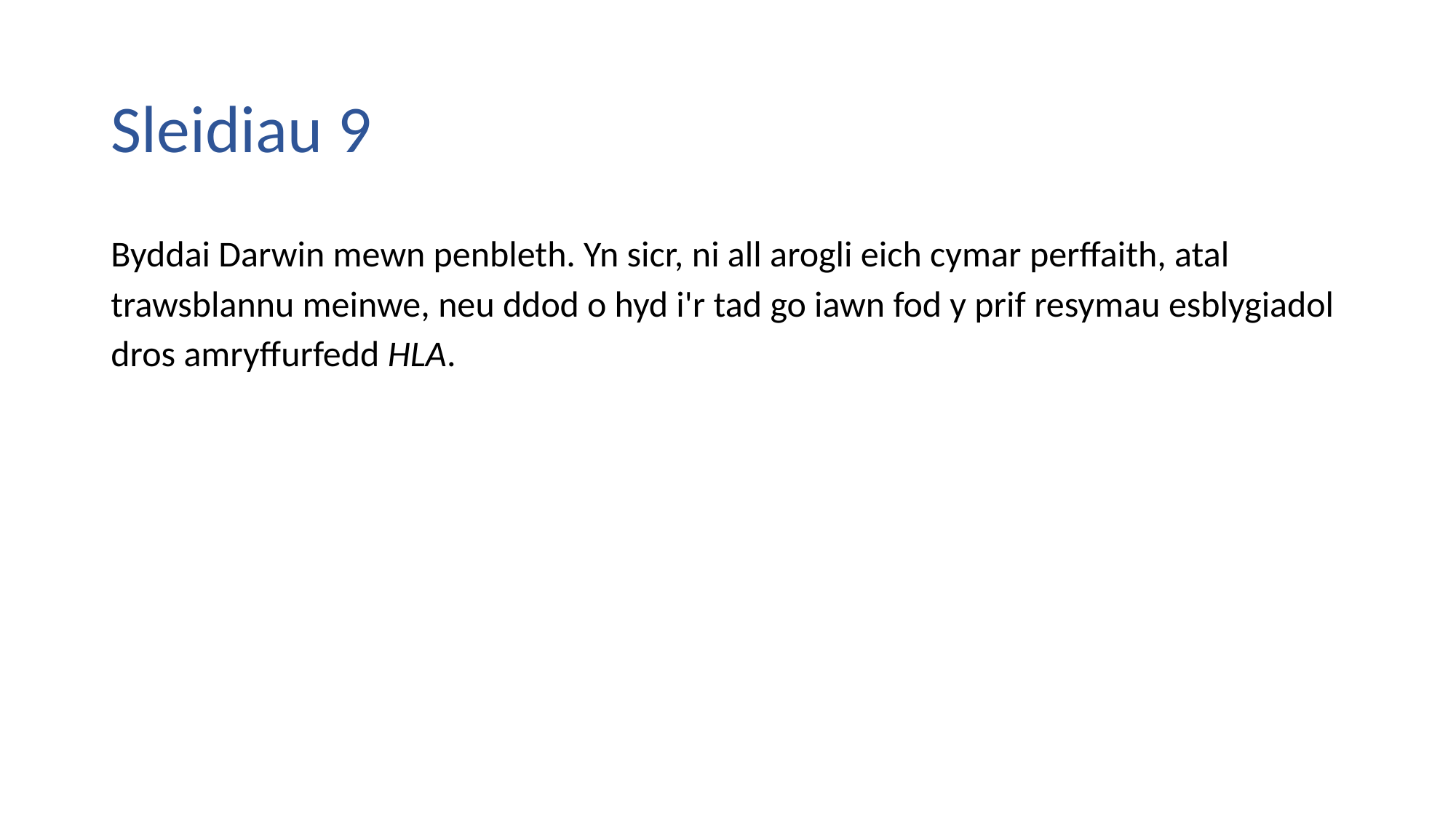

# Sleidiau 9
Byddai Darwin mewn penbleth. Yn sicr, ni all arogli eich cymar perffaith, atal trawsblannu meinwe, neu ddod o hyd i'r tad go iawn fod y prif resymau esblygiadol dros amryffurfedd HLA.

## Slide 11
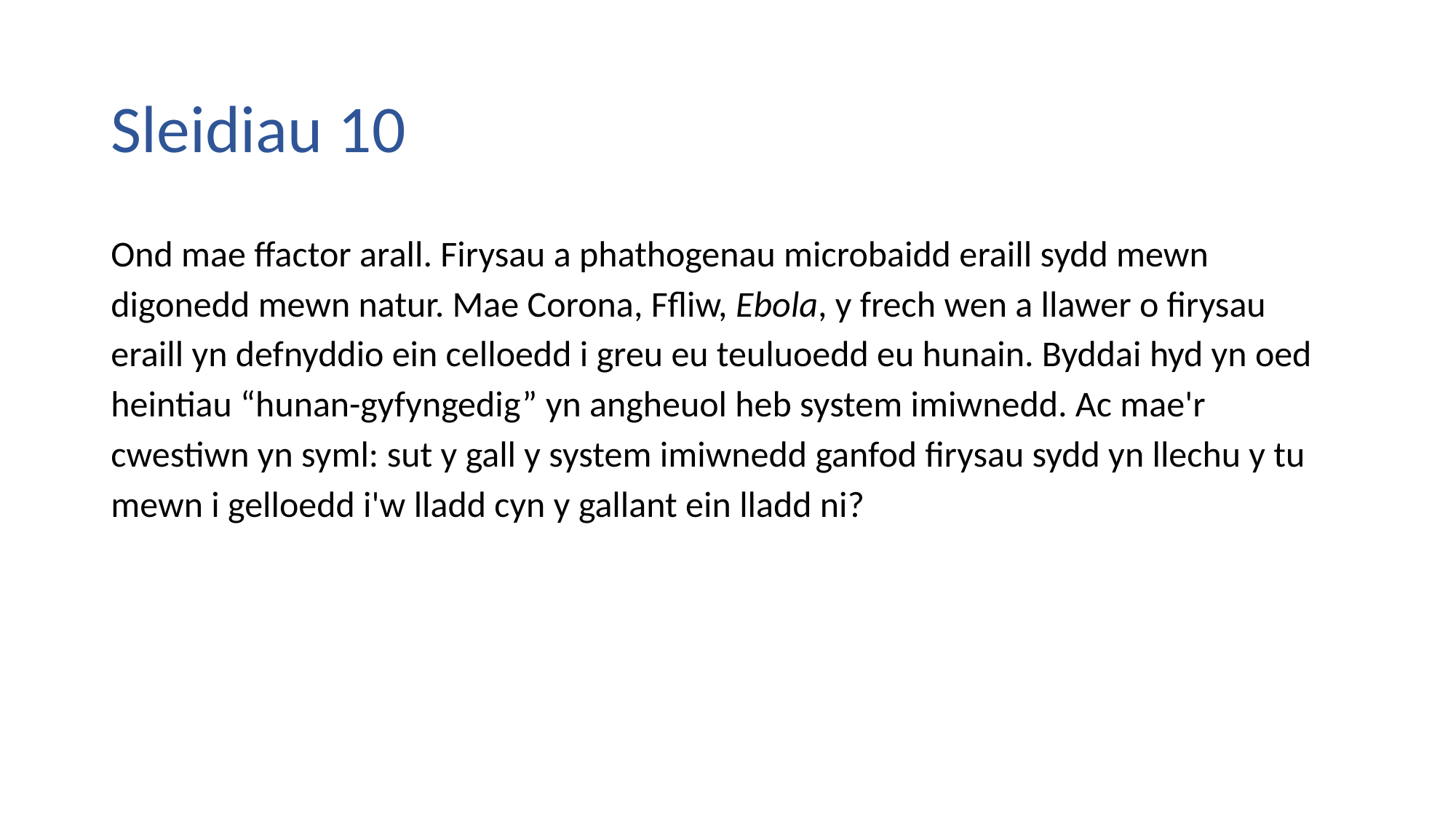

# Sleidiau 10
Ond mae ffactor arall. Firysau a phathogenau microbaidd eraill sydd mewn digonedd mewn natur. Mae Corona, Ffliw, Ebola, y frech wen a llawer o firysau eraill yn defnyddio ein celloedd i greu eu teuluoedd eu hunain. Byddai hyd yn oed heintiau “hunan-gyfyngedig” yn angheuol heb system imiwnedd. Ac mae'r cwestiwn yn syml: sut y gall y system imiwnedd ganfod firysau sydd yn llechu y tu mewn i gelloedd i'w lladd cyn y gallant ein lladd ni?

## Slide 12
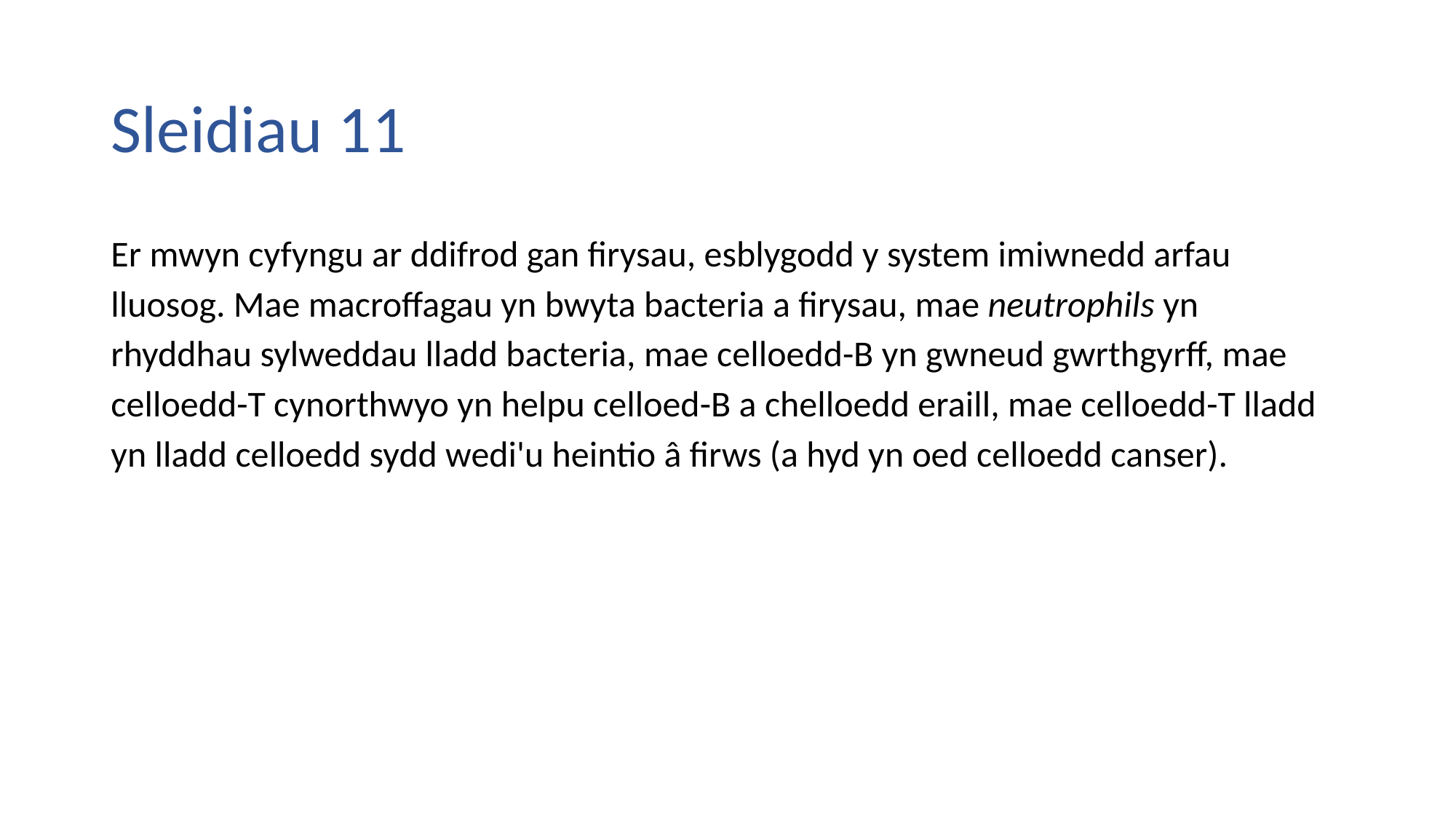

# Sleidiau 11
Er mwyn cyfyngu ar ddifrod gan firysau, esblygodd y system imiwnedd arfau lluosog. Mae macroffagau yn bwyta bacteria a firysau, mae neutrophils yn rhyddhau sylweddau lladd bacteria, mae celloedd-B yn gwneud gwrthgyrff, mae celloedd-T cynorthwyo yn helpu celloed-B a chelloedd eraill, mae celloedd-T lladd yn lladd celloedd sydd wedi'u heintio â firws (a hyd yn oed celloedd canser).

## Slide 13
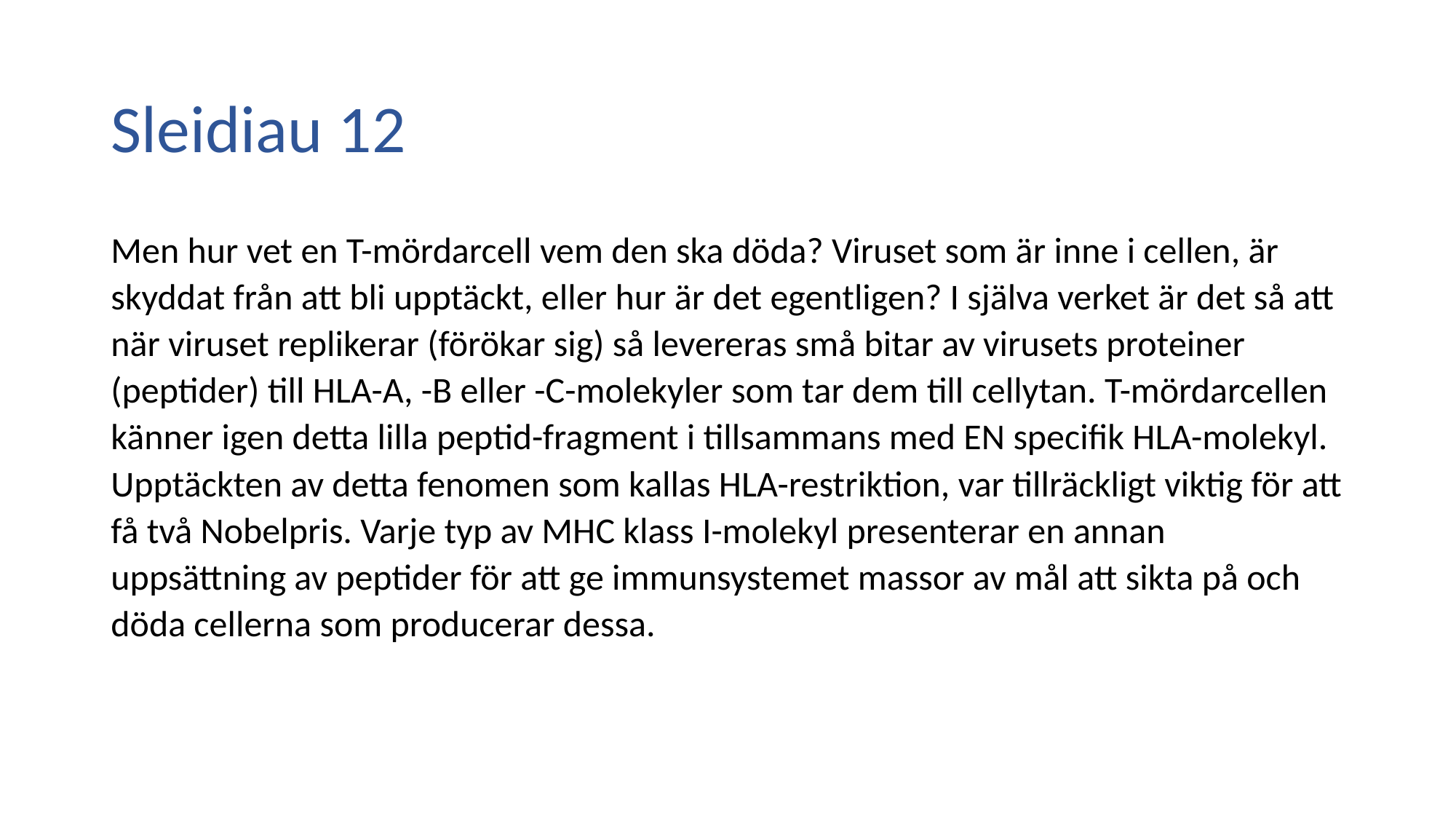

# Sleidiau 12
Men hur vet en T-mördarcell vem den ska döda? Viruset som är inne i cellen, är skyddat från att bli upptäckt, eller hur är det egentligen? I själva verket är det så att när viruset replikerar (förökar sig) så levereras små bitar av virusets proteiner (peptider) till HLA-A, -B eller -C-molekyler som tar dem till cellytan. T-mördarcellen känner igen detta lilla peptid-fragment i tillsammans med EN specifik HLA-molekyl. Upptäckten av detta fenomen som kallas HLA-restriktion, var tillräckligt viktig för att få två Nobelpris. Varje typ av MHC klass I-molekyl presenterar en annan uppsättning av peptider för att ge immunsystemet massor av mål att sikta på och döda cellerna som producerar dessa.

## Slide 14
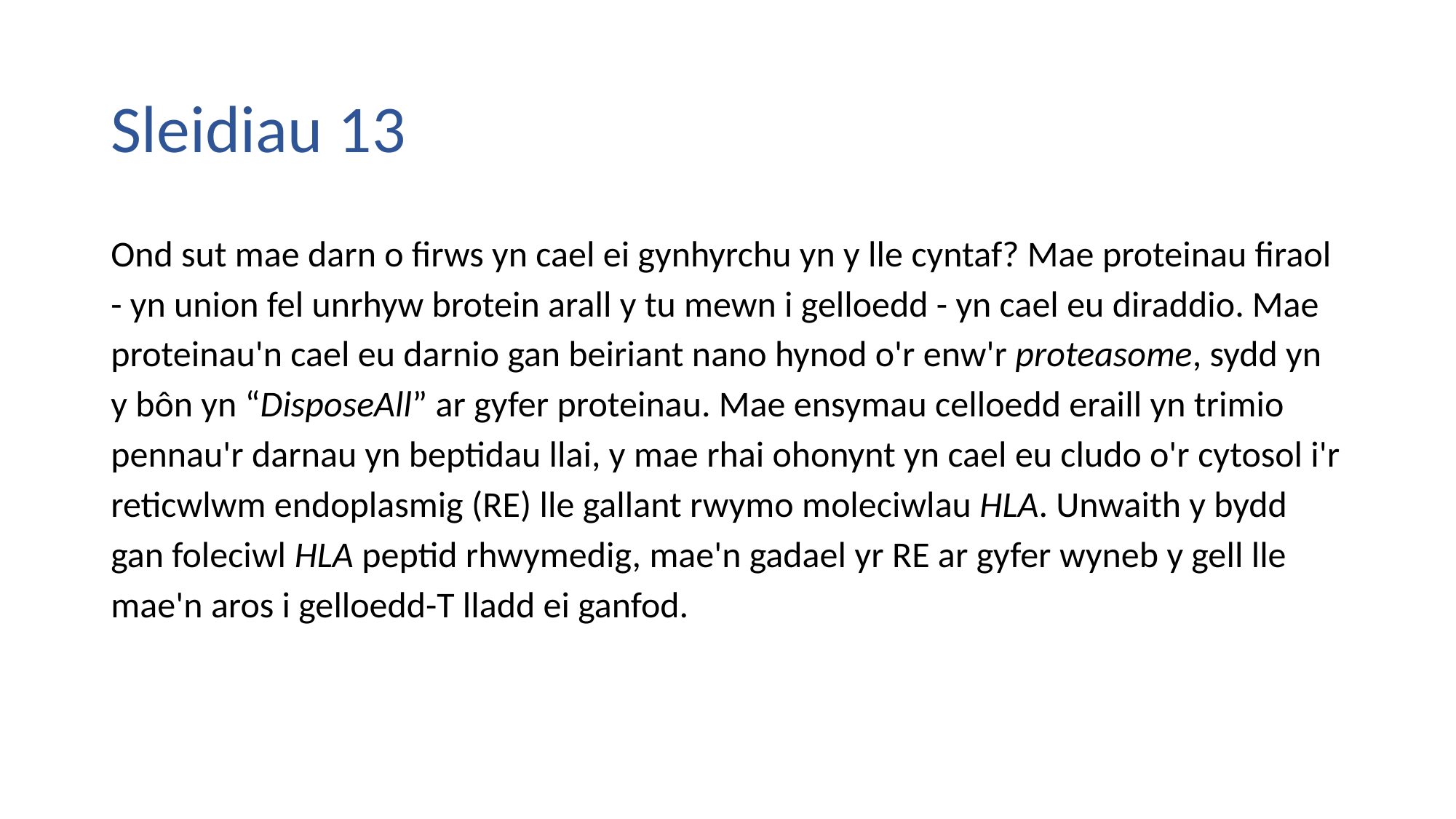

# Sleidiau 13
Ond sut mae darn o firws yn cael ei gynhyrchu yn y lle cyntaf? Mae proteinau firaol - yn union fel unrhyw brotein arall y tu mewn i gelloedd - yn cael eu diraddio. Mae proteinau'n cael eu darnio gan beiriant nano hynod o'r enw'r proteasome, sydd yn y bôn yn “DisposeAll” ar gyfer proteinau. Mae ensymau celloedd eraill yn trimio pennau'r darnau yn beptidau llai, y mae rhai ohonynt yn cael eu cludo o'r cytosol i'r reticwlwm endoplasmig (RE) lle gallant rwymo moleciwlau HLA. Unwaith y bydd gan foleciwl HLA peptid rhwymedig, mae'n gadael yr RE ar gyfer wyneb y gell lle mae'n aros i gelloedd-T lladd ei ganfod.

## Slide 15
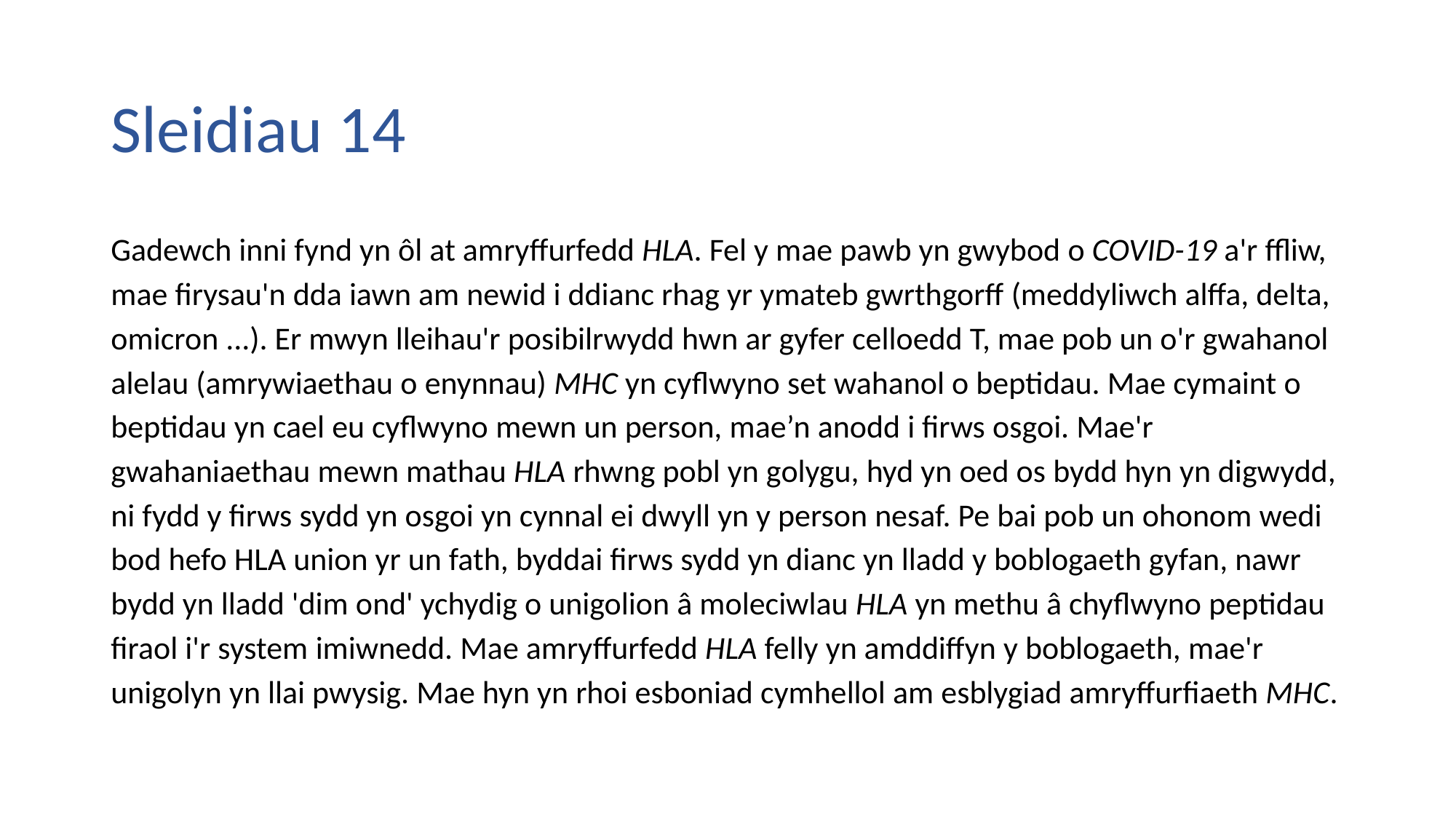

# Sleidiau 14
Gadewch inni fynd yn ôl at amryffurfedd HLA. Fel y mae pawb yn gwybod o COVID-19 a'r ffliw, mae firysau'n dda iawn am newid i ddianc rhag yr ymateb gwrthgorff (meddyliwch alffa, delta, omicron ...). Er mwyn lleihau'r posibilrwydd hwn ar gyfer celloedd T, mae pob un o'r gwahanol alelau (amrywiaethau o enynnau) MHC yn cyflwyno set wahanol o beptidau. Mae cymaint o beptidau yn cael eu cyflwyno mewn un person, mae’n anodd i firws osgoi. Mae'r gwahaniaethau mewn mathau HLA rhwng pobl yn golygu, hyd yn oed os bydd hyn yn digwydd, ni fydd y firws sydd yn osgoi yn cynnal ei dwyll yn y person nesaf. Pe bai pob un ohonom wedi bod hefo HLA union yr un fath, byddai firws sydd yn dianc yn lladd y boblogaeth gyfan, nawr bydd yn lladd 'dim ond' ychydig o unigolion â moleciwlau HLA yn methu â chyflwyno peptidau firaol i'r system imiwnedd. Mae amryffurfedd HLA felly yn amddiffyn y boblogaeth, mae'r unigolyn yn llai pwysig. Mae hyn yn rhoi esboniad cymhellol am esblygiad amryffurfiaeth MHC.

## Slide 16
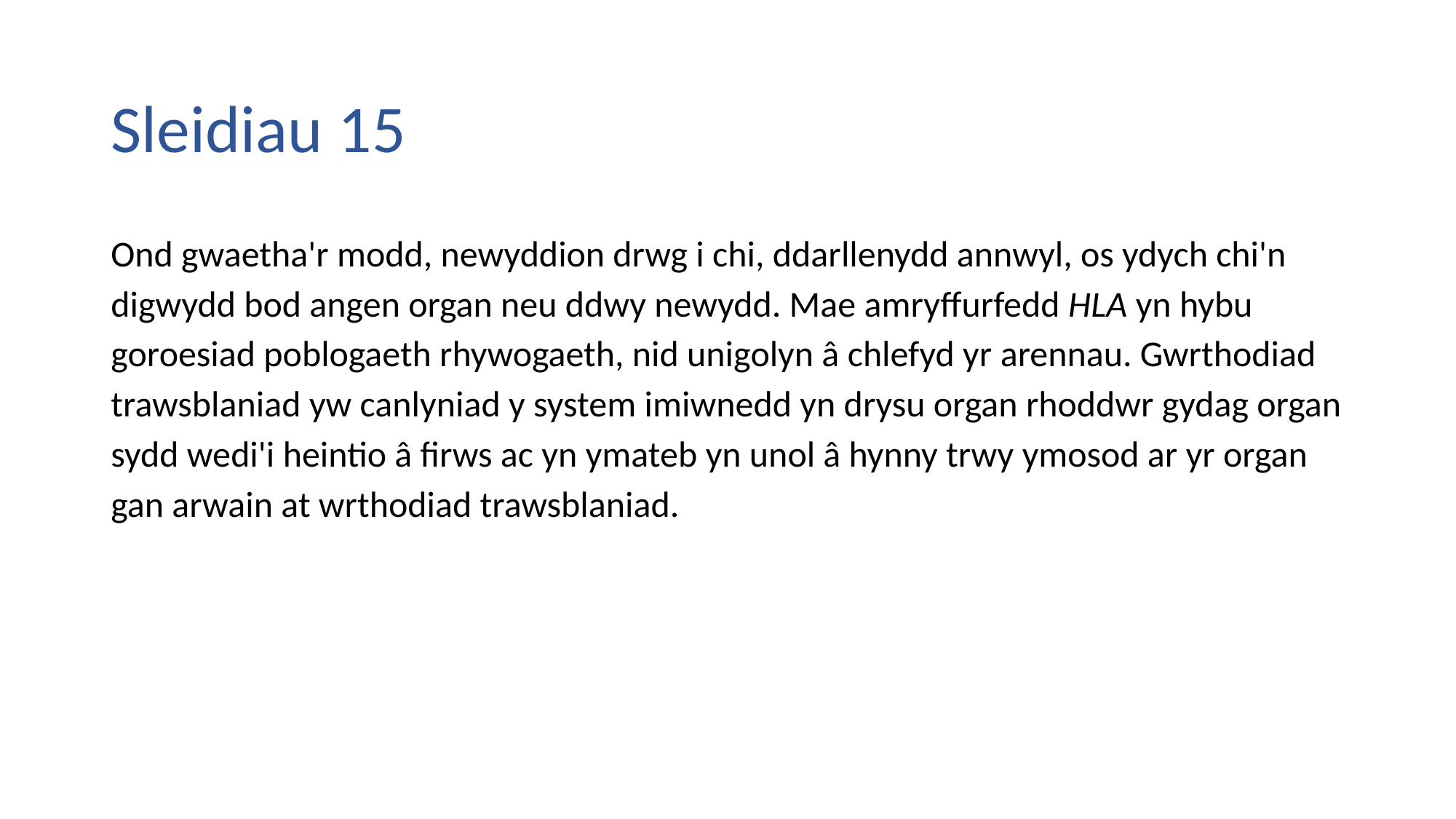

# Sleidiau 15
Ond gwaetha'r modd, newyddion drwg i chi, ddarllenydd annwyl, os ydych chi'n digwydd bod angen organ neu ddwy newydd. Mae amryffurfedd HLA yn hybu goroesiad poblogaeth rhywogaeth, nid unigolyn â chlefyd yr arennau. Gwrthodiad trawsblaniad yw canlyniad y system imiwnedd yn drysu organ rhoddwr gydag organ sydd wedi'i heintio â firws ac yn ymateb yn unol â hynny trwy ymosod ar yr organ gan arwain at wrthodiad trawsblaniad.

## Slide 17
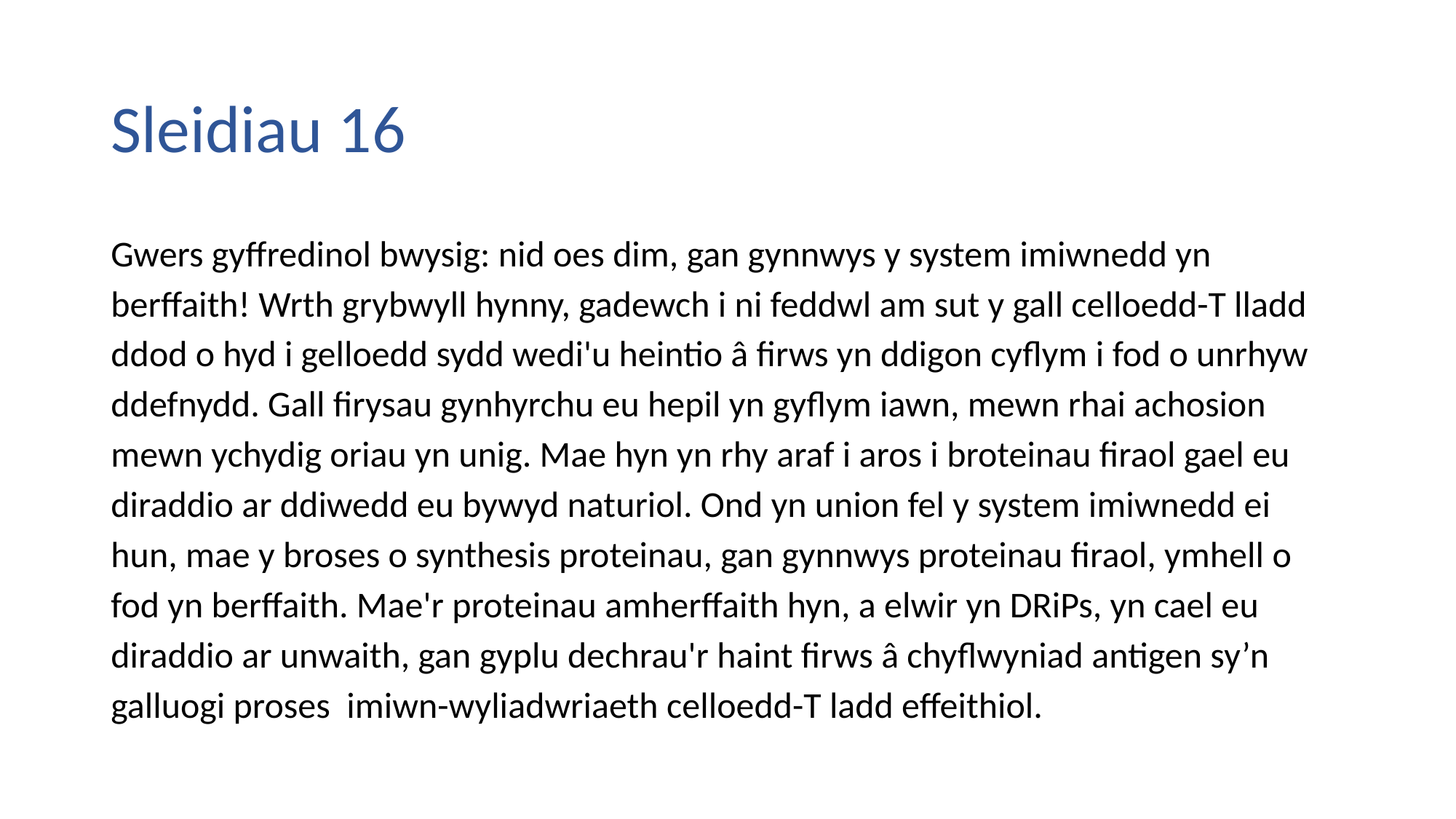

# Sleidiau 16
Gwers gyffredinol bwysig: nid oes dim, gan gynnwys y system imiwnedd yn berffaith! Wrth grybwyll hynny, gadewch i ni feddwl am sut y gall celloedd-T lladd ddod o hyd i gelloedd sydd wedi'u heintio â firws yn ddigon cyflym i fod o unrhyw ddefnydd. Gall firysau gynhyrchu eu hepil yn gyflym iawn, mewn rhai achosion mewn ychydig oriau yn unig. Mae hyn yn rhy araf i aros i broteinau firaol gael eu diraddio ar ddiwedd eu bywyd naturiol. Ond yn union fel y system imiwnedd ei hun, mae y broses o synthesis proteinau, gan gynnwys proteinau firaol, ymhell o fod yn berffaith. Mae'r proteinau amherffaith hyn, a elwir yn DRiPs, yn cael eu diraddio ar unwaith, gan gyplu dechrau'r haint firws â chyflwyniad antigen sy’n galluogi proses imiwn-wyliadwriaeth celloedd-T ladd effeithiol.

## Slide 18
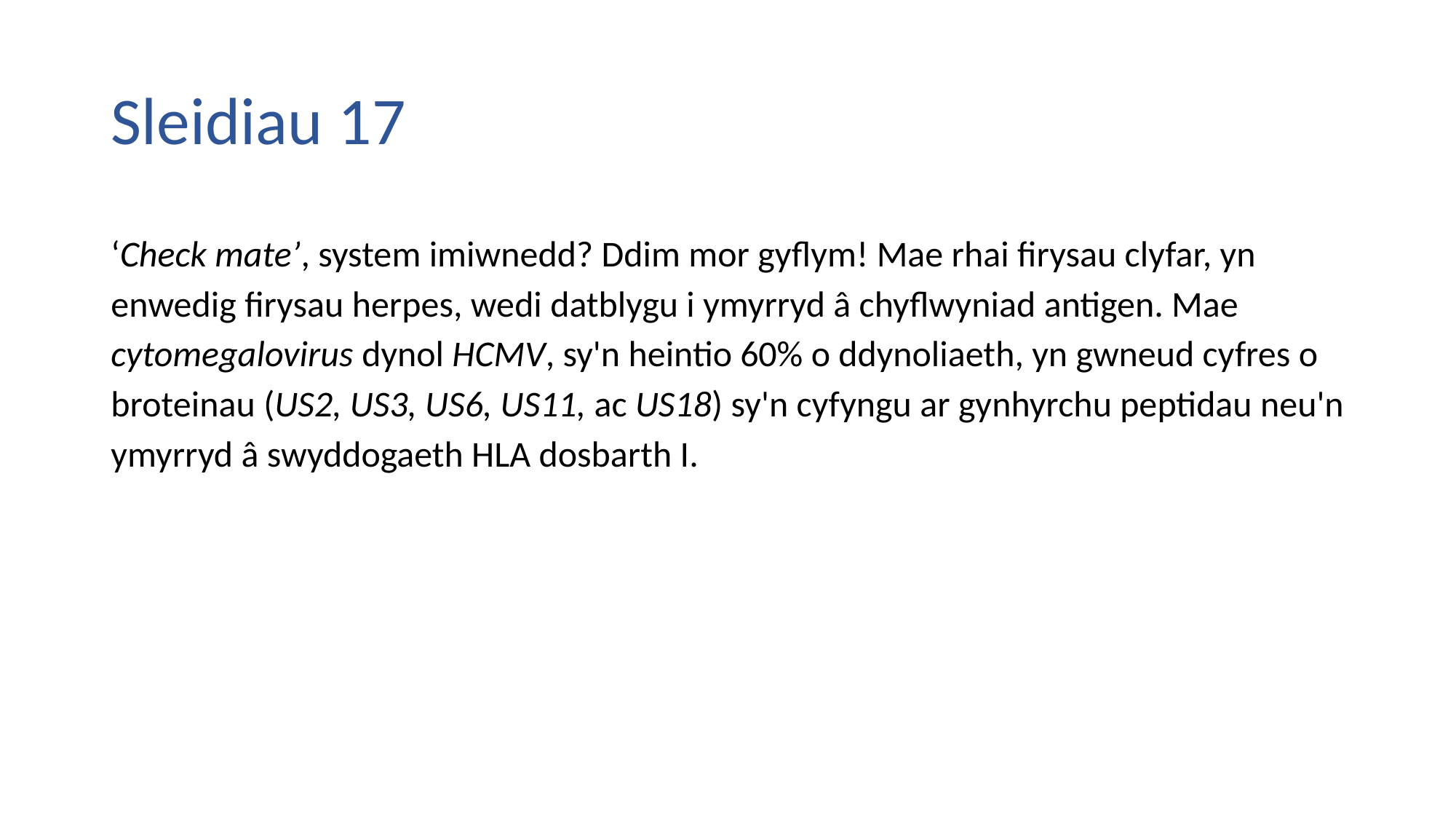

# Sleidiau 17
‘Check mate’, system imiwnedd? Ddim mor gyflym! Mae rhai firysau clyfar, yn enwedig firysau herpes, wedi datblygu i ymyrryd â chyflwyniad antigen. Mae cytomegalovirus dynol HCMV, sy'n heintio 60% o ddynoliaeth, yn gwneud cyfres o broteinau (US2, US3, US6, US11, ac US18) sy'n cyfyngu ar gynhyrchu peptidau neu'n ymyrryd â swyddogaeth HLA dosbarth I.

## Slide 19
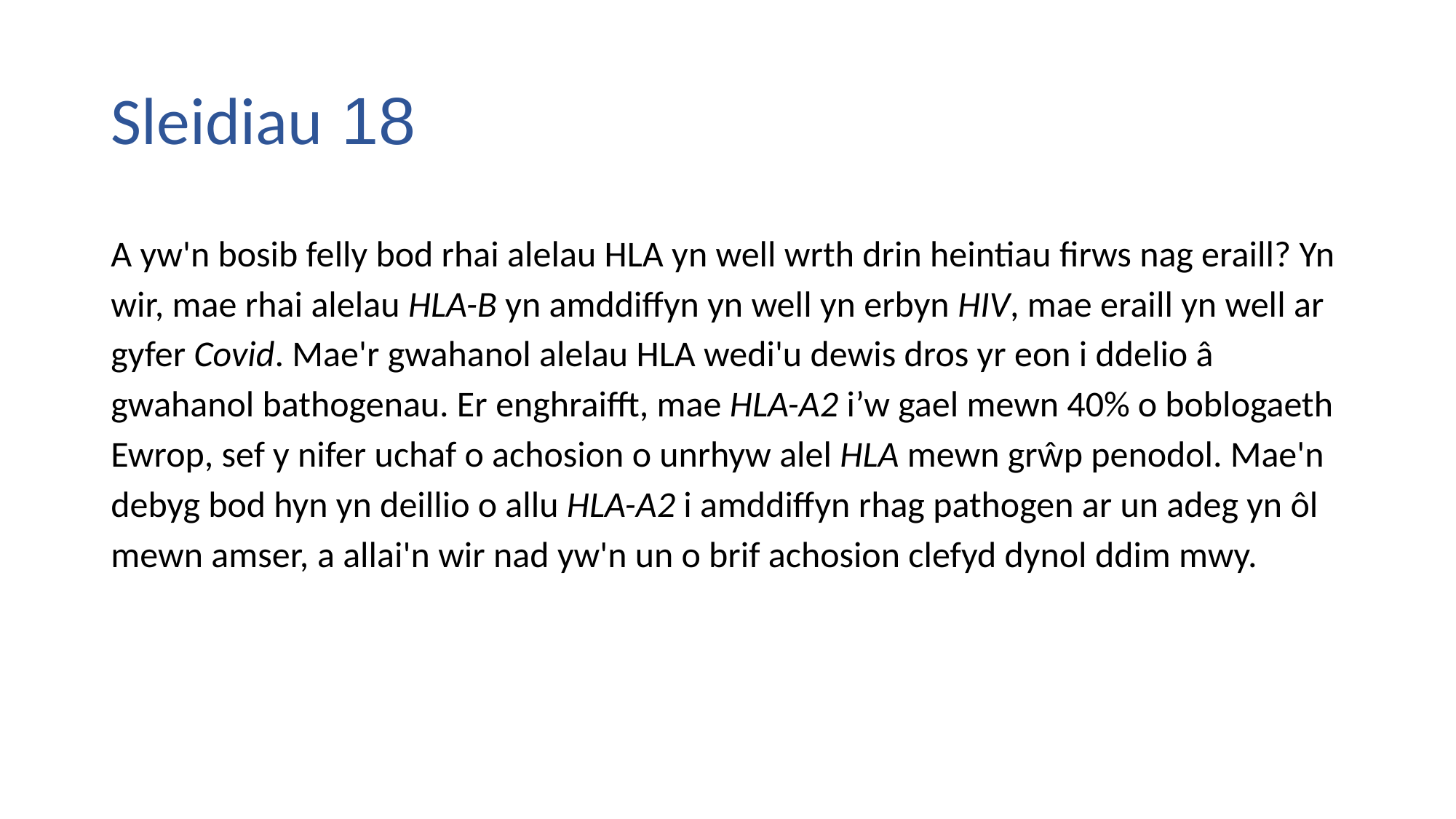

# Sleidiau 18
A yw'n bosib felly bod rhai alelau HLA yn well wrth drin heintiau firws nag eraill? Yn wir, mae rhai alelau HLA-B yn amddiffyn yn well yn erbyn HIV, mae eraill yn well ar gyfer Covid. Mae'r gwahanol alelau HLA wedi'u dewis dros yr eon i ddelio â gwahanol bathogenau. Er enghraifft, mae HLA-A2 i’w gael mewn 40% o boblogaeth Ewrop, sef y nifer uchaf o achosion o unrhyw alel HLA mewn grŵp penodol. Mae'n debyg bod hyn yn deillio o allu HLA-A2 i amddiffyn rhag pathogen ar un adeg yn ôl mewn amser, a allai'n wir nad yw'n un o brif achosion clefyd dynol ddim mwy.

## Slide 20
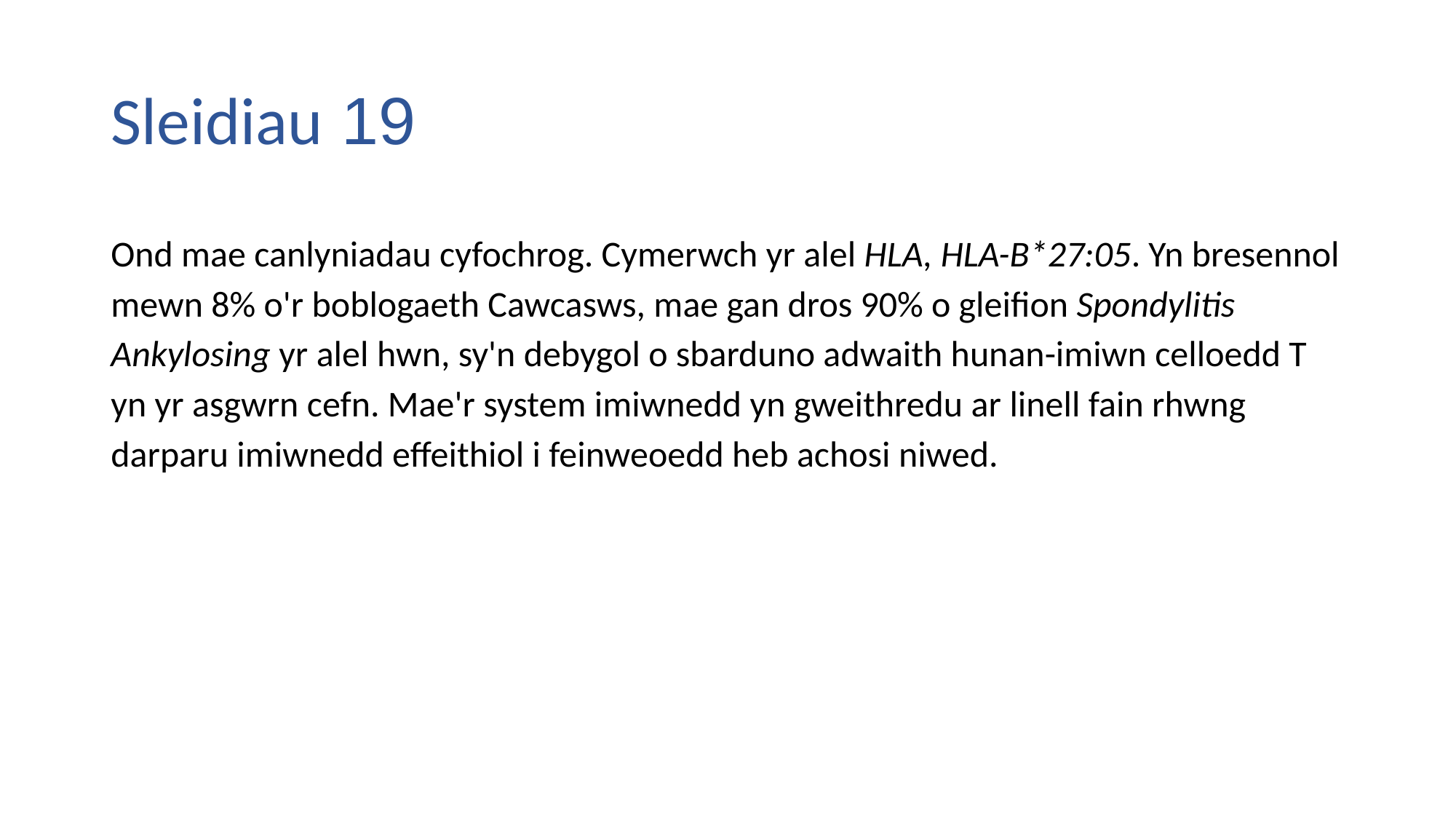

# Sleidiau 19
Ond mae canlyniadau cyfochrog. Cymerwch yr alel HLA, HLA-B*27:05. Yn bresennol mewn 8% o'r boblogaeth Cawcasws, mae gan dros 90% o gleifion Spondylitis Ankylosing yr alel hwn, sy'n debygol o sbarduno adwaith hunan-imiwn celloedd T yn yr asgwrn cefn. Mae'r system imiwnedd yn gweithredu ar linell fain rhwng darparu imiwnedd effeithiol i feinweoedd heb achosi niwed.

## Slide 21
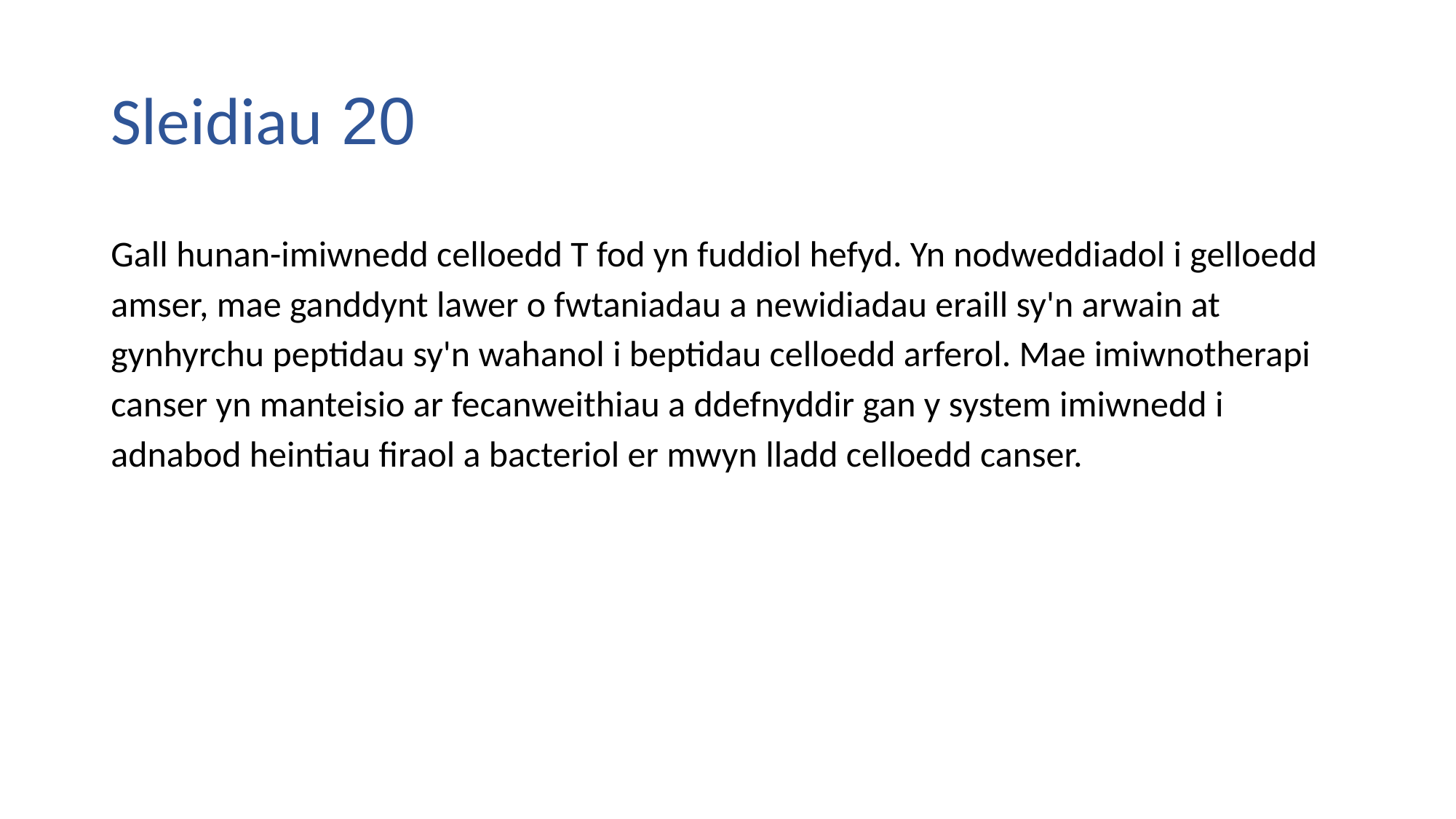

# Sleidiau 20
Gall hunan-imiwnedd celloedd T fod yn fuddiol hefyd. Yn nodweddiadol i gelloedd amser, mae ganddynt lawer o fwtaniadau a newidiadau eraill sy'n arwain at gynhyrchu peptidau sy'n wahanol i beptidau celloedd arferol. Mae imiwnotherapi canser yn manteisio ar fecanweithiau a ddefnyddir gan y system imiwnedd i adnabod heintiau firaol a bacteriol er mwyn lladd celloedd canser.

## Slide 22
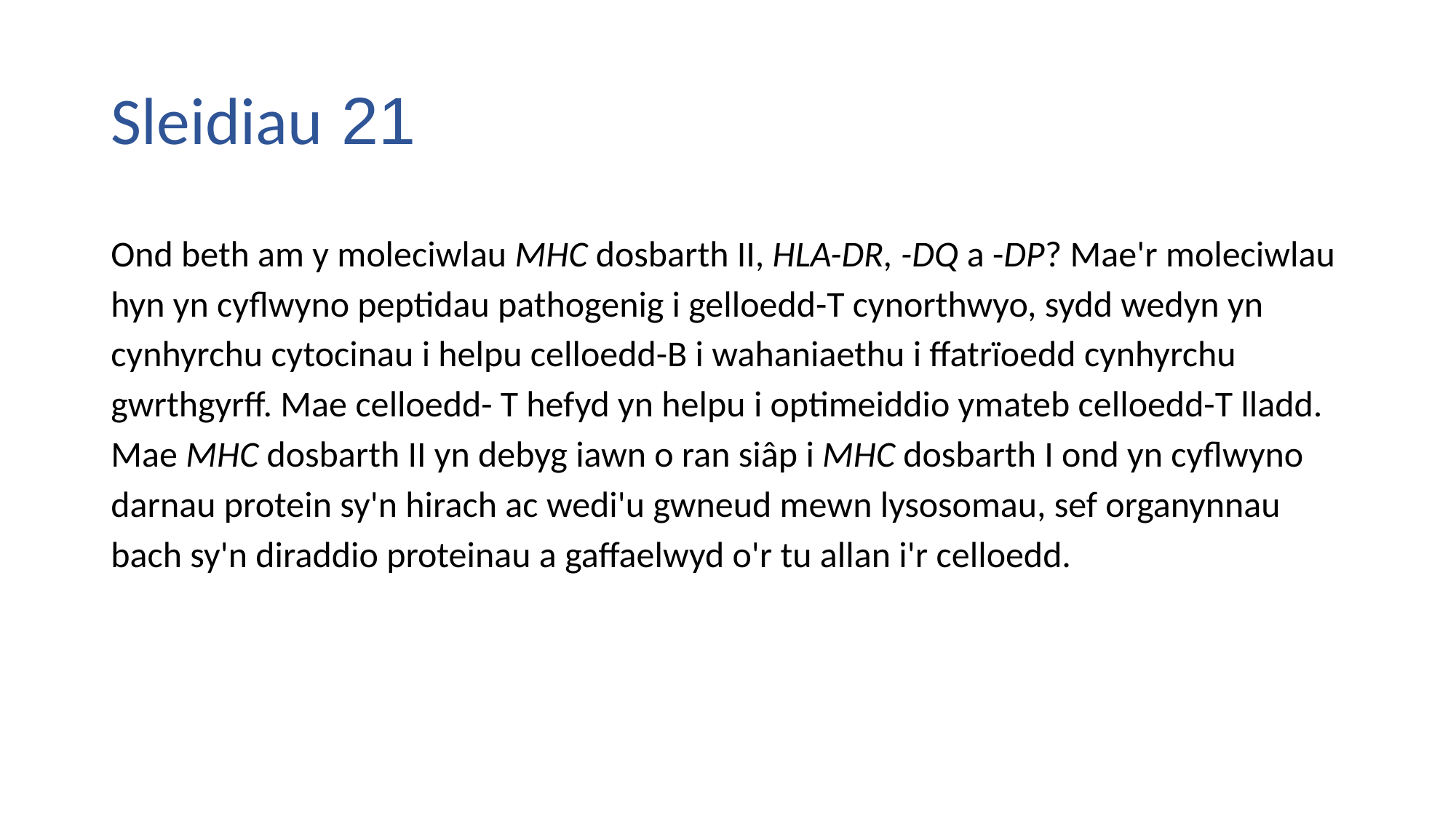

# Sleidiau 21
Ond beth am y moleciwlau MHC dosbarth II, HLA-DR, -DQ a -DP? Mae'r moleciwlau hyn yn cyflwyno peptidau pathogenig i gelloedd-T cynorthwyo, sydd wedyn yn cynhyrchu cytocinau i helpu celloedd-B i wahaniaethu i ffatrïoedd cynhyrchu gwrthgyrff. Mae celloedd- T hefyd yn helpu i optimeiddio ymateb celloedd-T lladd. Mae MHC dosbarth II yn debyg iawn o ran siâp i MHC dosbarth I ond yn cyflwyno darnau protein sy'n hirach ac wedi'u gwneud mewn lysosomau, sef organynnau bach sy'n diraddio proteinau a gaffaelwyd o'r tu allan i'r celloedd.

## Slide 23
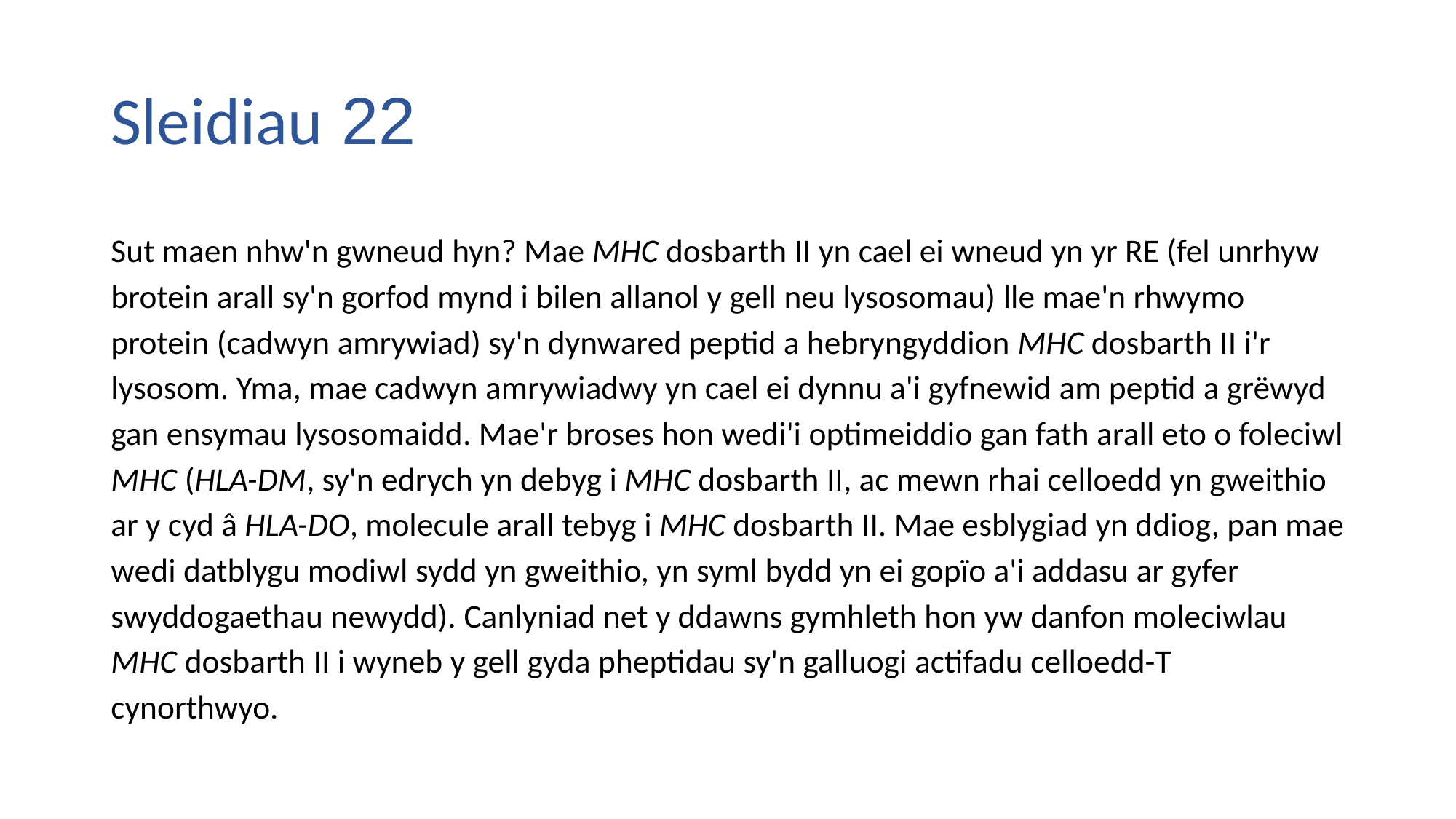

# Sleidiau 22
Sut maen nhw'n gwneud hyn? Mae MHC dosbarth II yn cael ei wneud yn yr RE (fel unrhyw brotein arall sy'n gorfod mynd i bilen allanol y gell neu lysosomau) lle mae'n rhwymo protein (cadwyn amrywiad) sy'n dynwared peptid a hebryngyddion MHC dosbarth II i'r lysosom. Yma, mae cadwyn amrywiadwy yn cael ei dynnu a'i gyfnewid am peptid a grëwyd gan ensymau lysosomaidd. Mae'r broses hon wedi'i optimeiddio gan fath arall eto o foleciwl MHC (HLA-DM, sy'n edrych yn debyg i MHC dosbarth II, ac mewn rhai celloedd yn gweithio ar y cyd â HLA-DO, molecule arall tebyg i MHC dosbarth II. Mae esblygiad yn ddiog, pan mae wedi datblygu modiwl sydd yn gweithio, yn syml bydd yn ei gopïo a'i addasu ar gyfer swyddogaethau newydd). Canlyniad net y ddawns gymhleth hon yw danfon moleciwlau MHC dosbarth II i wyneb y gell gyda pheptidau sy'n galluogi actifadu celloedd-T cynorthwyo.

## Slide 24
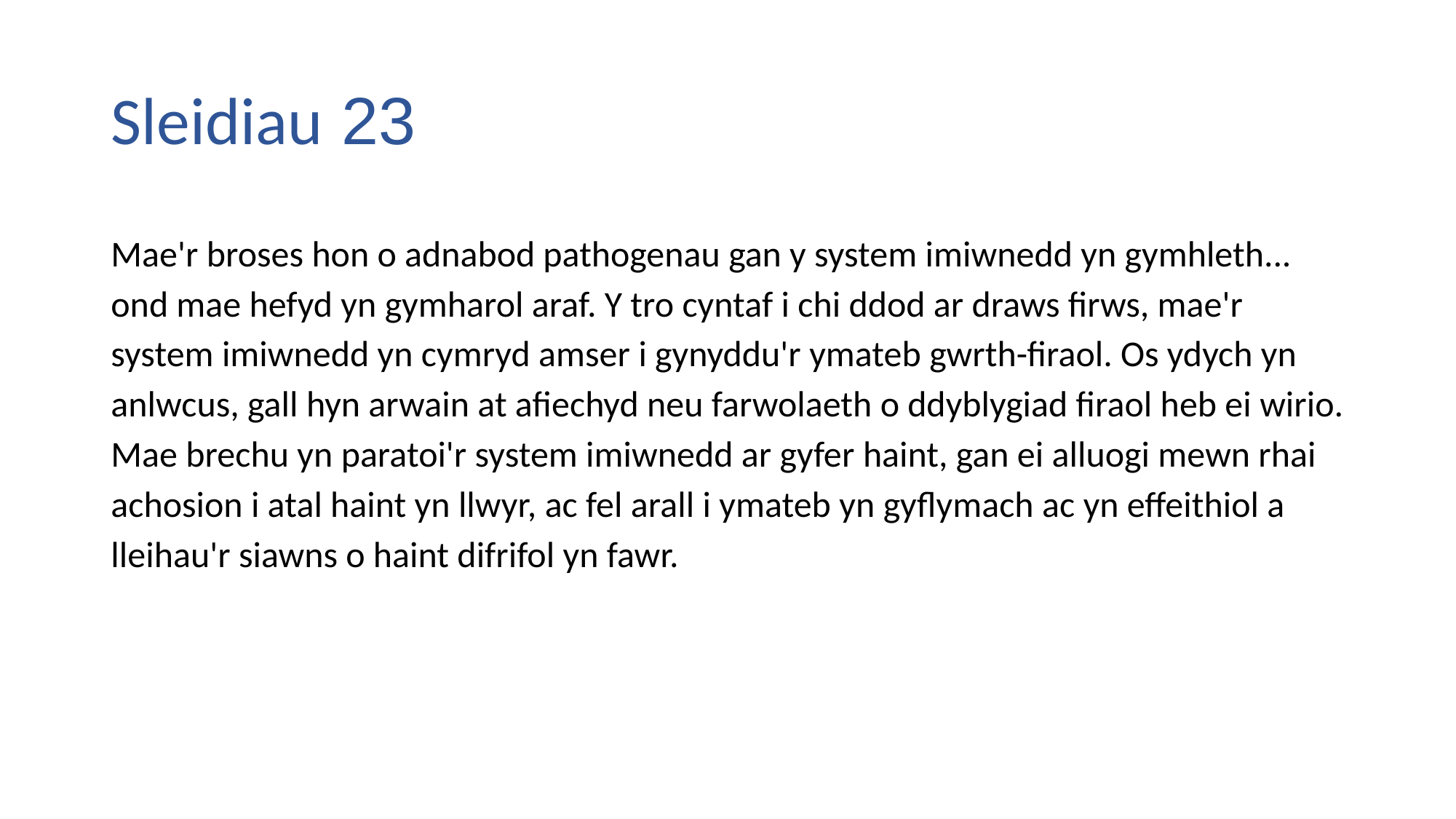

# Sleidiau 23
Mae'r broses hon o adnabod pathogenau gan y system imiwnedd yn gymhleth... ond mae hefyd yn gymharol araf. Y tro cyntaf i chi ddod ar draws firws, mae'r system imiwnedd yn cymryd amser i gynyddu'r ymateb gwrth-firaol. Os ydych yn anlwcus, gall hyn arwain at afiechyd neu farwolaeth o ddyblygiad firaol heb ei wirio. Mae brechu yn paratoi'r system imiwnedd ar gyfer haint, gan ei alluogi mewn rhai achosion i atal haint yn llwyr, ac fel arall i ymateb yn gyflymach ac yn effeithiol a lleihau'r siawns o haint difrifol yn fawr.

## Slide 25
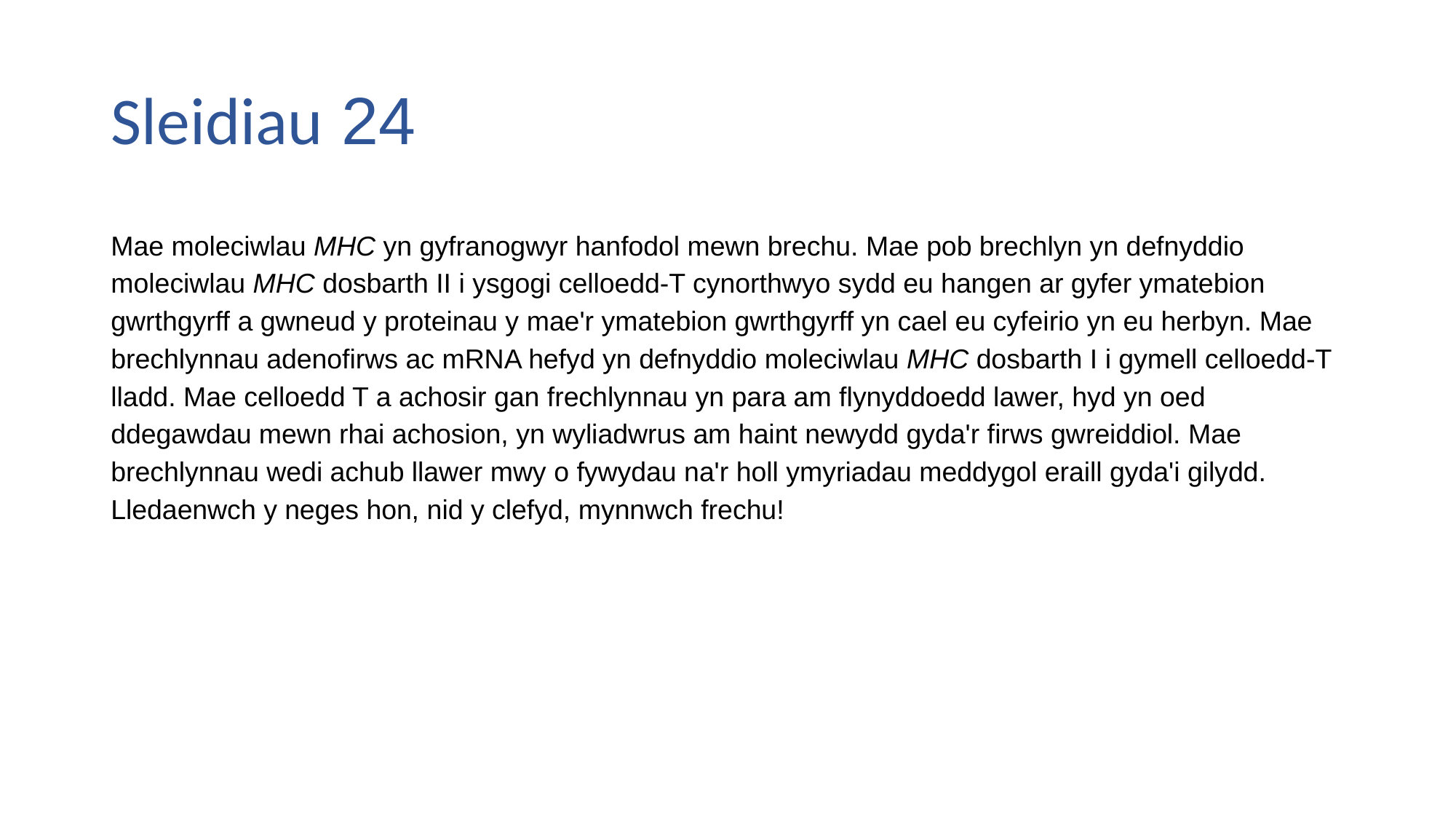

# Sleidiau 24
Mae moleciwlau MHC yn gyfranogwyr hanfodol mewn brechu. Mae pob brechlyn yn defnyddio moleciwlau MHC dosbarth II i ysgogi celloedd-T cynorthwyo sydd eu hangen ar gyfer ymatebion gwrthgyrff a gwneud y proteinau y mae'r ymatebion gwrthgyrff yn cael eu cyfeirio yn eu herbyn. Mae brechlynnau adenofirws ac mRNA hefyd yn defnyddio moleciwlau MHC dosbarth I i gymell celloedd-T lladd. Mae celloedd T a achosir gan frechlynnau yn para am flynyddoedd lawer, hyd yn oed ddegawdau mewn rhai achosion, yn wyliadwrus am haint newydd gyda'r firws gwreiddiol. Mae brechlynnau wedi achub llawer mwy o fywydau na'r holl ymyriadau meddygol eraill gyda'i gilydd. Lledaenwch y neges hon, nid y clefyd, mynnwch frechu!
